# Population attributable fractions of modifiable risk factors for dementia: a systematic review and meta-analysis

**DOI:** 10.1016/S2666-7568(24)00061-8

**Published:** 2024-06

**Authors:** Blossom C M Stephan, Louie Cochrane, Aysegul Humeyra Kafadar, Jacob Brain, Elissa Burton, Bronwyn Myers, Carol Brayne, Aliya Naheed, Kaarin J Anstey, Ammar W Ashor, Mario Siervo

**Affiliations:** a Dementia Centre of Excellence, Curtin enAble Institute, Faculty of Health Sciences, Curtin University, Perth, WA, Australia; b Curtin School of Allied Health, Faculty of Health Sciences, Curtin University, Perth, WA, Australia; c Institute of Mental Health, The University of Nottingham Medical School, Nottingham, UK; d Freemasons Foundation Centre for Men's Health, Discipline of Medicine, School of Psychology, The University of Adelaide, Adelaide, SA, Australia; e Mental Health, Alcohol, Substance Use, and Tobacco Research Unit, South African Medical Research Council, Tygerberg, South Africa; f Division of Addiction Psychiatry, Department of Psychiatry and Mental Health, University of Cape Town, Cape Town, South Africa; g Cambridge Public Health, University of Cambridge, Cambridge, UK; h Non-Communicable Diseases, Nutrition Research Division, International Centre for Diarrhoeal Disease Research, Dhaka, Bangladesh; i UNSW Ageing Futures Institute, University of New South Wales, Sydney, NSW, Australia; j Brain Health and Dementia Centre, Neuroscience Research Australia, Sydney, NSW, Australia; k Department of Internal Medicine, College of Medicine, Mustansiriyah University, Baghdad, Iraq; l School of Population Health, Curtin University, Perth, WA, Australia

## Abstract

**Background:**

More than 57 million people have dementia worldwide. Evidence indicates a change in dementia prevalence and incidence in high-income countries, which is likely to be due to improved life-course population health. Identifying key modifiable risk factors for dementia is essential for informing risk reduction and prevention strategies. We therefore aimed to estimate the population attributable fraction (PAF) for dementia associated with modifiable risk factors.

**Methods:**

In this systematic review and meta-analysis, we searched Embase, MEDLINE, and PsycINFO, via Ovid, from database inception up to June 29, 2023, for population-derived or community-based studies and reviews reporting a PAF value for one or more modifiable risk factor for later-life dementia (prevalent or incident dementia in people aged ≥60 years), with no restrictions on dementia subtype, the sex or baseline age of participants, or the period of study. Articles were independently screened for inclusion by four authors, with disagreements resolved through consensus. Data including unweighted and weighted PAF values (weighted to account for communality or overlap in risk) were independently extracted into a predefined template by two authors and checked by two other authors. When five or more unique studies investigated a given risk factor or combination of the same factors, random-effects meta-analyses were used to calculate a pooled PAF percentage estimate for the factor or combination of factors. The review protocol was registered on PROSPERO, CRD42022323429.

**Findings:**

4024 articles were identified, and 74 were included in our narrative synthesis. Overall, PAFs were reported for 61 modifiable risk factors, with sufficient data available for meta-analysis of 12 factors (n=48 studies). In meta-analyses, the highest pooled unweighted PAF values were estimated for low education (17·2% [95% CI 14·4–20·0], p<0·0001), hypertension (15·8% [14·7–17·1], p<0·0001), hearing loss (15·6% [10·3–20·9], p<0·0001), physical inactivity (15·2% [12·8–17·7], p<0·0001), and obesity (9·4% [7·3–11·7], p<0·0001). According to weighted PAF values, low education (9·3% [6·9–11·7], p<0·0001), physical inactivity (7·3% [3·9–11·2], p=0·0021), hearing loss (7·2% [5·2–9·7], p<0·0001), hypertension (7·1% [5·4–8·8], p<0·0001), and obesity (5·3% [3·2–7·4], p=0·0001) had the highest pooled estimates. When low education, midlife hypertension, midlife obesity, smoking, physical inactivity, depression, and diabetes were combined (Barnes and Yaffe seven-factor model; n=9 studies), the pooled unweighted and weighted PAF values were 55·0% (46·5–63·5; p<0·0001) and 32·0% (26·6–37·5; p<0·0001), respectively. The pooled PAF values for most individual risk factors were higher in low-income and middle-income countries (LMICs) versus high-income countries.

**Interpretation:**

Governments need to invest in a life-course approach to dementia prevention, including policies that enable quality education, health-promoting environments, and improved health. This investment is particularly important in LMICs, where the potential for prevention is high, but resources, infrastructure, budgets, and research focused on ageing and dementia are limited.

**Funding:**

UK Research and Innovation (Medical Research Council).

## Introduction

Dementia is a public health priority with more than 57 million people living with dementia worldwide.^1^ In the absence of a cure and given recent reductions in both prevalent and incident dementia in high-income countries (HICs),^2^ the development of risk reduction and prevention programmes has become a key focus. However, effective dementia prevention requires an understanding of modifiable risk factors, in terms of evolution across the life course, strengths of associations, patterns of clustering in different populations, time windows for modifying risk, and whether the effects of risk factors are analogous across different regions. Only with this understanding can effective, context-specific risk reduction strategies be developed and implemented.

In 2020, a *Lancet* Commission reported on 12 factors across the life course that had a combined population attributable fraction (PAF) for dementia worldwide of around 40%.^3^ These factors were low educational attainment in early life (age <45 years); hearing loss, traumatic brain injury, hypertension, increased alcohol intake, and obesity in midlife (age 45–65 years); and smoking, depression, social isolation, physical inactivity, air pollution, and diabetes in later life (age >65 years). Replication of this analysis in low-income and middle-income countries (LMICs), comprising nine of the 12 factors (low education, hearing loss, hypertension, obesity, smoking, depression, physical inactivity, social isolation, and diabetes), highlighted an even greater potential for risk reduction in these settings, with combined PAFs of 56% in Latin America, 41% in India, and 40% in China.^4^ Other studies have estimated the PAFs for different modifiable risk factors, with variable results.^5^, ^6^, ^7^ Calculation of PAF values requires a robust evidence base, including epidemiological evidence of an association between a risk factor and dementia; systematic reviews and meta-analyses quantifying the overall effect of a given risk factor for dementia; knowledge of potential interactions between risk factors; and data on risk factor prevalence. Given differences in the extent to which risk factors have been studied and the reported variability in PAF estimates, there is a need to synthesise the evidence.

**Research in context**

**Evidence before this study**

Dementia is a major public health priority requiring urgent action. The *Lancet* Commission on dementia prevention, intervention, and care (2020) reported that up to approximately 40% of dementia cases globally could be prevented or delayed by addressing key modifiable sociodemographic (ie, education), health, lifestyle, and environmental factors across the life course. Other studies have also calculated the population attributable fraction (PAF) estimates for different modifiable risk factors and their combination, highlighting the potential impact of prevention efforts. We therefore searched PROSPERO and PubMed, from database inception to June 6, 2022, using terms for dementia (and its subtypes; eg, Alzheimer's disease) and PAF, for other studies that had investigated the potential impact of prevention efforts. There were no restrictions on language, study design, or publication date. We found many studies, including reviews and cross-sectional and cohort studies, that had calculated PAF estimates for a variety of different modifiable risk factors for dementia and their combination in both high-income countries (HICs) and low-income and middle-income countries (LMICs). Across these studies, the PAF estimates varied and there did not appear to be consistency in which factors (and their combination) had been assessed. Furthermore, we found no review that had synthesised this literature.

**Added value of this study**

This systematic review and meta-analysis quantified the proportion of dementia cases that could be potentially prevented if modifiable risk factors were eliminated. From 61 modifiable risk factors with previously reported PAFs, 12 were meta-analysed. Among these factors, low education, physical inactivity, hearing loss, hypertension, and obesity were the most prominent risk factors for dementia, with pooled weighted PAF percentage estimates (weighted for communality or overlap in risk) ranging across the 12 factors from 9·3% (95% CI 6·9–11·7) for low education to 0·2% (0·0–0·5) for alcohol consumption. Compared with HICs, LMICs had higher unweighted and weighted PAF values for education and hearing loss.

**Implications of all the available evidence**

Given the epidemic scale of dementia forecasted in the next 20 years and the empirical evidence of the potential to reduce the incidence and prevalence of dementia in populations, research focused on mitigating the impacts is crucial. Urgent investment is needed into the development of a comprehensive preventive approach that involves policies and strategies to address the key modifiable risk factors unique to each setting. This investment is especially the case in LMICs, where the burden of dementia and its risk factors is greatest and investment in dementia and dementia prevention research is lowest.

We therefore sought to estimate the PAFs of modifiable dementia risk factors (independently and in combination) and assess the consistency of PAF values for a given risk factor across HIC and LMIC settings. Collating the PAF evidence will reveal which factors to target to ensure the greatest potential reduction in dementia cases. Clarifying variability across income setting will guide the development of dementia interventions and risk reduction policy at the local level.

## Methods

### Search strategy and selection criteria

We did a systematic review and meta-analysis in accordance with PRISMA guidelines^8^ (checklist provided in the appendix [pp 2–4]). We searched Embase, PsycINFO, and MEDLINE, using Ovid, covering all published literature from database inception up to June 29, 2023. The search included terms for dementia and its subtypes and terms for PAF. The search strategy is detailed in the appendix (p 5).

Population-derived or community-based studies (cross-sectional or longitudinal) and reviews (including systematic reviews and meta-analysis) were eligible for inclusion. Studies in specific populations (eg, people with diabetes, or veterans) were excluded. In addition, trials, opinion pieces, editorials, consensus statements, book chapters, methodology papers, predictive studies, and conference abstracts were excluded. Studies must have reported a PAF value for one or more modifiable risk factor for dementia in people aged 60 years or older, with no restriction on the type of risk factor (eg, health or lifestyle). If only combined PAF values including non-modifiable factors were available, the study was excluded. Studies that assessed either prevalent or incident dementia were eligible. There were no restrictions on language, date of publication, dementia subtype, the sex of participants, or the period of study. We did not restrict on baseline age (ie, to capture risk factors assessed throughout the life course) but the study was required to report later-life dementia estimates (age ≥60 years).

Four authors (LC, AHK, BCMS, and MS) independently screened titles and abstracts for eligible articles. Subsequently, the full texts of articles deemed eligible by initial screening were retrieved, and these texts were independently screened for inclusion (LC, AHK, BCMS, and MS). The reference lists of the screened full-text articles were checked to identify studies missing from the electronic search. Any disagreements were resolved through consensus. Non-English articles were translated or extracted by native speakers.

### Data analysis

Data extraction was done independently by two authors (LC and AHK) and checked by two authors (JB and EB). Information extracted included the lead author, publication year, study design (including location), sample size when available, dementia outcome investigated (eg, all-cause dementia, Alzheimer's disease, or vascular dementia, which, depending on study design, could be prevalent or incident cases), modifiable risk factors investigated, and risk factor PAF percentage estimates (with 95% CIs; for individual and combined modifiable risk factors). The results from the electronic search were imported into EndNote (version 20) and duplicates removed with use of the in-built function.

The outcome was the PAF, which provides an estimation of the proportion of disease, in a population, that can be attributed to a risk factor (formula provided in the appendix [p 6]). Information on individual unweighted PAF values and combined values (with their 95% CIs) was extracted. Combined PAF values are important as they highlight the multifactorial nature of dementia risk and the potential of prevention strategies addressing co-occurring risk factors. When studies reported PAF values for both modifiable factors and non-modifiable (eg, genetic) factors, only individual data for modifiable risk factors were extracted. If the combined PAF included non-modifiable factors, this was not extracted. When available, the weighted PAF values were also extracted. Weighted PAF values account for communality or overlap in risk. If secular trends in PAF values were reported, only the most up-to-date estimates were extracted to increase the relevance of the data. When PAF values were calculated in the context of studies that had been updated (eg, Livingston et al^3^, ^9^), only the most recent published values were included in the meta-analysis. If studies reported previously published findings and built on these, only unique findings of each paper were extracted and used in the meta-analysis. We also did not extract forecasted data.

Two authors (LC and MS) completed risk of bias assessments for all included studies. Different scales were used depending on study design, including the Newcastle-Ottawa Scale for cohort studies,^10^ the Measurement Tool to Assess Systematic Reviews 2 for systematic reviews and meta-analyses,^11^ and the Scale for the Assessment of Narrative Review Articles for non-systematic reviews.^12^ On the basis of the cutoffs for each scale, articles were classified as being of critically low, low, moderate, or high quality (appendix p 7).

A descriptive synthesis was done to summarise the study populations and risk factors for dementia as well as their estimated PAF (individual and combined) values, with a focus on combined PAF values (for combinations of ≥2 risk factors) at the global and regional levels. When available, we also extracted and reported individual and combined PAF values stratified by ethnicity. We also qualitatively compared PAF values by ethnic subgroups for individual PAF results representing the 12 risk factors reported in the *Lancet* Commission,^3^ as well as combined PAF values for any modifiable factors including those not reported in the *Lancet* Commission. Given the variability in how risk factors were measured, we present estimates for lifetime depression, as well as the previously reported risk factor of later-life depression.^3^ When five or more unique studies investigated a given risk factor or combination of the same factors, the results were pooled and a meta-analysis was done. We chose a cutoff of five studies to ensure validity of the results. The meta-analysis required the PAF estimates and their 95% CIs. When a study did not report the 95% CIs for PAFs for individual risk factors, but there was sufficient information (ie, the odds ratio, hazard ratio, or relative risk estimate with a 95% CI and the risk factor's prevalence) the 95% CIs for PAF were calculated as outlined in the appendix (p 6). If information was insufficient to calculate a missing 95% CI, the study was excluded from the meta-analysis.

Meta-analysis was done with use of the Meta-Analysis module of SPSS software (version 28.0). Pooled PAF percentage values were calculated with 95% CIs with use of random-effect models and standard errors of all analyses were adjusted with the truncated Knapp-Hartung method to provide conservative pooled estimates of the analyses that included a small number of studies or were characterised by small sample size. Separate analyses were run for each risk factor stratified by weighting (ie, unweighted *vs* weighted). Forest plots were generated for all dementia types combined and stratified by dementia type and study site (global, HIC, or LMIC). Country income level was defined according to World Bank income group classification based on the year of baseline assessment in each study for both cross-sectional and longitudinal designs; for reviews, we used the review-specific criteria for classifying countries, unless the information was not available, in which case World Bank classification was used based on publication date. For meta-analysis of a given combination of risk factors (in ≥5 studies), pooled PAF estimates were presented for all dementia types combined and by dementia type, stratified by weighting. Sample sizes for pooled estimates are not reported as samples were not reported consistently across studies (some PAF estimates based on literature reviews rather than raw data without a sample size value, and some studies that used raw data did not always report sample size). We report the number of PAF estimates from which pooled estimates were derived. Heterogeneity was evaluated with the χ^2^ test and quantified with the *I*^2^ statistic. Publication bias was assessed with the Egger's regression test and visualised with funnel plots. A sensitivity analysis was done to evaluate the influence of low-quality studies on the unweighted pooled estimates for each factor by excluding low-quality studies and repeating the meta-analyses.

We used 95% CIs to describe the statistical precision of our estimates and a p value of less than 0·05 was used to indicate statistical significance.

Ethical approval was sought from the Division of Psychiatry & Applied Psychology Research Ethics Committee at Nottingham University (Nottingham, UK). As the research was a systematic review and meta-analysis the committee recommended the project without the requirement of ethical approval. The review protocol was registered on PROSPERO, CRD42022323429.

### Role of the funding source

The funder of the study had no role in study design, data collection, data analysis, data interpretation, or writing of the report.

## Results

**Figure 1:**
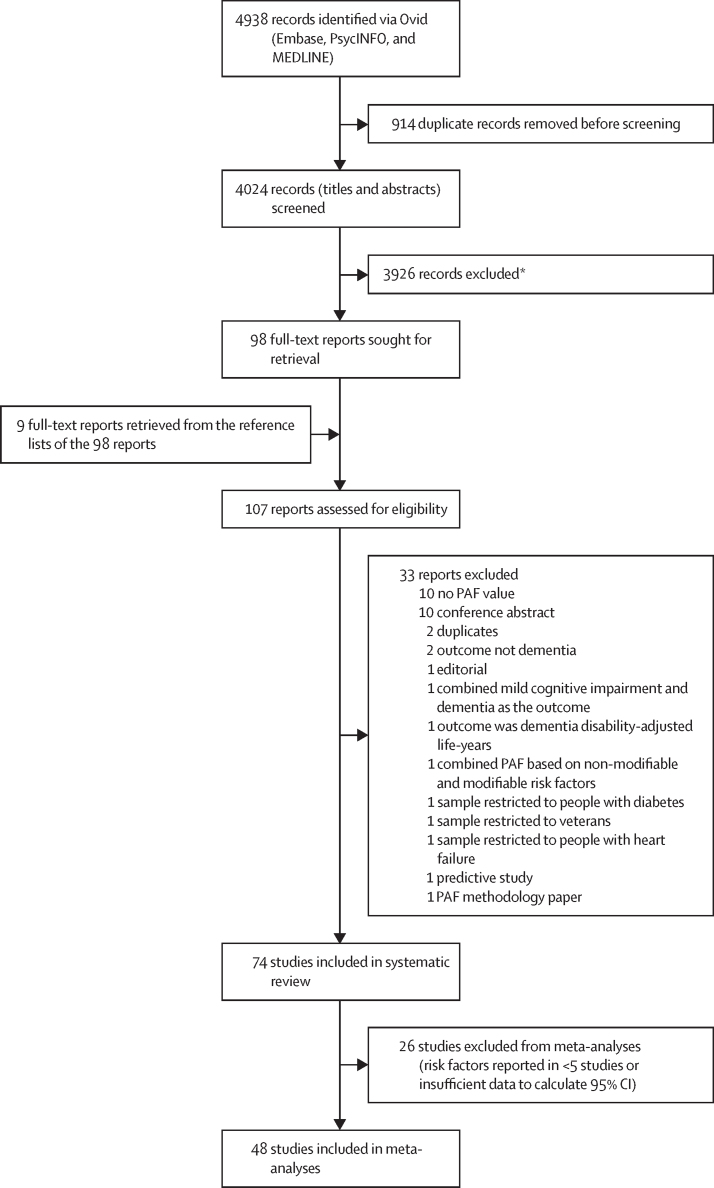
PRISMA flow diagram PAF=population attributable fraction. *Based on title and abstract screening, papers were outside the scope of the review (eg, did not focus on dementia as the outcome, did not investigate modifiable risk factors, or did not calculate a PAF value).

4024 articles were identified from the electronic search after the removal of duplicates (n=914). After title and abstract screening, 98 articles were identified for full-text review, and from these articles an additional nine articles were identified from reference lists. From full-text assessment (n=107), 74 articles were deemed to be eligible for inclusion (figure 1).^3^, ^4^, ^5^, ^6^, ^7^, ^9^, ^13^, ^14^, ^15^, ^16^, ^17^, ^18^, ^19^, ^20^, ^21^, ^22^, ^23^, ^24^, ^25^, ^26^, ^27^, ^28^, ^29^, ^30^, ^31^, ^32^, ^33^, ^34^, ^35^, ^36^, ^37^, ^38^, ^39^, ^40^, ^41^, ^42^, ^43^, ^44^, ^45^, ^46^, ^47^, ^48^, ^49^, ^50^, ^51^, ^52^, ^53^, ^54^, ^55^, ^56^, ^57^, ^58^, ^59^, ^60^, ^61^, ^62^, ^63^, ^64^, ^65^, ^66^, ^67^, ^68^, ^69^, ^70^, ^71^, ^72^, ^73^, ^74^, ^75^, ^76^, ^77^, ^78^, ^79^, ^80^ Key characteristics of the included studies, including sample sizes when available, and a descriptive overview of PAF values are presented in the appendix (pp 8–18). Most studies reported PAFs for all-cause dementia (n=61), followed by Alzheimer's disease (n=15) or Alzheimer's disease and related dementias (n=1; total number on Alzheimer's disease, n=16), and vascular dementia (n=3). 18 studies reported global PAFs with the remaining studies covering individual countries, regions, or stratifying by income level. When individual country PAF values were reported (25 different countries in total), the majority of countries studied were HICs (the USA, n=16 studies; the UK, n=9; Denmark, n=3; Sweden, n=3; Australia, n=2; Italy, n=2; Japan, n=2; and one study each for Barbados, Canada, Chile, Germany, Hong Kong, the Netherlands, New Zealand, Norway, Portugal, South Korea, and Spain). 16 studies reported on upper-middle-income countries including China (n=8), Brazil (n=5), and one study each on Iran, Jamaica, and South Africa. One study reported on a lower-middle-income country (India) and one on a low-income country (Mozambique). Three studies reported PAF values at the global, regional, or income level that represented more than 50 individual countries; the countries are listed separately in the appendix (pp 19–21).

We scored each study on risk of bias (appendix pp 22–26). Based on the summary score, most studies (n=44) were rated as high quality, followed by moderate quality (n=21), low quality (n=7), and critically low quality (n=2). Of 55 studies that calculated PAF values using cohort study data, 35 (63·6%) were deemed to have used representative sources.

PAFs were reported for 61 different modifiable risk factors covering socioeconomic, social, lifestyle, environmental, and health-related factors, medication and drug use, and blood-based biomarkers (appendix p 27). 31 (42%) of 74 studies reported weighted PAFs (for individual or combined risk factors, or both). Sufficient data for meta-analysis was available for 12 factors (n=48 studies). These factors were low education, hearing loss, traumatic brain injury, hypertension, alcohol consumption, obesity, smoking, depression (as two factors: lifetime depression and later-life depression), social isolation, physical inactivity, and diabetes. The appendix (pp 28–33) provides a description of the measurement of each risk factor included in the meta-analysis across the different studies.

In meta-analyses based on unweighted estimates, the top five risk factors for dementia were low education (PAF 17·2% [95% CI 14·4–20·0], p<0·0001), hypertension (PAF 15·8% [14·7–17·1], p<0·0001), hearing loss (PAF 15·6% [10·3–20·9], p<0·0001), physical inactivity (PAF 15·2% [12·8–17·7], p<0·0001), and obesity (PAF 9·4% [7·3–11·7], p<0·0001; table 1). Alcohol consumption had the lowest unweighted PAF (0·8% [0·4–1·2], p=0·0008). The numbers of PAF estimates from which pooled estimates were derived are listed in table 1. Heterogeneity was high for all risk factors, with *I*^2^ varying between 77% (alcohol consumption) and 100% (traumatic brain injury, social isolation, and hearing loss; χ^2^ p<0·0001 for all factors; table 1). When stratified by country income level, we found that overall, LMICs had higher pooled PAF values than HICs for most risk factors. For depression and social isolation, pooled PAFs were higher in HICs than LMICs (table 1). When considering dementia subtype, the role of low education was greater for vascular dementia (PAF 31·8% [24·0–39·5]) than for Alzheimer's disease (12·6% [9·9–15·3]), whereas physical inactivity appeared to be associated with greater risk of Alzheimer's disease (18·1% [14·0–22·2]) than vascular dementia (15·8% [2·7–29·0]; table 1). The appendix (pp 34–59) presents forest plots of the unweighted pooled PAF estimates for each factor overall and stratified by country income level and dementia subtype. The sensitivity analyses showed that the removal of seven studies rated as low quality did not modify the unweighted results apart from for hypertension, with the pooled PAF value changing from 15·8% (14·7–17·1) to 10·8% (8·6–13·1; appendix p 60). According to weighted pooled estimates, low education was associated with the highest risk for dementia (PAF 9·3% [6·9–11·7], p<0·0001) followed by physical inactivity (PAF 7·3% [3·9–11·2], p=0·0021), hearing loss (PAF 7·2% [5·2–9·7], p<0·0001), hypertension (PAF 7·1% [5·4–8·8], p<0·0001), and obesity (PAF 5·3% [3·2–7·4], p=0·0001; table 1). Alcohol consumption had the lowest weighted PAF (0·2% [0·0–0·5], p=0·072). Heterogeneity (*I*^2^) ranged from 20% (traumatic brain injury; χ^2^ p=0·27) to 100% (physical inactivity, social isolation, and later-life depression; χ^2^ p values <0·0001). Forest plots summarising the weighted pooled PAF values for each factor are shown in the appendix (pp 61–73). Funnel plots and Egger's regression test results to assess publication bias of the PAFs for individual risk factors (unweighted and weighted) are also shown in the appendix (pp 74–98). For unweighted pooled estimates, all factors showed significant publication bias except for alcohol consumption and later-life depression. For weighted pooled estimates, all factors showed significant publication bias except for alcohol consumption, depression (lifetime and later life), and social isolation.

**Table 1:** Meta-analysis results of PAF for each individual risk factor, overall and stratified by country income level and dementia subtype

		**Unweighted**				**Weighted**			
		Number of estimates	Pooled PAF, % (95% CI)	p value	*I*^2^, % (χ^2^ p value)	Number of estimates	Pooled PAF, % (95% CI)	p value	*I*^2^, % (χ^2^ p value)
**Low education**									
Overall		41	17·2% (14·4 to 20·0)	<0·0001	99% (p<0·0001)	11	9·3% (6·9 to 11·7)	<0·0001	98% (p<0·0001)
Global status									
	Global	6	15·6% (9·4 to 21·8)	0·0017	..	0	NA	NA	..
	HIC	21	12·8% (9·9 to 15·8)	<0·0001	..	2	5·7% (−9·8 to 21·2)	0·13	..
	LMIC	14	23·7% (18·4 to 29·1)	<0·0001	..	9	9·7% (7·1 to 12·3)	<0·0001	..
Dementia type									
	All-cause dementia	26	18·8% (14·8 to 22·9)	<0·0001	..	11	9·3% (6·9 to 11·7)	<0·0001	..
	Alzheimer's disease	14	12·6% (9·9 to 15·3)	<0·0001	..	0	NA	NA	..
	Vascular dementia	1	31·8% (24·0 to 39·5)	<0·0001	..	0	NA	NA	..
**Hearing loss**									
Overall		17	15·6% (10·3 to 20·9)	<0·0001	100% (p<0·0001)	9	7·2% (5·2 to 9·7)	<0·0001	97% (p<0·0001)
Global status									
	Global	1	22·2% (10·3 to 34·0)	<0·0001	..	0	NA	NA	..
	HIC	9	8·9% (3·6 to 14·2)	0·0046	..	1	5·1% (2·9 to 7·3)	0·0009	..
	LMIC	7	21·5% (12·6 to 30·5)	0·0010	..	8	7·4% (5·2 to 9·7)	0·0001	
Dementia type									
	All-cause dementia	16	16·6% (11·3 to 21·9)	<0·0001	..	9	7·2% (5·2 to 9·7)	<0·0001	..
	Alzheimer's disease	1	2·3% (2·2 to 2·3)	<0·0001	..	0	NA	NA	..
	Vascular dementia	0	NA	NA	..	0	NA	NA	..
**Traumatic brain injury**									
Overall		31	2·1% (0·8 to 3·3)	0·0020	100% (p<0·0001)	2	3·2% (−2·0 to 8·5)	0·080	20% (p=0·27)
Global status									
	Global	3	3·2% (−7·9 to 14·3)	0·34	..	0	..	..	..
	HIC	13	4·2% (1·2 to 7·2)	0·0088	..	1	4·3% (2·2 to 6·3)	0·0017	..
	LMIC	15	0·6% (0·2 to 1·0)	0·0086	..	1	3·1% (2·6 to 3·5)	0·0021	..
Dementia type									
	All-cause dementia	31	2·1% (0·8 to 3·3)	0·0020	..	2	3·2% (−2·0 to 8·5)	0·080	..
	Alzheimer's disease	0	NA	NA	..	0	NA	NA	..
	Vascular dementia	0	NA	NA	..	0	NA	NA	..
**Hypertension**									
Overall		227	15·8% (14·7 to 17·1)	<0·0001	97% (p<0·0001)	10	7·1% (5·4 to 8·8)	<0·0001	94% (p<0·0001)
Global status									
	Global	4	3·3% (−1·3 to 8·0)	0·11	..	0	NA	NA	..
	HIC	76	13·9% (12·6 to 15·2)	<0·0001	..	2	10·5% (−9·4 to 30·5)	0·094	..
	LMIC	147	17·0% (16·3 to 17·7)	<0·0001	..	8	6·8% (5·0 to 8·5)	<0·0001	..
Dementia type									
	All-cause dementia	213	16·5% (15·3 to 17·6)	<0·0001	..	10	7·1% (5·4 to 8·8)	<0·0001	..
	Alzheimer's disease	13	6·5% (4·1 to 8·8)	<0·0001	..	0	NA	NA	..
	Vascular dementia	1	6·6% (1·8 to 11·3)	<0·0001	..	0	NA	NA	..
**Alcohol consumption**									
Overall		10	0·8% (0·4 to 1·2)	0·0008	77% (p<0·0001)	4	0·2% (0·0 to 0·5)	0·072	61% (p=0·072)
Global status									
	Global	1	2·1% (0·9 to 3·2)	0·0075	..	0	NA	NA	..
	HIC	6	0·7% (0·2 to 1·2)	0·012	..	1	0·3% (−0·2 to 0·8)	0·28	..
	LMIC	3	0·7% (−0·3 to 1·7)	0·10	..	3	0·2% (−0·2 to 0·8)	0·16	..
Dementia type									
	All-cause dementia	10	0·8% (0·4 to 1·2)	0·0008	..	4	0·2% (0·0 to 0·5)	0·072	..
	Alzheimer's disease	0	NA	NA	..	0	NA	NA	..
	Vascular dementia	0	NA	NA	..	0	NA	NA	..
**Obesity**									
Overall		40	9·4% (7·3 to 11·7)	<0·0001	99% (p<0·0001)	9	5·3% (3·2 to 7·4)	0·0001	97% (p<0·0001)
Global status									
	Global	5	2·1% (1·4 to 2·7)	0·0008	..	0	NA	NA	..
	HIC	22	9·9% (7·2 to 12·6)	<0·0001	..	1	6·3% (3·8 to 8·8)	0·0014	..
	LMIC	13	11·7% (6·8 to 16·8)	0·0002	..	8	5·3% (2·8 to 7·7)	0·0012	..
Dementia type									
	All-cause dementia	24	11·8% (8·7 to 14·9)	<0·0001	..	9	5·3% (3·2 to 7·4)	0·0001	..
	Alzheimer's disease	15	6·4% (3·6 to 9·2)	0·0002	..	0	NA	NA	..
	Vascular dementia	1	2·3% (−0·2 to 4·8)	0·069	..	0	NA	NA	..
**Smoking**									
Overall		42	9·1% (7·2 to 11·0)	<0·0001	97% (p<0·0001)	9	4·5% (3·2 to 5·9)	<0·0001	94% (p<0·0001)
Global status									
	Global	7	14·6% (5·5 to 23·8)	0·0078	..	0	NA	NA	..
	HIC	20	6·8% (4·8 to 8·9)	<0·0001	..	1	4·1% (2·1 to 6·1)	0·0014	..
	LMIC	15	9·7% (6·2 to 13·2)	<0·0001	..	8	4·6% (3·1 to 5·9)	0·0001	..
Dementia type									
	All-cause dementia	24	8·3% (5·9 to 10·9)	<0·0001	..	9	4·5% (3·2 to 5·9)	<0·0001	..
	Alzheimer's disease	17	10·8% (7·4 to 14·0)	<0·0001	..	0	NA	NA	..
	Vascular dementia	1	6·5% (1·3 to 11·7)	0·014	..	0	NA	NA	..
**Depression, lifetime**									
Overall		28	8·1% (6·4 to 9·8)	<0·0001	97% (p<0·0001)	5	2·5% (0·7 to 4·3)	0·016	90% (p<0·0001)
Global status									
	Global	7	5·6% (1·8 to 9·4)	0·011	..	0	NA	NA	..
	HIC	14	9·9% (8·1 to 11·8)	<0·0001	..	1	1·9% (0·5 to 3·3)	0·016	..
	LMIC	7	6·1% (2·8 to 9·3)	0·0039	..	4	2·7% (0·2 to 5·2)	0·041	..
Dementia type									
	All-cause dementia	14	8·3% (6·0 to 10·7)	<0·0001	..	5	2·5% (0·7 to 4·3)	0·016	..
	Alzheimer's disease	13	7·5% (5·0 to 10·1)	<0·0001	..	0	NA	NA	..
	Vascular dementia	1	26·2% (13·3 to 39·1)	<0·0001	..	0	NA	NA	..
**Depression, later life**									
Overall		13	7·0% (4·0 to 10·0)	0·0002	99% (p<0·0001)	4	2·4% (−2·0 to 6·9)	0·18	100% (p<0·0001)
Global status									
	Global	3	5·7% (−3·6 to 15·1)	0·12	..	0	NA	NA	..
	HIC	5	6·7% (2·0 to 11·4)	0·016	..	0	NA	NA	..
	LMIC	5	7·7% (−0·9 to 16·4)	0·068	..	4	2·4% (−2·0 to 6·9)	0·18	..
Dementia type									
	All-cause dementia	12	7·3% (4·1 to 10·6)	0·0003	..	4	2·4% (−2·0 to 6·9)	0·18	..
	Alzheimer's disease	1	3·4% (2·0 to 4·8)	0·0008	..	0	NA	NA	..
	Vascular dementia	0	NA	NA	..	0	NA	NA	..
**Social isolation**									
Overall		14	6·0% (3·2 to 8·9)	0·0004	100% (p<0·0001)	8	3·5% (−0·6 to 7·7)	0·083	100% (p<0·0001)
Global status									
	Global	1	6·1% (3·2 to 9·0)	0·0005	..	0	NA	NA	..
	HIC	6	6·5% (1·6 to 11·3)	0·018	..	1	1·6% (0·3 to 2·8)	0·016	..
	LMIC	7	5·5% (0·3 to 10·7)	0·039	..	7	3·8% (−1·0 to 8·7)	0·10	..
Dementia type									
	All-cause dementia	14	6·0% (3·2 to 8·9)	0·0004	..	8	3·5% (−0·6 to 7·7)	0·083	..
	Alzheimer's disease	0	NA	NA	..	0	NA	NA	..
	Vascular dementia	0	NA	NA	..	0	NA	NA	..
**Physical inactivity**									
Overall		49	15·2% (12·8 to 17·7)	<0·0001	98% (p<0·0001)	10	7·3% (3·9 to 11·2)	0·0021	100% (p<0·0001)
Global status									
	Global	7	13·6% (6·0 to 21·2)	0·0046	..	0	NA	NA	..
	HIC	20	15·1% (11·7 to 18·4)	<0·0001	..	1	3·5% (1·6 to 5·3)	0·0087	..
	LMIC	22	15·1% (10·9 to 19·2)	<0·0001	..	9	7·7% (3·4 to 12·0)	0·0031	..
Dementia type									
	All-cause dementia	32	14·0% (10·8 to 17·1)	<0·0001	..	10	7·3% (3·9 to 11·2)	0·0021	..
	Alzheimer's disease	16	18·1% (14·0 to 22·2)	<0·0001	..	0	NA	NA	..
	Vascular dementia	1	15·8% (2·7 to 29·0)	<0·0001	..	0	NA	NA	..
**Diabetes**									
Overall		47	4·9% (4·0 to 5·8)	<0·0001	97% (p<0·0001)	10	2·8% (1·1 to 4·5)	0·0042	98% (p<0·0001)
Global status									
	Global	9	3·8% (2·7 to 4·9)	<0·0001	..	0	NA	NA	..
	HIC	23	5·2% (3·6 to 6·7)	<0·0001	..	2	9·1% (−9·2 to 27·4)	0·10	..
	LMIC	15	5·5% (3·9 to 7·2)	<0·0001	..	8	2·1% (1·1 to 3·0)	0·0011	..
Dementia type									
	All-cause dementia	27	5·6% (4·1 to 7·2)	<0·0001	..	10	2·8% (1·1 to 4·5)	0·0042	..
	Alzheimer's disease	18	4·1% (3·2 to 5·0)	0·0002	..	0	NA	NA	..
	Vascular dementia	2	7·3% (−1·6 to 16·1)	0·061	..	0	NA	NA	..

HIC=high-income country. LMIC=low-income and middle-income country. NA=not applicable (no data available to run the analysis). PAF=population attributable fraction.

No study reported a combined PAF for more than 12 risk factors (range 2–12). Most combined weighted (36 [85·7%] of 42) and unweighted (28 [96·6%] of 29) PAF values were higher than 30% (table 2). There was no obvious pattern in regional variability.

**Table 2:** Weighted and unweighted combined PAF values (combining ≥2 risk factors) by region

	**Location**	**Sample size, n**	**Outcome**	**Number of factors**	**Factors**	PAF_unw_ (95% CI)*	PAF_w_ (95% CI)*
**Global**							
Livingston et al (2020)^3^	Global	NA	All-cause dementia	12	Education, depression, diabetes, hearing loss, hypertension, obesity, traumatic brain injury, alcohol use, physical activity, smoking, social isolation or low social contact, air pollution	NR	40
Livingston et al (2017)^9^	Global	NA	All-cause dementia	9	Education, depression, diabetes, hearing loss, hypertension, obesity, physical activity, smoking, social isolation or low social contact	NR	35
Xu et al (2015)^78^	Global	NA	Alzheimer's disease	9	Education, depression, diabetes†, hypertension, obesity, smoking†, carotid atherosclerosis, homocysteine concentration, frailty	66·0	Not reported
Barnes and Yaffe (2011)^13^	Global	NA	Alzheimer's disease	7	Education, depression, diabetes, hypertension, obesity, physical activity, smoking	50·7	Not reported
Norton et al (2014)^18^	Global	NA	Alzheimer's disease	7	Education, depression, diabetes, hypertension, obesity, physical activity, smoking	49·4 (25·7 to 68·4)	28·2 (14·2 to 41·5)
Hazar et al (2016)^46^	Global	NA	Alzheimer's disease	5	Diabetes, obesity, overweight, physical activity, smoking	35·2	Not reported
**North America**							
Ehrlich et al (2022)^6^	USA	16 690	All-cause dementia	12	Education, depression, diabetes, hearing loss, hypertension, obesity, traumatic brain injury, alcohol use, physical activity, smoking, social isolation or low social contact, vision impairment	NR	62·4
Lee et al (2022)^21^	USA	15 792 to calculate communality weights	All-cause dementia	12	Education, depression, diabetes, hearing loss, hypertension, obesity, traumatic brain injury, alcohol use, physical activity, smoking, social isolation or low social contact, air pollution	NR	41·0 (22·7 to 55·9)
Nianogo et al (2022)^22^	USA	378 615	Alzheimer's disease	8	Education, depression, diabetes, hearing loss, hypertension, obesity, physical activity, smoking	NR	36·9 (36·5 to 37·3)
Barnes and Yaffe (2011)^13^	USA	NA	Alzheimer's disease	7	Education, depression, diabetes, hypertension, obesity, physical activity, smoking	54·1	NR
Norton et al (2014)^18^	USA	NA	Alzheimer's disease	7	Education, depression, diabetes, hypertension, obesity, physical activity, smoking	52·7 (25·9 to 72·8)	30·6 (14·5 to 45·3)
MacDonald et al (2015)^28^	Canada (non-Indigenous)	NR	Alzheimer's disease	6	Education, diabetes, hypertension, obesity, physical activity, smoking	67·1	NR
MacDonald et al (2015)^28^	Canada (Indigenous)	NR	Alzheimer's disease	6	Education, diabetes, hypertension, obesity, physical activity, smoking	76·1	NR
**Latin America and the Caribbean**							
Suemoto et al (2023)^26^	Brazil	9412	All-cause dementia	12	Education, depression, diabetes, hearing loss, hypertension, obesity, traumatic brain injury, alcohol use, physical activity, smoking, social isolation or low social contact, air pollution	77·6 (76·8 to 78·4)	48·2 (47·2 to 49·2)
Borelli et al (2022)^34^	Brazil	9255	All-cause dementia	10	Education, depression, diabetes, hearing loss, hypertension, obesity, alcohol use, physical activity, smoking, social isolation or low social contact	NR	50·5
Mukadam et al (2019)^4^	Latin America (Cuba, Dominican Republic, Mexico, Peru, Puerto Rico, and Venezuela)	12 865	All-cause dementia	9	Education, depression, diabetes, hearing loss, hypertension, obesity, physical activity, smoking, social isolation or low social contact	NR	55·8 (54·9 to 56·7)
Vergara et al (2022)^75^	Chile	3379	All-cause dementia	9	Education, depression, diabetes, hearing loss, hypertension, obesity, physical activity, alcohol use, smoking	NR	45·8 (42·2 to 49·3)
Oliveira et al (2019)^19^	Brazil	NR	All-cause dementia	7	Education, depression, diabetes, hypertension, obesity, physical activity, smoking	55·3 (28·3 to 74·3)	32·3 (15·8 to 46·3)
Ashby-Mitchell et al (2018)^30^	Barbados	NR	All-cause dementia	6	Education, diabetes, hypertension, obesity, physical activity, smoking	58·7 (32·4 to 76·2)	50·9 (28·0 to 67·5)
Ashby-Mitchell et al (2020)^31^	Jamaica	2848 to calculate prevalence estimates; 2943 to calculate shared variance among risk factors	All-cause dementia	5	Education, depression, diabetes, physical activity, smoking	40·1 (25·5 to 52·8)	34·5 (22·0 to 45·7)
Scazufca et al (2010)^66^	Brazil	2003	All-cause dementia	3	Illiteracy, occupation or employment, income (low)	NR	50·4 (29·9 to 64·9)
Scazufca et al (2010)^66^	Brazil	2003	All-cause dementia	2	Illiteracy, occupation or employment	NR	44·8 (21·7 to 59·6)
Scazufca et al (2010)^66^	Brazil	2003	All-cause dementia	2	Occupation or employment, income (low)	NR	48·4 (28·0 to 63·0)
**East Asia and Pacific**							
Ma'u et al (2021)^29^	New Zealand	7745	All-cause dementia	12	Education, depression, diabetes, hearing loss, hypertension, obesity, traumatic brain injury, alcohol use, physical activity, smoking, social isolation or low social contact, air pollution	NR	47·7
Thompson (2022)^27^	Australia (First Nations peoples, including Torres Strait Islander and Aboriginal Peoples in Far North Queensland)	371	All-cause dementia	12	Education, depression, diabetes, hearing loss, hypertension, obesity, traumatic brain injury, alcohol use, physical activity, smoking, social isolation or low social contact, chronic kidney disease	77·9	52·2
Thompson (2022)^27^	Australia (First Nations peoples, including Torres Strait Islander and Aboriginal Peoples in Far North Queensland)	371	All-cause dementia	11	Education, depression, diabetes, hearing loss, hypertension, obesity, traumatic brain injury, alcohol use, physical activity, smoking, social isolation or low social contact	75·4	52·8 (47·8 to 57·9)‡
Mukadam et al (2019)^4^	China	2162	All-cause dementia	9	Education, depression, diabetes, hearing loss, hypertension, obesity, physical activity, smoking, social isolation or low social contact	NR	39·5 (37·5 to 41·6)
Wu et al (2022)^77^	China (Jiangxi province)	2713	All-cause dementia	9	Education, diabetes, hearing loss, hypertension, obesity, physical activity, smoking, social isolation or low social contact, no spouse or unmarried	NR	66·8 (59·6 to 72·3)
Hu et al (2022)^49^	China	17 589	All-cause dementia	8	Education, depression, diabetes, hearing loss, physical activity, social isolation or low social contact, no spouse or unmarried, olfactory decline	NR	53·7 (52·7 to 54·7)
Ashby-Mitchell et al (2017)^5^	Australia	NR	All-cause dementia	7	Education, depression, diabetes, hypertension, obesity, physical activity, smoking	57·0 (33·7 to 73·6)	48·4 (28·1 to 64·2)
Kotaki et al (2019)^14^	Japan	8563	All-cause dementia	7	Education, severe psychological distress, diabetes, hypertension, obesity, physical activity, smoking	50·5	29·6§
Liu et al (2020)^15^	China	Prevalence data source range, 29 345 to 174 621; relative risk data source range, 837 to 8593	All-cause dementia	7	Education, depression, diabetes, hypertension, obesity, physical activity, smoking	55	NR
Woo and Wong (2014)^20^	Hong Kong	NR	Alzheimer's disease	7	Education, depression, diabetes, hypertension, obesity, physical activity, smoking	49·3	NR
Zhang et al (2021)^79^¶	China	3361	All-cause dementia	6	Education, diabetes, hypertension, obesity¶, smoking, social isolation or low social contact	NR	28·8
Hu et al (2022)^7^	China	NR	All-cause dementia	4	Physical activity, mental stimulation activities, stroke or cardiovascular diseases	47·7	NR
**Sub-Saharan Africa**							
Bobrow et al (2021)^33^	South Africa	10 336 (for prevalence of education, hypertension, obesity, diabetes, and smoking only); NR for other risk factors	All-cause dementia	8	Education, depression, diabetes, hypertension, obesity, physical activity, smoking, social isolation or low social contact	NR	45 (27 to 67)
Oliveira et al (2019)^19^	Mozambique	NR	All-cause dementia	7	Education, depression, diabetes, hypertension, obesity, physical activity, smoking	44·0 (23·4 to 61·8)	24·4 (12·9 to 36·1)
**Europe**							
Jørgensen et al (2023)^51^	Denmark	Range across risk factors approximately 2390 to 5 330 000	All-cause dementia	12	Education, depression, diabetes, hearing loss, hypertension, obesity, traumatic brain injury, alcohol use, physical activity, smoking, social isolation or low social contact, air pollution	NR	35·2 (18·6 to 49·8)
Ren et al (2022)^58^	UK (women)	239 508	All-cause dementia	11	Education, diabetes, hypertension, underweight, occupation or employment, alcohol use, cardiovascular disease smoking, respiratory disease, cerebrovascular disease, sleepiness	NR	53·4
Ren et al (2022)^58^	UK (men)	205 187	All-cause dementia	9	Education, diabetes, hypertension, underweight, occupation or employment, alcohol use, respiratory disease, cerebrovascular disease, sleepiness	NR	31·7
Tomata et al (2020)^73^	Sweden	9017	All-cause dementia	9	Education, depression, diabetes, hearing loss, hypertension, obesity, physical activity, smoking, living alone	10·4 (−2·3 to 21·5)‖	NR
Luck et al (2016)^16^	Germany	NR	Alzheimer's disease	7	Education, depression, diabetes, hypertension, obesity, physical activity, smoking	NR	30·5 (13·9 to 45·4)
Mayer et al (2018)^17^	Italy	146 526	Alzheimer's disease	7	Education, depression, diabetes, hypertension, obesity, physical activity, smoking	56·8	45·2
Mayer et al (2018)^17^	Europe	NA	Vascular dementia	7	Education, depression, diabetes, hypertension, obesity, physical activity, smoking	66·8 (42·5 to 83·0)	37·8 (21·2 to 52·5)
Mayer et al (2018)^17^	Italy	146 526	Vascular dementia	7	Education, depression, diabetes, hypertension, obesity, physical activity, smoking	65·9	53·1
Norton et al (2014)^18^	Europe	NA	Alzheimer's disease	7	Education, depression, diabetes, hypertension, obesity, physical activity, smoking	54·0 (27·2 to 73·7)	31·4 (15·3 to 46·0)
Norton et al (2014)^18^	UK	NA	Alzheimer's disease	7	Education, depression, diabetes, hypertension, obesity, physical activity, smoking	52·0 (25·6 to 71·9)	30·0 (14·3 to 44·4)
Oliveira et al (2019)^19^	Portugal	NR	All-cause dementia	7	Education, depression, diabetes, hypertension, obesity, physical activity, smoking	65·8 (36·2 to 83·4)	40·1 (20·7 to 55·4)
Rolandi et al (2020)^60^	Italy	1100	All-cause dementia	6	Education, diabetes, heart disease, stroke, physical activity, delirium	40·0	NR
Zhang et al (2023)^80^**	UK	344 324	All-cause dementia	6	Lifestyle factors, medical history, local environmental factors, psychosocial factors, physical measures, socioeconomic status factors	NR	47·0
de Bruijn et al (2015)^37^	Netherlands	2953	All-cause dementia	5	Education, diabetes, hypertension, coronary heart disease, stroke	NR	33·0 (7·0 to 77·0)
de Bruijn et al (2015)^37^	Netherlands	2953	All-cause dementia	3	Education, diabetes, hypertension	NR	30·0 (6·0 to 76·0)
**South Asia**							
Mukadam et al (2019)^4^	India	2004	All-cause dementia	9	Education, depression, diabetes, hearing loss, hypertension, obesity, physical activity, smoking, social isolation or low social contact	NR	41·2 (39·1 to 43·4)
**Middle East and north Africa**							
Hazar et al (2016)^46^	Iran	NA	Alzheimer's disease	5	Diabetes, obesity, overweight, physical activity, smoking	30·8	NR

PAF=population attributable fraction. PAF_unw_=population attributable fraction (unweighted). PAF_w_=population attributable fraction (weighted for communality or overlap in risk between factors). NA=not applicable (NA is used for sample size when PAF estimates were created based on reviews or meta-analyses). NR=not reported (NR is used for sample size when PAF estimates were based on original data but sample size was not provided).

* 95% CIs reported when available.

† Diabetes and current smoking were risk factors in Asian individuals.

‡ Age-standardised prevalence-weighted PAF was 52·1% (47·1 to 57·2).

§ Results of full adjusted model in which hazard ratios were adjusted for age, sex, history of diabetes, hypertension, BMI, time spent walking per day, Kessler 6-Item Psychological Distress Scale score, smoking status, and educational level.

¶ Overweight and obesity combined.

‖ Controlled for age, sex, and cognitive function in the modelling.

** Results reported for a conservative model shifting unfavourable profiles to intermediate and favourable profiles based on risk factor score tertiles.

**Figure 2:**
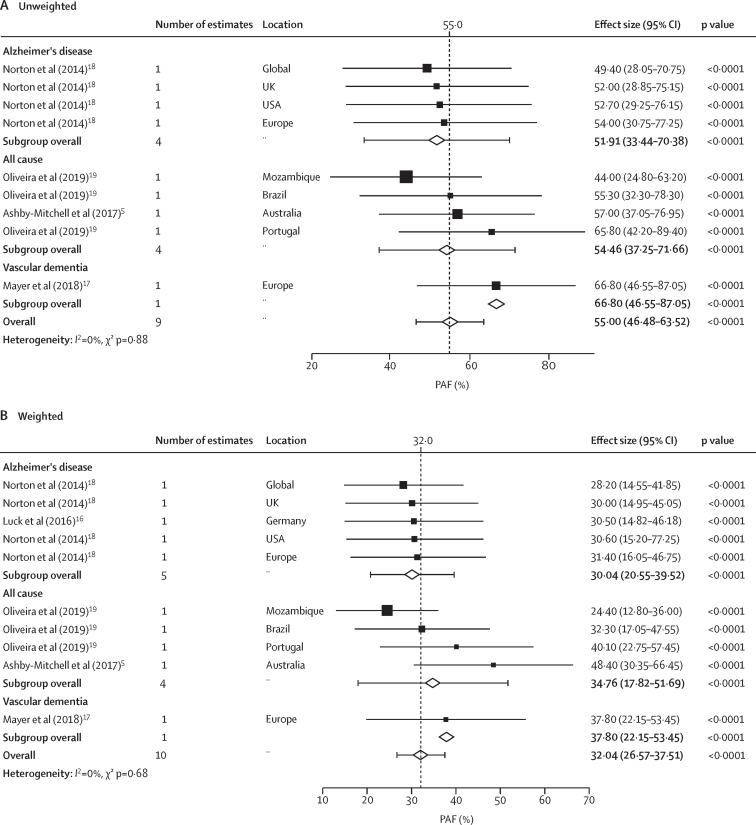
Random-effects meta-analysis of unweighted (A) and weighted (B) PAF estimates for seven risk factors combined, stratified by dementia type The combined risk factors were low education, midlife hypertension, midlife obesity, smoking, physical inactivity, depression, and diabetes (ie, the seven-factor model proposed by Barnes and Yaffe^13^). PAF=population attributable fraction.

Regarding the effect of combined risk factors, the only combination of risk factors to have been assessed in five or more studies was the seven-factor model proposed by Barnes and Yaffe,^13^ which combines low education, midlife hypertension, midlife obesity, smoking, physical inactivity, depression, and diabetes (n=9 studies^5^, ^13^, ^14^, ^15^, ^16^, ^17^, ^18^, ^19^, ^20^). The unweighted PAF values from these studies were generally higher than 50% (appendix p 99). The weighted PAF values were lower and showed high variability across countries (ranging from 24·4% [95% CI 12·9–36·1] for all-cause dementia in Mozambique,^19^ to 53·1% [95% CI not reported] for vascular dementia in Italy;^17^ appendix p 99). When the results were pooled in a meta-analysis (collapsed across all dementia outcomes and study locations), the combined unweighted PAF was 55·0% (46·5–63·5; p<0·0001) and the combined weighted PAF was 32·0% (26·6–37·5; p<0·0001). When stratified by dementia type, PAF estimates were similar for all-cause dementia versus Alzheimer's disease in both unweighted and weighted models (figure 2A, B). Funnel plots and Egger's regression test results to assess publication bias in the analyses (unweighted and weighted) of the seven-factor model proposed by Barnes and Yaffe are shown in the appendix (pp 100–101).

Ten studies investigated ethnic variability in PAF values including five^16^, ^21^, ^22^, ^23^, ^24^ in the USA, two in Brazil,^25^, ^26^ and one study each in Australia,^27^ Canada,^28^ and New Zealand.^29^ Within studies, compared with White and Asian participants, the combined PAF values (combining multiple risk factors; for Alzheimer's disease and all-cause dementia) were generally higher in individuals who identified as Black, Hispanic, or Indigenous (eg, Native American and Alaska Native, Māori, or Pacific Peoples; appendix pp 102–103). The largest reported unweighted PAF difference was between White (non-Hispanic) and Hispanic women in the USA when combining socioeconomic resources, lifestyle characteristics, and medical conditions (PAF difference 49·3 percentage points, lower PAF in White non-Hispanic women).^24^ For the weighted results, the largest difference was between Asian and Black ethnicities in the USA, when combining education, hearing loss, midlife hypertension, midlife obesity, current smoking, depression, physical inactivity, and diabetes (PAF difference 24·0 percentage points, lower PAF in Asian group).^22^ Regarding PAF values for the individual risk factors in the *Lancet* Commission,^3^ generally we found no consistent pattern in which factors were associated with the highest or lowest risk of dementia in a given ethnic group; however, the PAF values for low educational attainment were generally higher in people of non-White and non-Asian ethnicities across most studies. Furthermore, relative to other ethnic groups, individuals who identified as Asian typically had lower PAF values associated with obesity in individual studies (appendix pp 104–13).

## Discussion

To our knowledge, this is the first study to collate PAF values associated with modifiable risk factors for dementia. The results highlight that a considerable proportion of dementia cases are potentially preventable. Among the factors investigated, there is robust evidence to suggest that low levels of education, physical inactivity, hearing loss, hypertension, and obesity are key risks for dementia globally. The findings suggest that combined prevention programmes, that simultaneously address these modifiable risk factors, could yield greater benefits for dementia prevention than interventions targeting single risk factors, particularly in LMIC settings.

The present study has several strengths. First, the search strategy was comprehensive without restrictions on dementia type or the risk factors investigated. This approach ensured all relevant literature was captured. Second, we set no restriction on language to minimise English bias. Third, when a sufficient number of studies (five or more) was available on a given risk factor or combination of risk factors, a meta-analysis was undertaken to provide a pooled PAF estimate.

This study also had limitations. First, PAF estimates vary depending on the population and the risk factors considered. Very few studies assessed the same set of risk factors, making cross-study comparison difficult and meta-analysis of most combinations of factors not possible. Data were also limited for some individual risk factors, such as air pollution. Ideally, PAFs should be computed on the basis of the intended intervention and specific populations with unique risk profiles, despite the challenges in cross-study comparisons. Second, heterogeneity was high in the meta-analysis results. Variability in PAF values could be due to several reasons, including differences in risk factor assessment (eg, midlife *vs* later life; and self-reported *vs* objective measures), disease prevalence across populations, study design, sample characteristics, and dementia outcome. More consideration is needed about how risks are measured, sex differences in risk, and the influence of the timing of risk factor occurrence and assessment throughout various life stages, and across diverse world regions with varying life expectancies, on PAF estimates. These aspects have implications for the design of preventive interventions tailored to a given population. Third, the PAF calculation assumes causality; thus, if no causal relationship exists, the PAF calculation might yield inaccurate or misleading results. Last, most risk factors have been identified from research in HICs and the literature predominately focuses on one or more of the 12 factors reported by the *Lancet* Commission.^3^ Other key risks, such as environmental factors (eg, pollution), diet (individual foods such as fish intake, and dietary patterns), location (urban *vs* rural), and factors more common in LMICs than HICs (eg, poverty and food security) are typically missing from PAF analyses. Additional analyses incorporating different dementia subtypes and novel risk factors, and across multiple world regions, including LMICs, are needed to enhance understanding of modifiable risk factors.

Regarding individual risk factors, the results support existing evidence of a strong association between risk of dementia and educational attainment, health (hearing loss, hypertension, and obesity), and lifestyle (physical inactivity and smoking). More years of education has been linked to high cognitive reserve and improved health outcomes leading to reduced dementia risk.^81^ When comparing across regions, the higher unweighted pooled PAF estimate for low education in LMICs (*vs* HICs) is likely to reflect the gaps in education systems (eg, poorer resource availability, infrastructure, teaching quality, and materials and technology), less rigid compulsory schooling, and often poorer quality public education and low literacy levels in these settings.^82^, ^83^ Although the mechanisms linking hearing loss to dementia are not well understood, several potential pathways have been proposed. These pathways include shared pathology (ie, accumulation of amyloid β in auditory regions), and hearing loss resulting in structural and functional brain changes and imposing increased cognitive load, leading to reduced cognitive resources and decline, reduced social interaction, and increased dementia risk.^84^ Poor cardiometabolic health and unhealthy lifestyle factors have been consistently linked to reduced blood flow, atherosclerosis, inflammation, oxidative stress, impaired brain glucose metabolism, disruption to energy production and neural function, and neurotransmitter and neurotrophic factor imbalance, all of which are associated with dementia.^85^, ^86^ Therefore, strategies to increase education, improve management of auditory and cardiometabolic health, and increase physical activity levels have the potential to mitigate a large proportion of dementia cases.

When risk factors were combined in individual studies, regardless of the combination, most of the PAF values (unweighted and weighted) for dementia were higher than 30%. Based on a current estimate of approximately 57 million people with dementia worldwide in 2019,^1^ being able to prevent a minimum of 30% of cases would translate as over 17 million fewer people with dementia globally. The combined PAF estimates showed variability across countries with a range of risk factors included; with no consistent patterns. To inform the development of context-specific risk reduction and prevention strategies it will be important to ascertain region-specific estimates for the most prevalent risk factors and their combinations.

PAF estimates also showed intranational ethnic variability. Compared with White and Asian participants, people identifying as Black or Hispanic or from Indigenous groups generally had higher combined PAF values. When looking at individual risk factors, although the pattern of results was generally similar across ethnicities, we observed some notable exceptions (ie, high PAF values for low education in Black, Hispanic, and Indigenous groups, and low PAF values for obesity in Asian people). Unequal educational outcomes could be due to several factors, including clustering in communities of intergenerational poverty and disadvantage across the life course in addition to early education experience and socioeconomic status. Compared with HICs, some Asian countries have considerably lower rates of overweight and obesity, possibly driven by cultural, lifestyle, and dietary factors;^87^ and these might continue to exert influence regardless of where individuals live. However, this hypothesis requires testing. As health status (eg, rising obesity and cardiovascular disease rates) and lifestyle factors (changes in dietary habits, increased alcohol consumption and smoking, and trends towards sedentary lifestyles) change in Asian societies,^88^, ^89^, ^90^ it will be important to establish whether these changes impact the patterns in PAF values. Overall, the results highlight that interventions will not only need to be tailored to different countries but also to the unique risk profiles and needs of different groups within countries. All countries should also address inequality and poverty.

Interventions targeting key risk factors including low educational attainment, hearing loss, physical inactivity, obesity, and hypertension have the potential to lead to a substantiable reduction in future dementia cases worldwide. Gaps were also identified. Indeed, there are key factors, such as diet and air pollution, for which data are not yet sufficient to estimate PAF, but which could be included among key targets for intervention. When risk factors are combined, the potential for prevention increases. The next steps will be to establish the most effective, cost-effective, and feasible strategies for reducing these risks, that can be sustained within local contexts and achieve the best impacts, at the population level, in the short, medium, and long term. Studies into future strategies are necessary to inform public policy, to ultimately limit the burden of disease associated with dementia.

## Data sharing

All data used in this paper, including the extracted PAF data from individual studies, may be requested for use in further research from the corresponding author (blossom.stephan@curtin.edu.au). The data are in Excel format and can be requested following Article publication.
