# Supplementary material for: Population attributable fractions of modifiable risk factors for dementia: a systematic review and meta-analysis
Source: Lancet Healthy Longev. 2024 Jun;5(6):e406–21. doi: 10.1016/S2666-7568(24)00061-8 (PMC11139659; doi:10.1016/S2666-7568(24)00061-8)
Supplement: Supplementary appendix [file mmc1.pdf]

THE LANCET
Healthy Longevity

Supplementary appendix

This appendix formed part of the original submission and has been peer reviewed. We post it as supplied by the authors.

Supplement to: Stephan BCM, Cochrane L, Kafadar AH, et al. Population attributable fractions of modifiable risk factors for dementia: a systematic review and meta-analysis. *Lancet Healthy Longev* 2024; **5:** e406–21.

Appendix Materials

Article Title

Population Attributable Fractions of modifiable risk factors for dementia: A systematic review and meta-analysis

Author List

Blossom CM Stephan, Louie Cochrane, Aysegul Humeyra Kafadar, Jacob Brain, Elissa Burton, Bronwyn Myers, Carol Brayne, Aliya Naheed, Kaarin J Anstey, Ammar W Ashor, Mario Siervo

Appendix 1 PRISMA checklist of items to include when reporting a systematic review or meta-analysis

Section/topic	*#*	*Checklist item*	*Reported on page #*
**TITLE**			
Title	1	Identify the report as a systematic review, meta-analysis, or both.	Page 1
**ABSTRACT**			
Structured summary	2	Provide a structured summary including, as applicable: background; objectives; data sources; study eligibility criteria, participants, and interventions; study appraisal and synthesis methods; results; limitations; conclusions and implications of key findings; systematic review registration number.	Page 1
**INTRODUCTION**			
Rationale	3	Describe the rationale for the review in the context of what is already known.	Pages 1, 2
Objectives	4	Provide an explicit statement of questions being addressed with reference to participants, interventions, comparisons, outcomes, and study design (PICOS).	Page 2
**METHODS**			
Protocol and registration	5	Indicate if a review protocol exists, if and where it can be accessed (e.g., Web address), and, if available, provide registration information including registration number.	Page 4
Eligibility criteria	6	Specify study characteristics (e.g., PICOS, length of follow-up) and report characteristics (e.g., years considered, language, publication status) used as criteria for eligibility, giving rationale.	Pages 2, 3
Information sources	7	Describe all information sources (e.g., databases with dates of coverage, contact with study authors to identify additional studies) in the search and date last searched.	Page 2
Search	8	Present full electronic search strategy for at least one database, including any limits used, such that it could be repeated.	Appendix page 5
Study selection	9	State the process for selecting studies (i.e., screening, eligibility, included in systematic review, and, if applicable, included in the meta-analysis).	Pages 2, 3
Data collection process	10	Describe method of data extraction from reports (e.g., piloted forms, independently, in duplicate) and any processes for obtaining and confirming data from investigators.	Page 3

Section/topic	#	Checklist item	Reported on page #
Data items	11	List and define all variables for which data were sought (e.g., PICOS, funding sources) and any assumptions and simplifications made.	Page 3
Risk of bias in individual studies	12	Describe methods used for assessing risk of bias of individual studies (including specification of whether this was done at the study or outcome level), and how this information is to be used in any data synthesis.	Page 3
Summary measures	13	State the principal summary measures (e.g., risk ratio, difference in means).	Page 3
Synthesis of results	14	Describe the methods of handling data and combining results of studies, if done, including measures of consistency (e.g., I^2) for each meta-analysis.	Pages 3, 4
Risk of bias across studies	15	Specify any assessment of risk of bias that may affect the cumulative evidence (e.g., publication bias, selective reporting within studies).	Pages 3, 4
Additional analyses	16	Describe methods of additional analyses (e.g., sensitivity or subgroup analyses, meta-regression), if done, indicating which were pre-specified.	Pages 3, 4
RESULTS			
Study selection	17	Give numbers of studies screened, assessed for eligibility, and included in the review, with reasons for exclusions at each stage, ideally with a flow diagram.	Pages 4, 5
Study characteristics	18	For each study, present characteristics for which data were extracted (e.g., study size, PICOS, follow-up period) and provide the citations.	Appendix pages 8-18
Risk of bias within studies	19	Present data on risk of bias of each study and, if available, any outcome-level assessment (see Item 12).	Appendix pages 22-26
Results of individual studies	20	For all outcomes considered (benefits or harms), present, for each study: (a) simple summary data for each intervention group and (b) effect estimates and confidence intervals, ideally with a forest plot.	Pages 9-11<br>Appendix pages 8-18
Synthesis of results	21	Present results of each meta-analysis done, including confidence intervals and measures of consistency.	Pages 4-8 & 12<br>Appendix pages 34-59
Risk of bias across studies	22	Present results of any assessment of risk of bias across studies (see Item 15).	Pages 4, 5<br>Appendix pages 74-100

Section/topic	*#*	*Checklist item*	*Reported on page #*
Additional analysis	23	Give results of additional analyses, if done (e.g., sensitivity or subgroup analyses, meta-regression [see Item 16]).	Appendix page 60
DISCUSSION			
Summary of evidence	24	Summarize the main findings including the strength of evidence for each main outcome; consider their relevance to key groups (e.g., health care providers, users, and policy makers).	Pages 8 & 11
Limitations	25	Discuss limitations at study and outcome level (e.g., risk of bias), and at review level (e.g., incomplete retrieval of identified research, reporting bias).	Pages 11-13
Conclusions	26	Provide a general interpretation of the results in the context of other evidence, and implications for future research.	Pages 13, 14
FUNDING			
Funding	27	Describe sources of funding for the systematic review and other support (e.g., supply of data); role of funders for the systematic review.	Page 1
This checklist has been downloaded from PRISMA - For meta-analyses and systematic reviews — http://www.plosmedicine.org/article/info%3Adoi%2F10.1371%2Fjournal.pmed.1000097			

Appendix 2 Ovid search strategy

1) (dementia OR demented OR Alzheimer OR AD OR vascular OR VaD).ti,ab,kw.
2) population attributable fraction [Including Related Terms]
3) population attributable risk [Including Related Terms]
4) PAF [Including Related Terms]
5) etiologic fraction [Including Related Terms]
6) 2 OR 3 OR 4 OR 5
7) 1 AND 6

Appendix 3 Formulae for calculating a PAF value and 95% confidence interval (95%CI)

The Population Attributable Fraction (PAF) can be calculated using Levin's formula as follows:

$$PAF = \frac{P(RR-1)}{1+\ P(RR-1)}$$

Where: P=Prevalence of the exposure in the study population, and RR=the relative risk or risk ratio associated with the exposure.

You can multiple this value by 100 to get the PAF percent.

If you have the 95% confidence interval for the RR and you know the prevalence of the exposure in the population, you can use this information to estimate the 95% confidence interval (95%CI) for the PAF using the following:

$$1.\, PAF_{lower} = \frac{P(RR_{lower}-1)}{1+\ P(RR_{lower}-1)}$$

$$2.\, PAF_{upper} = \frac{P(RR_{upper}-1)}{1+\ P(RR_{upper}-1)}$$

Where:

PAF_{lower} and PAF_{upper} are the estimated lower and upper bounds of the 95%CI, respectively.

RR_{upper} and RR_{upper} are the lower and upper bounds of the 95%CI for the relative risk (RR), respectively.

Appendix 4 Strategy for classifying articles into different quality levels by scale

Scale	Categorisation
Newcastle-Ottawa Scale (NOS)	Total number of stars categorised into three groups:<br>1. High quality (Scores≥7)<br>2. Moderate quality (Scores range: 4-6)<br>3. Low quality (Scores range: 1-3)
A Measurement Tool to Assess Systematic Reviews, Version 2 (AMSTAR-2)	Scores classified into four groups:<br>1. High (No or one non-critical weakness)<br>2. Moderate (Greater than one non-criterial weakness)<br>3. Low (One critical flaw with/without a non-critical weakness)<br>4. Critically low (Greater than one critical flaw with/without non-critical weaknesses)
Scale for the Assessment of Narrative Review Articles (SANRA)	Decisions on high, moderate, or low quality are generally made from best judgement rather than a designated score but a score of ≤4 has been used to classify a narrative review into "weak quality of method"

Appendix 5 Key characteristics of included studies (ordered alphabetically)

First author (year)	Study Design and Data Source	Population Representative	Prevalent or Incident Dementia Study	Geographical Location	Sample Size§	Outcome	Number of Modifiable Risk Factors	Modifiable Risk Factors Examined (NB if the study included non-modifiable factors these have not been included)	Combined PAF_{unw} (95%CI) (or individual PAF_{unw}, for those studies that investigated only one factor)	Combined PAF_{w} (95%CI)	Quality
Ashby-Mitchell (2017)[1]	Cohort study (2011–2013 Australian Health Survey; Age>5)	Yes	Prevalent	Australia	Not clear	All-cause	7	Education (low), depression (lifetime), diabetes (adult), hypertension (ML), obesity (ML), physical inactivity (adult) & smoking (adult)	57.0% (33.7-73.6)	48.4% (28.1-64.2)	High
Ashby-Mitchell (2018)[2]	Cohort study (Cross-sectional survey of the Barbados population, 2014 World Alzheimer's Report & SABE; Age≥25)	Yes	Prevalent	Barbados	Not reported	All-cause	6	Education (low), diabetes (adult), hypertension (ML), obesity (ML), physical inactivity (adult) & smoking (adult)	58.7% (32.4 –76.2)	50.9% (28.0 –67.5)	Mod
Ashby-Mitchell (2020)[3]	Cohort study (Jamaica Health and Lifestyle Survey 2008 [JHLS], World Alzheimer's Report 2014 & Health and Social Status of Older Persons in Jamaica Study [HSSOPJS]; Age≥15)	Yes	Prevalent	Jamaica	JHLS: 2,848<br>HSSOPJS: 2,943	All-cause	5	Education (low), depression (lifetime), diabetes (adult), physical inactivity (adult) & smoking (adult)	40.1% (25.5-52.8)	34.5% (22.0-45.7)	High
Barnes (2011)[4]	Review	Not appliable	Incident	Global & USA	Not applicable	AD	7	Education (low), depression (lifetime), diabetes (adult), hypertension (ML), obesity (ML), physical inactivity (lifetime) & smoking (≥15 years)	Global: 50.7%<br>USA: 54.1%	Not reported	High
Beydoun (2014)[5]	SR	Not applicable	Prevalent & incident	Global	Not applicable	AD	5	Education (low), homocysteine level, fish consumption, physical inactivity & smoking (ML)	Not reported	Not reported	Low
Bobrow (2021)[6]	Cohort study (2016 South African Demographic Health and Surveillance study & WHO SA-SAGE 2007-2008; Adults)	Yes	Prevalent	South Africa	10,336 (for prevalence of education, hypertension, obesity, diabetes and smoking only). Sample size not reported for the other risk factors	All-cause	8	Education (low), depression, diabetes (20-79 years), hypertension (ML), obesity (ML), physical inactivity, smoking & social isolation	Not reported	45% (27-67)	High
Borelli (2022)[7] & Borelli (2023)[8]	Cohort study (Brazilian Longitudinal Study of Aging; Age≥50)	Yes	Prevalent	Brazil	Borelli (2022) n=9,255<br><br>Borelli (2023) n=9,070	All-cause	10	Education (low), depression, diabetes, hearing loss, hypertension, obesity, alcohol (high), physical inactivity, smoking (LL) & social isolation	Not reported	Total: 50.5%<br>Asian: 41.2%<br>Black: 50.0%<br>Brown: 50.1%<br>Indigenous: 38.9%<br>White: 49.2%	Moderate
Bothongo (2022)[9]	Cohort study (EMIS electronic health care records for the Secure Health Analysis and Research in East London project; Age: 27-103)	Yes	Prevalent	England (London)	19,891	All-cause	8	Area level deprivation, depression, diabetes, hearing loss, head injury, hypertension, underweight & smoking (ever)	Not reported	Not reported* (included Ethnicity in the calculation)	High
Bubu (2017)[10]	SR	Not applicable	Prevalent & incident	Global	Not applicable	AD	1	Sleep problems	15%	Not applicable	High
de Bruijn (2015)[11]	Cohort study (Rotterdam Study; Age≥55)	No	Incident	Netherlands	2,953	All-cause	5	**2000 cohort**<br>Set 1: Education, diabetes & hypertension<br>Set 2: Set 1 + coronary heart disease & stroke	Not reported	Set 1: 30.0% (6.0-76.0)<br>Set 2: 33.0% (7.0-77.0)	High
Desai (2020)[12]	SR (prevalence estimate from UK Census Data)	Yes	Incident	UK	Not reported	All-cause	1	Social isolation	8.9%	Not applicable	Moderate
Dodge (2011)[13]	Cohort study (Monongahela Valley	No	Incident	USA	822	All-cause, AD	1	Lifetime cerebrovascular disease (including stroke or TIA)	**All-cause dementia**<br>Total Sample: 8.4%	Not applicable	High

First author (year)	Study Design and Data Source	Population Representative	Prevalent or Incident Dementia Study	Geographical Location	Sample Size§	Outcome	Number of Modifiable Risk Factors	Modifiable Risk Factors Examined (NB if the study included non-modifiable factors these have not been included)	Combined PAF_{unw} (95%CI) (or individual PAF_{unw}, for those studies that investigated only one factor)	Combined PAF_w (95%CI)	Quality
	Independent Elders Survey; Age≥65)								APOE e4 non-carriers: 10.8%<br>**Probable/possible AD**<br>APOE e4 non-carriers: 9.1%		
Dragioti (2022)[14]	SR	Not applicable	Incident	Global, Europe, USA, country income level (high, middle, & low)	Not applicable	All-cause, AD, VaD	4	Benzodiazepines use, depression (lifetime and LL analysed separately), diabetes & physical inactivity	Not reported	Not reported	Low
Ehrlich (2022)[15]	Cohort study (Health and Retirement Study; Age≥50)	Yes	Prevalent	USA	16,690	All-cause	12	Education (low), depression, diabetes, hearing loss, hypertension, obesity, TBI, alcohol (high), physical inactivity, smoking, social isolation & vision impairment	Not reported	Total: 62.4%<br>Black, non-Hispanic: 68.6%<br>Hispanic, any race: 69.3%<br>White, non-Hispanic: 60.6%	Moderate
Esteban-Cornejo (2022)[16]	Cohort study (UK Biobank; Age: 37-73)	No	Incident	UK	466,830	All-cause	1	Handgrip strength	Lowest quartile: 30.1% (25.3-34.6)<br>Muscle weakness (Fried criteria): 10.0% (8.1-11.8)	Not applicable	High
Feter (2022)[17]	Cohort study (Repository of the Global Health Observatory 2015 & United Nations Development Program website 2019; Age>18)	No	Prevalent	LMIC sites (N=160 countries#)	Not reported	All-cause	1	Physical activity	All LMIC sites: 12.3% (10.7-13.9)<br>LMIC Africa: 12.8% (10.6–14.9)<br>LMIC America: 6.3%<br>LMIC Asia: 11.5% (8.8–14.2)<br>LMIC Oceania: 9.3% (4.0–20.1)	Not applicable	Moderate
Gardner (2023)[18]	SR and cohort study (Health and Retirement Study; Age≥50)	Yes	Prevalent	USA	1,489	All-cause	1	TBI	Total population: 14%<br>Males: 32%<br>Females: 9%<br>Veterans: 28%<br>Male veterans: 29%<br>Female veterans: 3.8%	Not applicable	Moderate
GBD 2019 Dementia Collaborators (2021)[19]	Review	Not applicable	Prevalent & incident	Global (and N=21 regional estimates+)	Not applicable	All-cause	3	Parkinson's disease, stroke & TBI	Not reported (combined the estimate with Down's Syndrome)	Not reported	High
Hagstrom (2014)[20]	Cohort study (Uppsala Longitudinal Study Of Adult Men; Age 71)	No	Incident	Sweden	998	VaD	6	Education (low), diabetes, hypercholesterolemia, hypertension, obesity & plasma parathyroid hormone	Not reported	Not reported	High
Hazar (2016)[21]	SR	Not applicable	Case-control, cohort & prospective cohort	Global & Iran	Not applicable	AD	5	Diabetes, obesity (ML), overweight (ML), physical inactivity & smoking	Global: 35.2%<br>Iran: 30.8%	Not reported	Low
Hegelund (2021)[22]	Cohort study (National Register Based Study 200-18; Age ≥65)	Yes	Incident	Denmark	1,757,168	All-cause	4	Education (low), polypharmacy, stroke & household wealth	Not reported	Not reported	High
Hodis (2020)[23]	Cohort study (Atherosclerosis Risk in Communities Study; Age 45-64)	No	Incident	USA	13,971	All-cause	1	Hypertension (Midlife; 45-64 years)	**JNC7 Guidelines**<br>Prehypertension: 6.3% (2.9-9.1)<br>Stage 1: 0.1% (-1.6-1.5)<br>Stage 2: 9.4% (5.1-13.2)<br>Binary: 4.4% (-0.9-8.5)<br>**ACC/AHA Guidelines**<br>Elevated: 3.1% (1.3-4.7)<br>Stage 1: 3.1% (0.9-4.9)<br>Stage 2: 9.1% (4.0-13.7)<br>Binary: 8.1% (1.7-13.5)	Not applicable	High

First author (year)	Study Design and Data Source	Population Representative	Prevalent or Incident Dementia Study	Geographical Location	Sample Size§	Outcome	Number of Modifiable Risk Factors	Modifiable Risk Factors Examined (NB if the study included non-modifiable factors these have not been included)	Combined PAF_{unw} (95%CI) (or individual PAF_{unw}, for those studies that investigated only one factor)	Combined PAF_{w} (95%CI)	Quality
Fei-fei Hu (2022)[24]	Cohort study (China Multicentre Dementia Survey: CMDS; Age≥65)	No	Prevalent	China	17,589	All-cause	8	Education (low), depression (LL), diabetes (LL), hearing impairment (ML), olfactory decline (LL), physical inactivity (LL), social isolation (LL) & unmarried (LL)	Not reported	Total: 53.7% (52.7–54.7)<br>Urban: 50.6% (49.4–51.9)<br>Rural: 56.5% (55.6–57.5)	High
Mingyue Hu (2022)[25]	Cohort study (Chinese Longitudinal Healthy Longevity Survey; Age≥65)	Yes	Incident	China	Not clear	All-cause (MMSE<18)	4	Physical activity (duration), playing cards/mah-jongg, watching TV/listening to the radio & stroke/cardiovascular diseases	47.7%	Not reported	High
Johannesdottir Schmidt (2022)[26]	Cohort study (Nationwide Danish registries; Age≥40)	Yes	Incident	Denmark	1,483,195	All-cause	1	Herpes Zoster infection involving the CNS	0.014%	Not applicable	High
Jørgensen (2023)[27]	Cohort study (Danish National Health Survey 2021, Danish Health Data Authority & Danish National Patient Register; Age≥16)	Yes	Prevalent	Denmark	Range across risk factors: Approximately 2,390 to 5,330,000	All-cause	12	Education (low; at age 45-64 years), depression (LL), diabetes (LL), hearing loss (ML), hypertension (ML), obesity (ML), TBI (anytime), alcohol (excessive; ML), physical inactivity (LL), smoking (LL), social isolation (LL) & air pollution (anytime)	Not reported	35.2% (18.6-49.8)	High
Katzmarzyk (2022)[28]	Review	Not applicable	Incident	Global (168 countries, 3 income levels & 10 regions#)	Not applicable	All-cause	1	Physical inactivity	Global: 8.1% (2.6-14.9)<br>Low-income: 4.9% (1.5-9.2)<br>Middle-income: 7.7% (2.4-14.3)<br>High-income: 10.5% (3.3-19.5)<br>Central Asia, Middle East & North Africa: 9.5% (2.8-17.5)<br>Central & Eastern Europe: 7.0% (2.2- 12.9)<br>East & Southeast Asia: 5.3% (1.6-9.7)<br>High-Income Asia Pacific: 10.3% (3.2-18.8)<br>High-Income Western: 10.5% (3.3-19.1)<br>Latin America & Caribbean: 11.1% (3.6-20.4)<br>Oceania: 5.0% (1.5-9.3)<br>South: 9.6% (2.6-19.1)<br>Sub-Saharan Africa: 6.4% (2.0-11.8)	Not applicable	High
Kloppenborg (2008)[29]	SR	Not applicable	Incident Studies	Global	Not applicable	All-cause	4	Diabetes, dyslipidaemia, hypertension & obesity	Not reported	Not reported	Critically low
Kotaki (2019)[30]	Cohort study (Ohsaki Cohort 2006 Study and Long-term Care Insurance database; Age≥65)	No	Incident	Japan	8,563	All-cause	7	Education, severe psychological distress (including depression), diabetes, hypertension, obesity, physical inactivity & smoking	Unadjusted: 50.5%	Age & sex adjusted: 35.0%<br>Adjusted for age, sex, education, diabetes, hypertension, BMI, time spent walking, psychological distress & smoking: 29.6%	High
Launer (2010)[31]	Cohort study (Honolulu Asia Aging Study; Age: 45-68)	No	Incident	USA	7,878	All-cause	1	Hypertension (ML; controlling for age, education, ML smoking, obesity, diabetes, CHD (angina, myocardial infarction, coronary insufficiency) & stroke/coronary heart disease)	Untreated: 27.0% (8.9-42.1)<br>Treated: 17.7% (4.6-29.1)	Not applicable	High
Lee (2022)[32]	Cohort study (American Community Survey, National Health and Nutrition Examination Survey, National Health	Yes	Prevalent	USA	Not clear (n=15,792 used to determine combined	All-cause	12	Education (low), depression (LL), diabetes (LL), hearing loss (ML), hypertension (ML), obesity (ML), TBI (ML), alcohol (high; ML), physical inactivity (LL), smoking (LL), social isolation (LL) & air pollution (LL)	Not reported	Total: 41.0% (22.7-55.9)<br>White: 39.4% (22.7-55.9)<br>Black: 45.6% (25.7-60.5)<br>Hispanic: 46.7% (27.3-61.5)<br>Asian: 35.8% (19.5-49.9)	Moderate

First author (year)	Study Design and Data Source	Population Representative	Prevalent or Incident Dementia Study	Geographical Location	Sample Size§	Outcome	Number of Modifiable Risk Factors	Modifiable Risk Factors Examined (NB if the study included non-modifiable factors these have not been included)	Combined PAF_{unw} (95%CI) (or individual PAF_{unw}, for those studies that investigated only one factor)	Combined PAF_{w} (95%CI)	Quality
	Interview Survey, National Social Life, Health, and Aging Project & the Atherosclerosis Risk in Communities study; Age≥25)				prevalence of dementia associated with n=12 factors)						
Liu (2020)[33]	Cohort study (China Chronic Disease and Risk Factor Surveillance Survey 2013, China National Survey of Chronic Kidney Disease 2010 & China Family Panel Studies Survey; Age≥18)	Yes	Prevalent	China	Prevalence data source range: 29,345-174,621<br><br>RR data source range: 837-8,593	All-cause	7	Education (low), depression (adults), diabetes (adult), hypertension (ML), obesity (ML), physical inactivity (adult) & smoking (current)	55%	Not reported	High
Livingston (2017)[34]	Review	Not applicable	Prevalent	Global	Not applicable	All-cause	9	Education, depression (LL), diabetes (LL), hearing loss (ML), hypertension (ML), obesity (ML), physical inactivity (LL), smoking (LL) & social isolation (LL)	Not reported	35%	High
Livingston (2020)[35]	Review	Not applicable	Prevalent	Global	Not applicable	All-cause	12	Education (low), depression (LL), diabetes (LL), hearing loss (ML), hypertension (ML), obesity (ML), TBI (ML), alcohol (high, ML), physical inactivity (LL), smoking (LL), social isolation (LL) & air pollution (LL)	Not reported	40%	High
Loef (2013)[36]	SR	Not applicable	Incident (prospective studies)	China, USA	Not applicable	All-cause	1	Weight (ML obesity assessed in the USA and overweight/obesity assessed in China) – results are adjusted for mortality	**USA - Obesity**<br>Men: 13.0% (9.0-17.0)<br>Women: 14.0% (10.0-19.0)<br>**China – Overweight & obesity**<br>Men: 4.0% (3.0-5.0)<br>Women: 6.0% (4.0-7.0)	Not applicable	Low
Luck (2016)[37]	Cohort study (Study on Adult Health in Germany, Study on Adult Health in Germany – Mental Health Module & Statistical Offices of the Federal and State Governments)	Yes	Prevalent	Germany	Not reported	AD	7	Education (low), depression (lifetime), diabetes (lifetime), hypertension (ML), obesity (ML), physical inactivity (adult) & smoking (current)	Not reported	30.5% (13.9–45.4)	Moderate
Ma'u (2021)[38]	Cohort study (New Zealand Health Survey 2018/19; Age≥45)	Yes	Prevalent	New Zealand	7,745	All-cause	12	Education (low), depression (LL), diabetes (LL), hearing loss (ML), hypertension (ML), obesity (ML), TBI (ML), alcohol (high, ML), physical inactivity (LL), smoking (LL), social isolation (LL) & air pollution (LL)	Not reported	Total: 47.7%<br>Europeans: 47.6%<br>Māori: 51.4%<br>Pacific peoples: 50.8%<br>Asian: 40.8%	High
MacDonald (2015)[39]	Cohort study (Statistics Canada or the First Nations Regional Health Survey; Age ≥45)	Yes	Prevalent	Canada	Not reported	AD	6	Education (low), diabetes, hypertension (ML), obesity (ML), physical inactivity & smoking	Non-First Nation: 67.1%<br>Total First Nation: 76.1%<br>First Nation on-reserve: 79.6%<br>First Nation off-reserve: 74.9%	Not reported	High
Mayer (2018)[40]	Review (Europe) & Cohort study (Progressi delle Aziende Sanitarie per la Salute in Italia – Progresses by local health units towards a healthier Italy 2013-16; Age: 18-69)	Yes	Prevalent	Europe, Italy	Italy: 146,526	AD, VaD	7	Education (low), depression, diabetes, hypertension (ML), obesity (ML), physical inactivity & smoking	**Europe**<br>VaD: 66.8% (42.5-83.0)<br>**Italy**[∞]<br>AD: 56.8%<br>VaD: 65.9%	**Europe**<br>VaD: 37.8% (21.2-52.5)<br>**Italy***<br>AD: 45.2%<br>VaD: 53.1%	High
Mukadam (2019)[41]	Cohort study (10/66 Study; Age≥65)	No	Prevalent	China, India, Latin America	17,031	All-cause	9	Education (low), depression (LL), diabetes (LL), hearing loss (ML), hypertension (ML), obesity (ML), physical inactivity (LL), smoking (LL) & social contract (low; LL)	Not reported	China: 39.5% (37.5-41.6)<br>India: 41.2% (39.1-43.4)<br>Latin America: 55.8% (54.9-56.7)	Moderate

First author (year)	Study Design and Data Source	Population Representative	Prevalent or Incident Dementia Study	Geographical Location	Sample Size§	Outcome	Number of Modifiable Risk Factors	Modifiable Risk Factors Examined (NB if the study included non-modifiable factors these have not been included)	Combined PAF_{unw} (95%CI) (or individual PAF_{unw}, for those studies that investigated only one factor)	Combined PAF_{w} (95%CI)	Quality
Mukadam (2020)[42]	Review & Cohort study (2014 Health Survey for England)	Yes	Prevalent	England	Not reported	All-cause	4	Diabetes (ML), hearing loss (ML), hypertension (ML) & smoking	Not reported	Not reported	High
Mulligan (2023)[43]	SR and cohort study (UN World Population Prospectus 2019, NCD-Risk, UK Biobank & ARIC studies)	No	Incident	Global (n=186 individual countries and n=6 regions[#])	Not reported	All-cause	1	Hypertension (stratified by age of hypertension: 30-44, 45-54, 55-64 and 65-74 years)	**Global**<br>Total: 15.8% (8.8–22.7)<br>30-44 years: 8.4% (3.4–13.5)<br>45-54 years: 2.9% (1.0–4.9)<br>55-64 years: 2.6% (1.2–4.0)<br>65-74 years: 1.8% (−2.3-6.0)<br>**Africa**<br>Total: 16.5% (8.7–24.3)<br>30-44 years: 10.3% (3.3–17.2)<br>45-54 years: 2.8% (0.9–4.7)<br>55-64 years: 2.2% (1.0–3.4)<br>65-74 years: 1.2% (−1.5-3.9)<br>**Asia**<br>Total: 15.1% (8.5–21.7)<br>30-44 years: 8.3% (3.3–13.3)<br>45-54 years: 2.8% (0.9–4.7)<br>55-64 years: 2.4% (0.9–3.9)<br>65-74 years: 1.6% (−2.2-5.5)<br>**Europe**<br>Total: 17.2% (9.6–24.7)<br>30-44 years: 8.4% (3.5–13.2)<br>45-54 years: 3.3% (1.1–5.6)<br>55-64 years: 3.2% (1.4–4.9)<br>65-74 years: 2.3% (−2.8-7.4)<br>**Latin America/Caribbean**<br>Total: 18.0% (9.4–26.6)<br>30-44 years: 10.48 (3.41–17.56)<br>45-54 years: 3.05 (0.91–5.19)<br>55-64 years: 2.57 (1.05–4.09)<br>65-74 years: 1.87 (−2.37 to 6.12)<br>**North America**<br>Total: 15.4% (7.7–23.1)<br>30-44 years: 7.3% (2.3–12.3)<br>45-54 years: 2.9% (0.7–5.1)<br>55-64 years: 2.9% (1.0–4.5)<br>65-74 years: 2.4% (−3.2-7.9)<br>**Oceania**<br>Total: 14.8% (7.3–22.3)<br>30-44 years: 7.0% (2.2–11.8)<br>45-54 years: 2.7% (0.6–4.8)<br>55-64 years: 2.8% (0.9–4.7)<br>65-74 years: 2.3% (−3.1-7.9)	Not applicable	Critically Low
Nianogo (2022)[44]	Cohort study (US Behavioral Risk Factor Surveillance System Data; Age≥65)	Yes	Prevalent	USA	378,615	AD and Related Dementias	8	Education (low), depression (lifetime), diabetes (lifetime), hearing loss (lifetime), hypertension (ML), obesity (ML), physical inactivity (past month) & smoking (current; ML)	Not reported	Total: 36.9% (36.5-37.3)<br>All women: 30.1% (29.6-30.6)<br>All men: 35.9% (35.3-36.5)<br>All Black: 39.8% (38.6-41.0)<br>Black (women): 41.5% (39.9-42.9)<br>Black (men): 38.4% (36.5-40.3)<br>All American Indian & Alaska: 39.2% (36.1-42.0)	Moderate

First author (year)	Study Design and Data Source	Population Representative	Prevalent or Incident Dementia Study	Geographical Location	Sample Size§	Outcome	Number of Modifiable Risk Factors	Modifiable Risk Factors Examined (NB if the study included non-modifiable factors these have not been included)	Combined PAF_{unw} (95%CI) (or individual PAF_{unw}, for those studies that investigated only one factor)	Combined PAF_{w} (95%CI)	Quality
										All American Indian & Alaska (women): 36.8% (32.8-40.4)<br>All American Indian & Alaska (men): 36.7% (32.4-40.6)<br>All Hispanic: 34.2% (33.0-35.3)<br>Hispanic (women): 37.9% (36.3-39.5)<br>Hispanic (men): 32.5% (30.7-34.1)<br>All White: 28.5% (28.1-28.9)<br>White (women): 28.3% (27.8-28.8)<br>White (men): 34.2% (33.5-34.8)<br>All Asian: 15.8% (13.8-17.8)<br>Asian (women): 14.4% (11.4-17.2)<br>Asian (men): 15.9% (13.1-18.6)	
Norton (2014)[45]	Review and cohort study (Health Survey for England 2010, UK National Institute for Health & Care Excellence, US Census Bureau International Data Base, US National Comorbidity Survey Replication, National Health Interview Survey 2009 (USA), WHO figures, WHO World Mental Health Survey, World Health Survey 2002-2003, Centers for Disease Control and Protection data, Eurostat database, Eurobarometer study & European Health Interview Survey)	Yes	Prevalent	Global, UK, USA & Europe	Not applicable	AD	7	Education (low), depression (lifetime), diabetes (adult), hypertension (ML), obesity (ML), physical inactivity (adult) & smoking (adult; current)	Global: 49.4% (25.7–68.4)<br>USA: 52.7% (25.9–72.8)<br>Europe: 54.0% (27.2–73.7)<br>UK: 52.0% (25.6–71.9)	Global: 28.2% (14.2–41.5)<br>USA: 30.6% (14.5–45.3)<br>Europe: 31.4% (15.3–46.0)<br>UK: 30.0% (14.3–44.4)	High
Oliverira (2019)[46]	Cohort study (National Sample Household Survey and the National Health Survey in Brazil as well as published epidemiological studies from Mozambique and Portugal)	Yes	Prevalent	Brazil, Portugal, Mozambique	Not reported	All-cause	7	Education (low), depression (lifetime), diabetes (adult), hypertension (ML), obesity (ML), physical inactivity (adult) & smoking (adult; current)	Brazil: 55.3% (28.3–74.3)<br>Portugal: 65.8% (36.2–83.4)<br>Mozambique: 44.0% (23.4–61.8)	Brazil: 32.3% (15.8–46.3)<br>Portugal: 40.1% (20.7–55.4)<br>Mozambique: 24.4% (12.9–36.1)	High
Ren (2022)[47]	Cohort study (UK Biobank; Age 35-73)	No	Incident	UK (Women)	239,508	All-cause	11	Education, occupation, low BMI (<18.5), diabetes, hypertension, cerebrovascular disease, respiratory disease, cardiovascular disease, sleepiness, alcohol use & smoking	Not reported	53.4%	High
Ren (2022)[47]	Cohort study (UK Biobank; Age 35-73)	No	Incident	UK (Men)	205,187	All-cause	9	Education, occupation, low BMI (<18.5), diabetes, hypertension, cerebrovascular disease, respiratory disease, sleepiness & alcohol use	Not reported	31.7%	As above

First author (year)	Study Design and Data Source	Population Representative	Prevalent or Incident Dementia Study	Geographical Location	Sample Size§	Outcome	Number of Modifiable Risk Factors	Modifiable Risk Factors Examined (NB if the study included non-modifiable factors these have not been included)	Combined PAF_{unw} (95%CI) (or individual PAF_{unw}, for those studies that investigated only one factor)	Combined PAF_{w} (95%CI)	Quality
Rogers (2009)[48]	Cohort study (Aging, Demographics, and Memory Study – with parental education information in the Health and Retirement Study; Age≥70)	Yes	Incident	USA	856	All-cause	1	Low maternal education (adjusted for age and APOE e4 status)	18.8% (9.4-28.2)	Not applicable	Moderate
Rolandi (2020)[49]	Cohort study (InveCe.Ab; Age 70-74)	No	Incident	Italy	1,100	All-cause	6	Education (low), delirium (LL), diabetes (LL), heart disease (LL), stroke (LL) & physical inactivity (LL)	40%	Not reported	High
Rydén (2019)[50]	Cohort study (Gothenburg H70 Birth Cohort; Age 70, 75, 79)	No	Incident	Sweden	561	All-cause	1	Atrial fibrillation	Total sample: 12.9% (1.0-25.9)<br>Stroke-free: 12.1% (0.4-25.3)	Not applicable	High
Santabarbara (2019a)[51]	SR	Not applicable	Incident	Global	Not applicable	All-cause, AD	1	Depression	All-cause: 8.6% (0.9-17.8)<br>AD: 10.8% (0.3-17.5)	Not applicable	Moderate
Santabarbara (2019b)[52]	Cohort study (Zaragoza Dementia and Depression (ZARADEMP) Study; Age≥55)	Yes	Incident	Spain	4,057	AD	1	Anxiety	6.1% (1.3-16.2)	Not applicable	High
Santabarbara (2020a)[53]	SR	Not applicable	Incident	Global	Not applicable	All-cause	1	Anxiety	3.9% (1.9-6.0)	Not applicable	Moderate
Santabarbara (2020b)[54]	SR	Not applicable	Incident	Global	Not applicable	All-cause	1	Depression	9.0% (4.5-14.1)	Not applicable	Low
Scazufca (2010)[55]	Cohort study (Sao Paulo Ageing & Health Study; Age≥65)	No (but sample socio-demographic characteristics reflect census data)	Prevalent	Brazil	2,003	All-cause	3	Illiteracy, non-skilled occupations & low income (Note: PAF values are age and gender adjusted)	Not reported	Illiterate & non-skilled occupation: 44.8% (21.7-59.6)<br>Non-skilled occupation & low income: 48.4% (28.0-63.0)<br>All three factors: 50.4% (29.9-64.9)	High
Shang (2021)[56]	SR	Not applicable	Incident	Global	Not applicable	All-cause	1	Vision impairment	Global: 4.7% (80%CI: 2.3-7.5)<br>High-income: 2.2% (80%CI: 1.0-3.6)<br>Middle-income: 5.7% (80%CI: 2.9-9.0)<br>Low-income: 6.6% (80%CI: 3.2-10.2)<br>Africa: 6.3% (80%CI: 3.1–9.8)<br>Asia: 5.8% (80%CI: 2.9–8.9)<br>Latin America/Caribbean: 3.5% (80%CI: 1.5–5.9)<br>Oceania: 2.7% (80%CI: 1.2–4.5)<br>Europe: 2.2% (80%CI: 1.0–3.8)<br>North America: 2.2% (80%CI: 0.9–3.6)	Not applicable	High
Shang (2022)[57]	Research Study (UK Biobank; Age 38-73)	No	Incident	UK	471,485	All-cause	1	Multimorbidity score incorporating 33 diseases including: hearing impairment, hypertension, depression, high cholesterol, painful conditions, diabetes, coronary heart disease, obesity, asthma, PD, osteoporosis, dyspepsia, epilepsy, anxiety, stroke, schizophrenia, atrial fibrillation, treated constipation, eczema, diverticulitis, head injury, other cardiac problem, connective tissue disorders, alcohol problems, cataract, multiple sclerosis, psychoactive substance abuse, COPD, CKD, fracture, heart failure, chronic fatigue syndrome & prostate disorders (males only)	**Total sample**<br>All dementia: 51.2% (45.5-56.3)<br>YOD: 64.7% (49.9-75.2)<br>LOAD: 47.4% (40.8-53.2)	Not reported	Moderate

First author (year)	Study Design and Data Source	Population Representative	Prevalent or Incident Dementia Study	Geographical Location	Sample Size§	Outcome	Number of Modifiable Risk Factors	Modifiable Risk Factors Examined (NB if the study included non-modifiable factors these have not been included)	Combined PAF_{unw} (95%CI) (or individual PAF_{unw}, for those studies that investigated only one factor)	Combined PAF_{w} (95%CI)	Quality
Skirbekk (2023)[58]	Cohort study (HUNT Study; Age 70+)	Yes	Incident	Norway	8,706	All-cause	1	Marital status (continuously married vs. unmarried; marriage status was taken at mid-life defined as at ages 44-68 years)	6.0% (2.3-9.5)	Not applicable	High
Smith (2023)[59]	Cohort study (National Health and Aging Trends Study; Age 65+)	Yes	Prevalent	USA	2,470	All-cause	1	Audiometric hearing loss	**Any loss**<br>Total sample: 18.7% (−5.3-40.1)<br><br>**Mild loss**<br>Total sample: 3.9% (−8.7-15.8)<br><br>**Moderate or greater loss**<br>Total sample: 16.9% (4.1-28.7)<br>Age <80: 15.1% (1.3-30.0)<br>Age≥80: 17.7% (-1.4-34.2)<br>Men: 40.5% (19.5-57.2)<br>Women: 3.2% (-12.7-17.9)<br><br>**No hearing aid use (vs use)**<br>Aid sample: 22.5% (2.7-41.0)	Not applicable	High
Smith (2023)[60]	Cohort study (Atherosclerosis Risk in Communities Study; Age 45-64)	No	Incident	USA	45-54: 7,668<br>55-64: 12,129<br>65-74: 6,712<br>75-84: 2,254	All-cause	1	Hypertension	**Dementia by age 80 years**<br>Non-normal (45-54): 15.3% (6.9–22.3)<br>Non-normal (55-64): 19.1% (9.9–26.9)<br>Non-normal (64-74): 19.9% (−4.4–38.5)<br><br>Stage 1 PAR Range: 2.3%–3.4%<br>Stage 1 (55-64): 3.4% (0.9-5.5)<br>Stage 1 (65-74): Null<br><br>Stage 2 (45-54): 11.9% (6.4-16.2)<br>Stage 2 (55-64): 14.4% (8.0-19.8)<br>Stage 2 (64-74): 21.3% (2.8-35.4)<br><br>**Dementia by age 90 years**<br>Non-normal (45-54): 13.8% (6.6-20.0)<br>Non-normal (55-64): 12.9% (7.0-18.2)<br>Non-normal (64-74): 10.9% (0.02-21.0)<br>Non-normal (75-84): Null	Not applicable	High
Suemoto (2023)[61]	Cohort study (Brazilian Longitudinal Study of Aging (Age≥50)	Yes	Prevalent	Brazil	9,412	All-cause	12	Education (low), depression, diabetes, hearing loss, hypertension, obesity, TBI, alcohol (high), physical inactivity, smoking, social isolation & air pollution	Total: 77.6% (76.8-78.4)<br>Rich: 78.1% (77.6-78.6)<br>Poor: 77.0% (75.7-78.3)<br>White: 76.7% (75.3-78.1)<br>Black: 78.0% (76.9-79.1)	Total: 48.2% (47.2-49.2)<br>Rich: 49.2% (47.9-50.7)<br>Poor: 54.0% (52.5-55.5)<br>White: 47.9% (46.3-49.6)<br>Black: 46.9% (45.5-48.3)	Moderate
Suh (2016)[62]	Cohort study (2008 Nationwide Survey on Dementia Epidemiology of Korea; other data resources were used for other sites, but paper	Yes, for South Korea (but not other sites)	Prevalent	South Korea, Latin American, South Asian/Middle East & Africa	Not reported	All-cause	1	Illiteracy (in 2015)	South Korea: 15.6%<br>Latin America: 15.6-56.6%$<br>South Asia/Middle East: 5.7-61.5%$<br>Africa: 13.2-72.9%$	Not applicable	High

First author (year)	Study Design and Data Source	Population Representative	Prevalent or Incident Dementia Study	Geographical Location	Sample Size§	Outcome	Number of Modifiable Risk Factors	Modifiable Risk Factors Examined (NB if the study included non-modifiable factors these have not been included)	Combined PAF_{unw} (95%CI) (or individual PAF_{unw}, for those studies that investigated only one factor)	Combined PAF_{w} (95%CI)	Quality
	lacks in-depth information; Age<65)										
Thompson (2022)[63]	Cohort study (Dementia Prevalence Survey 2015-18 & the Zenadth-Kes Health Partnership; Age≥40)	No	Prevalent	Australia (First Nations peoples, including Torres Strait Islander and Aboriginal peoples in Far North Queensland)	371	All-cause	12	Set 1: Education (low), depression (LL), diabetes (LL), hearing loss (ML), hypertension (ML), obesity (ML), TBI (ML), alcohol (excessive; ML), physical inactivity (LL), smoking (LL) & social isolation (LL)<br><br>Set 2: Set 1 + chronic kidney disease	Set 1: 75.4%<br>Set 2: 77.9%	Set 1: 52.8% (47.8-57.9)<br>Set 1 (Age standardised): 52.1% (47.1-57.2)<br>Set 2: 52.2%	Moderate
Tomata (2019)[64]	Cohort study (Ohsaki Cohort 2006 Study and Long-term Care Insurance database; Age≥65)	Yes	Incident	Japan	13,990	All-cause	1	Physical inactivity (time spend walking)	All: 14.0%<br><75 years: 17.7%<br>≥75 years: 13.8%	Not applicable	High
Tomata (2020)[65]	Cohort study (Swedish Twin Registry; Age≥65)	No	Incident	Sweden	9,017	All-cause	9	Education (low), depression (LL), diabetes (LL), hearing loss (LL), hypertension (LL), obesity (LL), physical inactivity (LL), smoking (LL) & living alone (LL)<br><br>Model 1 = Nine factors + (age & sex)<br>Model 2 = Model 1 + cognition	**Total Sample**<br>Model 1: 14.7% (3.0-25.0)<br>Model 2: 10.4% (-2.3-21.5)<br>**Life-course Variables:**<br>Model 1: 15.1% (-4.6-31.0)<br>Model 2: 12.6% (-7.8-29.1)<br>**Later-life Variables**<br>Model 1: 18.2% (-0.4-33.4)<br>Model 2: 16.0% (-3.3-31.6)	Not reported	high
Vagelatos (2013)[66]	SR	Not applicable	Incident	Global, USA	Not applicable	AD	1	Diabetes (adulthood)	USA: 8%<br>Global: 6%	Not applicable	Low
Vergara (2022)[67]	Cohort study (2017 Chilean National Health Survey; Age≥45)	Yes	Prevalent	Chile	3,379	All-cause	9	Education (low), depression, diabetes, hearing loss, hypertension, obesity, alcohol (high), physical inactivity & smoking	Not reported	Overall: 45.8% (42.2-49.3)<br>ML: 38.8% (32.2-45.4)<br>LL: 56.0% (48.1-64.0)<br>LL (excluding high blood pressure & obesity): 32.8% (26.1-39.4)<br>ML (women): 43.2% (36.2-50.4)<br>LL (women): 65.1% (57.3-73.0)<br>ML (men): 34.4% (28.6-40.2)<br>LL (men): 54.3% (45.8-62.6)	Moderate
Wallace (2021)[68]	Cohort study (CC75C)	Yes	Incident	UK	542	All-cause	1	Frailty (n=39 items; vision problems, hearing problems, arthritis/rheumatism, back pain, chest pain. shortness of breath, weakness in arm or leg, unsteady on feet, falls, how do you manage with using a telephone, how do you manage with shopping, how do you manage with preparing meals, how do you manage with housework, how do you manage with laundry, how do you manage with walking, do you use a walking stick or other aid, how do you manage with bathing or showering, how do you manage with reaching up to comb your hair (or shave) or down to cut your toenails, how do you manage with dressing and undressing, how do you manage with getting to the toilet on time, ow do you manage taking medicines, how do you manage with finance, how do you manage with transportation, how do you manage with feeding?/eating, angina, heart attack, problems with circulation in your legs, high blood pressure, chronic bronchitis, stroke, stroke symptoms, thyroid problems,	14.2%	Not applicable	High

First author (year)	Study Design and Data Source	Population Representative	Prevalent or Incident Dementia Study	Geographical Location	Sample Size§	Outcome	Number of Modifiable Risk Factors	Modifiable Risk Factors Examined (NB if the study included non-modifiable factors these have not been included)	Combined PAF_{unw} (95%CI) (or individual PAF_{unw}, for those studies that investigated only one factor)	Combined PAF_{w} (95%CI)	Quality
								migraine/headaches, trouble with nerves, have you had to go into hospital to stay because of any of these difficulties in the last 6 months, how many times have you been in hospital in the last 6 months/year, keep fit, walking &gardening)			
Weiss (2021)[69]	Cohort study (Health and Retirement Study; Age≥51)	Yes	Incident	USA	16,234	All-cause	14	Combined PAF based on three merged domains: socio-economic resources, lifestyle characteristics and medical conditions.<br><br>The domains are made up of different combinations of n=14 variables: education (low), occupation, low neighbourhood safety, food insecurity, physical inactivity, BMI, smoking, alcohol, lonely, diabetes, hypertension, stroke, heart condition & poor hearing	White (men): 45.4% (35.5-53.8)<br>White (women): 41.0% (33.0-47.9)<br>Black (men): 78.7% (67.2-86.2)<br>Black (women): 64.1% (51.9-73.2)<br>Hispanic (men): 78.2% (57.0-89.0)<br>Hispanic (women): 90.3% (79.7-95.4)	Not reported	Moderate
Woo (2014)[70]	Cohort study (Department of Health & Census and Statistics Department; Age≥15)	Yes	Incident	Hong Kong	Not reported	AD	7	Education (low; ≥15 years), depression (≥60 years), diabetes (20-79 years), hypertension (≥15 years), obesity (18-64 years), physical inactivity (18-64 years) & smoking (≥15 years)	49.3%	Not reported	Low
Wu (2022)[71]	Cohort study (Sixth National Health Service Survey, 2018; Age≥60)	Yes	Prevalent	China – Jiangxi Province	2,713	All-cause	9	Education (low), diabetes, hearing loss, hypertension, obesity, physical inactivity, smoking, social contact (low) & no spouse	Not reported	66.8% (59.6-72.3)	High
Xu (2015)[72]	SR	Not applicable	Incident	Global	Not applicable	AD	9	Education (low), carotid atherosclerosis, depression, diabetes (in Asian population), frailty, total homocysteine levels (high), hypertension (SBP≥160 mmHg), obesity (ML) & current smoking (in Asian population)	66%	Not reported	Moderate
Zhang (2021)[73]<br><br>[IN CHINESE]	Cohort study (Hubei Elderly Memory Cohort Study; Age≥65)	No	Prevalent	China (Wuhan City; Urban only region)	1,908	All-cause	7	Education, diabetes, hypertension, overweight/obesity, physical inactivity, smoking & lack of social interaction	Not reported	Urban only: 39.5%	Moderate
Zhang (2021)[73]<br><br>[IN CHINESE]	Cohort study (Hubei Elderly Memory Cohort Study; Age≥65)	No	Prevalent	China (Wuhan City)	Total: 3,361<br>Rural: 1,453	All-cause	6	Education, diabetes, hypertension, overweight/obesity, smoking & lack of social interaction	Not reported	Urban & rural combined: 28.8%<br>Rural only: 31.7%	As above
Zhang (2023)[74]***	Cohort study (UK Biobank; Age 37-73)	No	Incident	UK	344,324	All-cause	6	Six domain scores including lifestyles, medical history, local environment, psychosocial factors, physical measures & SES	Not reported	M1: 47.0%<br>M2: 72.6%	High

Key

95%CI = 95% Confidence Interval; AD=Alzheimer's disease; APOE=Apolipoprotein e4; BMI=Body mass index; CNS=Central nervous system; CKD=Chronic kidney disease; COPD=Chronic obstructive pulmonary disease; LL=Later-life; ML=Mid-life; Mod=Moderate; pop=Population; PAF=Population attributable fraction; PAF_{unw}=Population attributable fraction (unweighted); PAF_{w}=Population attributable fraction (weighted for commonality between factors); PD=Parkinson's disease; RR=Relative risk; TBI=Traumatic brain injury; SABE= Survey on Health, Well-bring and Aging in Latin America and the Caribbean; SBP=Systolic blood pressure; SES=Socio economic status; SR=Systematic review; TIA=Transient ischemic attack; UK=United Kingdon; USA=United States of America; VaD=Vascular dementia; WHO SA-SAGE=World Health Organisation South Africa data from the Study on global AGEing and adult health

Notes

§ We use the term "not applicable" for those studies where PAF estimates have been created based on reviews/meta-analyses

$ Range of values based on smallest and largest confidence intervals.

+ Regional estimates for each risk are provided; n=21 sites including Central Asia, Central Europe, Eastern Europe, Australasia, High-income Asia Pacific, High-income North America, Southern Latin America, Western Europe, Andean Latin America, Caribbean, Central Latin America, Tropical Latin America, North Africa and Middle East, South Asia, East Asia, Oceania, Southeast Asia, Central sub-Saharan Africa, Eastern sub-Saharan Africa, Southern sub-Saharan Africa, Western sub-Saharan Africa).

∞ Combined estimates are available for the different regions of Italy (n=21 regions including adjusted and not-adjusted PAR values) but are not extracted here.

*** Zhang (2023) M1 = Shifting unfavourable profiles to intermediate and favourable ones; and M2 = Shifting all factors to the favourable tertile.

See the original publication for individual country results

Appendix 6 Individual countries in those studies that have reported PAF estimates for n>50 countries

	**Hypertension**	**Physical Inactivity**	**Physical Inactivity**
	**Mulligan (2023)[43]**	**Katzmarzky (2022)[28]**	**Feter (2022)[17]**
1	Afghanistan	Algeria	Benin
2	Albania	American Samoa	Burkina Faso
3	Algeria	Andorra	Cape Verde
4	Angola	Argentina	Cameroon
5	Antigua and Barbuda	Armenia	Central African Republic
6	Argentina	Australia	Chad
7	Armenia	Austria	Comoros
8	Australia	Bahamas	Congo
9	Austria	Bangladesh	Côte d'Ivoire
10	Azerbaijan	Barbados	Democratic Republic of the Congo
11	Bahamas	Belarus	Egypt
12	Bahrain	Belgium	Eritrea
13	Bangladesh	Benin	Eswatini
14	Barbados	Bermuda	Ethiopia
15	Belarus	Bhutan	Guinea
16	Belgium	Bosnia & Herzegovina	Ghana
17	Belize	Botswana	Kenya
18	Benin	Brazil	Lesotho
19	Bhutan	British Virgin Islands	Liberia
20	Bolivia	Brunei Darussalam	Madagascar
21	Bosnia and Herzegovina	Bulgaria	Malawi
22	Botswana	Burkina Faso	Mali
23	Brazil	Cabo Verde	Mauritania
24	Brunei Darussalam	Cambodia	Morocco
25	Bulgaria	Cameroon	Mozambique
26	Burkina Faso	Canada	Namibia
27	Burundi	Cayman Islands	Niger
28	Cabo Verde	Central African Republic	Nigeria
29	Cambodia	Chad	Rwanda
30	Cameroon	Chile	Sao Tome and Principe
31	Canada	China	Senegal
32	Central African Republic	Colombia	Sierra Leoa
33	Chad	Comoros	South Africa
34	Chile	Congo	Uganda
35	China	Cook Islands	United Republic of Tanzania
36	Colombia	Costa Rica	The Gambia
37	Comoros	Cote d'Ivoire	Zambia
38	Congo	Croatia	Zimbabwe
39	Costa Rica	Cuba	Guatemala
40	Cote d'Ivoire	Cyprus	Bhutan
41	Croatia	Czech Republic	Cambodia
42	Cuba	Democratic Republic of the Congo	Cayman Islands
43	Cyprus	Denmark	Indonesia
44	Czech Republic	Dominica	Iraq
45	Denmark	Dominican Republic	Kiribati
46	Djibouti	Ecuador	Kyrgyzstan
47	Dominican Republic	Egypt	Lao
48	DR Congo	Eritrea	Myanmar
49	Ecuador	Estonia	Nepal
50	Egypt	Ethiopia	Pakistan
51	El Salvador	Fiji	Timor-Leste
52	Equatorial Guinea	Finland	Vietnã
53	Eritrea	France	Yemen
54	Estonia	French Polynesia	Federated States of Micronesia
55	Ethiopia	Gabon	Papua New Guinea
56	Fiji	Gambia	Togo
57	Finland	Georgia	Vanuatu
58	France	Germany	
59	Gabon	Ghana	
60	Gambia	Greece	
61	Georgia	Grenada	
62	Germany	Guatemala	
63	Ghana	Guinea	

	Hypertension	Physical Inactivity	Physical Inactivity
	**Mulligan (2023)[43]**	**Katzmarzky (2022)[28]**	**Feter (2022)[17]**
64	Greece	Hungary	
65	Grenada	India	
66	Guatemala	Indonesia	
67	Guinea	Iran (Islamic Republic of)	
68	Guinea Bissau	Iraq	
69	Guyana	Ireland	
70	Haiti	Italy	
71	Honduras	Jamaica	
72	Hungary	Japan	
73	Iceland	Jordan	
74	India	Kazakhstan	
75	Indonesia	Kenya	
76	Iran	Kiribati	
77	Iraq	Kuwait	
78	Ireland	Kyrgyzstan	
79	Israel	Lao People's Democratic Republic	
80	Italy	Latvia	
81	Jamaica	Lebanon	
82	Japan	Lesotho	
83	Jordan	Liberia	
84	Kazakhstan	Libya	
85	Kenya	Lithuania	
86	Kiribati	Luxembourg	
87	Kuwait	Madagascar	
88	Kyrgyzstan	Malawi	
89	Lao PDR	Malaysia	
90	Latvia	Maldives	
91	Lebanon	Mali	
92	Lesotho	Malta	
93	Liberia	Marshall Islands	
94	Libya	Mauritania	
95	Lithuania	Mauritius	
96	Luxembourg	Mexico	
97	Macedonia (TFYR)	Micronesia (Federated States of)	
98	Madagascar	Mongolia	
99	Malawi	Morocco	
100	Malaysia	Mozambique	
101	Maldives	Myanmar	
102	Mali	Namibia	
103	Malta	Nauru	
104	Mauritania	Nepal	
105	Mauritius	Netherlands	
106	Mexico	New Zealand	
107	Micronesia (Federated States of)	Niger	
108	Moldova	Nigeria	
109	Mongolia	Niue	
110	Montenegro	Norway	
111	Morocco	Oman	
112	Mozambique	Pakistan	
113	Myanmar	Palau	
114	Namibia	Papua New Guinea	
115	Nepal	Paraguay	
116	Netherlands	Philippines	
117	New Zealand	Poland	
118	Nicaragua	Portugal	
119	Niger	Qatar	
120	Nigeria	Republic of Korea	
121	North Korea	Republic of Moldova	
122	Norway	Romania	
123	Occupied Palestinian Territory	Russian Federation	
124	Oman	Rwanda	
125	Pakistan	Saint Kitts & Nevis	
126	Panama	Saint Lucia	
127	Papua New Guinea	Samoa	
128	Paraguay	Sao Tome & Principe	

	Hypertension	Physical Inactivity	Physical Inactivity
	Mulligan (2023)[43]	Katzmarzky (2022)[28]	Feter (2022)[17]
129	Peru	Saudi Arabia	
130	Philippines	Senegal	
131	Poland	Serbia	
132	Portugal	Seychelles	
133	Puerto Rico	Sierra Leone	
134	Qatar	Singapore	
135	Romania	Slovakia	
136	Russian Federation	Slovenia	
137	Rwanda	Solomon Islands	
138	Saint Lucia	South Africa	
139	Saint Vincent and the Grenadines	Spain	
140	Samoa	Sri Lanka	
141	Sao Tome and Principe	State of Palestine	
142	Saudi Arabia	Suriname	
143	Senegal	Swaziland	
144	Serbia	Sweden	
145	Seychelles	Switzerland	
146	Sierra Leone	Tajikistan	
147	Singapore	Thailand	
148	Slovakia	Timor-Leste	
149	Slovenia	Togo	
150	Solomon Islands	Tokelau	
151	Somalia	Tonga	
152	South Africa	Trinidad and Tobago	
153	South Korea	Tunisia	
154	South Sudan	Turkey	
155	Spain	Tuvalu	
156	Sri Lanka	Uganda	
157	Sudan	Ukraine	
158	Suriname	United Arab Emirates	
159	Swaziland	United Kingdom	
160	Sweden	United Republic of Tanzania	
161	Switzerland	United States of America	
162	Syrian Arab Republic	Uruguay	
163	Taiwan	Uzbekistan	
164	Tajikistan	Vanuatu	
165	Tanzania	Venezuela (Bolivarian Republic of)	
166	Thailand	Viet Nam	
167	Timor-Leste	Zambia	
168	Togo	Zimbabwe	
169	Tonga		
170	Trinidad and Tobago		
171	Tunisia		
172	Turkey		
173	Turkmenistan		
174	Uganda		
175	Ukraine		
176	United Arab Emirates		
177	United Kingdom		
178	United States of America		
179	Uruguay		
180	Uzbekistan		
181	Vanuatu		
182	Venezuela		
183	Vietnam		
184	Yemen		
185	Zambia		
186	Zimbabwe		

Appendix 7 Risk of bias scores

Table 1 Risk of bias scores generated using the Newcastle-Ottawa Scale (NOS) for cohort studies

Reference	Selection				Comparability		Outcomes			Total
	1	*2*	*3*	*4*	*5*	*6*	*7*	*8*	*9*	*/9*
de Bruijn (2015)[11]	0	1	1	1	1	1	1	1	1	8
Dodge (2011)[13]	0	1	0	1	1	1	1	1	1	7
Esteban-Cornejo (2022)[16]	0	1	1	1	1	1	1	1	0	7
Hagstrom (2014)[20]	0	0	1	1	1	1	1	1	1	7
Hegelund (2021)[22]	1	1	1	1	1	1	1	1	1	9
Hodis (2020)[23]	0	1	1	1	1	1	1	1	1	8
Mingyue Hu[25]	1	1	0	1	1	1	1	1	1	8
Johannesdottir (2022)[26]	1	0	1	1	1	1	1	0	1	7
Kotaki (2019)[30]	0	1	0	1	1	1	1	1	1	7
Launer (2010)[31]	0	1	1	1	1	1	1	1	1	8
Ren (2022)[47]	0	1	0	1	1	1	1	1	1	7
Rogers (2009)[48]	1	0	0	1	1	1	1	1	0	6
Rolandi (2020)[49]	0	1	1	1	1	1	1	1	1	8
Ryden (2019)[50]	0	1	1	1	1	1	1	1	1	8
Santabarbara (2019b)[52]	1	1	1	1	1	1	1	1	0	8
Scazufca (2010)[55]	0	1	1	1	1	0	1	1	1	7
Shang (2022)[57]	0	1	1	1	1	1	1	1	0	7
Shirbekk (2023)[58]	1	1	1	0	1	1	1	1	1	8
Smith (2023)[60]	0	1	1	1	1	1	1	1	0	7
Tomata (2019)[64]	1	1	0	1	1	1	1	0	1	7
Tomata (2020)[65]	0	1	1	1	1	1	1	1	1	8
Wallace (2021)[68]	1	1	1	1	1	1	1	1	1	9
Weiss (2021)[69]	1	1	0	1	1	0	0	1	1	6
Woo (2014)[70]	1	1	1	0	0	0	0	0	0	3
Zhang (2023)[74]	0	1	1	1	1	1	1	1	1	8

Notes 1=Representativeness of the exposed cohort; 2=Selection of the non-exposed cohort; 3=Ascertainment of exposure; 4=Demonstration that outcome of interest was not present at start of study; 5=Study controls for age, sex, marital status; 6=Study controls for other factors; 7=Comparability of cohorts based on the design or analysis controlled for confounders; 8=Was follow-up long enough for outcomes to occur; and 9=Adequacy of follow-up of cohorts

Table 2 Risk of bias scores generated using the Newcastle-Ottawa Scale (NOS) for cross-sectional studies

Reference	Selection				Comparability		Outcomes		Total
	1*	***2****	***3****	***4*****	**5***	***6****	***7*****	***8****	***/10***
Ashby-Mitchell (2017)[1]	1	1	1	1	0	1	1	1	7
Ashby-Mitchell (2018)[2]	1	1	0	1	0	1	1	1	6
Ashby-Mitchell (2020)[3]	1	1	1	1	0	1	1	1	7
Bobrow (2021)[6]	1	1	1	1	1	1	1	1	8
Borelli (2022[7] & 2023[8])	1	1	1	1	1	0	0	1	6
Bothongo (2022)[9]	1	1	1	1	1	1	2	1	9
Ehrlich (2022)[15]	1	1	1	1	0	1	0	1	6
Feter (2022)[17]	0	0	0	1	0	1	1	1	4
Gardner (2023)[18]	0	1	1	1	1	1	0	1	6
Fei-fei Hu (2022)[24]	1	1	0	1	1	1	2	1	8
Jørgensen (2023)[27]	1	1	1	1	1	1	1	1	8
Lee (2022)[32]	1	0	1	1	0	1	0	1	5
Liu (2020)[33]	1	1	1	1	1	0	1	1	7
Luck (2016)[37]	1	0	0	1	0	1	0	1	4
Ma'u (2021)[38]	1	1	1	1	1	1	0	1	7
MacDonald (2015)[39]	1	1	1	1	0	1	1	1	7
Mayer (2018)[40]	1	1	1	1	1	1	0	1	7
Mukadam (2019)[41]	0	1	1	1	0	1	1	1	6
Mukadam (2020)[42]	1	1	1	1	1	1	1	1	8
Nianogo (2022)[44]	1	1	0	1	1	1	0	1	6
Norton (2014)[45]	1	1	1	1	0	1	1	1	7
Oliviera (2019)[46]	1	1	1	1	0	1	1	1	7
Smith (2023)[59]	1	1	0	1	1	1	2	1	8

Reference	Selection				Comparability		Outcomes		Total
	1*	***2****	***3****	***4*****	***5****	***6****	***7*****	***8****	***/10***
Suemoto (2022)[61]	1	1	0	1	0	1	0	1	5
Suh (2016)[62]	1	1	1	1	1	0	1	1	7
Thompson (2022)[63]	0	0	0	2	1	0	2	1	6
Vergara (2022)[67]	1	1	0	1	1	1	0	1	6
Wu (2022)[71]	1	1	1	1	1	1	1	1	8
Zhang (2021)[73]	0	1	0	1	1	1	0	1	5

Notes 1=Representativeness of the sample; 2=Sample size justified; 3=Non-respondents; 4=Ascertainment of the exposure (risk factor); 5=Confounding factors controlled including age, sex, etc.; 6=Other predictors/confounders controlled; 7=Assessment of outcome; and 8=Statistical test

Table 3 Risk of bias scores generated using the “A Measurement Tool to Assess Systematic Reviews, Version 2” (AMSTAR-2) for Systematic Reviews and Meta-Analyses

Reference	*1*	*2**	*3*	*4**	*5*	*6*	*7**	*8*	*9**	*10*	*11**	*12*	*13*	*14*	*15**	*16*	Total
Beydoun (2014)[5]	Y	Y	Y	N	N	N	Y	Y	N	N	Y	N	N	Y	Y	Y	9
Bubu (2017)[10]	Y	Y	Y	Y	Y	Y	Y	Y	Y	Y	Y	Y	Y	Y	Y	Y	16
Desai (2020)[12]	Y	Y	Y	Y	Y	Y	Y	Y	Y	N	Y	Y	N	Y	Y	Y	14
Dragioti (2022)[14]	Y	Y	Y	Y	Y	Y	Y	Y	Y	Y	Y	Y	N	N	N	Y	13
Gardner (2023)[18]	Y	Y	Y	Y	Y	Y	Y	N	Y	Y	Y	Y	Y	Y	Y	Y	15
Hazar (2016)[21]	Y	Y	Y	Y	Y	Y	Y	Y	Y	N	PY	PY	N	PY	N	Y	11.5
Kloppenborg (2008)[29]	Y	Y	Y	N	N	N	Y	Y	Y	Y	PY	PY	0	PY	0	0	8.5
Loef (2013)[36]	Y	Y	Y	Y	N	N	N	Y	N	Y	Y	N	N	Y	Y	Y	10
Mulligan (2023)[43]	Y	Y	Y	N	Y	Y	Y	N	N	Y	PY	PY	N	N	N	Y	9
Santabarbara (2019a)[51]	Y	Y	Y	Y	Y	Y	Y	Y	Y	N	Y	Y	Y	Y	Y	N	14
Santabarbara (2020a)[53]	Y	Y	Y	Y	N	N	Y	Y	Y	Y	Y	N	Y	Y	Y	Y	13
Santabarbara (2020b)[54]	Y	Y	Y	N	Y	Y	Y	Y	Y	N	Y	Y	Y	Y	Y	Y	14
Shang (2021)[56]	Y	Y	Y	Y	Y	Y	Y	Y	Y	Y	Y	Y	N	N	Y	Y	14
Vagelatos (2013)[66]	Y	Y	Y	Y	N	N	N	Y	N	N	Y	N	N	N	Y	Y	8
Xu (2015)[72]	Y	Y	Y	Y	N	N	Y	Y	Y	N	Y	Y	N	Y	Y	Y	12

Notes 1=PICO Components; 2=A priori design; 3=Study design explanation; 4=Comprehensive search strategy; 5=Duplicate study selection; 6=Duplicate data extraction; 7=Details of excluded studies; 8=Description of included studies; 9=Satisfactory risk of bias assessment; 10=Funding sources reported; 11=Methods for data collection; 12=Risk of bias impact on meta-analysis; 13=Risk of bias impact on interpretation; 14=Heterogeneity; 15=Publication bias; and 16=Reports conflict of interest

Key

* Critical AMSTAR-2 items

Y=Met criteria (Green; SCORE=1)

N=Didn’t meet criteria (Red; SCORE=0)

PY=Partial yes in meeting criteria (Yellow; SCORE=0.5)

Table 4 Risk of bias scores generated using the Scale for the Assessment of Narrative Review Articles (SANRA)

Reference	*1*	*2*	*3*	*4*	*5*	*6*	Total /12
Barnes (2011)[4]	2	2	1	2	2	2	11
GDB 2019 Dementia Collaborators (2021)[19]	2	2	2	2	1	2	11
Katzmarzyk (2022)[28]	2	2	1	2	2	2	11
Livingston (2017)[34]	2	2	1	2	2	2	11
Livingston (2020)[35]	2	2	1	2	2	2	11

Notes 1=Justification of article's importance; 2=Statement of concrete aims or formulation of questions; 3=Description of the literature search; 4=Referencing; 5=Scientific reasoning; and 6=Appropriate presentation of data. Each item is worth 2 points

Appendix 8 The risk factors (n=61*) investigated in the different studies

Socio-economic Factors

Education, food insecurity, household wealth, illiteracy, low income, low maternal education and occupation

Environmental Factors

Air pollution, area deprivation and low neighbourhood safety

Lifestyle Factors

Alcohol use, fish intake, physical activity and smoking

Social Factors

Marital status and social isolation/loneliness

Medication and Drug Use

Benzodiazepine use, psychoactive substance abuse and polypharmacy

Health Related Factors

Anxiety, asthma, atrial fibrillation, cardiac conditions (e.g., heart disease, coronary heart disease, cardiac problems, heart failure), carotid atherosclerosis, cataract, cerebrovascular disease, chronic fatigue syndrome, connective tissue disorders, constipation (treated), COPD, delirium, depression, diabetes, diverticulitis, dyspepsia, eczema, epilepsy, fracture, frailty, handgrip strength, hearing loss, herpes zoster, hypertension, kidney disease (chronic), multiple sclerosis, olfactory decline, osteoporosis, painful conditions, Parkinson's disease, prostrate conditions (men-only), respiratory disease, schizophrenia, sleep problems, stroke, traumatic brain injury, vision impairment and weight (including underweight, overweight and obesity)

Blood-based Biomarkers

Dyslipidaemia, homocysteine level, hypercholesterolemia and parathyroid hormone

* Zhang (2023)[74] reported PAF values for six domains including lifestyles, medical history, local environment, psychosocial factors, physical measures, and socio-economic status created using a combination of n=62 factors. These have not been added to the figure as no individual PAF values were calculated by the authors. The n=62 factors include (1) Townsend deprivation score; (2) education; (3) employment status; (4) total household income; (5) tea intake; (6) water intake; (7) time spent outdoors; (8) number of sexual partners; (9) physical activity; (10) time spent on tv or computers; (11) glucosamine supplements; (12) vitamin b supplements; (13) folic acid or folate supplements; (14) fruit consumption; (15) leisure or social activity; (16) nap during day; (17) insomnia; (18) narcolepsy; (19) exposure to tobacco smoke; (20) alcohol consumption; (21) sleep duration; (22) matter air pollution; (23) greenspace percentage buffer; (24) water percentage buffer; (25) natural environment percentage buffer; (26) varicella; (27) anaemia; (28) obesity; (29) dyslipidaemia; (30) anxiety; (31) severe stress; (32) sleep disorders; (33) other cataract; (34) atrial fibrillation; (35) GI disorders (36) spondylosis; (37) disorders of urinary system; (38) dental problems; (39) diabetes; (40) coronary heart disease; (41) stroke; (42) depression; (43) hypertension; (44) vision impairment; (45) disability; (46) traumatic brain injury; (47) body fat percent; (48) basal metabolic rate; (49) trunk fat percent; (50) diastolic blood pressure; (51) arm fat percent; (52) hand grip strength; (53) lung function; (54) mood swings; (55) miserableness; (56) fed up feelings; (57) nervous feelings; (58) worried feelings; (59) tense highly strung; (60) suffer from nerves; (61) isolation; and, (62) able to confide rarely.

Appendix 9 Measurement of all individual factors included in the meta-analysis (alphabetically ordered by study; n=48 studies with data to include in the meta-analysis)

Reference	No of risks meta-analysed	Sample Age in years	PAF Formula used	Quality Rating	Low education	Hearing	TBI	Hypertension	Alcohol	Obesity	Smoking	Depression	Social Isolation	PA	Diabetes
Ashby-Mitchell (2018)[2]	6	≥25	Levin's formula	Mod	Proportion of adults < a secondary school education			Mid-life prevalence in adults (45–64 years)		Mid-life obesity (BMI ≥30; 45–64 years)	Proportion of adult smokers			<150 minutes of moderate intensity per week (Adults)	Adult prevalence of type 2 diabetes
Ashby-Mitchell (2017)[1]	7	5+	Levin's formula	High	Proportion of adults with SR of primary and/or secondary school education			Mid-life prevalence in adults based on SR (45–54 years)		Mid-life obesity (BMI ≥30; 45–54 years)	Proportion of adult smokers	Lifetime prevalence of major depression using DSM or ICD criteria (based on questionnaire data including diagnostic interviews and psychological distress scales)		SR adults past 7 days and pedometer data (150–300 min moderate intensity or 75–150 min vigorous intensity, or an equivalent combination of both, each week)	Adult prevalence of diagnosed diabetes (SR and biomedical data)
Ashby-Mitchell (2020)[3]	5	≥15	Levin's formula	High	Proportion of adults with SR of primary and/or secondary school education						Proportion of adult smokers (SR)	Presence of ≥5 depressive symptoms and/or expressions of suicidal ideations (DSM-V criteria)		SR physical fitness activity ≤6 times/week	Adult prevalence (SR and biomedical data)
Barnes (2011)[4]	7	N/A	Levin's formula	High	No formal school, only primary school or <12 years of high school for people ≥25 years (USA)			Mid-life		BMI ≥30 (Mid-life)	Current (≥15 years)	Lifetime prevalence		Prevalence of inactivity in the total population	Adult (≥18 years)
Beydoun (2014)[5]	3	N/A	Levin's formula	Low	<8 years						Current or ever vs. never (Mid-life)			Different criteria (high defined as a ≥3 times/week, ≥2/week, ≥2 vs. <2-mile walk/day or other definitions combining frequency and intensity of activity)	
Bobrow (2021)[6]	8	Adults	Levin's formula	High	International Standard Classification of Education level of ≤2 (pre-primary, primary, and lower secondary education)			SBP >140 or DBP >90 (35-64 years)		BMI ≥30 (35-64 years)	Current daily cigarette smoking	Reporting symptoms during the past 12 months	Having over or going to visit friends or relatives less than once or twice per month and going out fewer than once or twice per month	Don't do either 20 min of vigorous activity on ≥3 days or 30 min of moderate activity on ≥5 days per week	HbA1c ≥6.5% (20-79 years)
Borelli (2022)[7] and Borelli (2023)[8]	10	50-105	Levin's formula	Mod	≤4 years (primary education in Brazil)	SR hearing impairment		SR known diagnosis of hypertension by a physician	SR drinking above 168g of alcohol/week	BMI≥30	SR current smoking (later-life)	SR previous medical history (diagnosis) of major depression	SR social contact frequency less than once/month with relatives, friends, or outdoor group activities	SR less than 3 days a week of walking or any physical exercise	SR known diagnosis of diabetes by a physician
Bothongo (2022)[9]	6	27-103	Levin's formula	High		Present (Electronic Health Record)	Present (Electronic Health Record)	Present (Electronic Health Record)			Present or being an ex-smoker (Electronic Health Record)	Present (Electronic Health Record)			Present (Electronic Health Record)
de Bruijn (2015)[11]	3	≥55	Interactive Risk Attributable Program (US National Cancer Institute)	High	Primary education or lower vocational education			Blood pressure ≥140/90 or use of blood pressure lowering medication, prescribed for hypertension							Fasting serum glucose level ≥7.0, non-fasting serum glucose level ≥11.1, or use of anti-diabetic medication
Desai (2020)[12]	1	>65	Levin's formula	Mod									Prevalence of living alone from UK 2001 census (≥65 years)		
Dragioti (2022)[14]	3	N/A	Levin's formula	Low								Two definitions: (1) Late-life depression by valid diagnostic measurements in older adults		Physically active older adults compared to their non-active or	Adult patients (>40 years) with type 2 diabetes

Reference	No of risks meta-analysed	Sample Age in years	PAF Formula used	Quality Rating	Low education	Hearing	TBI	Hypertension	Alcohol	Obesity	Smoking	Depression	Social Isolation	PA	Diabetes
												compared to not diseased; and (2) Depressive disorders in patients at any age by valid diagnostic measurements compared to individuals with no mental disorder		insufficient active counterparts	
Ehrlich (2022)[15]	11	≥50	Levin's formula	Mod	No formal education or less than a high school degree	SR (Fair or poor hearing)	Lifetime head or neck injury and loss of consciousness	SR (Doctor diagnosed high blood pressure or hypertension)	≥21 alcoholic drinks per week	BMI ≥30	Current	SR (Doctor diagnosed)	Not taking part in any of the following (1) co-habitation; (2) talking to ≥2 people about important things; (3) attending religious services; or (4) participation in a club, class, or organized activity	Never or hardly ever report engagement in sports or activities that are moderately energetic	SR (Doctor diagnosed)
Feter (2022)[17]	1	N/A	Levin's formula	Mod										Did not meet minimum of 150 min/week of moderate intensity activity from leisure, commuting or occupational activity (domestic/work)	
Global Burden of Disease (2021)[19]	1	N/A	Levin's formula	High			GBD 2019 age-sex-location-year-specific estimates of the prevalence of TBI								
Hazar (2016)[21]	4	N/A	Levin's formula	Low						Obesity (45-65 years)	Figure taken from prevalence as reported for Iran by the WHO			Figure taken from prevalence as reported for Iran by the WHO	Figure taken from prevalence as reported for Iran by the WHO
Hodis (2020)[23]	1	45-64	Levin's formula	High				SBP ≥130 or DBP ≥80 according to new ACC/AHA guidelines (binary variable)							
Fei-fei Hu (2022)[24]	6	≥65	Levin's formula	High	Not receiving more than primary education in early life	SR impairment						Diagnosis in later life according to DSM-IV based on a structured Geriatric Mental State interview or SR of prior depression	SR contact (telephone or face-to-face) frequency with friends, relatives, and neighbours. Classified as: basically none, occasionally, and frequently. Those who answered "basically none" were classified as socially isolated	SR degree of physical activity in later life on a 3-point Likert scale (categories were mild, moderate, and serious physical activity). Classified mildly physically active subjects into inactive	SR clinical diagnosis of diabetes and glucose lowering medication use. If SR not available, used fasting blood glucose level (≥ 7)
Jørgensen (2023)[27]	11	≥16	Levin's formula	High	Basic only (9th grade or less)	Pure tone >25 dB threshold in better ear measured at 2, and 4 kHz - not compensated by hearing aid use (50-64 years)	One or more hospital contacts resulting in TBI diagnosis (All ages)	SR in Danish National Health Survey (45-64 years)	SR consumption >14 units/week (45-64 years)	BMI ≥30 (45-64 years)	SR daily smoking (≥65 years)	SR depression (≥65 years)	SER often unwanted alone (≥65 years)	SR not adhering to WHO recommendations: 150-300 min/week of moderate activity, 75-150 min/week of vigorous activity, or an equivalent combination (≥65 years)	Type 2 and type 1 diabetes (≥65 years)

Reference	No of risks meta-analysed	Sample Age in years	PAF Formula used	Quality Rating	Low education	Hearing	TBI	Hypertension	Alcohol	Obesity	Smoking	Depression	Social Isolation	PA	Diabetes
Katzmarzyk (2022)[28]	1	N/A	Levin's formula	High										At least 150 min of moderate intensity, or 75 min of vigorous intensity PA/week or any equivalent combination including at work, home, for transport and leisure time	
Kotaki (2019)[30]	7	≥65	Levin's formula	High	Age at school graduation <16 years			History of hypertension or SBP ≥140 and/or DBP ≥90		BMI ≥30	Current smoker	Kessler 6-Item Psychological Distress Scale score ≥13		Time spent walking <30 min/day	History of diabetes mellitus
Launer (2010)[31]	1	45-68	Prevalence multiplied by the AF (where AF=RR*total number of dementia cases)	High				JNC7 guidelines of untreated mid-life levels of SBP >120							
Lee (2022)[32]	11	25-95	Levin's formula	Mod	<12 year of schooling (25-44 years)	Pure tone mean >25 dB hearing threshold in better ear measured at 0.5, 1, 2, and 4 kHz (45-64 years)	SR of lifetime head injury resulting in loss of consciousness (45-64 years)	SR antihypertensive medications or measured SBP ≥140 or measured DBP ≥90 (45-64 years)	SR drinking >14 alcoholic drinks/week (45-64 years)	BMI ≥30 (45-64 years)	SR current smoking (≥65 years)	PHQ-9 score ≥10 (≥65 years)	SR of contact with family, friends, religious organizations, organized groups, or volunteering less than once/month (49-95 years)	SR of not doing either 75 min/week of vigorous activity or 150 min/week of moderate activity or 150 min/week of moderate/ vigorous activity (≥65 years)	SR diagnosis or measured fasting plasma glucose ≥126 mg/dL or measured HbA1C ≥6.5% (≥65 years)
Liu (2020)[33]	7	≥18	Levin's formula	High	Proportion of adults with less than a senior high school education			SBP ≥140, DBP ≥90 or SR antihypertensive medications in last 2 weeks irrespective of blood pressure (50–59 years)		BMI ≥30 (40–59 years)	Proportion of current adult smokers	Centre for Epidemiologic Studies Depression Scale score ≥28 (Adults)		Do not exercise ≥3.5 hours/week (Adults)	Fasting plasma glucose level ≥126, 2-hour plasma glucose level ≥200, HbA1c concentration ≥6.5% or SR previous diagnosis by healthcare professionals
Livingston (2020)[35]	11	N/A	Levin's formula	High	Less education (<45 years)	Hearing loss (45-65 years)	TBI (45-65 years)	Hypertension (45-65 years)	>21 units per week (45-65 years)	BMI ≥30 (45-65 years)	Smoking (>65 years)	Later life (>65 years)	Later life (>65 years)	Later life (>65 years)	Later life (>65 years)
Loef (2013)[36]	1	N/A	Modified Levin's formula (to account for follow-up time)	Low						USA: BMI>30 (40-59)<br>China: BMI>25 (ML)					
Luck (2016)[37]	7	N/A	Levin's formula	Mod	Proportion of adults with low level of education according to the ISCE category 2 or lower			SBP ≥140 and/or DBP ≥90 (35-64 years)		BMI ≥30.0 (35-64 years)	SR smoke daily or occasionally	Major depression (DSM-IV-TR) in the adult population (12-month prevalence)		SR do not engage in physical activity	SR medically diagnosed with diabetes, antidiabetic medication in the last seven days or based on HbA1c or serum glucose
Ma'u (2021)[38]	11	≥45 years	Levin's formula	High	SR highest education attained is less than upper secondary (< 15 years)	Difficulty hearing at a threshold of ≥20dB	WHO definition of TBI	SBP ≥140 mmHg	Consumes >17 units of alcohol/week (1 unit = 10g pure alcohol)	BMI ≥30	Identifies as a current smoker	SR ever diagnosed with depression by a doctor	Lives alone	Did not meet WHO physical activity guidelines for exercise of >30 minutes/day for at least 5 days a week	SR ever diagnosed with diabetes by a doctor
MacDonald (2015)[39]	6	≥45 years	Levin's formula	High	Less than high school diploma			SR (Mid-life)			SR	SR (Mid-life)		SR	SR
Mayer (2018)[40]	7	18–69 years	Levin's formula	High	Did not attend any type of school or only completed elementary school or junior high school			SR diagnosis by a physician		BMI ≥30	SR if smoked at least 100 cigarettes during lifetime and are currently smoking either	Number of days in the past 2 weeks experienced a lack of		Engaged in either moderate (cleaning, gardening, walking at a fast pace, riding a bicycle) or	SR diagnosis by a physician

Reference	No of risks meta-analysed	Sample Age in years	PAF Formula used	Quality Rating	Low education	Hearing	TBI	Hypertension	Alcohol	Obesity	Smoking	Depression	Social Isolation	PA	Diabetes
											every day or even occasionally	interest or pleasure in doing usual activities, and the number of days, in the same time span, during which felt down, depressed, or hopeless		vigorous (running, aerobic exercise, any type of heavy work) physical leisure activities during the 30 days before the interview	
Mukadam (2019)[41]	9	≥65	Levin's formula	Mod	Not receiving more than primary education in early life	SR hearing impairment		SR diagnosis of hypertension		Waist circumference ≥88cm in women and >102cm in men	SR smoking in later life	Diagnosis according to DSM-IV in later life following a structured Geriatric Mental State interview or self-report of previous depression	Social contact occurring less than once/month in later life, calculated using pooled SR contact frequency with friends, relatives, and neighbours or attendance at social clubs	SR of being either not at all or not very physically active in later life on a four-point Likert scale (categories are not at all, not very, fairly, and very physically active)	SR known diagnosis of diabetes in later life
Mulligan (2023)[43]	1		Levin's formula	Critically Low				Age specific hypertension from the NCD Risk Factor Collaboration (30-44 years, 45-54 years, 55-64 years, and 65-74 years)							
Nianogo (2022)[44]	8	>18	Levin's formula	Mod	Didn't graduate high school	Deaf or have serious difficulty hearing		SR of doctor, nurse or other health professional diagnosis (excluding pregnancy; 40-64 years)		BMI ≥30 (40-64 years)	Have smoked at least 100 cigarettes in entire life and now smoke every day	Doctor, nurse, or other health professional diagnosis including depression, major depression, dysthymia, or minor depression		During the past month, other than regular job, participate in any physical activities or exercises such as running, calisthenics, golf, gardening, or walking for exercise	Doctor, nurse, or other health professional diagnosis (excluding Type I diabetes and diabetes during pregnancy)
Norton (2014)[45]	7	N/A	Levin's formula	High	Proportion of adults with an ISCE level of 2 or less (pre-primary, primary, and lower secondary education)			Adult ML prevalence (35-64 years)		Adult ML prevalence BMI >30 (35-64 years)	Proportion of adult smokers	Lifetime prevalence major depressive disorder using DSM or ICD criteria		Proportion of adults who do not do either 20 min of vigorous activity on ≥3 days or 30 min of moderate activity on ≥5 days/week	Adult prevalence diagnosed diabetes (20-79 years)
Oliveira (2019)[46]	7	N/A	Levin's formula	High	Proportion of adults with an ISCE level of 2 or less (pre-primary, primary, and lower secondary education)			Adult ML prevalence (35-64 years)		Adult ML prevalence BMI >30 (35-64 years)	Proportion of adult smokers	Lifetime prevalence major depressive disorder using DSM or ICD criteria		Proportion of adults who do not do either 20 min of vigorous activity on ≥3 days or 30 min of moderate activity on ≥5 days/week	Adult prevalence diagnosed diabetes (20-79 years)
Ren (2022)[47]	2	35-73	Levin's formula	High	College degree recipient vs. nonrecipient (40-69 years)						Current (40-69 years)				
Rolandi (2020)[49]	10	70-74	"punafcc" Stata	High	≤5 years formal education	Evaluated by physician with Whispered Voice Test	Anamnestic diagnosis	SBP >130 or DBP >80 or antihypertensive use		BMI ≥30	Current smoking	DSM-IV-TR major depression or dysthymia OR at least 3 of (i) history of depression, (ii) depression treatment, (iii) GDS ≥8, and (iv) SR depressed mood during the last week	Feeling lonely during the last week	Absence of any weekly vigorous or moderate leisure time physical activity	Fasting glucose ≥126 mg/dL OR diabetes treatment
Santabarbara (2019a)[51]	1	≥50	Levin's formula	Mod								GMS AGE-CAT to establish clinical cases (later life)			

Reference	No of risks meta-analysed	Sample Age in years	PAF Formula used	Quality Rating	Low education	Hearing	TBI	Hypertension	Alcohol	Obesity	Smoking	Depression	Social Isolation	PA	Diabetes
Santabarbara (2020b)[54]	1	N/A	Levin's formula	Low								Diagnosis by NPI-Q, GDS-15, ICD-9 (major depression), ICD-10 (or antidepressant), CES-D or GHQ-30			
Shang (2022)[57]	7	38-73	N/A	Mod		Hearing aid use or difficult to follow a conversation. ICD9/10 codes H90, H91 or 389 (40-69 years)	Head injury or neurological injury/trauma. ICD9/10 codes S00, S01, S02, S03, S04, S05, S06, S07, S08, S09, S020, S021, S028, S0291, S0402, S0403, S0404, S06, S071, T744, 95901, 800, 801, 802, 803 or 804 (40-69 years)	Hypertension or essential hypertension (40-69 years)	Alcohol dependency or alcoholic liver disease/alcoholic cirrhosis. ICD9/10 codes F102, K70, F101, 3039, 291 or 303 (40-69 years)	BMI ≥30 (40-69 years)		Depression or postnatal depression. ICD9/10 codes F32, F33, F341, F381, F204, 2962, 2963, 3004 or 311 (40-69 years)			Diabetic nephropathy, diabetic neuropathy/ulcers, diabetes, Type 1, Type 2, or diabetic eye disease. ICD9/10 codes G632, G590, H360, H280, E10, E11, E12, E13, E14, E00, 240 or 250 (40-69 years)
Smith (2023)[59]	1	≥65	Levin's formula	High		Any hearing loss including mild hearing loss (26–40 dB HL) and moderate or greater hearing loss (≥41 dB HL) (65+ years)									
Suemoto (2023)[61]	11	≥50	Levin's formula	Mod	≤8 years of formal education (25 years)	SR of bad or very bad hearing, or current use of hearing aids (≥50 years)	Reported prevalence based on previous meta-analysis	Previous diagnoses by health-care professionals or current antihypertensive drug use (≥50 years)	>21 units per week (1 unit = 10 grams) (≥50 years)	BMI ≥30 (≥50 years)	Current tobacco use (≥50 years)	Previous diagnosis by health-care professional (≥50 years)	Seeing family members or friends less than once/month (≥50 years)	Vigorous activities <75 min/week, moderate activities, or walking <150 min/week, or an equivalent combination of moderate and vigorous activities according to WHO (≥50 years)	Previous diagnoses by health-care professionals or current use of insulin or hypoglycaemic drugs (≥50 years)
Thompson (2022)[63]	11	>40	Levin's formula	Mod	≤7 years or only some primary education (<45 years)	Failing either the left or right ear Whisper Test, hearing impairment from medical examination or medical records, defined as hearing aids, hearing loss/reduction/impairment, or deafness, or ICD9/10 codes H90, H91 or 389 (45-65 years)	Hit on head and knocked out (≥30 min) or medical record (45-65 years)	SR, identified from medical examination, medical records, hypertension from blood pressure (i.e., BBP ≥140 mmHg or DBP ≥90 mmHg), or ICD9/10 codes I10, I11, I110, I119, I12, I120, I129, I13, I130, I131, I132, I139, I15, I150, I151, I152, I158, I159, 401, 402, 403, 404 or 405 (45-65 years)	Drinking until drunk every day or 1-3, or 4-6 times/week, or drinking 4-6 drinks, 4-6 times/week OR drinking ≥61grams ≥3 times/week (45-65 years)	BMI ≥30 or obesity noted in medical history (45-65 years)	Smoking every day (>65 years)	PHQ-9 ≥10 (>65 years)	No other people living in the household i.e., living alone (>65 years)	Australia 2014 Physical Activity and Sedentary Behaviour Guidelines of ≥150 minutes of PA in the previous week, where 'hard' activity counts for double minutes (>65 years)	Self-reported, HbA1c ≥6.5%, identified from medical examination, or review of medical record (>65 years)
Vagelatos (2013)[66]	1		Levin's formula	Low											Varying definitions across studies included in the meta-analysis (and different sample ages)
Vergara (2022)[67]	9	≥45 NOTE: Analysis stratified by mid (45-64) vs later	Levin's formula	Mod	Not receiving more than primary education in early life (<7 years of scholarship)	SR negative response to any of the following: (1) Do you think you hear normally by both ears?; (2) Are you able to follow a TV program at a volume acceptable		SBP >140, a diagnosis of hypertension or treatment	>21 units/week	BMI ≥30	SR currently smoke cigarettes	Suspected major depression in the last 12 months (DSM-IV), assessed with the CIDI-SF		Low according to the Global Physical Activity Questionnaire version 2	SR and/or medical treatment for diabetes, or fasting blood glucose ≥126 mg/dL (≥7.0 mmol/L)

Reference	No of risks meta-analysed	Sample Age in years	PAF Formula used	Quality Rating	Low education	Hearing	TBI	Hypertension	Alcohol	Obesity	Smoking	Depression	Social Isolation	PA	Diabetes
		life (≥65)				to others?; and, (3) Are you able to follow a conversation of ≥3 people?									
Woo (2014)[70]	7	>18	Levin's formula	Low	Census & Statistics Department (≥15 years)			Department of Health (Midlife; >15 years)		Department of Health (Midlife; 18-64 years)	Census & Statistics Department (≥15 years)	Department of Health (Later life >60 years)		Department of Health (18-64 years)	Department of Health (20-79 years)
Wu (2022)[71]	8	≥60	Levin's formula	High	Not receiving more than primary education in early life	SR hearing impairment		Diagnosed by doctor		BMI ≥30	SR smoking in later life (Excludes smoking but has quit)		SR participation in social activities (community patrols, caring for other people, environmental protection, dispute mediation, accompanying chat, volunteer service, childcare, etc.) less than once	SR number of conscious physical exercises (morning exercises, break exercises, physical education classes, extracurricular sports classes, work exercises, square dancing, walking exercises, walks, running) per week is less than once	Diagnosed by doctor
Xu (2015)[72]	6	N/A	Levin's formula	Mod	Less than primary school)			SBP≥160 mmHg		BMI ≥30 (Midlife)	Current smoking in Asian population (Yes vs no)	Depression (Yes vs no)			Diabetes in Asian population (Yes vs no)
Zhang (2021)[73]	7	≥65 years	Levin's formula	High	Primary school or lower			SR clinical diagnosis of high blood pressure		BMI ≥24	SR history of smoking		Lack of good social relationships (social activities)	SR absence of physical exercise habit or exercise frequency < 1 time/week	SR clinical diagnosis

Key

ACC=American College of Cardiology; AF=Attributable fraction; AHA=American Heart Association; BMI=Body Mass Index; CES-D=Centre for Epidemiologic Studies Depression Scale; CIDI-SF =Composite International Diagnostic Interview Short Form; DBP=Diastolic Blood Pressure; DSM=Diagnostic and Statistical Manual of Mental Disorders; GDS=Geriatric Depression Scale; GHQ-30= General Health Questionnaire; HbA1C=Glycated haemoglobin; ICD= International Classification of Diseases; ISCE = International Standard Classification of Education; JNC7=Seventh Report of the Joint National Committee on Prevention, Detection, Evaluation, and Treatment of High Blood Pressure; ML=Midlife; Mod=Moderate quality; N/A=Not available; NPI-Q=Neuropsychiatric Inventory–Questionnaire; PHQ-9= 9-question Patient Health Questionnaire; RR=Relative risk; SBP = Systolic Blood Pressure; SR = Self-reported; TBI=Traumatic Brain Injury; WHO=World Health Organisation

Note

For Levin's formula see Appendix 3, page 6 (above)

Appendix 10 Forest plots summarising the unweighted population attributable fractions (PAF) for each individual risk factor stratified by country income level

Acronyms HIC=High income countries; LMIC=Low- and middle-income countries

Key:

#Indigenous Population Canada (MacDonald et al[39])

^Non-Indigenous Population Canada (MacDonald et al[39])

*Indigenous Population Australia (Thompson et al[63])

~Untreated Hypertension (Launer et al[31])

@Current smoking (Ren at al[47])

&Men (Ren et al[47], education)

$Women (Ren et al[47], education)

Education

Forest Plot

- Effect size of each study
- Estimated overall effect size
- No-effect value
- Confidence interval of effect size
- Overall effect size value
- Estimated overall confidence interval

Global_Status	ID	Effect Size	Lower	Upper	p-value
Global	Barnes (2011)	7.30	4.35	10.25	0.00
	Xu (2015)	14.10	7.44	20.76	0.00
	Barnes (2011)	19.10	12.45	25.75	0.00
	Norton (2014)	19.10	12.45	25.75	0.00
	Livingstone (2020)	19.40	10.47	28.33	0.00
	Beydoun (2014)	24.00	8.40	39.60	0.00
	Subgroup Overall	15.80	9.09	22.51	0.00
HIC	Kotaki (2018)	4.40	-0.47	9.27	0.08
	Jorgensen (2023)	5.40	2.20	8.60	0.00
	Lee (2022)	6.00	2.45	9.55	0.00
	Ehrlich (2022)	6.50	2.98	10.02	0.00
	Norton (2014)	7.30	4.35	10.25	0.00
	Luck (2016)	7.50	4.55	10.45	0.00
	Nianogo (2022)	11.70	11.45	11.95	0.00
	Woo (2014)	11.80	7.35	16.25	0.00
	MacDonald (2015)^	12.00	7.45	16.55	0.00
	Norton (2014)	12.20	7.55	16.85	0.00
	Mayer (2018)	13.60	8.55	18.65	0.00
	Norton (2014)	13.60	8.55	18.65	0.00
	Thompson (2022)*	13.83	6.36	21.30	0.00
	Ashby-Mitchell (2017)	14.70	10.90	18.50	0.00
	Ma'u (2021)	15.70	8.12	23.28	0.00
	Ren (2022)&	17.26	8.15	26.37	0.00
	Rolandi (2020)	18.50	7.45	29.55	0.00
	Ren (2022)$	19.22	8.62	29.82	0.00
	Oliviera (2019)	21.50	14.25	28.75	0.00
	MacDonald (2015)#	22.40	14.90	29.90	0.00
	Mayer (2018)	31.80	24.05	39.55	0.00
	Subgroup Overall	12.85	9.90	15.80	0.00
LMIC	Vergara (2022)	9.10	4.15	14.05	0.00
	Ashby-Mitchell (2018)	10.90	7.95	13.85	0.00
	Liu (2020)	11.90	-0.31	24.11	0.06
	Oliviera (2019)	20.90	13.75	28.05	0.00
	Oliviera (2019)	21.00	13.85	28.15	0.00
	Ashby-Mitchell (2020)	21.40	16.30	26.50	0.00
	Suemoto (2023)	21.90	21.05	22.75	0.00
	Zhang (2021)	22.87	12.79	32.95	0.00
	Vergara (2022)	23.00	12.55	33.45	0.00
	Bobrow (2021)	28.00	16.50	39.50	0.00
	Mukadam (2019)	29.20	28.40	30.00	0.00
	Mukadam (2019)	31.30	29.35	33.25	0.00
	Mukadam (2019)	35.60	33.50	37.70	0.00
	Hu (2022)	40.40	40.05	40.75	0.00
	Subgroup Overall	23.75	18.22	29.29	0.00
Overall		17.29	14.40	20.18	0.00

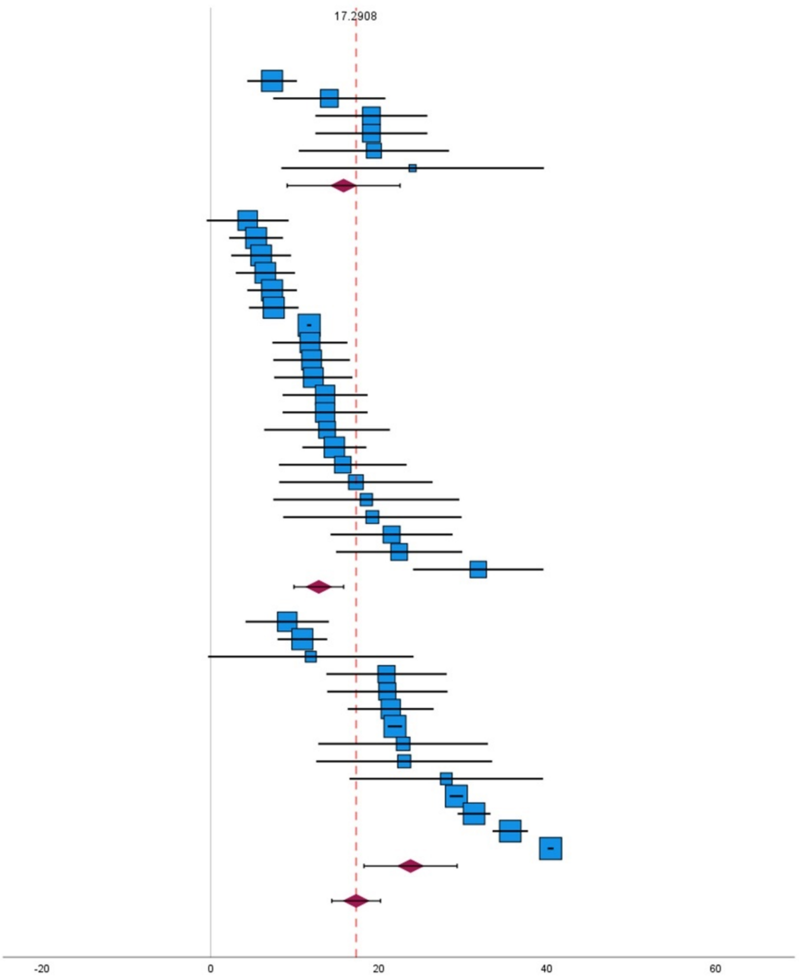

Model: Random-effects model
Heterogeneity: Tau-squared = 73.38, H-squared = 125.92, I-squared = 0.99
Homogeneity: Q = 18463.74, df = 40, p-value = 0.00
Test of overall effect size: t = 12.08, df = 40, p-value = 0.00
Test of between-subgroup homogeneity: Q = 14.08, df = 2, p-value = 0.00

Hearing Loss

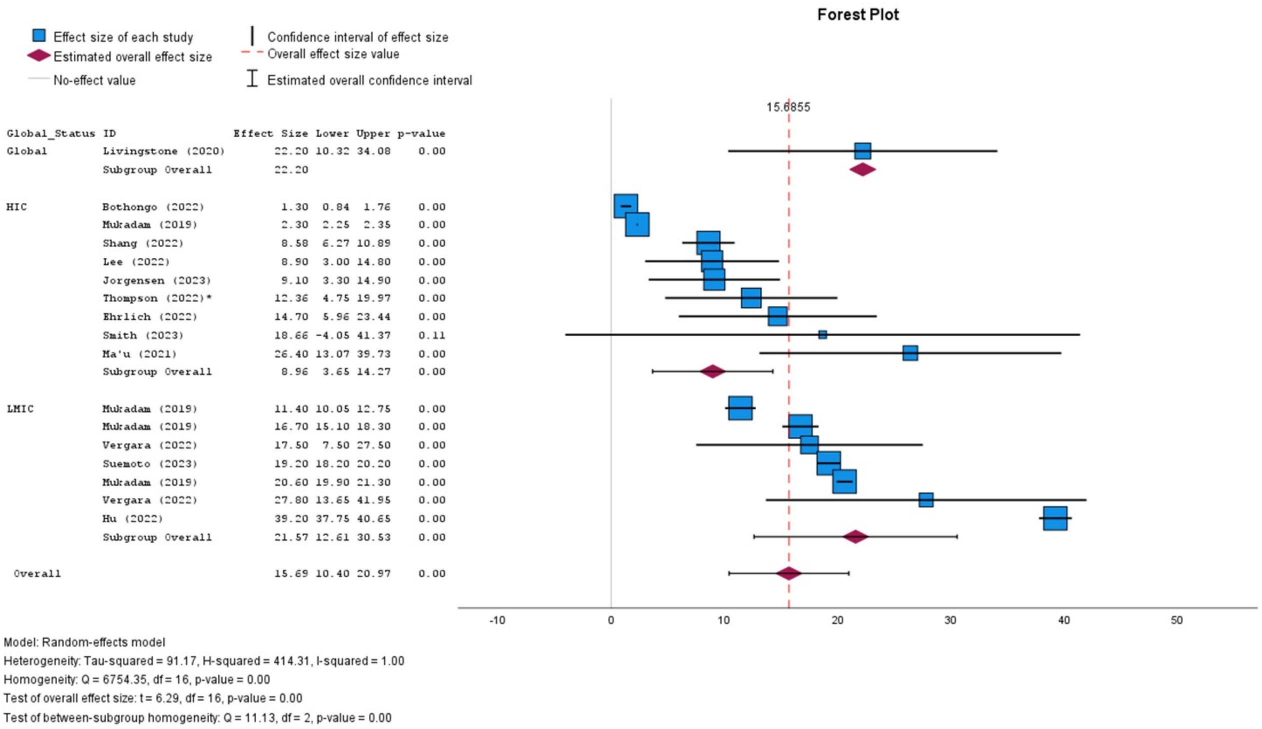

Traumatic Brain Injury

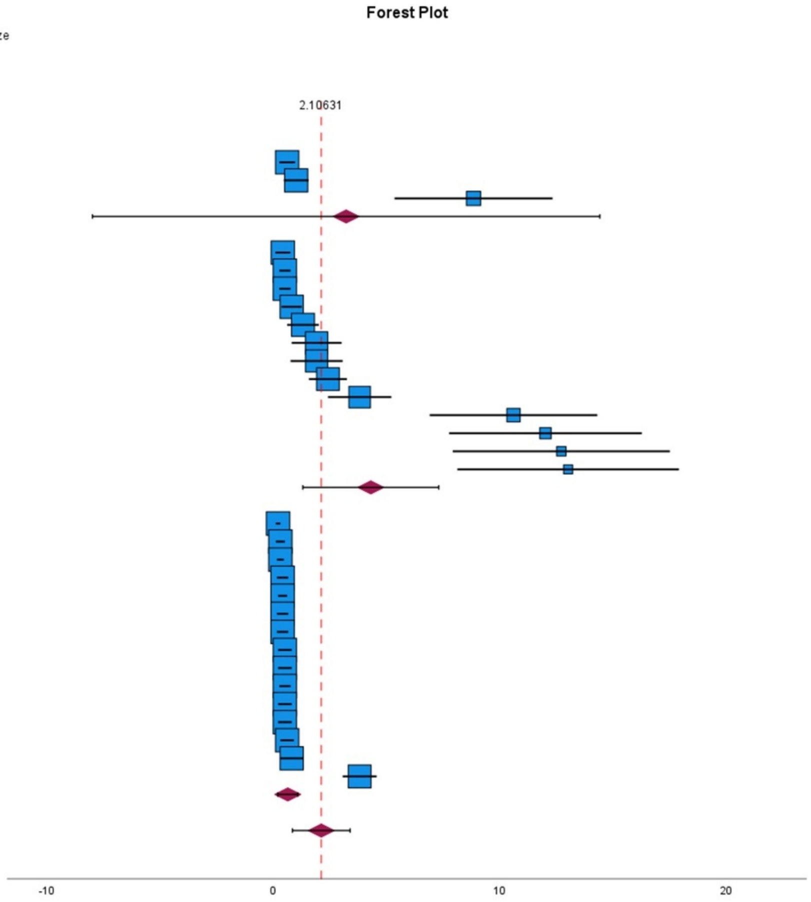

Global_Status	ID	Effect Size	Lower	Upper	p-value
Global	GBD (2021)	0.60	0.25	0.95	0.00
	GBD (2021)	1.00	0.45	1.55	0.00
	Livingstone (2020)	8.83	5.34	12.31	0.00
	Subgroup Overall	3.20	-7.99	14.40	0.34
HIC	Shang (2022)	0.41	0.08	0.74	0.01
	GBD (2021)	0.50	0.25	0.75	0.00
	GBD (2021)	0.50	0.25	0.75	0.00
	GBD (2021)	0.80	0.35	1.25	0.00
	GBD (2021)	1.30	0.60	2.00	0.00
	GBD (2021)	1.90	0.80	3.00	0.00
	GBD (2021)	1.90	0.75	3.05	0.00
	Bothongo (2022)	2.40	1.56	3.24	0.00
	Jorgensen (2023)	3.80	2.40	5.20	0.00
	Thompson (2022)*	10.59	6.89	14.29	0.00
	Lee (2022)	12.00	7.75	16.25	0.00
	Ma'u (2021)	12.70	7.91	17.49	0.00
	Ehrlich (2022)	13.00	8.11	17.89	0.00
	Subgroup Overall	4.29	1.29	7.29	0.01
LMIC	GBD (2021)	0.20	0.10	0.30	0.00
	GBD (2021)	0.30	0.10	0.50	0.00
	GBD (2021)	0.30	0.15	0.45	0.00
	GBD (2021)	0.40	0.15	0.65	0.00
	GBD (2021)	0.40	0.20	0.60	0.00
	GBD (2021)	0.40	0.15	0.65	0.00
	GBD (2021)	0.40	0.15	0.65	0.00
	GBD (2021)	0.50	0.20	0.80	0.00
	GBD (2021)	0.50	0.20	0.80	0.00
	GBD (2021)	0.50	0.25	0.75	0.00
	GBD (2021)	0.50	0.20	0.80	0.00
	GBD (2021)	0.50	0.20	0.80	0.00
	GBD (2021)	0.60	0.30	0.90	0.00
	GBD (2021)	0.80	0.30	1.30	0.00
	Suemoto (2023)	3.80	3.05	4.55	0.00
	Subgroup Overall	0.63	0.19	1.07	0.01
Overall		2.11	0.83	3.38	0.00

Model: Random-effects model
Heterogeneity: Tau-squared = 8.35, H-squared = 367.42, I-squared = 1.00
Homogeneity: Q = 308.22, df = 30, p-value = 0.00
Test of overall effect size: t = 3.38, df = 30, p-value = 0.00
Test of between-subgroup homogeneity: Q = 8.54, df = 2, p-value = 0.01

Hypertension

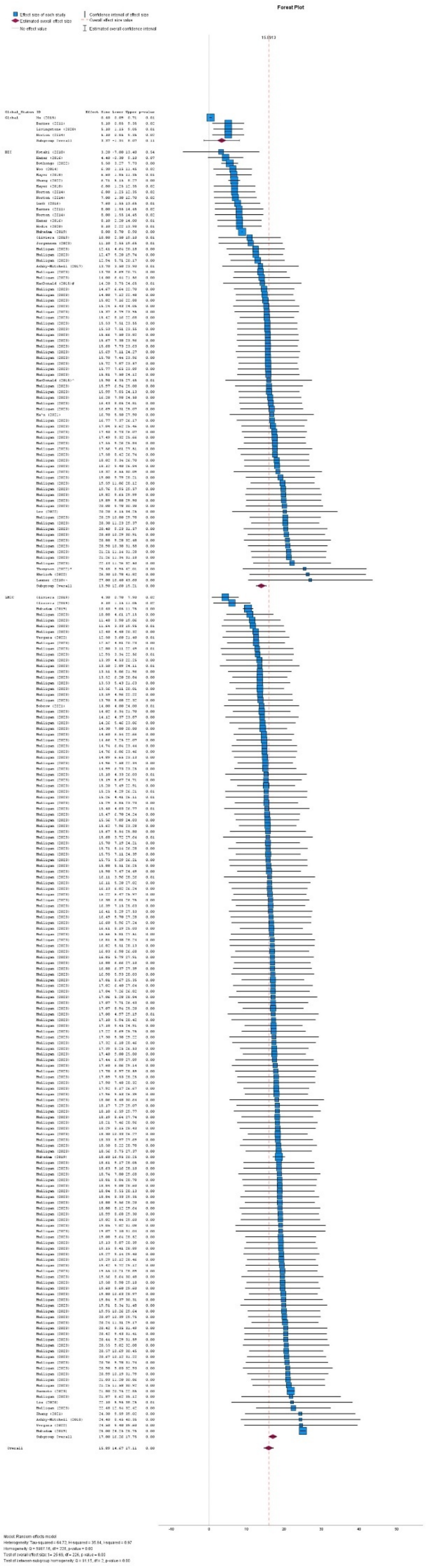

Alcohol Consumption

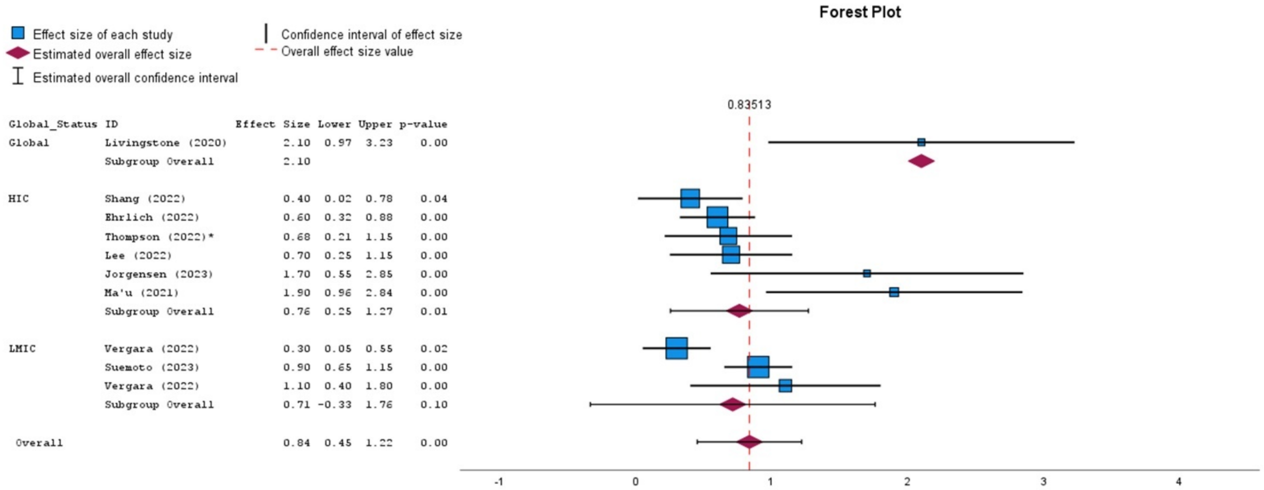

Model: Random-effects model
Heterogeneity: Tau-squared = 0.14, H-squared = 4.30, I-squared = 0.77
Homogeneity: Q = 31.06, df = 9, p-value = 0.00
Test of overall effect size: t = 4.92, df = 9, p-value = 0.00
Test of between-subgroup homogeneity: Q = 5.29, df = 2, p-value = 0.07

Obesity

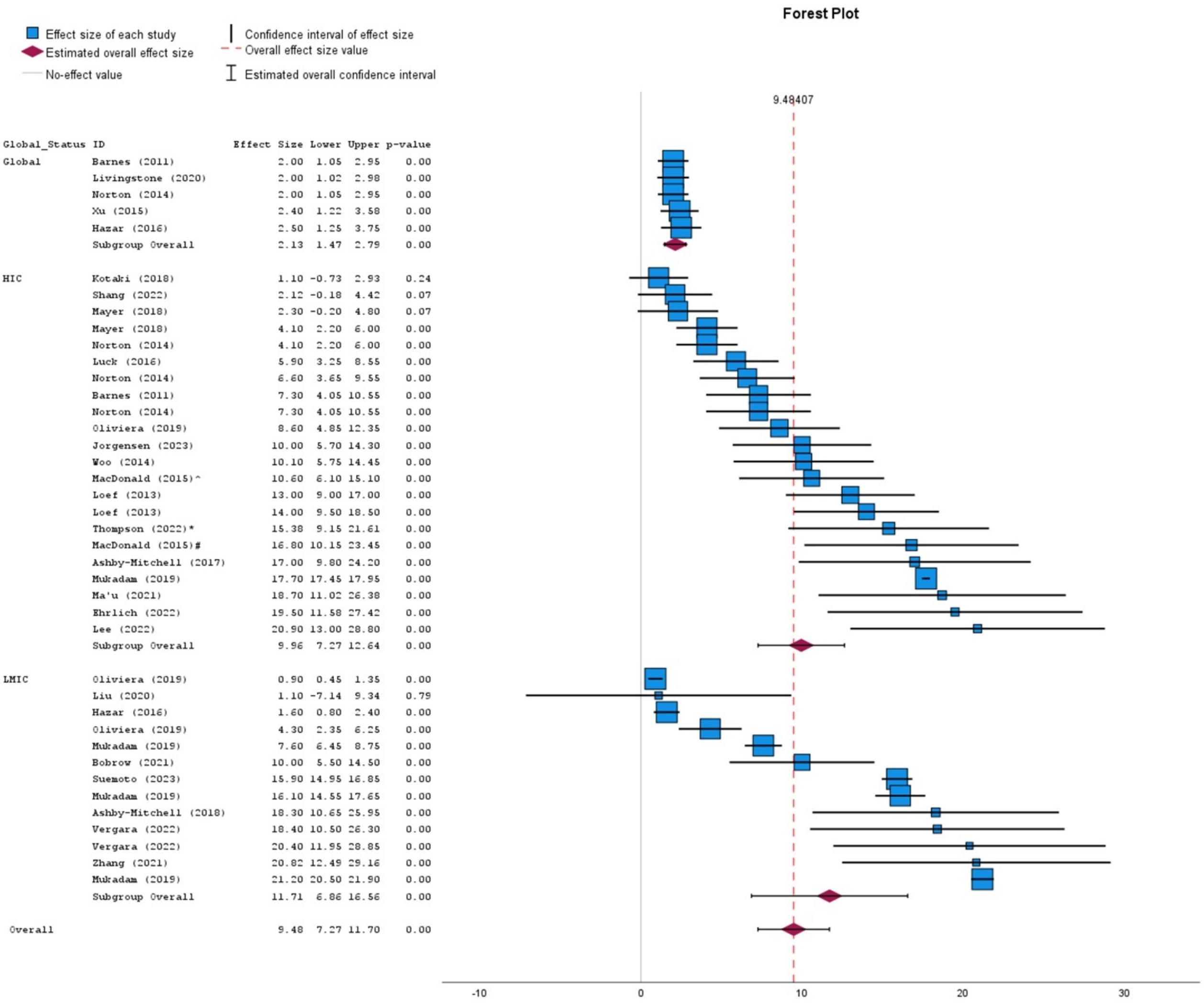

Global_Status	ID	Effect Size	Lower	Upper	p-value
Global	Barnes (2011)	2.00	1.05	2.95	0.00
	Livingstone (2020)	2.00	1.02	2.98	0.00
	Norton (2014)	2.00	1.05	2.95	0.00
	Xu (2015)	2.40	1.22	3.58	0.00
	Hazar (2016)	2.50	1.25	3.75	0.00
	Subgroup Overall	2.13	1.47	2.79	0.00
HIC	Kotaki (2018)	1.10	-0.73	2.93	0.24
	Shang (2022)	2.12	-0.18	4.42	0.07
	Mayer (2018)	2.30	-0.20	4.80	0.07
	Mayer (2018)	4.10	2.20	6.00	0.00
	Norton (2014)	4.10	2.20	6.00	0.00
	Luck (2016)	5.90	3.25	8.55	0.00
	Norton (2014)	6.60	3.65	9.55	0.00
	Barnes (2011)	7.30	4.05	10.55	0.00
	Norton (2014)	7.30	4.05	10.55	0.00
	Oliviera (2019)	8.60	4.85	12.35	0.00
	Jorgensen (2023)	10.00	5.70	14.30	0.00
	Woo (2014)	10.10	5.75	14.45	0.00
	MacDonald (2015)^	10.60	6.10	15.10	0.00
	Loef (2013)	13.00	9.00	17.00	0.00
	Loef (2013)	14.00	9.50	18.50	0.00
	Thompson (2022)*	15.38	9.15	21.61	0.00
	MacDonald (2015)#	16.80	10.15	23.45	0.00
	Ashby-Mitchell (2017)	17.00	9.80	24.20	0.00
	Mukadam (2019)	17.70	17.45	17.95	0.00
	Ma'u (2021)	18.70	11.02	26.38	0.00
	Ehrlich (2022)	19.50	11.58	27.42	0.00
	Lee (2022)	20.90	13.00	28.80	0.00
	Subgroup Overall	9.96	7.27	12.64	0.00
LMIC	Oliviera (2019)	0.90	0.45	1.35	0.00
	Liu (2020)	1.10	-7.14	9.34	0.79
	Hazar (2016)	1.60	0.80	2.40	0.00
	Oliviera (2019)	4.30	2.35	6.25	0.00
	Mukadam (2019)	7.60	6.45	8.75	0.00
	Bobrow (2021)	10.00	5.50	14.50	0.00
	Suemoto (2023)	15.90	14.95	16.85	0.00
	Mukadam (2019)	16.10	14.55	17.65	0.00
	Ashby-Mitchell (2018)	18.30	10.65	25.95	0.00
	Vergara (2022)	18.40	10.50	26.30	0.00
	Vergara (2022)	20.40	11.95	28.85	0.00
	Zhang (2021)	20.82	12.49	29.16	0.00
	Mukadam (2019)	21.20	20.50	21.90	0.00
	Subgroup Overall	11.71	6.86	16.56	0.00
Overall		9.48	7.27	11.70	0.00

Model: Random-effects model
Heterogeneity: Tau-squared = 42.45, H-squared = 106.63, I-squared = 0.99
Homogeneity: Q = 8030.76, df = 39, p-value = 0.00
Test of overall effect size: t = 8.65, df = 39, p-value = 0.00
Test of between-subgroup homogeneity: Q = 53.65, df = 2, p-value = 0.00

Smoking

Effect size of each study
Estimated overall effect size
No-effect value
Confidence interval of effect size
Overall effect size value
Estimated overall confidence interval

Global_Status	ID	Effect Size	Lower	Upper	p-value
Global	Xu (2015)	1.00	0.04	1.96	0.04
	Barnes (2011)	13.90	3.50	24.30	0.01
	Norton (2014)	13.90	3.50	24.30	0.01
	Livingstone (2020)	14.10	4.33	23.87	0.00
	Hazar (2016)	15.70	8.40	23.00	0.00
	Xu (2015)	24.50	5.88	43.12	0.01
	Beydoun (2014)	31.00	17.80	44.20	0.00
	Subgroup Overall	14.67	5.50	23.84	0.01
HIC	Bothongo (2022)	0.10	-0.92	1.12	0.85
	Ren (2022)@	1.51	-0.87	3.89	0.21
	Ashby-Mitchell (2017)	4.30	-0.20	8.80	0.06
	Lee (2022)	4.90	0.90	8.90	0.02
	Kotaki (2018)	5.80	1.51	10.09	0.01
	Mukadam (2019)	6.00	5.90	6.10	0.00
	Mayer (2018)	6.50	1.30	11.70	0.01
	Ehrlich (2022)	6.70	1.58	11.82	0.01
	Ma'u (2021)	7.50	1.84	13.16	0.01
	Jorgensen (2023)	7.60	1.45	13.75	0.02
	MacDonald (2015)^	9.90	2.15	17.65	0.01
	Thompson (2022)*	9.99	2.26	17.72	0.01
	Norton (2014)	10.60	2.35	18.85	0.01
	Oliviera (2019)	10.60	2.35	18.85	0.01
	Barnes (2011)	10.80	2.40	19.20	0.01
	Norton (2014)	10.80	2.40	19.20	0.01
	Mayer (2018)	13.60	3.40	23.80	0.01
	Norton (2014)	13.60	3.40	23.80	0.01
	Luck (2016)	14.90	3.90	25.90	0.01
	MacDonald (2015)#	19.40	5.85	32.95	0.01
	Subgroup Overall	6.88	4.81	8.95	0.00
LMIC	Liu (2020)	2.40	-11.51	16.31	0.74
	Ashby-Mitchell (2018)	2.50	-0.05	5.05	0.06
	Ashby-Mitchell (2020)	3.90	-0.15	7.95	0.06
	Hazar (2016)	5.50	2.65	8.35	0.00
	Suemoto (2023)	6.00	5.20	6.80	0.00
	Woo (2014)	6.10	1.00	11.20	0.02
	Vergara (2022)	7.30	1.65	12.95	0.01
	Oliviera (2019)	8.10	1.55	14.65	0.02
	Bobrow (2021)	11.00	-8.50	30.50	0.27
	Oliviera (2019)	11.20	2.55	19.85	0.01
	Zhang (2021)	14.65	4.58	24.72	0.00
	Mukadam (2019)	14.70	13.20	16.20	0.00
	Vergara (2022)	15.50	4.90	26.10	0.00
	Mukadam (2019)	17.80	17.15	18.45	0.00
	Mukadam (2019)	19.30	17.55	21.05	0.00
	Subgroup Overall	9.78	6.28	13.28	0.00
Overall		9.16	7.25	11.07	0.00

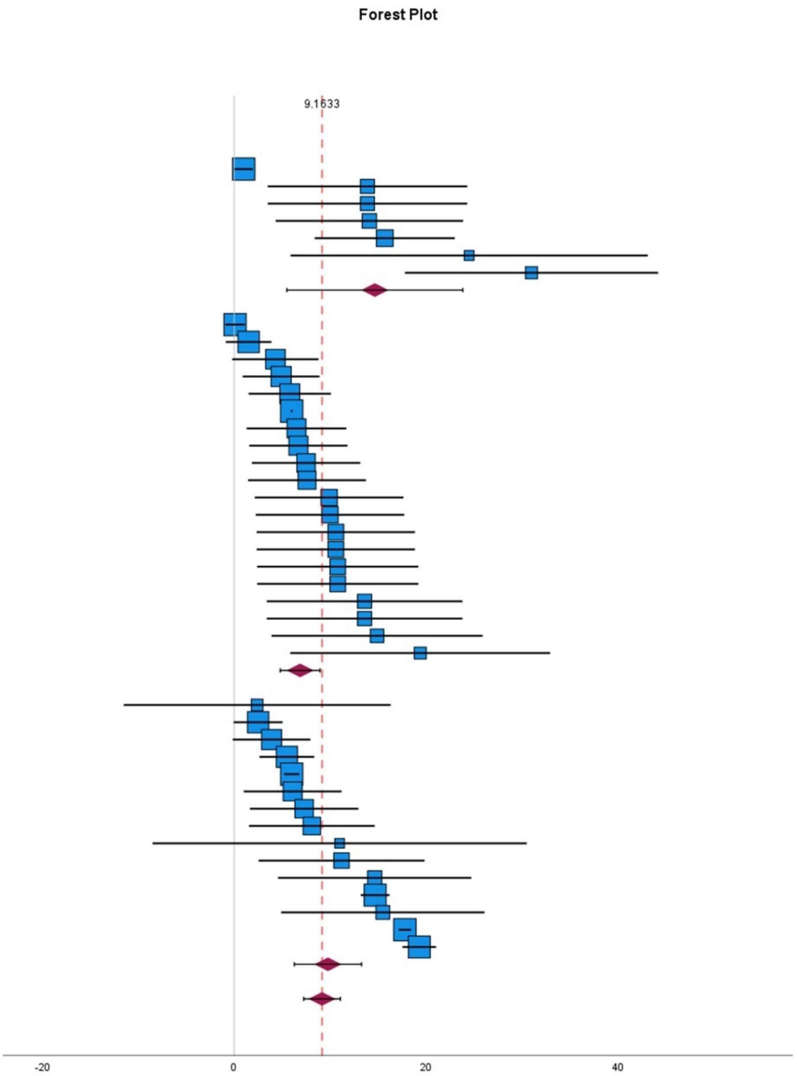

Model: Random-effects model
Heterogeneity: Tau-squared = 25.53, H-squared = 36.97, I-squared = 0.97
Homogeneity: Q = 1905.79, df = 41, p-value = 0.00
Test of overall effect size: t = 9.68, df = 41, p-value = 0.00
Test of between-subgroup homogeneity: Q = 5.63, df = 2, p-value = 0.06

Depression Lifetime

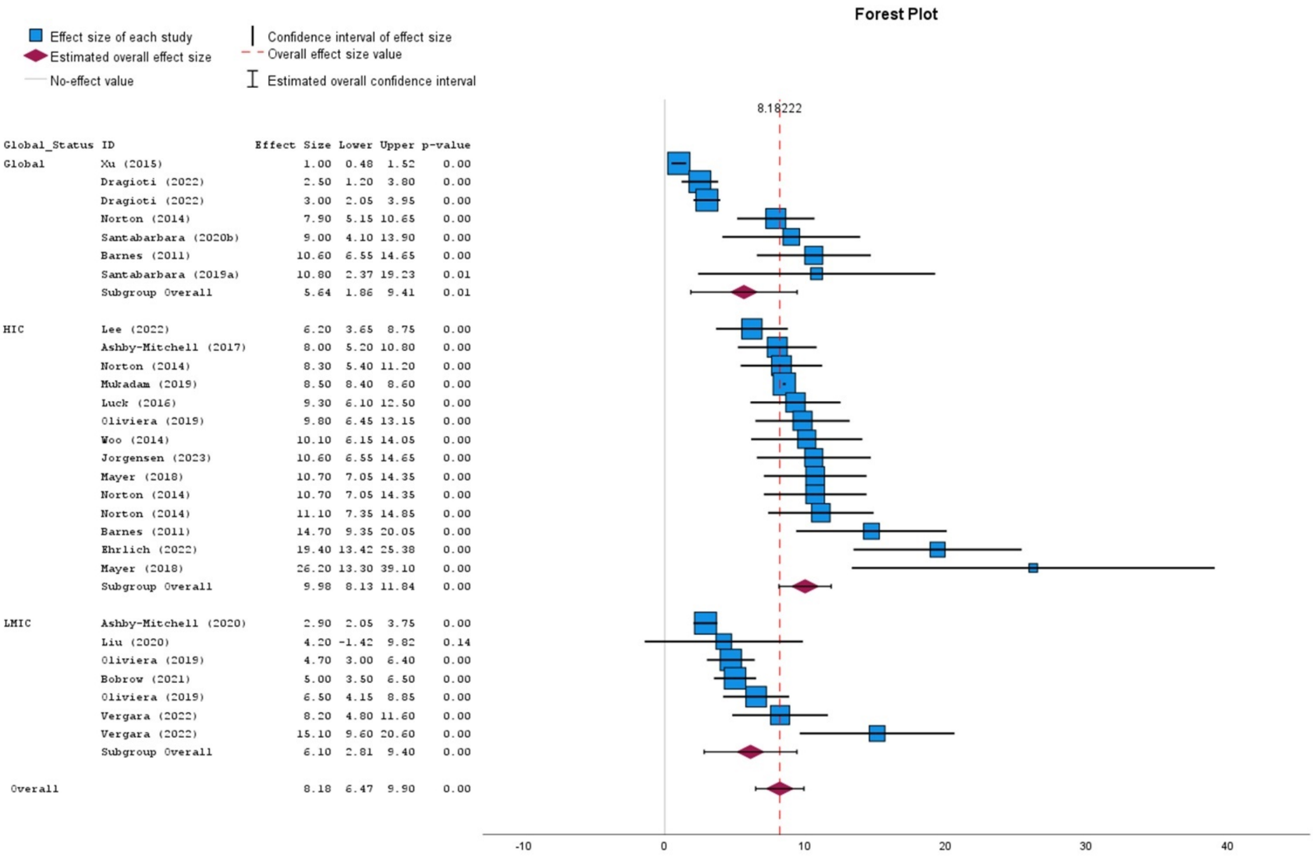

Global_Status	ID	Effect Size	Lower	Upper	p-value
Global	Xu (2015)	1.00	0.48	1.52	0.00
	Dragioti (2022)	2.50	1.20	3.80	0.00
	Dragioti (2022)	3.00	2.05	3.95	0.00
	Norton (2014)	7.90	5.15	10.65	0.00
	Santabarbara (2020b)	9.00	4.10	13.90	0.00
	Barnes (2011)	10.60	6.55	14.65	0.00
	Santabarbara (2019a)	10.80	2.37	19.23	0.01
	Subgroup Overall	5.64	1.86	9.41	0.01
HIC	Lee (2022)	6.20	3.65	8.75	0.00
	Ashby-Mitchell (2017)	8.00	5.20	10.80	0.00
	Norton (2014)	8.30	5.40	11.20	0.00
	Mukadam (2019)	8.50	8.40	8.60	0.00
	Luck (2016)	9.30	6.10	12.50	0.00
	Oliviera (2019)	9.80	6.45	13.15	0.00
	Woo (2014)	10.10	6.15	14.05	0.00
	Jorgensen (2023)	10.60	6.55	14.65	0.00
	Mayer (2018)	10.70	7.05	14.35	0.00
	Norton (2014)	10.70	7.05	14.35	0.00
	Norton (2014)	11.10	7.35	14.85	0.00
	Barnes (2011)	14.70	9.35	20.05	0.00
	Ehrlich (2022)	19.40	13.42	25.38	0.00
	Mayer (2018)	26.20	13.30	39.10	0.00
	Subgroup Overall	9.98	8.13	11.84	0.00
LMIC	Ashby-Mitchell (2020)	2.90	2.05	3.75	0.00
	Liu (2020)	4.20	-1.42	9.82	0.14
	Oliviera (2019)	4.70	3.00	6.40	0.00
	Bobrow (2021)	5.00	3.50	6.50	0.00
	Oliviera (2019)	6.50	4.15	8.85	0.00
	Vergara (2022)	8.20	4.80	11.60	0.00
	Vergara (2022)	15.10	9.60	20.60	0.00
	Subgroup Overall	6.10	2.81	9.40	0.00
Overall		8.18	6.47	9.90	0.00

Model: Random-effects model
Heterogeneity: Tau-squared = 13.70, H-squared = 35.03, I-squared = 0.97
Homogeneity: Q = 1194.43, df = 27, p-value = 0.00
Test of overall effect size: t = 9.79, df = 27, p-value = 0.00
Test of between-subgroup homogeneity: Q = 11.89, df = 2, p-value = 0.00

Depression Late Life

Effect size of each study
Estimated overall effect size
Estimated overall confidence interval
Confidence interval of effect size
Overall effect size value

Global_Status	ID	Effect Size	Lower	Upper	p-value
Global	Dragioti (2022)	3.40	2.00	4.80	0.00
	Dragioti (2022)	4.30	3.10	5.50	0.00
	Livingstone (2020)	10.60	6.95	14.25	0.00
	Subgroup Overall	5.78	-3.57	15.13	0.12
HIC	Shang (2022)	4.48	3.58	5.38	0.00
	Thompson (2022)*	4.63	2.72	6.54	0.00
	Kotaki (2018)	5.80	3.19	8.41	0.00
	Bothongo (2022)	6.80	5.58	8.02	0.00
	Ma'u (2021)	14.70	9.90	19.50	0.00
	Subgroup Overall	6.75	2.07	11.43	0.02
LMIC	Mukadam (2019)	1.30	0.80	1.80	0.00
	Hu (2022)	3.00	2.95	3.05	0.00
	Mukadam (2019)	4.50	3.60	5.40	0.00
	Suemoto (2023)	12.40	11.30	13.50	0.00
	Mukadam (2019)	17.70	17.05	18.35	0.00
	Subgroup Overall	7.77	-0.91	16.46	0.07
Overall		7.07	4.04	10.09	0.00

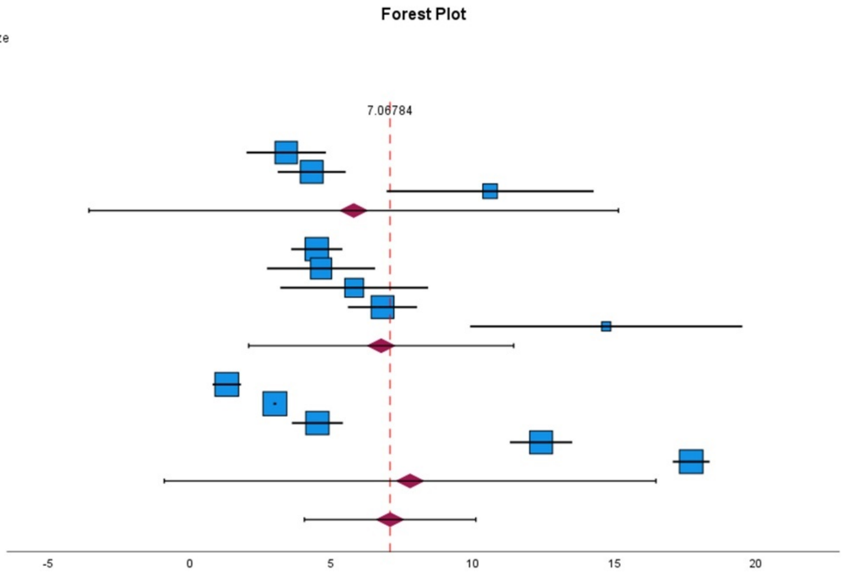

Model: Random-effects model
Heterogeneity: Tau-squared = 24.01, H-squared = 183.28, I-squared = 0.99
Homogeneity: Q = 2380.48, df = 12, p-value = 0.00
Test of overall effect size: t = 5.09, df = 12, p-value = 0.00
Test of between-subgroup homogeneity: Q = 0.30, df = 2, p-value = 0.86

Social Isolation

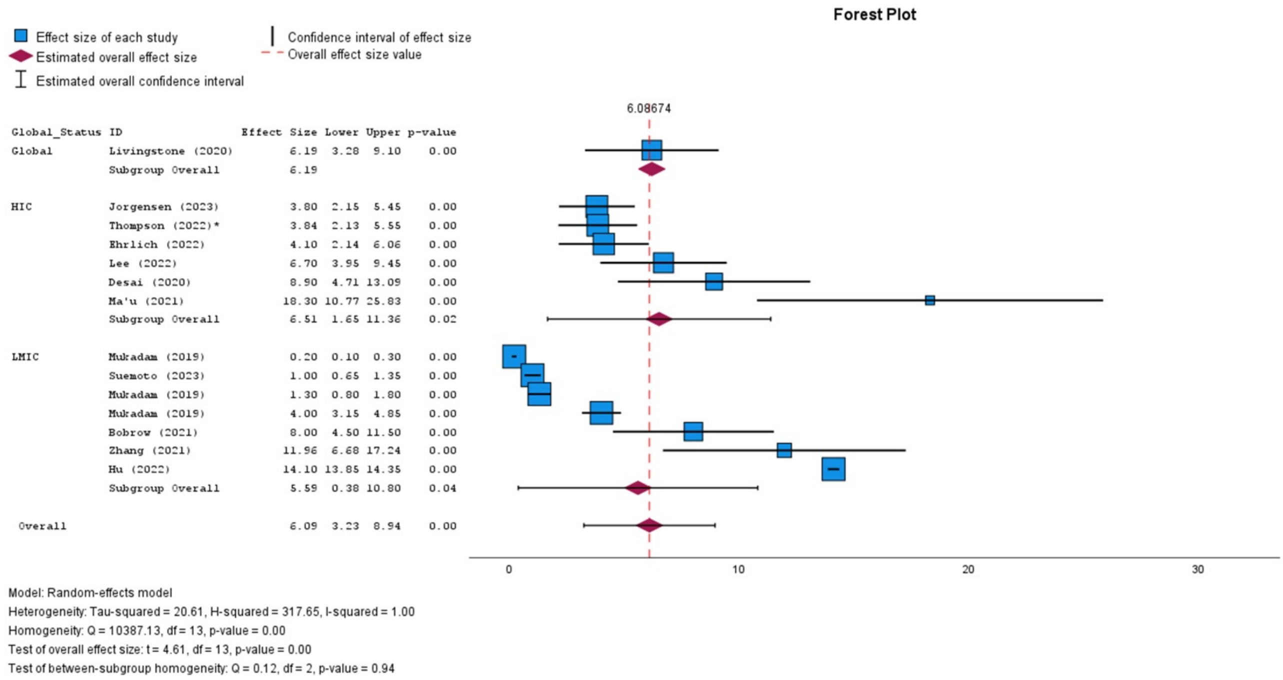

Physical Inactivity

Forest Plot

- Effect size of each study
- Estimated overall effect size
- Estimated overall confidence interval
- Confidence interval of effect size
- Overall effect size value

Global_Status	ID	Effect Size	Lower	Upper	p-value
Global	Livingstone (2020)	6.61	2.81	10.41	0.00
	Katzmarzyk (2022)	8.10	1.95	14.25	0.01
	Hazar (2016)	12.00	2.00	22.00	0.02
	Barnes (2011)	12.70	2.35	23.05	0.02
	Norton (2014)	12.70	2.35	23.05	0.02
	Dragioti (2022)	14.60	9.10	20.10	0.00
	Beydoun (2014)	31.90	22.65	41.15	0.00
	Subgroup Overall	13.65	6.03	21.26	0.00
HIC	Ehrlich (2022)	6.50	2.75	10.25	0.00
	Kotaki (2018)	7.30	1.30	13.30	0.02
	Thompson (2022)*	8.61	3.39	13.83	0.00
	Katzmarzyk (2022)	10.50	2.40	18.60	0.01
	Mukadam (2019)	11.80	11.70	11.90	0.00
	Rolandi (2020)	13.30	1.40	25.20	0.03
	Mayer (2018)	15.90	2.75	29.05	0.02
	Ma'u (2021)	17.60	8.80	26.40	0.00
	Ashby-Mitchell (2017)	17.90	8.35	27.45	0.00
	Jorgensen (2023)	18.50	8.75	28.25	0.00
	Lee (2022)	20.10	9.85	30.35	0.00
	Mayer (2018)	20.30	5.30	35.30	0.01
	Norton (2014)	20.30	5.30	35.30	0.01
	Barnes (2011)	21.00	5.60	36.40	0.01
	Norton (2014)	21.00	5.60	36.40	0.01
	Luck (2016)	21.70	5.95	37.45	0.01
	Norton (2014)	21.80	6.00	37.60	0.01
	MacDonald (2015)^	30.50	10.70	50.30	0.00
	Oliviera (2019)	30.70	10.85	50.55	0.00
	MacDonald (2015)#	32.50	12.00	53.00	0.00
	Subgroup Overall	15.10	11.76	18.44	0.00
LMIC	Katzmarzyk (2022)	4.90	1.05	8.75	0.01
	Zhang (2021)	6.29	2.66	9.92	0.00
	Oliviera (2019)	7.10	0.85	13.35	0.03
	Katzmarzyk (2022)	7.70	1.75	13.65	0.01
	Mukadam (2019)	8.40	7.20	9.60	0.00
	Feter (2022)	9.30	1.25	17.35	0.02
	Feter (2022)	11.50	8.79	14.21	0.00
	Vergara (2022)	11.90	5.50	18.30	0.00
	Feter (2022)	12.30	10.70	13.90	0.00
	Feter (2022)	12.80	10.65	14.95	0.00
	Suemoto (2023)	12.80	11.70	13.90	0.00
	Ashby-Mitchell (2020)	15.20	6.85	23.55	0.00
	Vergara (2022)	15.60	7.35	23.85	0.00
	Woo (2014)	16.10	3.55	28.65	0.01
	Ashby-Mitchell (2018)	16.30	7.45	25.15	0.00
	Mukadam (2019)	17.00	16.35	17.65	0.00
	Bobrow (2021)	20.00	10.50	29.50	0.00
	Hazar (2016)	22.00	6.00	38.00	0.01
	Mukadam (2019)	23.30	21.55	25.05	0.00
	Liu (2020)	24.30	3.31	45.29	0.02
	Oliviera (2019)	27.40	8.90	45.90	0.00
	Hu (2022)	43.60	42.50	44.70	0.00
	Subgroup Overall	15.11	10.94	19.29	0.00
Overall		15.26	12.78	17.75	0.00

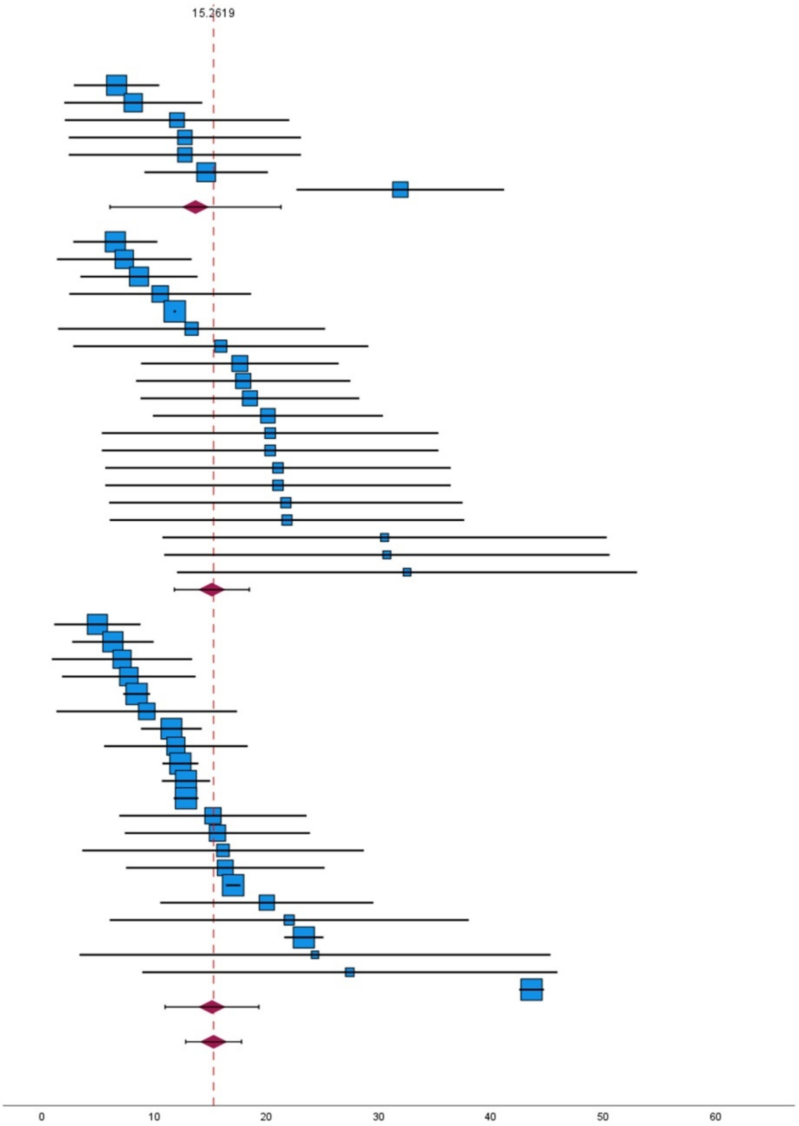

Model: Random-effects model
Heterogeneity: Tau-squared = 53.76, H-squared = 54.90, I-squared = 0.98
Homogeneity: Q = 3701.99, df = 48, p-value = 0.00
Test of overall effect size: t = 12.35, df = 48, p-value = 0.00
Test of between-subgroup homogeneity: Q = 0.19, df = 2, p-value = 0.91

Diabetes

Forest Plot

Effect size of each study | Confidence interval of effect size
Estimated overall effect size | Overall effect size value
No-effect value | Estimated overall confidence interval

Global_Status	ID	Effect Size	Lower	Upper	p-value
Global	Barnes (2011)	2.40	0.90	3.90	0.00
	Norton (2014)	2.90	1.20	4.60	0.00
	Dragioti (2022)	3.00	2.05	3.95	0.00
	Livingstone (2020)	3.10	1.61	4.59	0.00
	Dragioti (2022)	3.30	2.35	4.25	0.00
	Xu (2015)	3.70	3.21	4.19	0.00
	Hazar (2016)	4.80	1.95	7.65	0.00
	Vagelatos (2013)	6.00	4.32	7.68	0.00
	Dragioti (2022)	6.70	4.85	8.55	0.00
	Subgroup Overall	3.85	2.75	4.95	0.00
HIC	Norton (2014)	1.90	0.75	3.05	0.00
	Bothongo (2022)	2.30	1.65	2.95	0.00
	Ashby-Mitchell (2017)	2.40	0.95	3.85	0.00
	Shang (2022)	2.78	2.13	3.43	0.00
	Mayer (2018)	3.10	1.30	4.90	0.00
	Norton (2014)	3.10	1.30	4.90	0.00
	Barnes (2011)	3.30	1.35	5.25	0.00
	Jorgensen (2023)	3.30	1.90	4.70	0.00
	Woo (2014)	3.50	1.40	5.60	0.00
	Luck (2016)	4.10	1.65	6.55	0.00
	MacDonald (2015)^	4.10	1.55	6.65	0.00
	Oliviera (2019)	4.30	1.75	6.85	0.00
	Norton (2014)	4.50	1.85	7.15	0.00
	Ma'u (2021)	5.50	2.94	8.06	0.00
	MacDonald (2015)#	6.00	2.45	9.55	0.00
	Kotaki (2018)	6.60	2.84	10.36	0.00
	Mukadam (2019)	7.30	7.15	7.45	0.00
	Vagelatos (2013)	8.00	5.81	10.19	0.00
	Mayer (2018)	8.10	6.00	10.20	0.00
	Rolandi (2020)	9.90	2.20	17.60	0.01
	Ehrlich (2022)	10.90	6.14	15.66	0.00
	Lee (2022)	12.50	7.60	17.40	0.00
	Thompson (2022)*	23.02	15.22	30.82	0.00
	Subgroup Overall	5.20	3.65	6.75	0.00
LMIC	Oliviera (2019)	1.30	0.50	2.10	0.00
	Oliviera (2019)	2.80	1.10	4.50	0.00
	Hazar (2016)	3.60	1.40	5.80	0.00
	Hu (2022)	3.60	3.40	3.80	0.00
	Ashby-Mitchell (2020)	3.70	2.40	5.00	0.00
	Mukadam (2019)	4.40	3.50	5.30	0.00
	Mukadam (2019)	4.50	3.60	5.40	0.00
	Bobrow (2021)	5.00	2.50	7.50	0.00
	Liu (2020)	6.30	-1.20	13.80	0.10
	Zhang (2021)	7.60	4.14	11.06	0.00
	Ashby-Mitchell (2018)	8.00	3.45	12.55	0.00
	Mukadam (2019)	8.50	8.05	8.95	0.00
	Suemoto (2023)	9.00	8.05	9.95	0.00
	Vergara (2022)	9.20	4.90	13.50	0.00
	Vergara (2022)	14.00	7.85	20.15	0.00
	Subgroup Overall	5.58	3.91	7.25	0.00
Overall		4.96	4.09	5.83	0.00

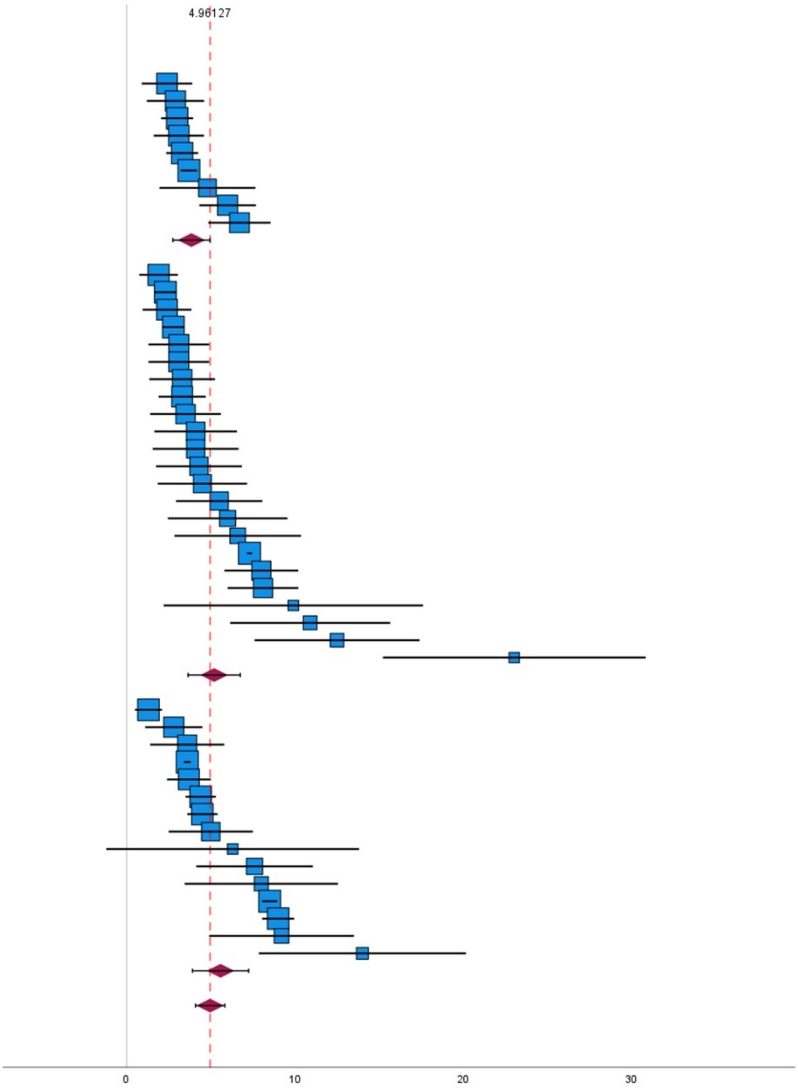

Model: Random-effects model
Heterogeneity: Tau-squared = 5.27, H-squared = 32.37, I-squared = 0.97
Homogeneity: Q = 1701.09, df = 46, p-value = 0.00
Test of overall effect size: t = 11.46, df = 46, p-value = 0.00
Test of between-subgroup homogeneity: Q = 5.27, df = 2, p-value = 0.07

Appendix 11 Forest plots summarising the unweighted population attributable fractions (PAF) for each individual risk factor stratified by dementia type

Acronyms AD=Alzheimer's disease; VaD=Vascular dementia

Key

#Indigenous Population Canada (MacDonald et al[39])

^Non-Indigenous Population Canada (MacDonald et al[39])

*Indigenous Population Australia (Thompson et al[63])

~Untreated Hypertension (Launer et al[31])

@Current smoking (Ren at al[47])

&Men (Ren et al[47], education)

$Women (Ren et al[47], education)

Education

Forest Plot

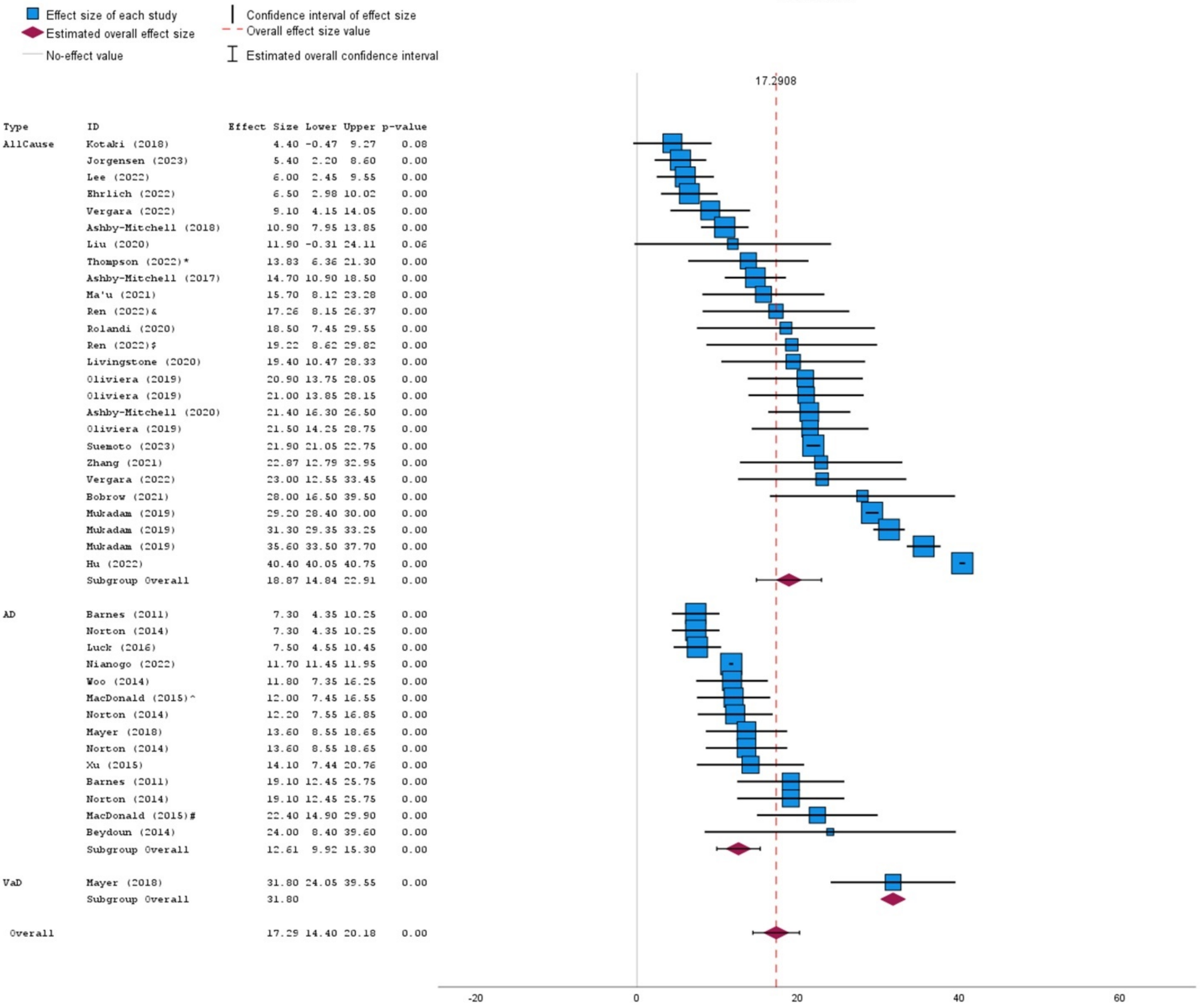

Type	ID	Effect Size	Lower	Upper	p-value
AllCause	Kotaki (2018)	4.40	-0.47	9.27	0.08
	Jorgensen (2023)	5.40	2.20	8.60	0.00
	Lee (2022)	6.00	2.45	9.55	0.00
	Ehrlich (2022)	6.50	2.98	10.02	0.00
	Vergara (2022)	9.10	4.15	14.05	0.00
	Ashby-Mitchell (2018)	10.90	7.95	13.85	0.00
	Liu (2020)	11.90	-0.31	24.11	0.06
	Thompson (2022)*	13.83	6.36	21.30	0.00
	Ashby-Mitchell (2017)	14.70	10.90	18.50	0.00
	Ma'u (2021)	15.70	8.12	23.28	0.00
	Ren (2022)&	17.26	8.15	26.37	0.00
	Rolandi (2020)	18.50	7.45	29.55	0.00
	Ren (2022)$	19.22	8.62	29.82	0.00
	Livingstone (2020)	19.40	10.47	28.33	0.00
	Oliviera (2019)	20.90	13.75	28.05	0.00
	Oliviera (2019)	21.00	13.85	28.15	0.00
	Ashby-Mitchell (2020)	21.40	16.30	26.50	0.00
	Oliviera (2019)	21.50	14.25	28.75	0.00
	Suemoto (2023)	21.90	21.05	22.75	0.00
	Zhang (2021)	22.87	12.79	32.95	0.00
	Vergara (2022)	23.00	12.55	33.45	0.00
	Bobrow (2021)	28.00	16.50	39.50	0.00
	Mukadam (2019)	29.20	28.40	30.00	0.00
	Mukadam (2019)	31.30	29.35	33.25	0.00
	Mukadam (2019)	35.60	33.50	37.70	0.00
	Hu (2022)	40.40	40.05	40.75	0.00
	Subgroup Overall	18.87	14.84	22.91	0.00
AD	Barnes (2011)	7.30	4.35	10.25	0.00
	Norton (2014)	7.30	4.35	10.25	0.00
	Luck (2016)	7.50	4.55	10.45	0.00
	Nianogo (2022)	11.70	11.45	11.95	0.00
	Woo (2014)	11.80	7.35	16.25	0.00
	MacDonald (2015)^	12.00	7.45	16.55	0.00
	Norton (2014)	12.20	7.55	16.85	0.00
	Mayer (2018)	13.60	8.55	18.65	0.00
	Norton (2014)	13.60	8.55	18.65	0.00
	Xu (2015)	14.10	7.44	20.76	0.00
	Barnes (2011)	19.10	12.45	25.75	0.00
	Norton (2014)	19.10	12.45	25.75	0.00
	MacDonald (2015)#	22.40	14.90	29.90	0.00
	Beydoun (2014)	24.00	8.40	39.60	0.00
	Subgroup Overall	12.61	9.92	15.30	0.00
VaD	Mayer (2018)	31.80	24.05	39.55	0.00
	Subgroup Overall	31.80			
Overall		17.29	14.40	20.18	0.00

Model: Random-effects model

Heterogeneity: Tau-squared = 73.38, H-squared = 125.92, I-squared = 0.99

Homogeneity: Q = 18463.74, df = 40, p-value = 0.00

Test of overall effect size: t = 12.08, df = 40, p-value = 0.00

Test of between-subgroup homogeneity: Q = 25.84, df = 2, p-value = 0.00

Hearing Loss

Forest Plot

Effect size of each study
Estimated overall effect size
No-effect value
Confidence interval of effect size
Overall effect size value
Estimated overall confidence interval

Type	ID	Effect Size	Lower	Upper	p-value
AllCause	Bothongo (2022)	1.30	0.84	1.76	0.00
	Shang (2022)	8.58	6.27	10.89	0.00
	Lee (2022)	8.90	3.00	14.80	0.00
	Jorgensen (2023)	9.10	3.30	14.90	0.00
	Mukadam (2019)	11.40	10.05	12.75	0.00
	Thompson (2022)*	12.36	4.75	19.97	0.00
	Ehrlich (2022)	14.70	5.96	23.44	0.00
	Mukadam (2019)	16.70	15.10	18.30	0.00
	Vergara (2022)	17.50	7.50	27.50	0.00
	Smith (2023)	18.66	-4.05	41.37	0.11
	Suemoto (2023)	19.20	18.20	20.20	0.00
	Mukadam (2019)	20.60	19.90	21.30	0.00
	Livingstone (2020)	22.20	10.32	34.08	0.00
	Ma'u (2021)	26.40	13.07	39.73	0.00
	Vergara (2022)	27.80	13.65	41.95	0.00
	Hu (2022)	39.20	37.75	40.65	0.00
	Subgroup Overall	16.64	11.33	21.95	0.00
AD	Mukadam (2019)	2.30	2.25	2.35	0.00
	Subgroup Overall	2.30			
Overall		15.69	10.40	20.97	0.00

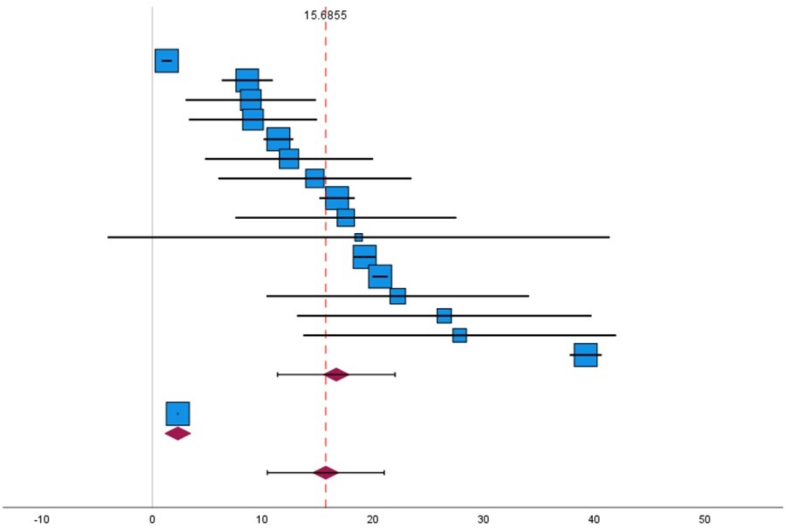

Model: Random-effects model
Heterogeneity: Tau-squared = 91.17, H-squared = 414.31, I-squared = 1.00
Homogeneity: Q = 6754.35, df = 16, p-value = 0.00
Test of overall effect size: t = 6.29, df = 16, p-value = 0.00
Test of between-subgroup homogeneity: Q = 33.08, df = 1, p-value = 0.00

Traumatic Brain Injury

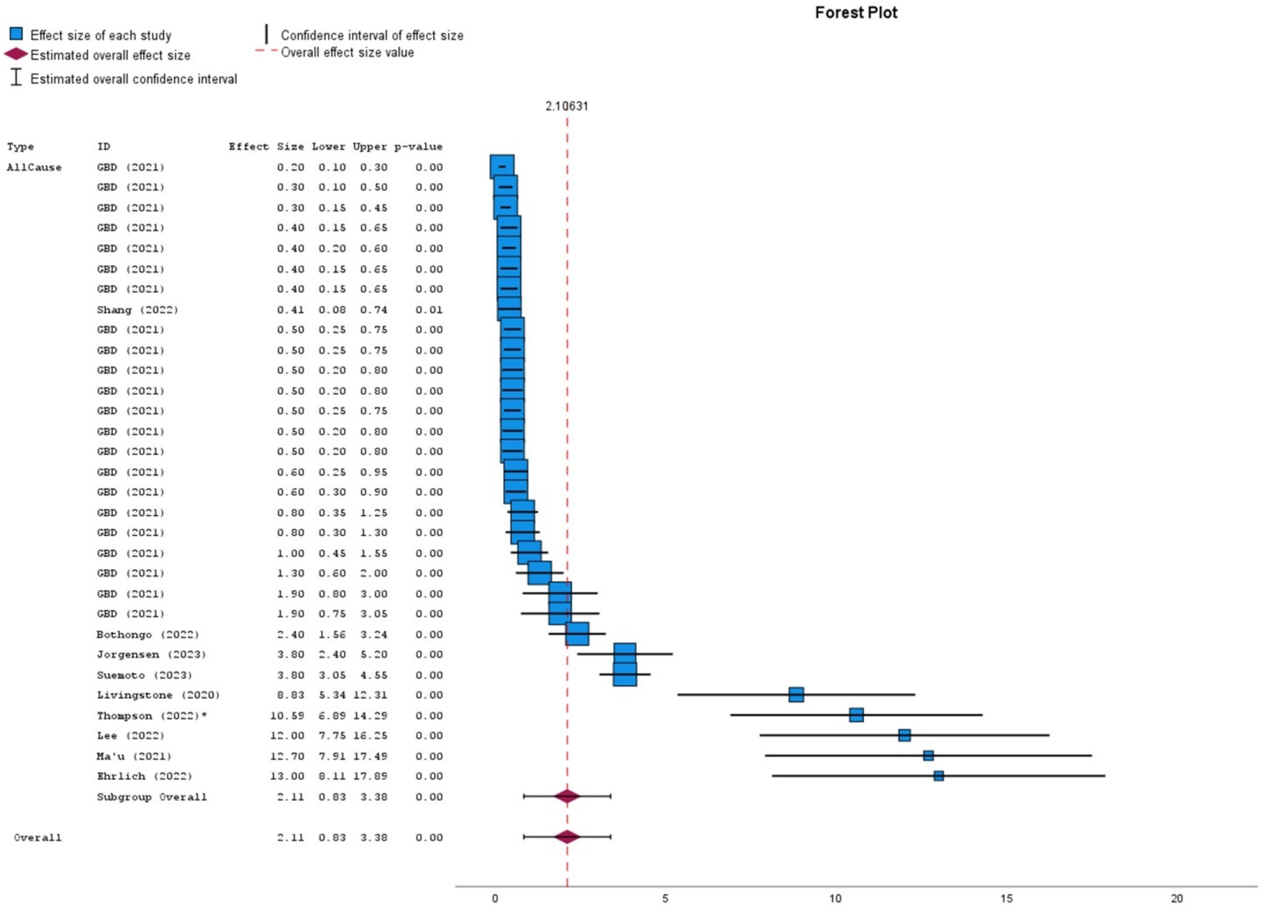

Model: Random-effects model
Heterogeneity: Tau-squared = 8.35, H-squared = 367.42, I-squared = 1.00
Homogeneity: Q = 308.22, df = 30, p-value = 0.00
Test of overall effect size: t = 3.38, df = 30, p-value = 0.00
Test of between-subgroup homogeneity: Q = 0.00, df = 0, p-value = 0.00

Hypertension

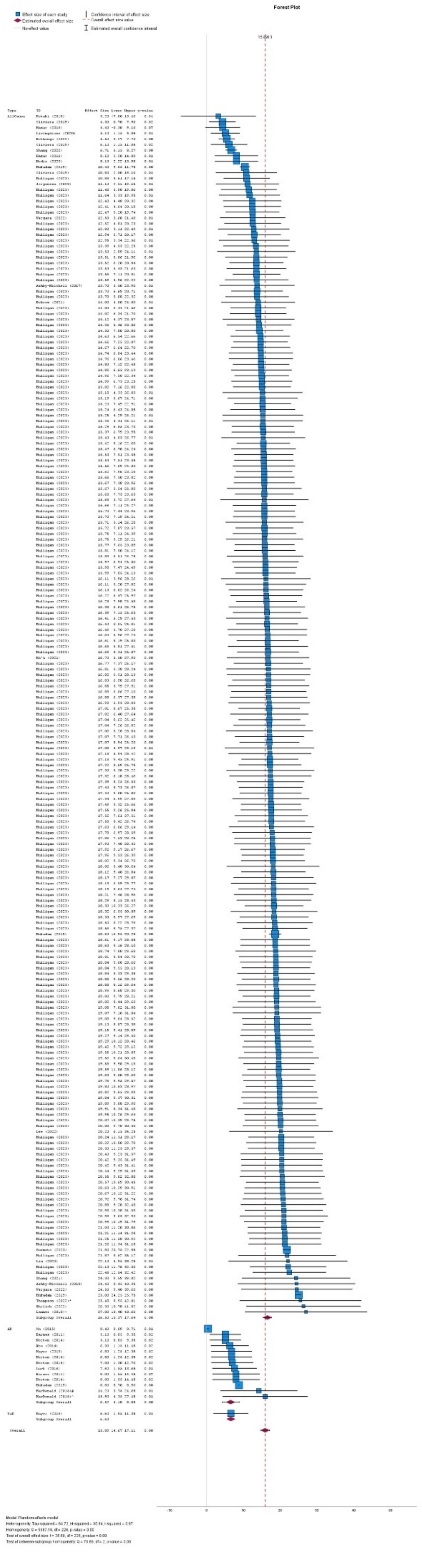

Alcohol Consumption

Effect size of each study
Estimated overall effect size
Estimated overall confidence interval
Confidence interval of effect size
Overall effect size value

Type	ID	Effect Size	Lower	Upper	p-value
AllCause	Ehrlich (2022)	0.60	0.32	0.88	0.00
	Jorgensen (2023)	1.70	0.55	2.85	0.00
	Lee (2022)	0.70	0.25	1.15	0.00
	Livingstone (2020)	2.10	0.97	3.23	0.00
	Ma'u (2021)	1.90	0.96	2.84	0.00
	Shang (2022)	0.40	0.02	0.78	0.04
	Suemoto (2023)	0.90	0.65	1.15	0.00
	Thompson (2022)*	0.68	0.21	1.15	0.00
	Vergara (2022)	1.10	0.40	1.80	0.00
	Vergara (2022)	0.30	0.05	0.55	0.02
	Subgroup Overall	0.84	0.45	1.22	0.00
Overall		0.84	0.45	1.22	0.00

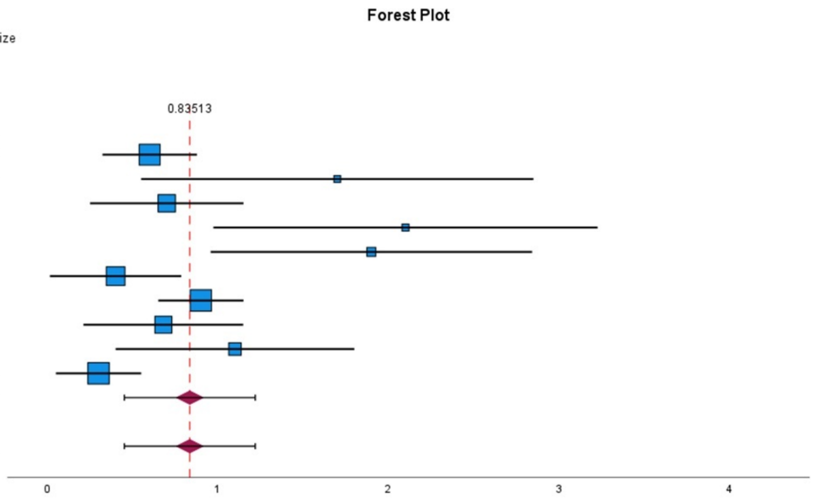

Model: Random-effects model
Heterogeneity: Tau-squared = 0.14, H-squared = 4.30, I-squared = 0.77
Homogeneity: Q = 31.06, df = 9, p-value = 0.00
Test of overall effect size: t = 4.92, df = 9, p-value = 0.00
Test of between-subgroup homogeneity: Q = 0.00, df = 0, p-value = 0.00

Obesity

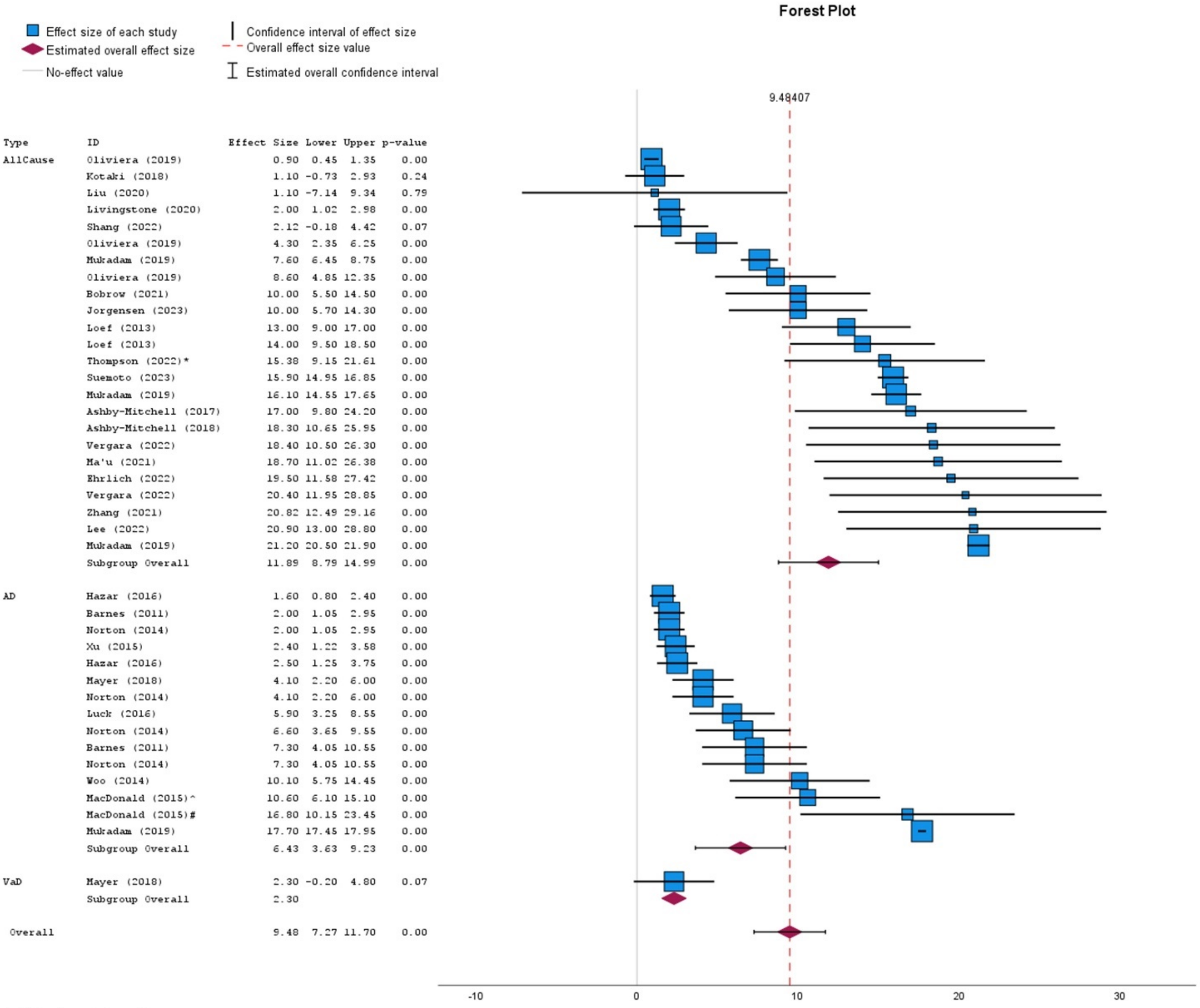

Type	ID	Effect Size	Lower	Upper	p-value
AllCause	Oliviera (2019)	0.90	0.45	1.35	0.00
	Kotaki (2018)	1.10	-0.73	2.93	0.24
	Liu (2020)	1.10	-7.14	9.34	0.79
	Livingstone (2020)	2.00	1.02	2.98	0.00
	Shang (2022)	2.12	-0.18	4.42	0.07
	Oliviera (2019)	4.30	2.35	6.25	0.00
	Mukadam (2019)	7.60	6.45	8.75	0.00
	Oliviera (2019)	8.60	4.85	12.35	0.00
	Bobrow (2021)	10.00	5.50	14.50	0.00
	Jorgensen (2023)	10.00	5.70	14.30	0.00
	Loef (2013)	13.00	9.00	17.00	0.00
	Loef (2013)	14.00	9.50	18.50	0.00
	Thompson (2022)*	15.38	9.15	21.61	0.00
	Suemoto (2023)	15.90	14.95	16.85	0.00
	Mukadam (2019)	16.10	14.55	17.65	0.00
	Ashby-Mitchell (2017)	17.00	9.80	24.20	0.00
	Ashby-Mitchell (2018)	18.30	10.65	25.95	0.00
	Vergara (2022)	18.40	10.50	26.30	0.00
	Ma'u (2021)	18.70	11.02	26.38	0.00
	Ehrlich (2022)	19.50	11.58	27.42	0.00
	Vergara (2022)	20.40	11.95	28.85	0.00
	Zhang (2021)	20.82	12.49	29.16	0.00
	Lee (2022)	20.90	13.00	28.80	0.00
	Mukadam (2019)	21.20	20.50	21.90	0.00
	Subgroup Overall	11.89	8.79	14.99	0.00
AD	Hazar (2016)	1.60	0.80	2.40	0.00
	Barnes (2011)	2.00	1.05	2.95	0.00
	Norton (2014)	2.00	1.05	2.95	0.00
	Xu (2015)	2.40	1.22	3.58	0.00
	Hazar (2016)	2.50	1.25	3.75	0.00
	Mayer (2018)	4.10	2.20	6.00	0.00
	Norton (2014)	4.10	2.20	6.00	0.00
	Luck (2016)	5.90	3.25	8.55	0.00
	Norton (2014)	6.60	3.65	9.55	0.00
	Barnes (2011)	7.30	4.05	10.55	0.00
	Norton (2014)	7.30	4.05	10.55	0.00
	Woo (2014)	10.10	5.75	14.45	0.00
	MacDonald (2015)^	10.60	6.10	15.10	0.00
	MacDonald (2015)#	16.80	10.15	23.45	0.00
	Mukadam (2019)	17.70	17.45	17.95	0.00
	Subgroup Overall	6.43	3.63	9.23	0.00
VaD	Mayer (2018)	2.30	-0.20	4.80	0.07
	Subgroup Overall	2.30			
Overall		9.48	7.27	11.70	0.00

Model: Random-effects model
Heterogeneity: Tau-squared = 42.45, H-squared = 106.63, I-squared = 0.99
Homogeneity: Q = 8030.76, df = 39, p-value = 0.00
Test of overall effect size: t = 8.65, df = 39, p-value = 0.00
Test of between-subgroup homogeneity: Q = 23.73, df = 2, p-value = 0.00

Smoking

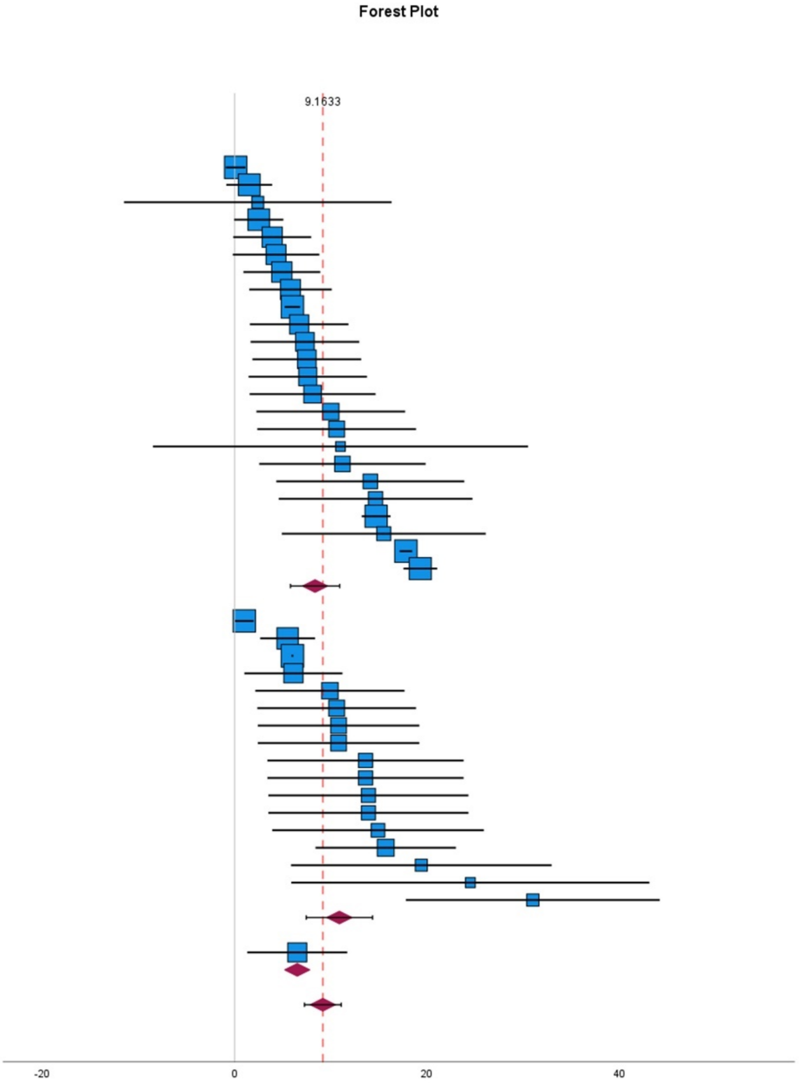

Effect size of each study
Estimated overall effect size
No-effect value
Confidence interval of effect size
Overall effect size value
Estimated overall confidence interval

Type	ID	Effect Size	Lower	Upper	p-value
AllCause	Bothongo (2022)	0.10	-0.92	1.12	0.85
	Ren (2022)@	1.51	-0.87	3.89	0.21
	Liu (2020)	2.40	-11.51	16.31	0.74
	Ashby-Mitchell (2018)	2.50	-0.05	5.05	0.06
	Ashby-Mitchell (2020)	3.90	-0.15	7.95	0.06
	Ashby-Mitchell (2017)	4.30	-0.20	8.80	0.06
	Lee (2022)	4.90	0.90	8.90	0.02
	Kotaki (2018)	5.80	1.51	10.09	0.01
	Suemoto (2023)	6.00	5.20	6.80	0.00
	Ehrlich (2022)	6.70	1.58	11.82	0.01
	Vergara (2022)	7.30	1.65	12.95	0.01
	Ma'u (2021)	7.50	1.84	13.16	0.01
	Jorgensen (2023)	7.60	1.45	13.75	0.02
	Oliviera (2019)	8.10	1.55	14.65	0.02
	Thompson (2022)*	9.99	2.26	17.72	0.01
	Oliviera (2019)	10.60	2.35	18.85	0.01
	Bobrow (2021)	11.00	-8.50	30.50	0.27
	Oliviera (2019)	11.20	2.55	19.85	0.01
	Livingstone (2020)	14.10	4.33	23.87	0.00
	Zhang (2021)	14.65	4.58	24.72	0.00
	Mukadam (2019)	14.70	13.20	16.20	0.00
	Vergara (2022)	15.50	4.90	26.10	0.00
	Mukadam (2019)	17.80	17.15	18.45	0.00
	Mukadam (2019)	19.30	17.55	21.05	0.00
	Subgroup Overall	8.35	5.80	10.91	0.00
AD	Xu (2015)	1.00	0.04	1.96	0.04
	Hazar (2016)	5.50	2.65	8.35	0.00
	Mukadam (2019)	6.00	5.90	6.10	0.00
	Woo (2014)	6.10	1.00	11.20	0.02
	MacDonald (2015)^	9.90	2.15	17.65	0.01
	Norton (2014)	10.60	2.35	18.85	0.01
	Barnes (2011)	10.80	2.40	19.20	0.01
	Norton (2014)	10.80	2.40	19.20	0.01
	Mayer (2018)	13.60	3.40	23.80	0.01
	Norton (2014)	13.60	3.40	23.80	0.01
	Barnes (2011)	13.90	3.50	24.30	0.01
	Norton (2014)	13.90	3.50	24.30	0.01
	Luck (2016)	14.90	3.90	25.90	0.01
	Hazar (2016)	15.70	8.40	23.00	0.00
	MacDonald (2015)#	19.40	5.85	32.95	0.01
	Xu (2015)	24.50	5.88	43.12	0.01
	Beydoun (2014)	31.00	17.80	44.20	0.00
	Subgroup Overall	10.88	7.44	14.33	0.00
VaD	Mayer (2018)	6.50	1.30	11.70	0.01
	Subgroup Overall	6.50			
Overall		9.16	7.25	11.07	0.00

Model: Random-effects model
Heterogeneity: Tau-squared = 25.53, H-squared = 36.97, I-squared = 0.97
Homogeneity: Q = 1905.79, df = 41, p-value = 0.00
Test of overall effect size: t = 9.68, df = 41, p-value = 0.00
Test of between-subgroup homogeneity: Q = 2.50, df = 2, p-value = 0.29

Depression Lifetime

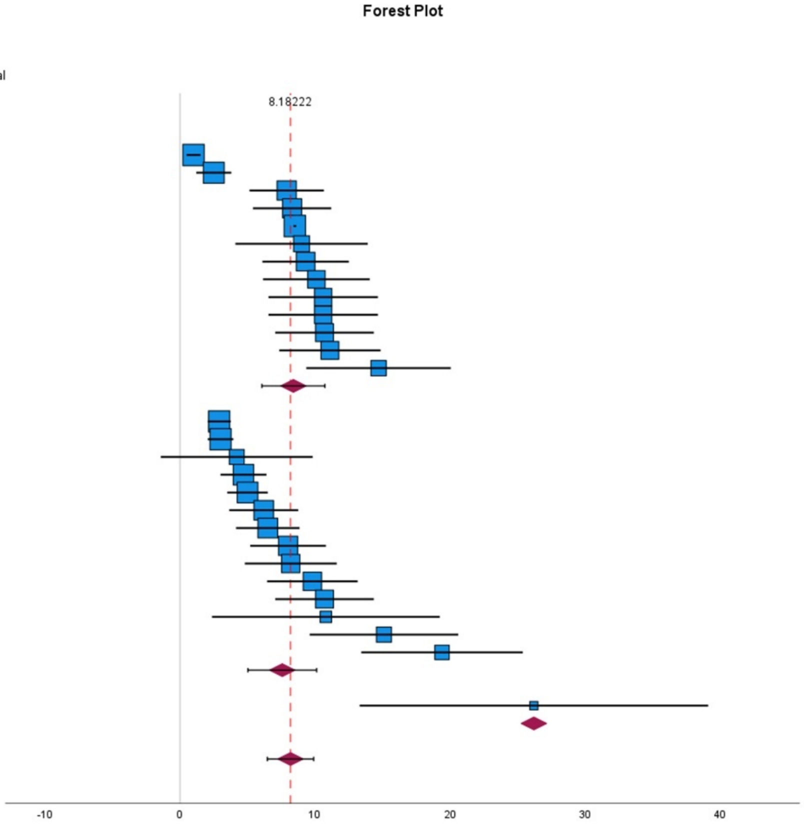

Type	ID	Effect Size	Lower	Upper	p-value
AllCause	Xu (2015)	1.00	0.48	1.52	0.00
	Dragioti (2022)	2.50	1.20	3.80	0.00
	Norton (2014)	7.90	5.15	10.65	0.00
	Norton (2014)	8.30	5.40	11.20	0.00
	Mukadam (2019)	8.50	8.40	8.60	0.00
	Santabarbara (2020b)	9.00	4.10	13.90	0.00
	Luck (2016)	9.30	6.10	12.50	0.00
	Woo (2014)	10.10	6.15	14.05	0.00
	Barnes (2011)	10.60	6.55	14.65	0.00
	Jorgensen (2023)	10.60	6.55	14.65	0.00
	Norton (2014)	10.70	7.05	14.35	0.00
	Norton (2014)	11.10	7.35	14.85	0.00
	Barnes (2011)	14.70	9.35	20.05	0.00
	Subgroup Overall	8.39	6.06	10.73	0.00
AD	Ashby-Mitchell (2020)	2.90	2.05	3.75	0.00
	Dragioti (2022)	3.00	2.05	3.95	0.00
	Liu (2020)	4.20	-1.42	9.82	0.14
	Oliviera (2019)	4.70	3.00	6.40	0.00
	Bobrow (2021)	5.00	3.50	6.50	0.00
	Lee (2022)	6.20	3.65	8.75	0.00
	Oliviera (2019)	6.50	4.15	8.85	0.00
	Ashby-Mitchell (2017)	8.00	5.20	10.80	0.00
	Vergara (2022)	8.20	4.80	11.60	0.00
	Oliviera (2019)	9.80	6.45	13.15	0.00
	Mayer (2018)	10.70	7.05	14.35	0.00
	Santabarbara (2019a)	10.80	2.37	19.23	0.01
	Vergara (2022)	15.10	9.60	20.60	0.00
	Ehrlich (2022)	19.40	13.42	25.38	0.00
	Subgroup Overall	7.57	5.03	10.11	0.00
VaD	Mayer (2018)	26.20	13.30	39.10	0.00
	Subgroup Overall	26.20			
Overall		8.18	6.47	9.90	0.00

Model: Random-effects model
Heterogeneity: Tau-squared = 13.70, H-squared = 35.03, I-squared = 0.97
Homogeneity: Q = 1194.43, df = 27, p-value = 0.00
Test of overall effect size: t = 9.79, df = 27, p-value = 0.00
Test of between-subgroup homogeneity: Q = 7.82, df = 2, p-value = 0.02

Depression Late Life

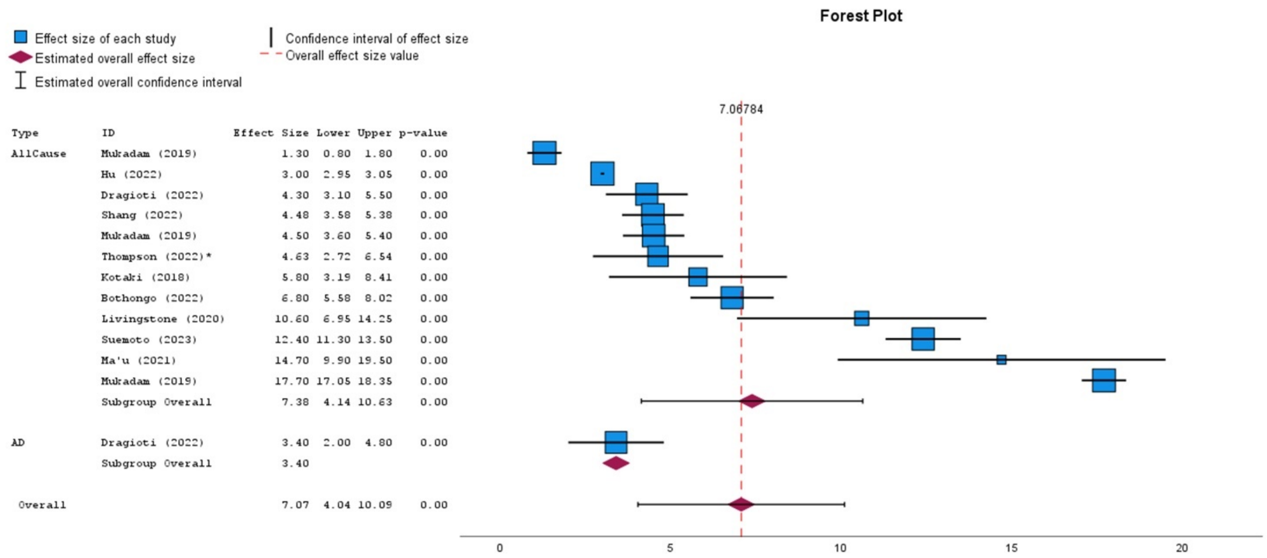

Model: Random-effects model
Heterogeneity: Tau-squared = 24.01, H-squared = 183.28, I-squared = 0.99
Homogeneity: Q = 2380.48, df = 12, p-value = 0.00
Test of overall effect size: t = 5.09, df = 12, p-value = 0.00
Test of between-subgroup homogeneity: Q = 5.92, df = 1, p-value = 0.01

Social Isolation

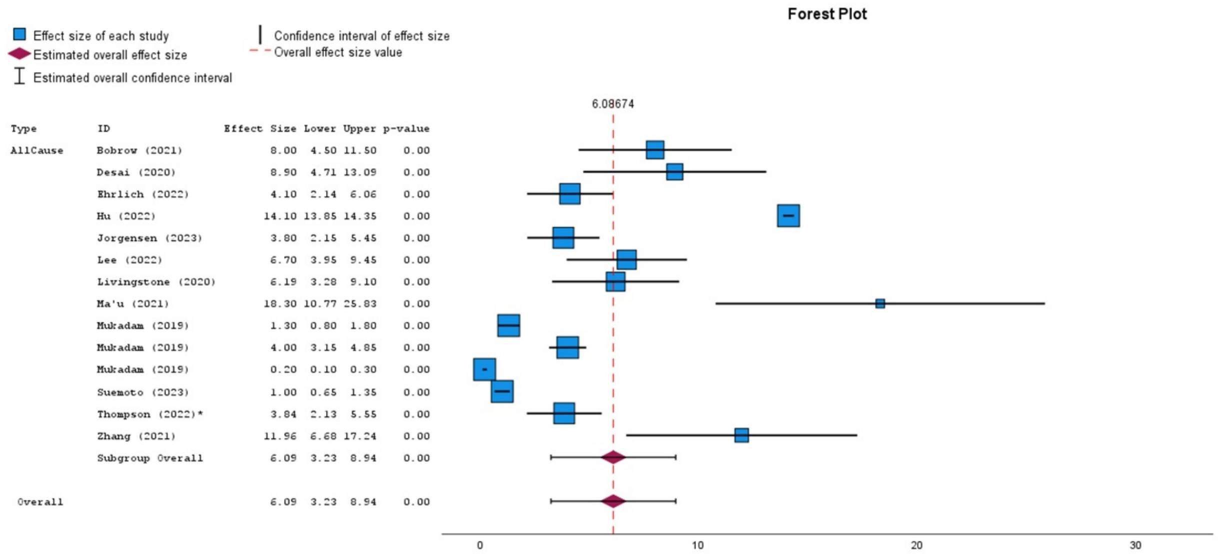

Type	ID	Effect Size	Lower	Upper	p-value
AllCause	Bobrow (2021)	8.00	4.50	11.50	0.00
	Desai (2020)	8.90	4.71	13.09	0.00
	Ehrlich (2022)	4.10	2.14	6.06	0.00
	Hu (2022)	14.10	13.85	14.35	0.00
	Jorgensen (2023)	3.80	2.15	5.45	0.00
	Lee (2022)	6.70	3.95	9.45	0.00
	Livingstone (2020)	6.19	3.28	9.10	0.00
	Ma'u (2021)	18.30	10.77	25.83	0.00
	Mukadam (2019)	1.30	0.80	1.80	0.00
	Mukadam (2019)	4.00	3.15	4.85	0.00
	Mukadam (2019)	0.20	0.10	0.30	0.00
	Suemoto (2023)	1.00	0.65	1.35	0.00
	Thompson (2022)*	3.84	2.13	5.55	0.00
	Zhang (2021)	11.96	6.68	17.24	0.00
	Subgroup Overall	6.09	3.23	8.94	0.00
Overall		6.09	3.23	8.94	0.00

Model: Random-effects model
Heterogeneity: Tau-squared = 20.61, H-squared = 317.65, I-squared = 1.00
Homogeneity: Q = 10387.13, df = 13, p-value = 0.00
Test of overall effect size: t = 4.61, df = 13, p-value = 0.00
Test of between-subgroup homogeneity: Q = 0.00, df = 0, p-value = 0.00

Physical Inactivity

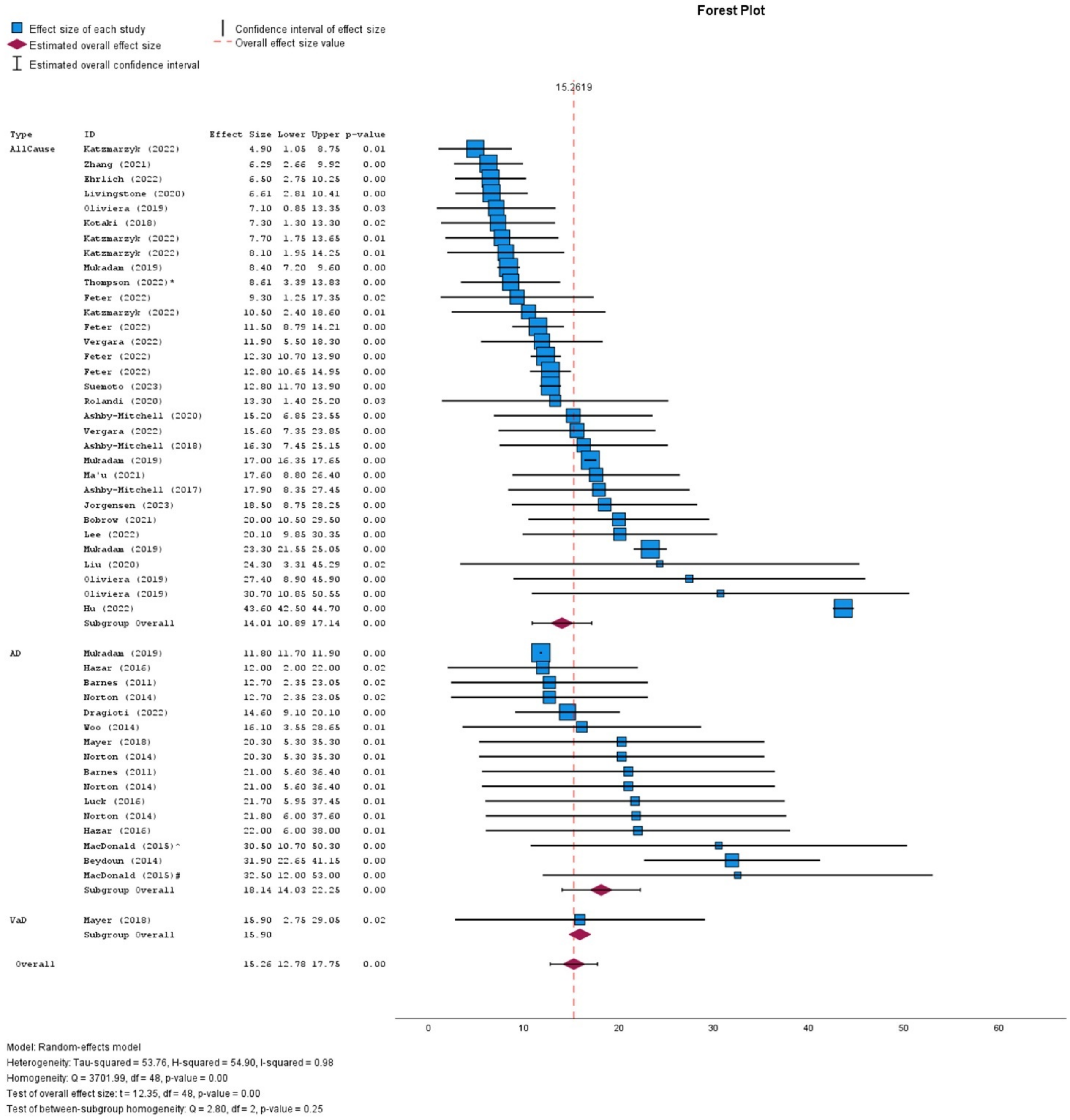

Diabetes

Forest Plot

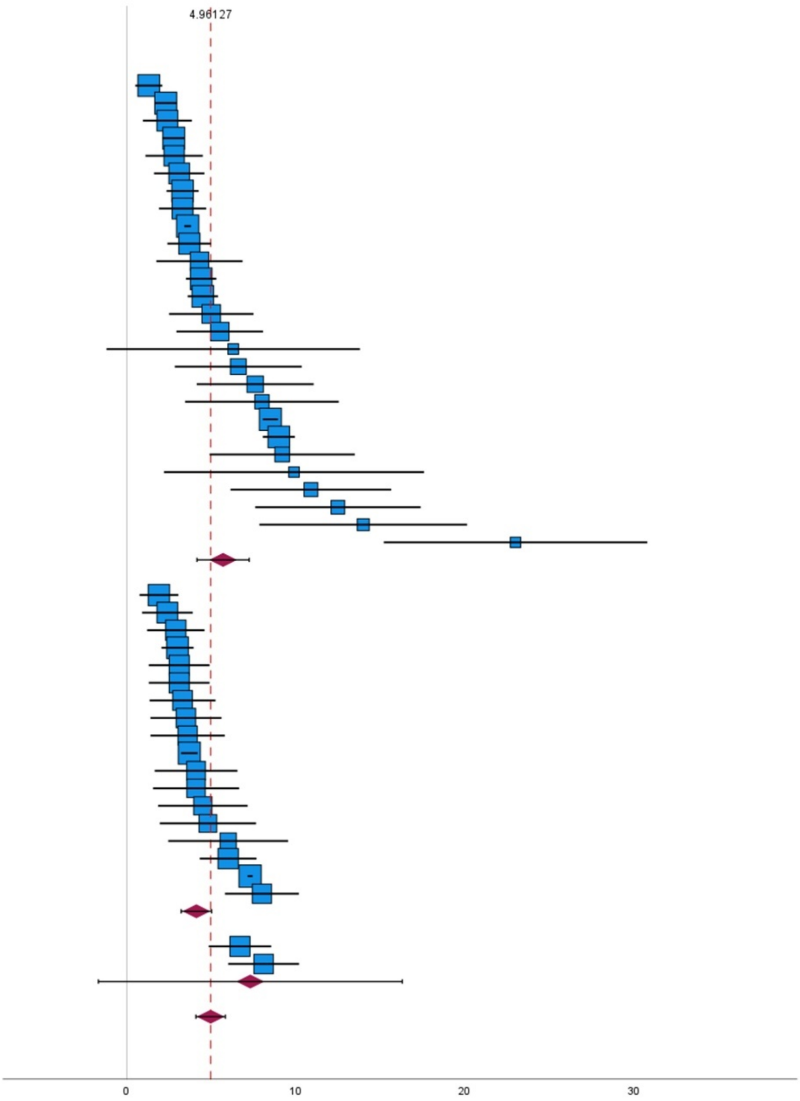

Type	ID	Effect Size	Lower	Upper	p-value
AllCause	Oliviera (2019)	1.30	0.50	2.10	0.00
	Bothongo (2022)	2.30	1.65	2.95	0.00
	Ashby-Mitchell (2017)	2.40	0.95	3.85	0.00
	Shang (2022)	2.78	2.13	3.43	0.00
	Oliviera (2019)	2.80	1.10	4.50	0.00
	Livingstone (2020)	3.10	1.61	4.59	0.00
	Dragioti (2022)	3.30	2.35	4.25	0.00
	Jorgensen (2023)	3.30	1.90	4.70	0.00
	Hu (2022)	3.60	3.40	3.80	0.00
	Ashby-Mitchell (2020)	3.70	2.40	5.00	0.00
	Oliviera (2019)	4.30	1.75	6.85	0.00
	Mukadam (2019)	4.40	3.50	5.30	0.00
	Mukadam (2019)	4.50	3.60	5.40	0.00
	Bobrow (2021)	5.00	2.50	7.50	0.00
	Ma'u (2021)	5.50	2.94	8.06	0.00
	Liu (2020)	6.30	-1.20	13.80	0.10
	Kotaki (2018)	6.60	2.84	10.36	0.00
	Zhang (2021)	7.60	4.14	11.06	0.00
	Ashby-Mitchell (2018)	8.00	3.45	12.55	0.00
	Mukadam (2019)	8.50	8.05	8.95	0.00
	Suemoto (2023)	9.00	8.05	9.95	0.00
	Vergara (2022)	9.20	4.90	13.50	0.00
	Rolandi (2020)	9.90	2.20	17.60	0.01
	Ehrlich (2022)	10.90	6.14	15.66	0.00
	Lee (2022)	12.50	7.60	17.40	0.00
	Vergara (2022)	14.00	7.85	20.15	0.00
	Thompson (2022)*	23.02	15.22	30.82	0.00
	Subgroup Overall	5.70	4.15	7.25	0.00
AD	Norton (2014)	1.90	0.75	3.05	0.00
	Barnes (2011)	2.40	0.90	3.90	0.00
	Norton (2014)	2.90	1.20	4.60	0.00
	Dragioti (2022)	3.00	2.05	3.95	0.00
	Mayer (2018)	3.10	1.30	4.90	0.00
	Norton (2014)	3.10	1.30	4.90	0.00
	Barnes (2011)	3.30	1.35	5.25	0.00
	Woo (2014)	3.50	1.40	5.60	0.00
	Hazar (2016)	3.60	1.40	5.80	0.00
	Xu (2015)	3.70	3.21	4.19	0.00
	Luck (2016)	4.10	1.65	6.55	0.00
	MacDonald (2015)^	4.10	1.55	6.65	0.00
	Norton (2014)	4.50	1.85	7.15	0.00
	Hazar (2016)	4.80	1.95	7.65	0.00
	MacDonald (2015)#	6.00	2.45	9.55	0.00
	Vagelatos (2013)	6.00	4.32	7.68	0.00
	Mukadam (2019)	7.30	7.15	7.45	0.00
	Vagelatos (2013)	8.00	5.81	10.19	0.00
	Subgroup Overall	4.12	3.22	5.02	0.00
VaD	Dragioti (2022)	6.70	4.85	8.55	0.00
	Mayer (2018)	8.10	6.00	10.20	0.00
	Subgroup Overall	7.31	-1.69	16.31	0.06
Overall		4.96	4.09	5.83	0.00

Model: Random-effects model
Heterogeneity: Tau-squared = 5.27, H-squared = 32.37, I-squared = 0.97
Homogeneity: Q = 1701.09, df = 46, p-value = 0.00
Test of overall effect size: t = 11.46, df = 46, p-value = 0.00
Test of between-subgroup homogeneity: Q = 15.81, df = 2, p-value = 0.00

Appendix 12 Sensitivity analyses to evaluate the effects of removal of low-quality studies (n=7) on the unweighted PAF value for each factor

Effect Size Estimates for Subgroup Analysis

	Number of Estimates	Effect Size	Std. Error	Z	Sig. (2-tailed)	95% Confidence Interval Lower	Upper
Education	39.00	17.338	1.4799	11.716	<.001	14.438	20.239
Hearing	17.00	15.685	2.4939	6.289	<.001	10.797	20.573
Traumatic Brain Injury	31.00	2.106	.5369	3.923	<.001	1.054	3.159
Hypertension	40.00	10.898	1.1222	9.711	<.001	8.699	13.098
Alcohol	10.00	.835	.1491	5.599	<.001	.543	1.127
Obesity	37.00	9.279	1.1634	7.976	<.001	6.999	11.559
Smoking	40.00	8.929	.9409	9.489	<.001	7.084	10.773
Depression-Lifetime	24.00	8.616	.8383	10.279	<.001	6.973	10.259
Depression-Late Life	11.00	7.681	1.5894	4.832	<.001	4.565	10.796
Social Isolation	14.00	6.087	1.2742	4.777	<.001	3.589	8.584
Physical Activity	46.00	14.893	1.2633	11.790	<.001	12.417	17.369
Diabetes	41.00	4.994	.4295	11.628	<.001	4.152	5.835
Overall	350.00	9.640	.4229	22.794	<.001	8.811	10.469

Appendix 13 Forest plots summarising the weighted population attributable fractions (PAF) for each individual risk factor

Acronyms HIC=High income countries; LMIC=Low- and middle-income countries

Key

*Indigenous Population Australia (Thompson et al[63])

Education

Forest Plot

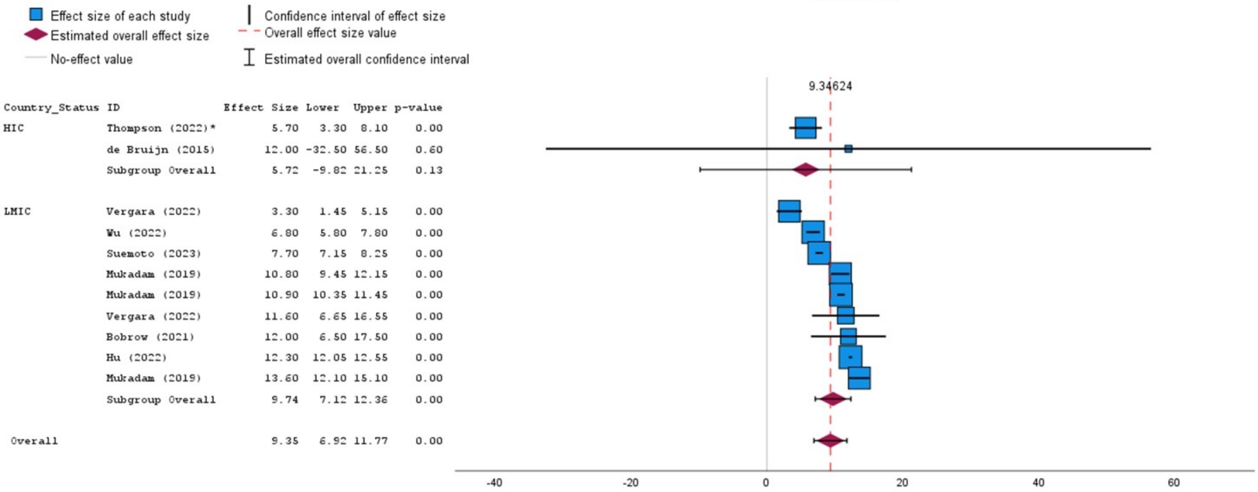

Country_Status	ID	Effect Size	Lower	Upper	p-value
HIC	Thompson (2022)*	5.70	3.30	8.10	0.00
	de Bruijn (2015)	12.00	-32.50	56.50	0.60
	Subgroup Overall	5.72	-9.82	21.25	0.13
LMIC	Vergara (2022)	3.30	1.45	5.15	0.00
	Wu (2022)	6.80	5.80	7.80	0.00
	Suemoto (2023)	7.70	7.15	8.25	0.00
	Mukadam (2019)	10.80	9.45	12.15	0.00
	Mukadam (2019)	10.90	10.35	11.45	0.00
	Vergara (2022)	11.60	6.65	16.55	0.00
	Bobrow (2021)	12.00	6.50	17.50	0.00
	Hu (2022)	12.30	12.05	12.55	0.00
	Mukadam (2019)	13.60	12.10	15.10	0.00
	Subgroup Overall	9.74	7.12	12.36	0.00
Overall		9.35	6.92	11.77	0.00

Model: Random-effects model
Heterogeneity: Tau-squared = 10.50, H-squared = 57.57, I-squared = 0.98
Homogeneity: Q = 405.51, df = 10, p-value = 0.00
Test of overall effect size: t = 8.59, df = 10, p-value = 0.00
Test of between-subgroup homogeneity: Q = 5.79, df = 1, p-value = 0.02

Hearing Loss

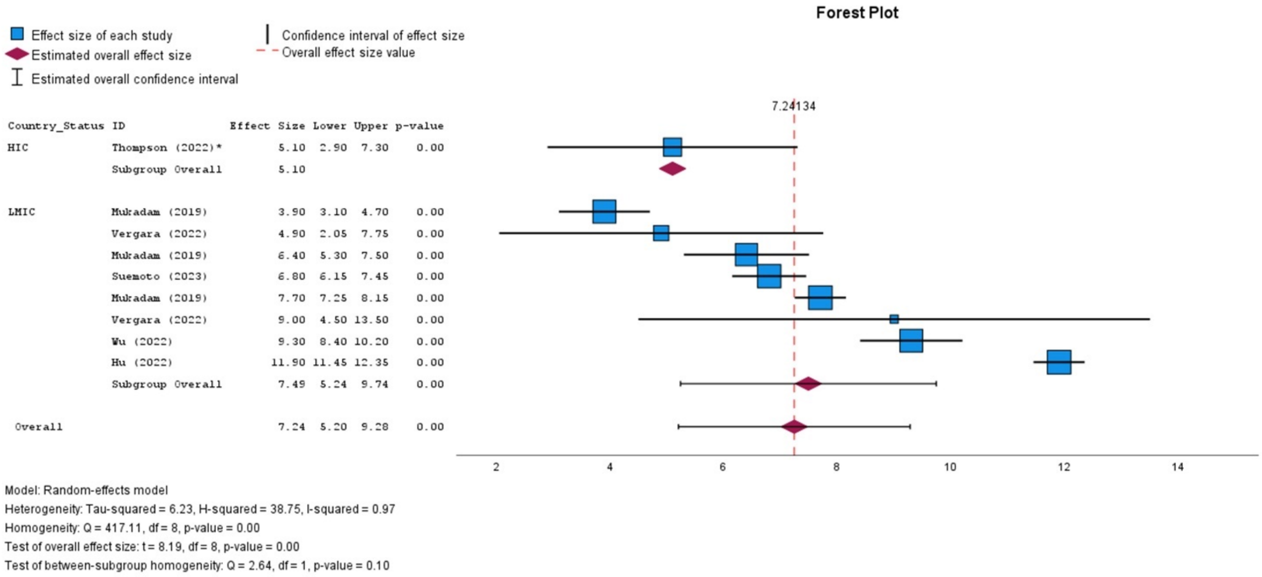

Traumatic Brain Injury

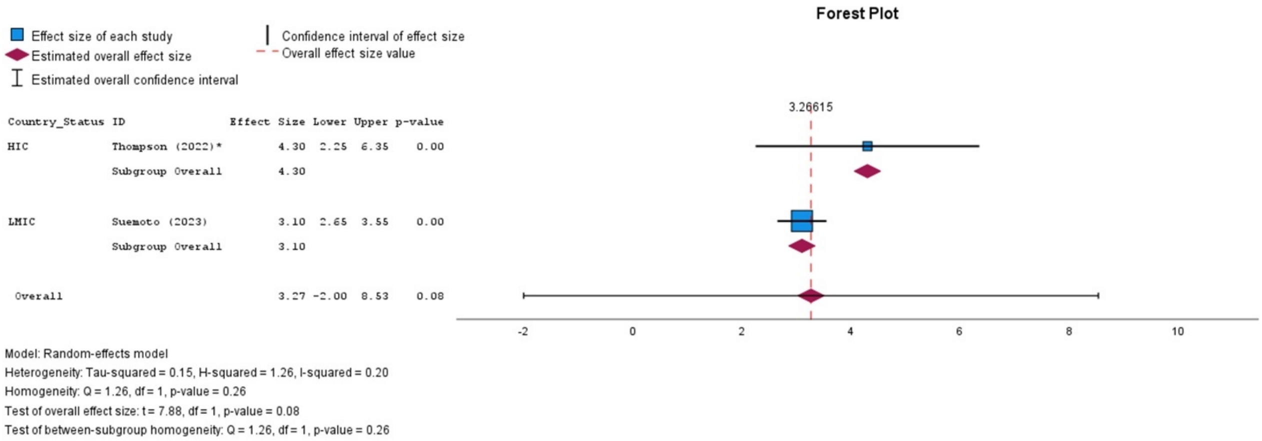

Hypertension

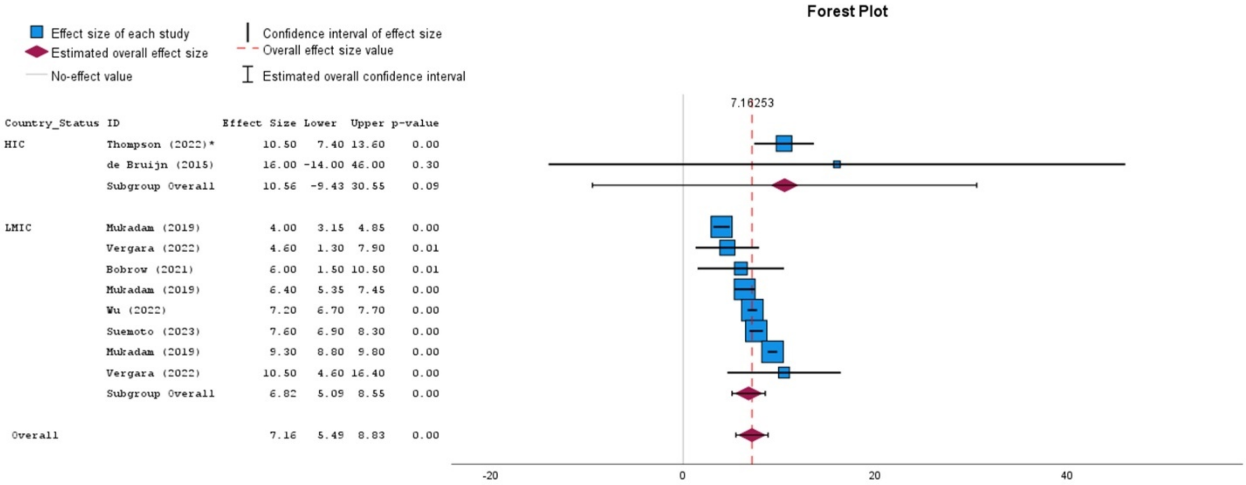

Country_Status	ID	Effect Size	Lower	Upper	p-value
HIC	Thompson (2022)*	10.50	7.40	13.60	0.00
	de Bruijn (2015)	16.00	-14.00	46.00	0.30
	Subgroup Overall	10.56	-9.43	30.55	0.09
LMIC	Mukadam (2019)	4.00	3.15	4.85	0.00
	Vergara (2022)	4.60	1.30	7.90	0.01
	Bobrow (2021)	6.00	1.50	10.50	0.01
	Mukadam (2019)	6.40	5.35	7.45	0.00
	Wu (2022)	7.20	6.70	7.70	0.00
	Suemoto (2023)	7.60	6.90	8.30	0.00
	Mukadam (2019)	9.30	8.80	9.80	0.00
	Vergara (2022)	10.50	4.60	16.40	0.00
	Subgroup Overall	6.82	5.09	8.55	0.00
Overall		7.16	5.49	8.83	0.00

Model: Random-effects model
Heterogeneity: Tau-squared = 3.64, H-squared = 15.80, I-squared = 0.94
Homogeneity: Q = 128.84, df = 9, p-value = 0.00
Test of overall effect size: t = 9.70, df = 9, p-value = 0.00
Test of between-subgroup homogeneity: Q = 4.65, df = 1, p-value = 0.03

Alcohol Consumption

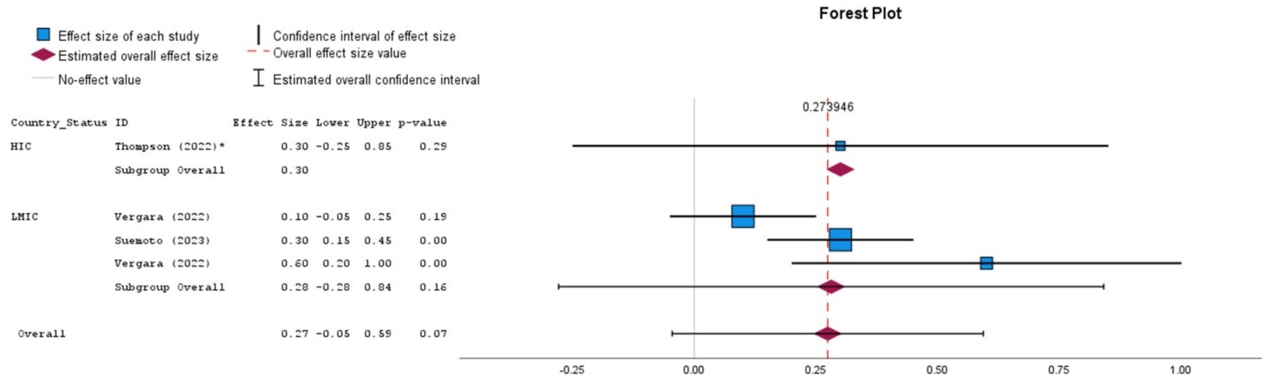

Model: Random-effects model
Heterogeneity: Tau-squared = 0.02, H-squared = 2.58, I-squared = 0.61
Homogeneity: Q = 7.07, df = 3, p-value = 0.07
Test of overall effect size: t = 2.73, df = 3, p-value = 0.07
Test of between-subgroup homogeneity: Q = 0.00, df = 1, p-value = 0.95

Obesity

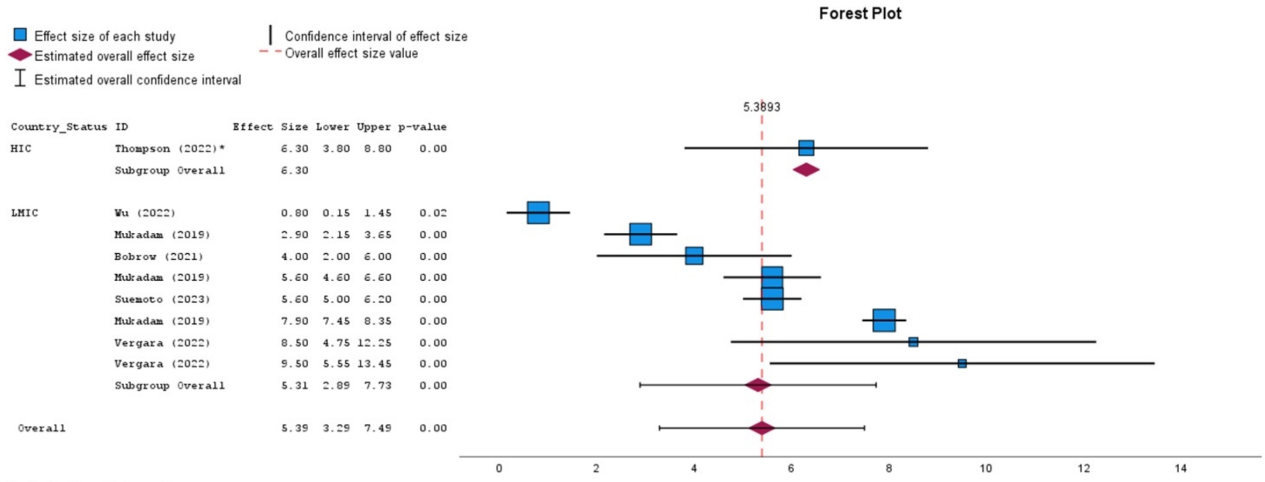

Model: Random-effects model
Heterogeneity: Tau-squared = 6.48, H-squared = 32.99, I-squared = 0.97
Homogeneity: Q = 362.28, df = 8, p-value = 0.00
Test of overall effect size: t = 5.91, df = 8, p-value = 0.00
Test of between-subgroup homogeneity: Q = 0.37, df = 1, p-value = 0.54

Smoking

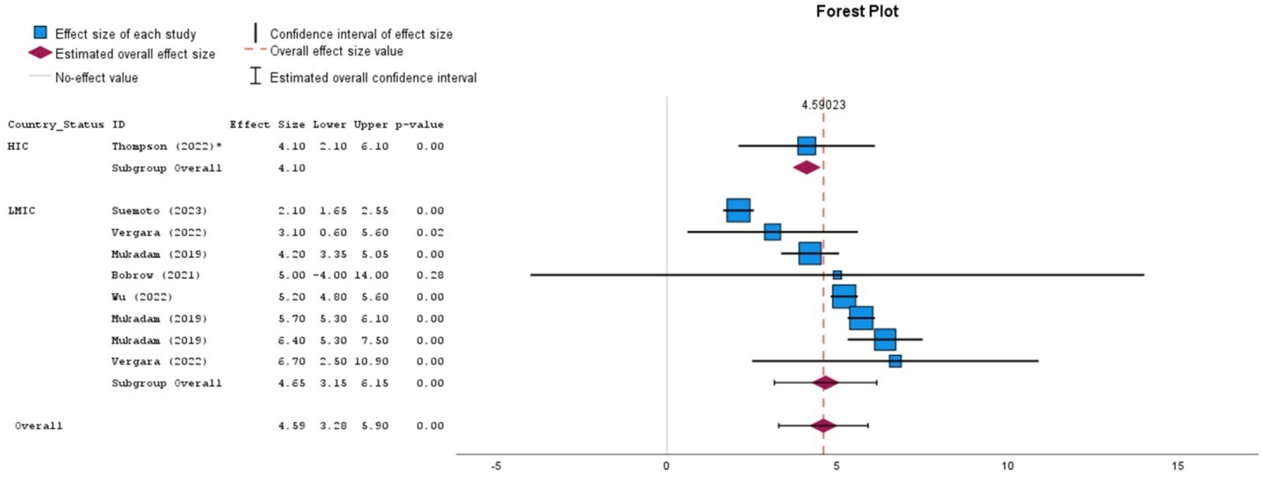

Model: Random-effects model
Heterogeneity: Tau-squared = 2.05, H-squared = 15.64, I-squared = 0.94
Homogeneity: Q = 169.79, df = 8, p-value = 0.00
Test of overall effect size: t = 8.08, df = 8, p-value = 0.00
Test of between-subgroup homogeneity: Q = 0.21, df = 1, p-value = 0.65

Depression Lifetime

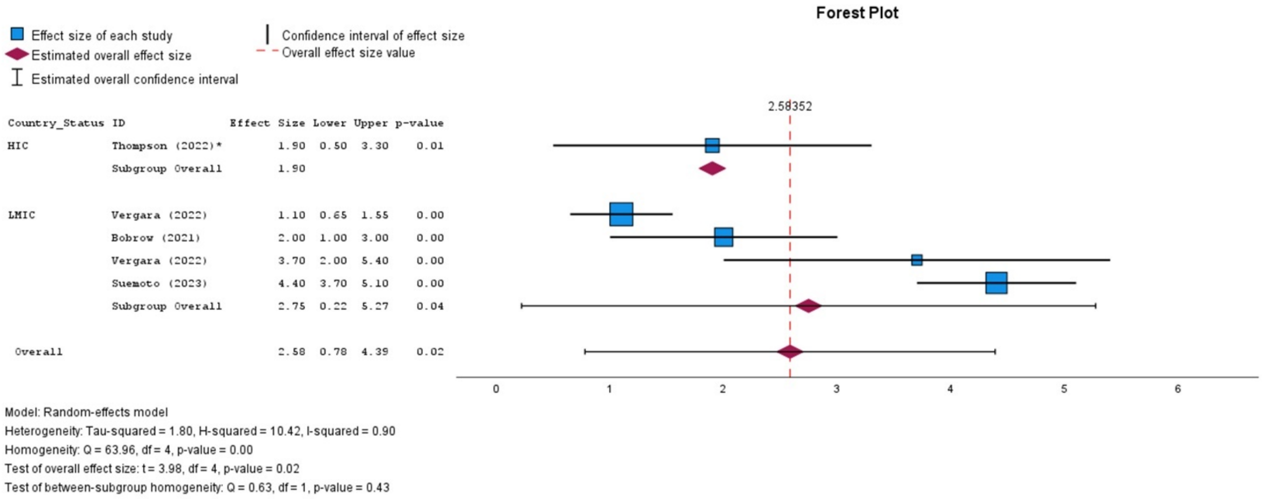

Depression Late Life

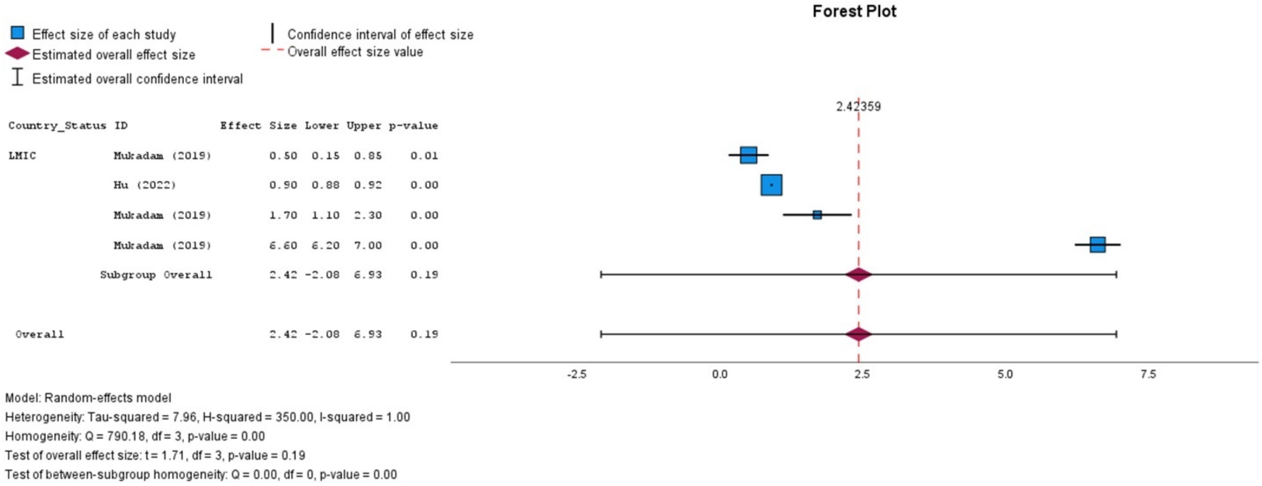

Social Isolation

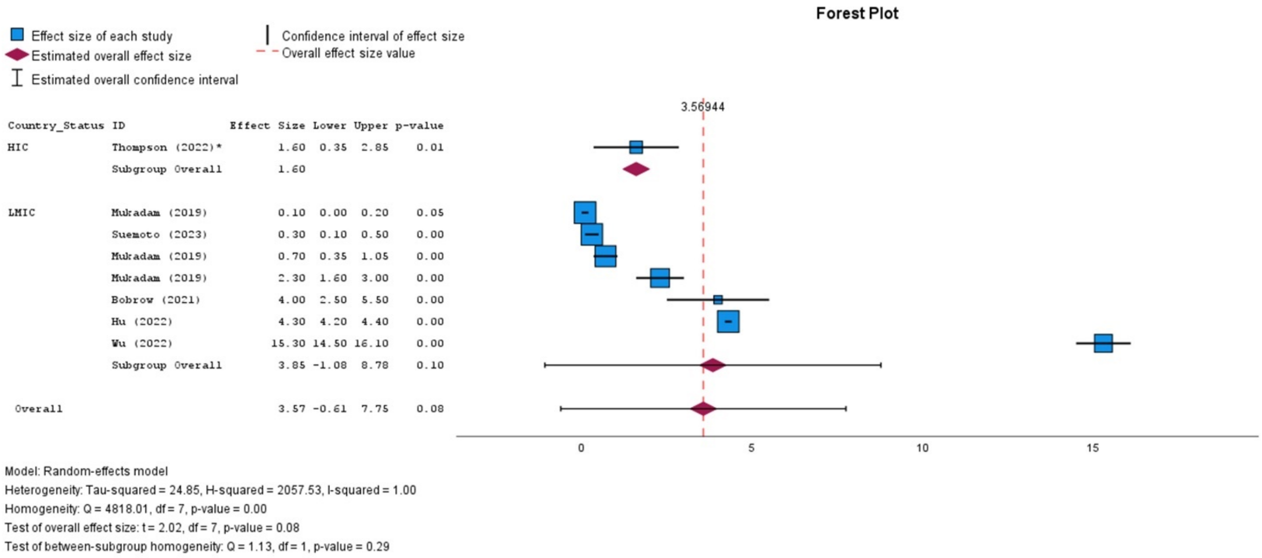

Physical Inactivity

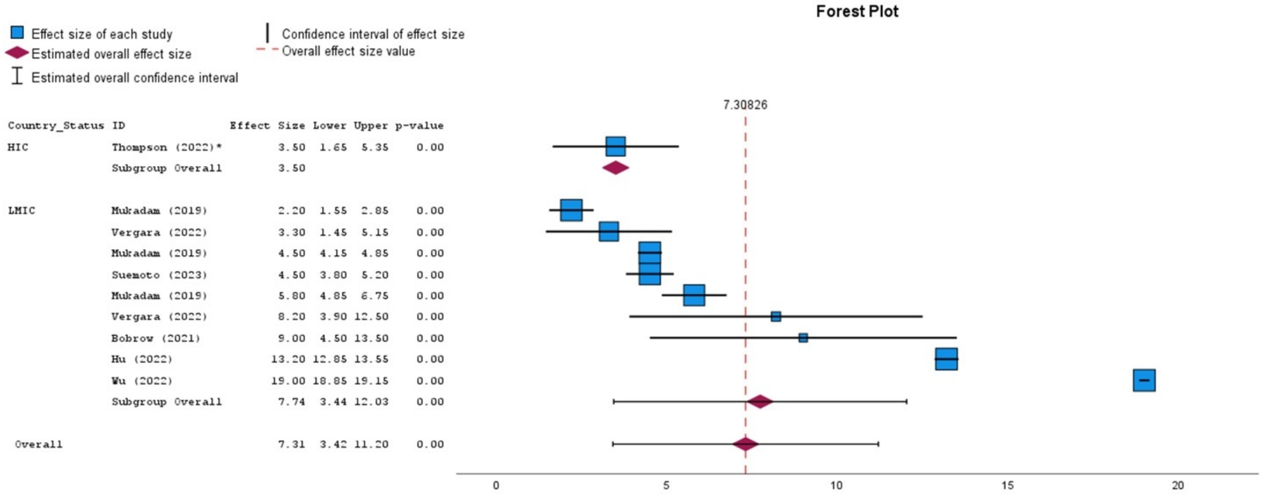

Model: Random-effects model
Heterogeneity: Tau-squared = 28.48, H-squared = 430.39, I-squared = 1.00
Homogeneity: Q = 9320.07, df = 9, p-value = 0.00
Test of overall effect size: t = 4.25, df = 9, p-value = 0.00
Test of between-subgroup homogeneity: Q = 4.11, df = 1, p-value = 0.04

Diabetes

Forest Plot

- Effect size of each study
- Estimated overall effect size
- No-effect value
- Confidence interval of effect size
- Overall effect size value
- Estimated overall confidence interval

Country_Status	ID	Effect Size	Lower	Upper	p-value
HIC	de Bruijn (2015)	6.00	-2.50	14.50	0.17
	Thompson (2022)*	9.50	6.50	12.50	0.00
	Subgroup Overall	9.11	-9.23	27.45	0.10
LMIC	Wu (2022)	0.80	0.35	1.25	0.00
	Vergara (2022)	1.30	0.60	2.00	0.00
	Mukadam (2019)	1.60	1.10	2.10	0.00
	Mukadam (2019)	1.70	1.10	2.30	0.00
	Bobrow (2021)	2.00	1.00	3.00	0.00
	Suemoto (2023)	3.10	2.55	3.65	0.00
	Mukadam (2019)	3.20	2.90	3.50	0.00
	Vergara (2022)	4.90	2.45	7.35	0.00
	Subgroup Overall	2.13	1.18	3.08	0.00
Overall		2.87	1.16	4.58	0.00

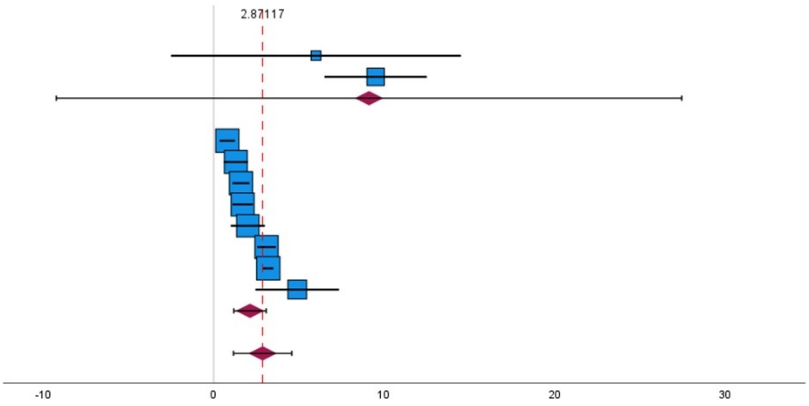

Model: Random-effects model
Heterogeneity: Tau-squared = 4.07, H-squared = 41.15, I-squared = 0.98
Homogeneity: Q = 132.11, df = 9, p-value = 0.00
Test of overall effect size: t = 3.80, df = 9, p-value = 0.00
Test of between-subgroup homogeneity: Q = 21.93, df = 1, p-value = 0.00

Appendix 14 Funnel plots and Egger's regression test results to assess publication bias of the individual risk factors (unweighted and weighted)

Key PAF=Population attributable fraction

Unweighted

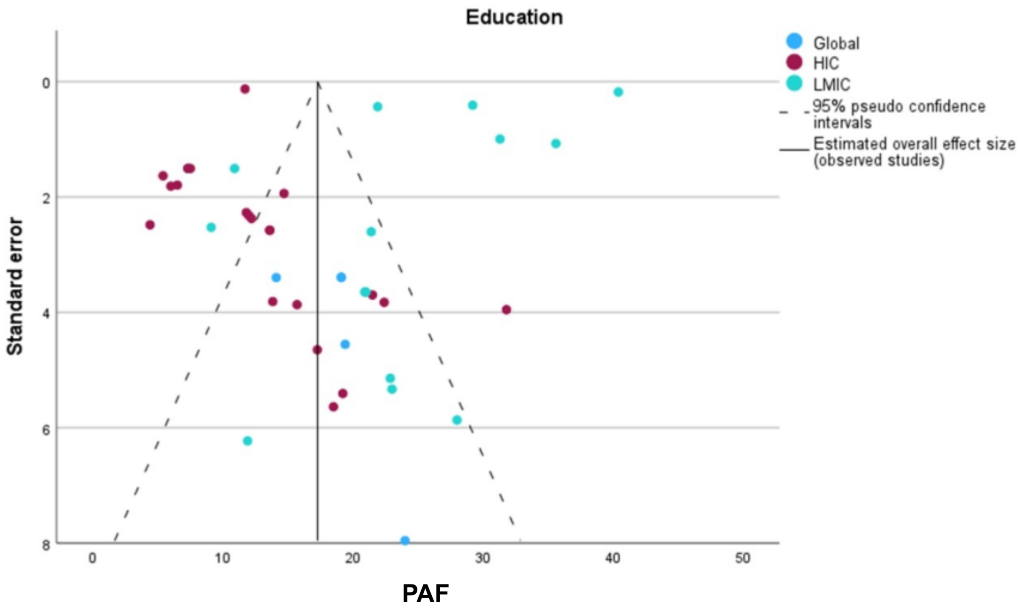

Egger's Regression-Based Test[a]

	Parameter	Coefficient	Std. Error	t	Sig. (2-tailed)	95% Confidence Interval Lower	Upper
Global	(Intercept)	2.461	2.5635	.960	.391	-4.656	9.579
	SE[b]	3.818	.8940	4.271	.013	1.336	6.300
HIC	(Intercept)	4.487	2.3933	1.875	.076	-.522	9.497
	SE[b]	3.197	.8608	3.714	.001	1.396	4.999
LMIC	(Intercept)	28.461	3.8067	7.477	<.001	20.167	36.755
	SE[b]	-1.844	1.1842	-1.558	.145	-4.425	.736
Overall	(Intercept)	16.362	2.6947	6.072	<.001	10.911	21.813
	SE[b]	.331	.8288	.399	.692	-1.345	2.008

a. Random effects meta-regression with the Truncated Knapp-Hartung SE adjustment.

b. Standard error of effect size

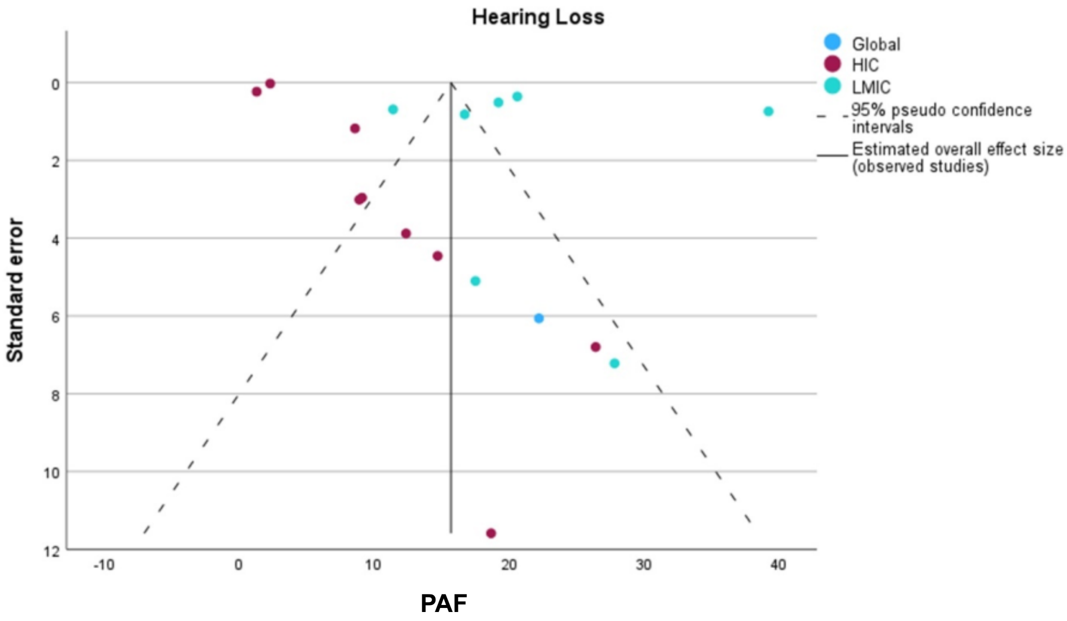

Egger's Regression-Based Test[a,b]

	Parameter	Coefficient	Std. Error	t	Sig. (2-tailed)	95% Confidence Interval Lower	95% Confidence Interval Upper
HIC	(Intercept)	2.375	1.1566	2.053	.079	-.360	5.110
	SE[c]	2.592	.5322	4.871	.002	1.334	3.851
LMIC	(Intercept)	20.881	4.9899	4.185	.009	8.054	33.708
	SE[c]	.382	1.6794	.227	.829	-3.935	4.699
Overall	(Intercept)	13.038	3.4677	3.760	.002	5.647	20.430
	SE[c]	.994	.9093	1.093	.292	-.944	2.932

a. Random effects meta-regression with the Truncated Knapp-Hartung SE adjustment.

b. Regression Based Test cannot be computed for subgroup(s) Global_Status = Global.

c. Standard error of effect size

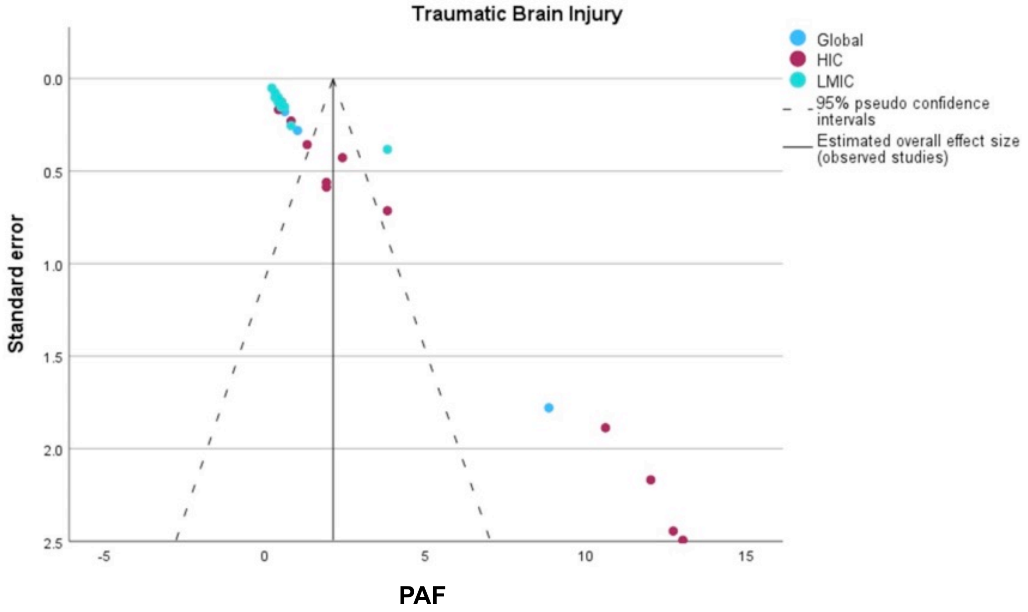

Egger's Regression-Based Test[a]

	Parameter	Coefficient	Std. Error	t	Sig. (2-tailed)	95% Confidence Interval Lower	Upper
Global	(Intercept)	-.329	.2790	-1.179	.448	-3.874	3.216
	SE[b]	5.027	1.0731	4.685	.134	-8.607	18.662
HIC	(Intercept)	-.237	.1057	-2.237	.047	-.469	-.004
	SE[b]	5.035	.4141	12.159	<.001	4.123	5.946
LMIC	(Intercept)	-.431	.1914	-2.253	.042	-.845	-.018
	SE[b]	7.152	1.3847	5.165	<.001	4.160	10.143
Overall	(Intercept)	-.159	.0633	-2.503	.018	-.288	-.029
	SE[b]	4.925	.3988	12.349	<.001	4.109	5.741

a. Random effects meta-regression with the Truncated Knapp-Hartung SE adjustment.

b. Standard error of effect size

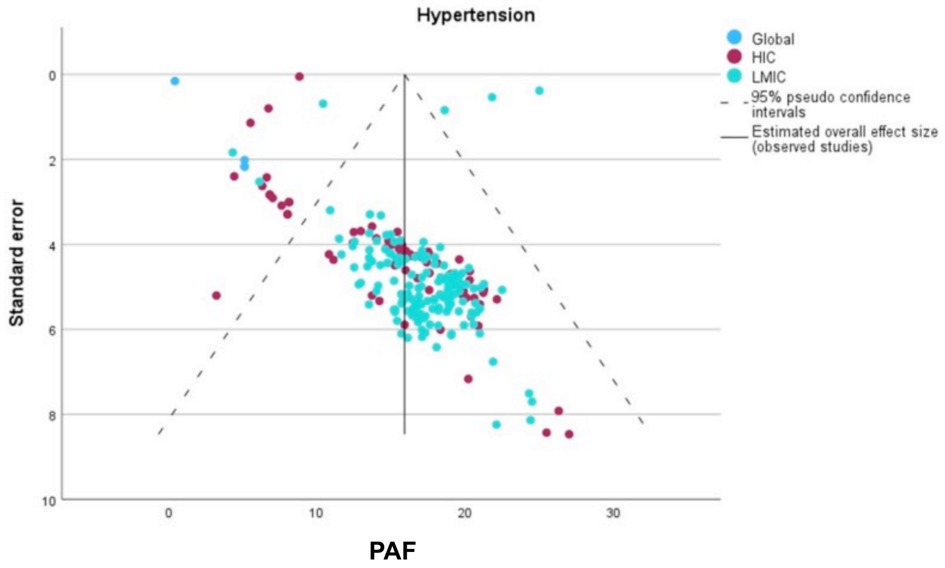

Egger's Regression-Based Test[a]

	Parameter	Coefficient	Std. Error	t	Sig. (2-tailed)	95% Confidence Interval Lower	Upper
Global	(Intercept)	.022	.1967	.110	.923	-.825	.868
	SE[b]	2.401	.6290	3.817	.062	-.305	5.108
HIC	(Intercept)	4.219	1.0035	4.205	<.001	2.220	6.219
	SE[b]	2.460	.2705	9.096	<.001	1.921	2.999
LMIC	(Intercept)	20.108	.6215	32.354	<.001	18.880	21.336
	SE[b]	-.732	.1939	-3.776	<.001	-1.116	-.349
Overall	(Intercept)	6.097	2.3106	2.639	.009	1.544	10.650
	SE[b]	2.174	.4895	4.440	<.001	1.209	3.138

a. Random effects meta-regression with the Truncated Knapp-Hartung SE adjustment.

b. Standard error of effect size

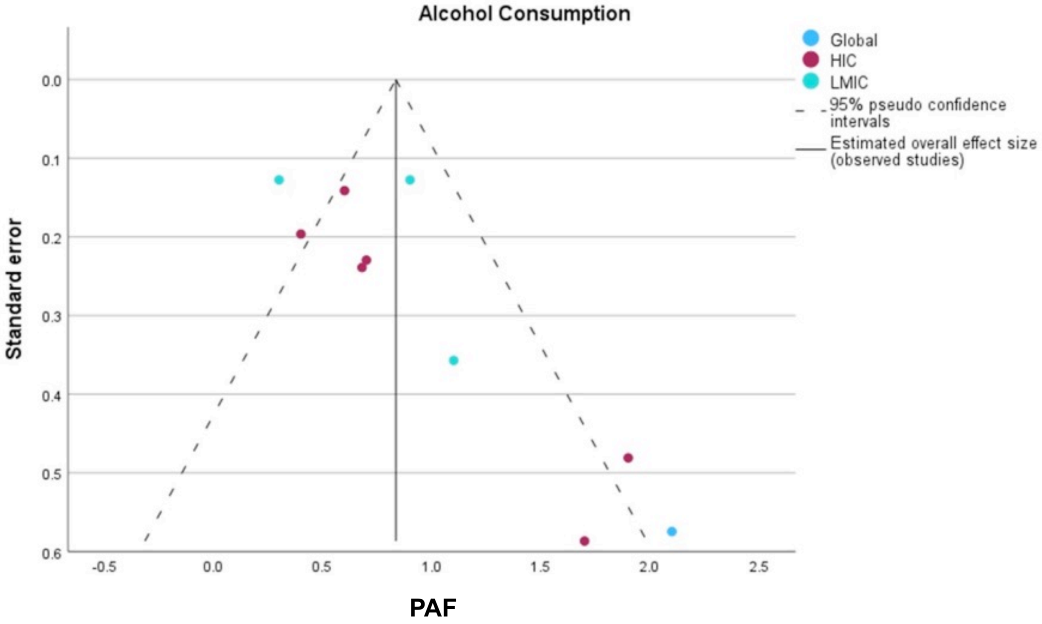

Egger's Regression-Based Test[a,b]

	Parameter	Coefficient	Std. Error	t	Sig. (2-tailed)	95% Confidence Interval Lower	Upper
HIC	(Intercept)	.040	.2349	.171	.873	-.612	.692
	SE[c]	3.002	1.0281	2.920	.043	.148	5.857
LMIC	(Intercept)	.322	.5547	.581	.665	-6.726	7.370
	SE[c]	2.178	2.6895	.810	.567	-31.995	36.351
Overall	(Intercept)	.108	.2045	.527	.612	-.364	.579
	SE[c]	2.989	.8348	3.581	.007	1.064	4.914

a. Random effects meta-regression with the Truncated Knapp-Hartung SE adjustment.

b. Regression Based Test cannot be computed for subgroup(s) Global_Status = Global.

c. Standard error of effect size

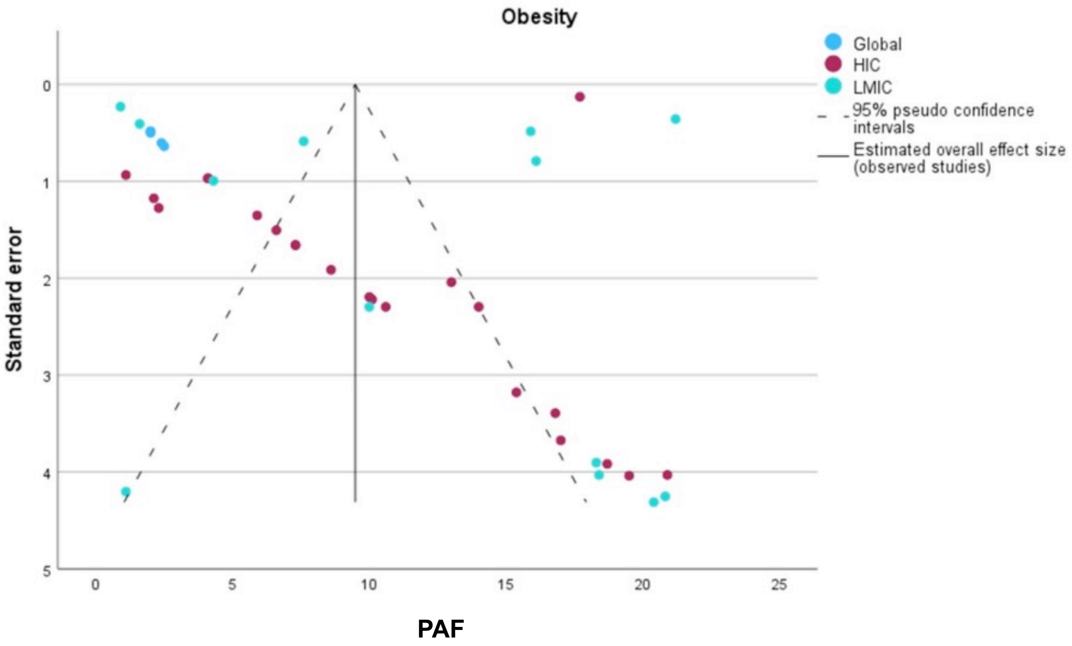

Egger's Regression-Based Test[a]

	Parameter	Coefficient	Std. Error	t	Sig. (2-tailed)	95% Confidence Interval Lower	Upper
Global	(Intercept)	.342	2.0929	.163	.881	-6.318	7.002
	SE[b]	3.390	3.9399	.861	.453	-9.148	15.929
HIC	(Intercept)	2.308	2.2190	1.040	.311	-2.321	6.937
	SE[b]	3.835	1.0126	3.788	.001	1.723	5.948
LMIC	(Intercept)	8.617	3.2602	2.643	.023	1.441	15.793
	SE[b]	1.658	1.3065	1.269	.231	-1.218	4.533
Overall	(Intercept)	4.197	1.5339	2.736	.009	1.092	7.302
	SE[b]	3.019	.7189	4.199	<.001	1.564	4.474

a. Random effects meta-regression with the Truncated Knapp-Hartung SE adjustment.

b. Standard error of effect size

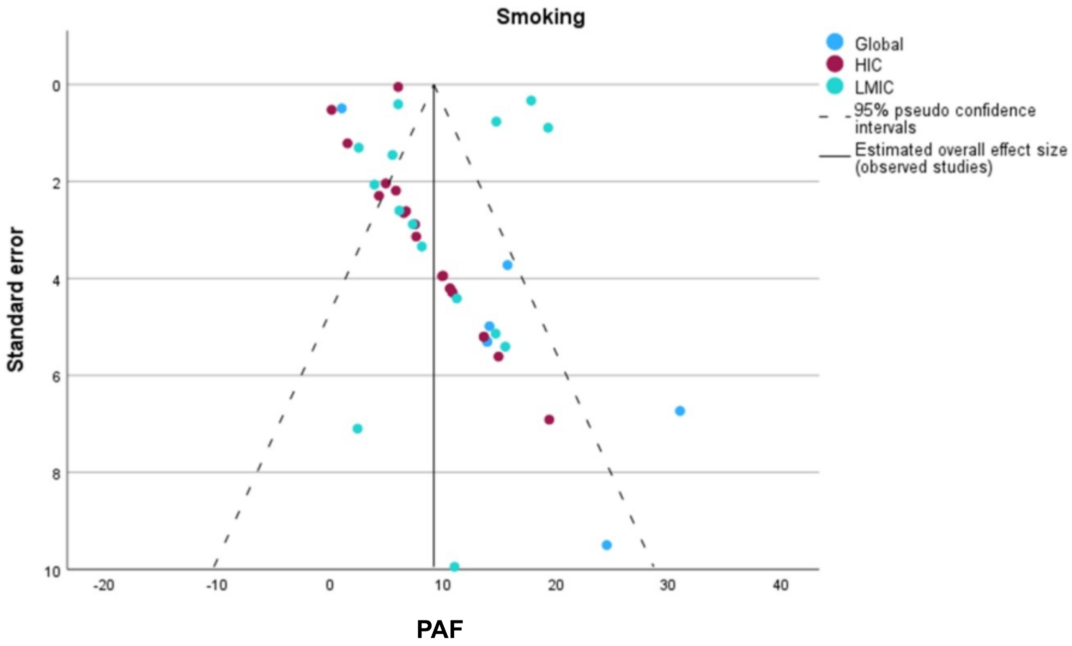

Egger's Regression-Based Test[a]

	Parameter	Coefficient	Std. Error	t	Sig. (2-tailed)	95% Confidence Interval Lower	Upper
Global	(Intercept)	-.627	.5828	-1.076	.331	-2.126	.871
	SE[b]	3.351	.4592	7.299	<.001	2.171	4.532
HIC	(Intercept)	1.449	1.3146	1.102	.285	-1.313	4.211
	SE[b]	2.075	.4765	4.355	<.001	1.074	3.076
LMIC	(Intercept)	10.442	2.5168	4.149	.001	5.005	15.879
	SE[b]	-.279	.7954	-.351	.731	-1.997	1.439
Overall	(Intercept)	5.238	1.5481	3.383	.002	2.109	8.367
	SE[b]	1.383	.4523	3.058	.004	.469	2.297

a. Random effects meta-regression with the Truncated Knapp-Hartung SE adjustment.

b. Standard error of effect size

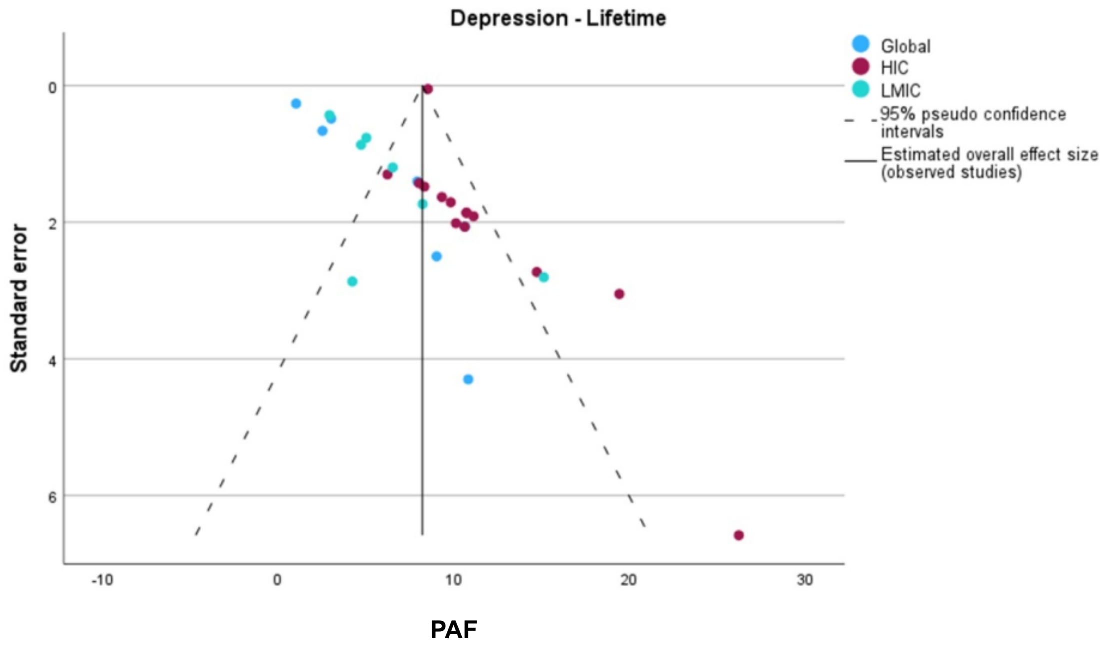

Egger's Regression-Based Test[a]

	Parameter	Coefficient	Std. Error	t	Sig. (2-tailed)	95% Confidence Interval Lower	95% Confidence Interval Upper
Global	(Intercept)	.498	.6850	.728	.499	-1.262	2.259
	SE[b]	3.861	.7603	5.078	.004	1.906	5.815
HIC	(Intercept)	5.910	1.2882	4.587	<.001	3.103	8.717
	SE[b]	2.541	.7196	3.532	.004	.973	4.109
LMIC	(Intercept)	1.693	.8354	2.026	.099	-.455	3.840
	SE[b]	3.427	.9799	3.497	.017	.908	5.945
Overall	(Intercept)	2.765	.9063	3.051	.005	.902	4.628
	SE[b]	3.427	.5450	6.287	<.001	2.306	4.547

a. Random effects meta-regression with the Truncated Knapp-Hartung SE adjustment.

b. Standard error of effect size

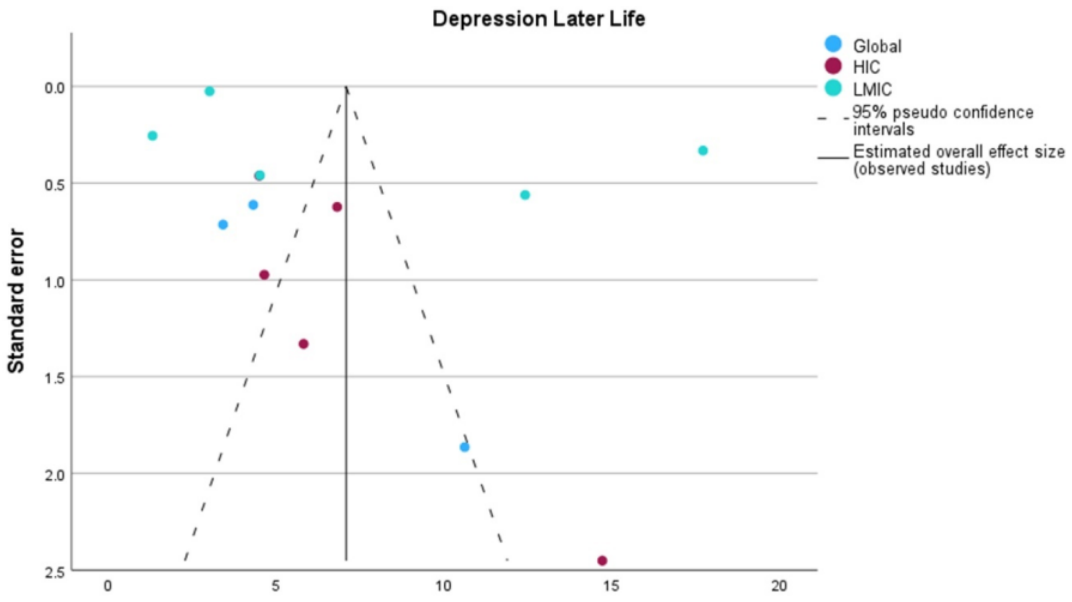

PAF

Egger's Regression-Based Test[a]

	Parameter	Coefficient	Std. Error	t	Sig. (2-tailed)	95% Confidence Interval Lower	Upper
Global	(Intercept)	.372	1.5710	.236	.852	-19.590	20.333
	SE[b]	5.360	1.7722	3.025	.203	-17.157	27.877
HIC	(Intercept)	2.398	1.8614	1.288	.288	-3.526	8.322
	SE[b]	4.080	1.6754	2.435	.093	-1.252	9.412
LMIC	(Intercept)	2.735	6.5604	.417	.705	-18.143	23.613
	SE[b]	15.452	17.5291	.882	.443	-40.333	71.237
Overall	(Intercept)	4.693	2.1488	2.184	.052	-.036	9.423
	SE[b]	3.036	2.1475	1.414	.185	-1.691	7.762

a. Random effects meta-regression with the Truncated Knapp-Hartung SE adjustment.

b. Standard error of effect size

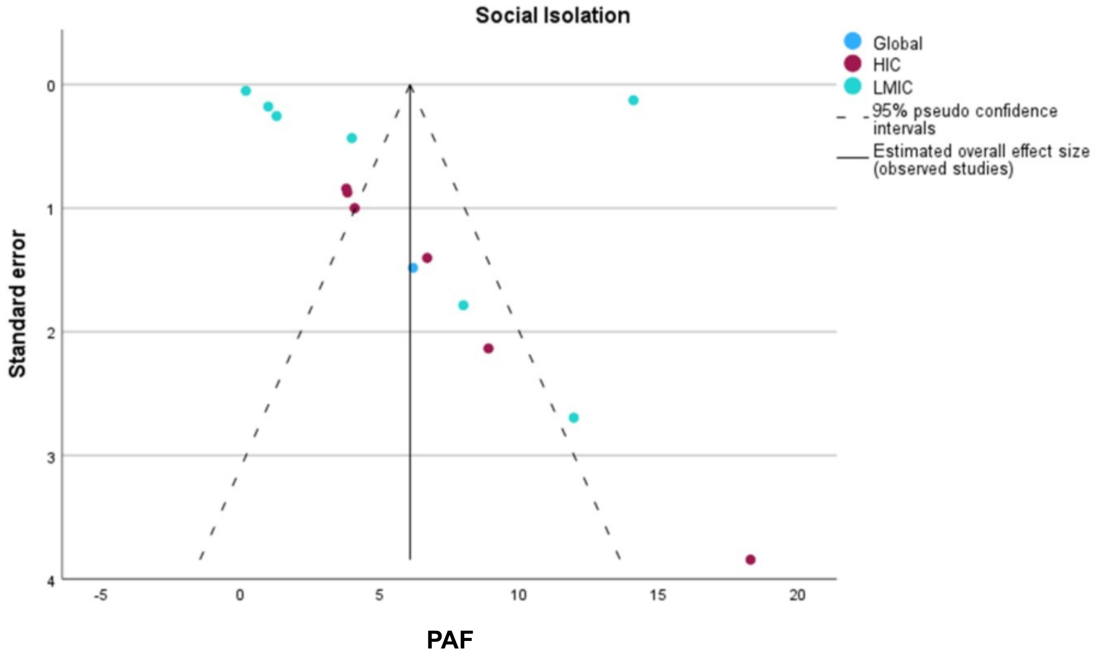

Effect Size Estimates for Subgroup Analysis

	Effect Size	Std. Error[a]	t	Sig. (2-tailed)	95% Confidence Interval Lower	Upper
Global[b]	6.191	1.4831	4.175	.	.	.
HIC	6.509	1.8888	3.446	.018	1.653	11.364
LMIC	5.589	2.1293	2.625	.039	.378	10.799
Overall	6.087	1.3208	4.608	<.001	3.233	8.940

a. Truncated Knapp-Hartung method is used for SE adjustment.

b. Some statistics cannot be computed because this subgroup contains a single record.

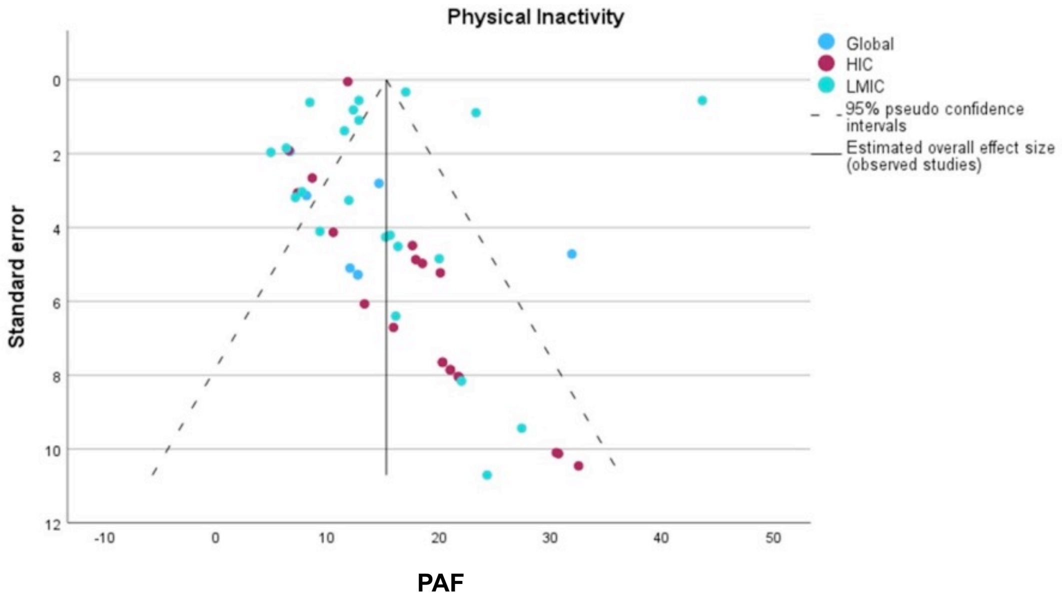

Egger's Regression-Based Test[a]

	Parameter	Coefficient	Std. Error	t	Sig. (2-tailed)	95% Confidence Interval Lower	Upper
Global	(Intercept)	3.768	9.4920	.397	.708	-20.632	28.168
	SE[b]	2.566	2.3443	1.095	.324	-3.460	8.592
HIC	(Intercept)	6.354	2.6082	2.436	.025	.875	11.834
	SE[b]	1.898	.5085	3.733	.002	.830	2.967
LMIC	(Intercept)	13.772	3.1224	4.411	<.001	7.259	20.285
	SE[b]	.473	.8231	.575	.572	-1.243	2.190
Overall	(Intercept)	11.052	2.1131	5.230	<.001	6.801	15.303
	SE[b]	1.124	.4613	2.436	.019	.196	2.052

a. Random effects meta-regression with the Truncated Knapp-Hartung SE adjustment.

b. Standard error of effect size

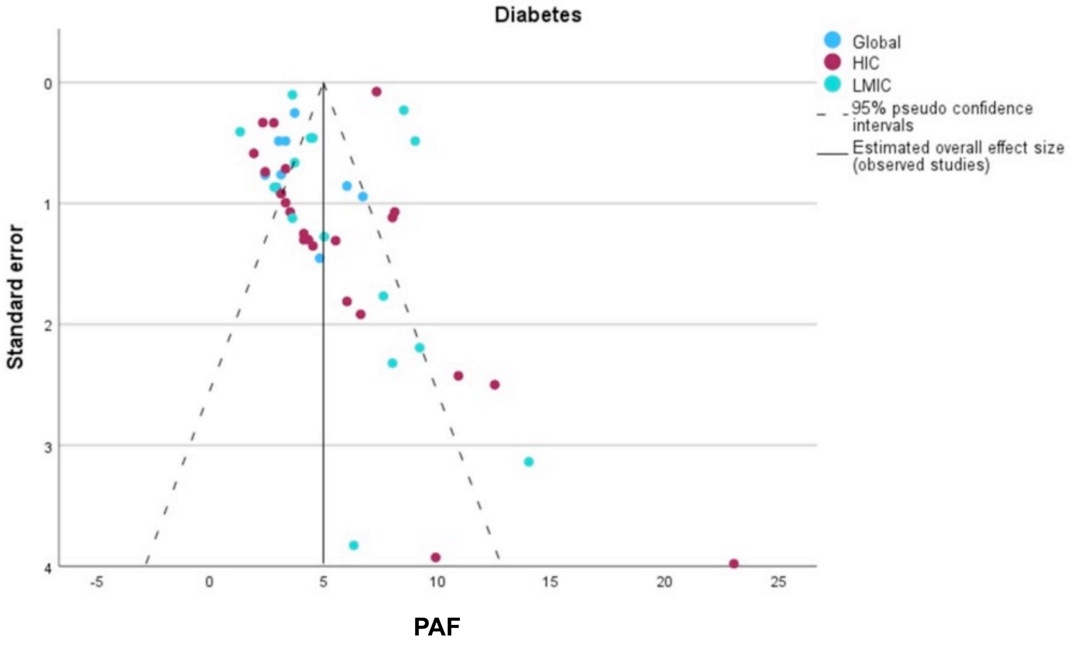

Egger's Regression-Based Test[a]

	Parameter	Coefficient	Std. Error	t	Sig. (2-tailed)	95% Confidence Interval Lower	95% Confidence Interval Upper
Global	(Intercept)	2.590	1.1706	2.213	.063	-.178	5.358
	SE[b]	1.822	1.5611	1.167	.281	-1.870	5.513
HIC	(Intercept)	1.543	.9133	1.689	.106	-.356	3.442
	SE[b]	3.164	.7020	4.507	<.001	1.704	4.624
LMIC	(Intercept)	3.958	1.0659	3.713	.003	1.655	6.260
	SE[b]	1.640	.8201	2.000	.067	-.132	3.412
Overall	(Intercept)	2.611	.5765	4.529	<.001	1.450	3.772
	SE[b]	2.397	.4880	4.912	<.001	1.414	3.380

a. Random effects meta-regression with the Truncated Knapp-Hartung SE adjustment.

b. Standard error of effect size

Weighted

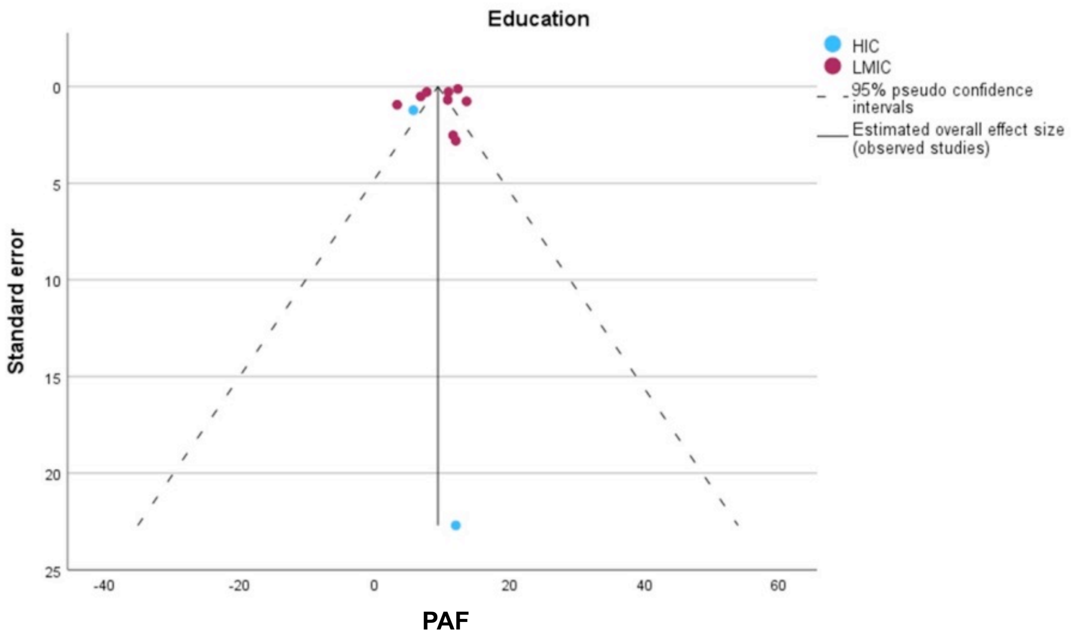

Egger's Regression-Based Test[a,b]

	Parameter	Coefficient	Std. Error	t	Sig. (2-tailed)	95% Confidence Interval	
						Lower	Upper
LMIC	(Intercept)	9.248	1.7058	5.421	<.001	5.214	13.281
	SE[c]	.596	1.4566	.409	.695	-2.848	4.040
Overall	(Intercept)	9.164	1.3538	6.769	<.001	6.101	12.226
	SE[c]	.201	.8448	.238	.817	-1.710	2.112

a. Random effects meta-regression with the Truncated Knapp-Hartung SE adjustment.

b. Regression Based Test cannot be computed for subgroup(s) Country_Status = HIC.

c. Standard error of effect size

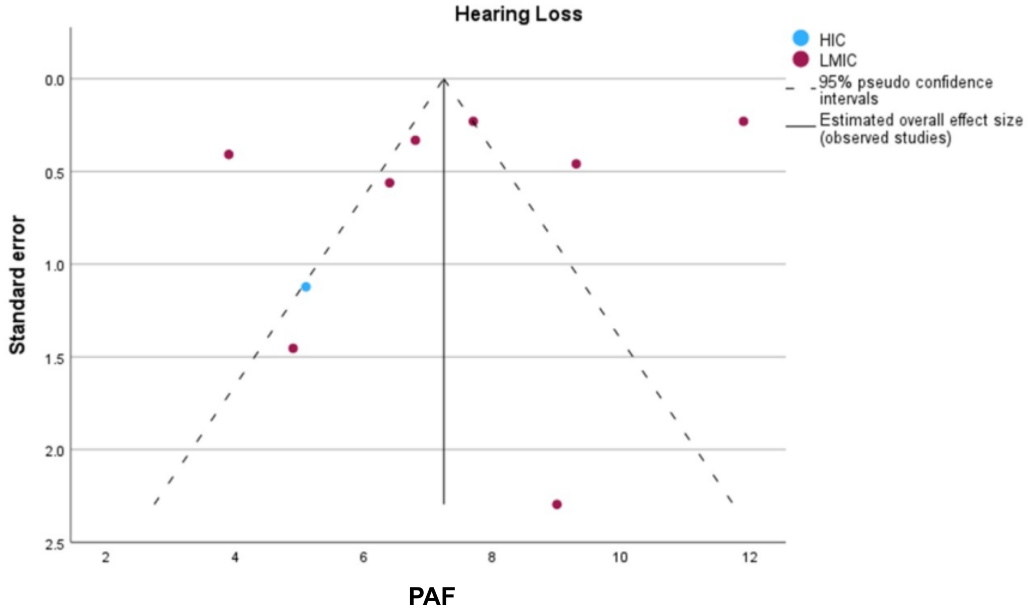

Egger's Regression-Based Test[a,b]

	Parameter	Coefficient	Std. Error	t	Sig. (2-tailed)	95% Confidence Interval Lower	Upper
LMIC	(Intercept)	7.802	1.4472	5.391	.002	4.261	11.343
	SE[c]	-.493	1.6524	-.299	.775	-4.537	3.550
Overall	(Intercept)	7.789	1.3897	5.605	<.001	4.503	11.075
	SE[c]	-.805	1.5493	-.520	.619	-4.469	2.858

a. Random effects meta-regression with the Truncated Knapp-Hartung SE adjustment.

b. Regression Based Test cannot be computed for subgroup(s) Country_Status = HIC.

c. Standard error of effect size

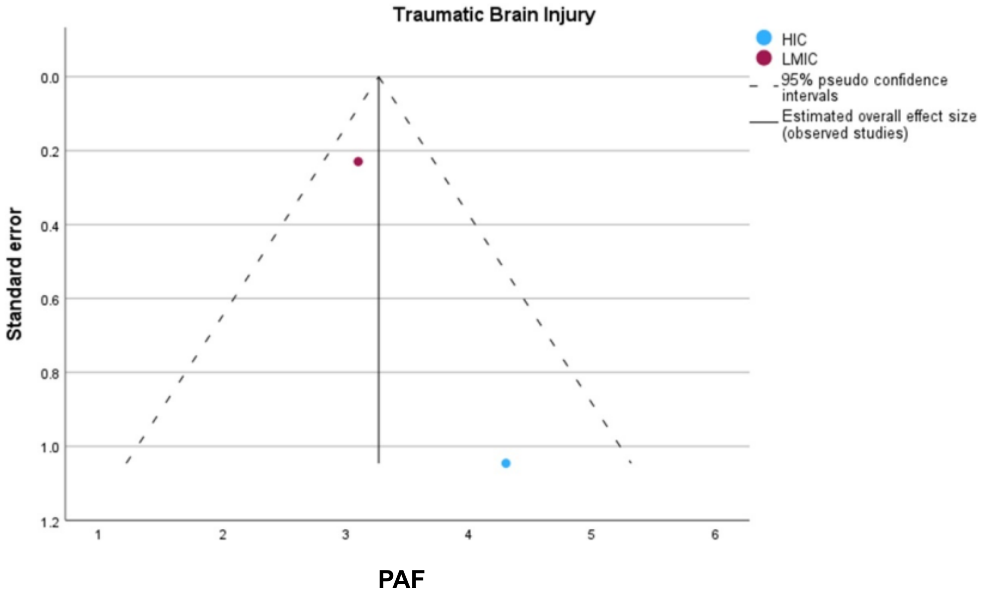

Note Egger's Regression-Based Test cannot be computed when the number of studies is less than or equal to two

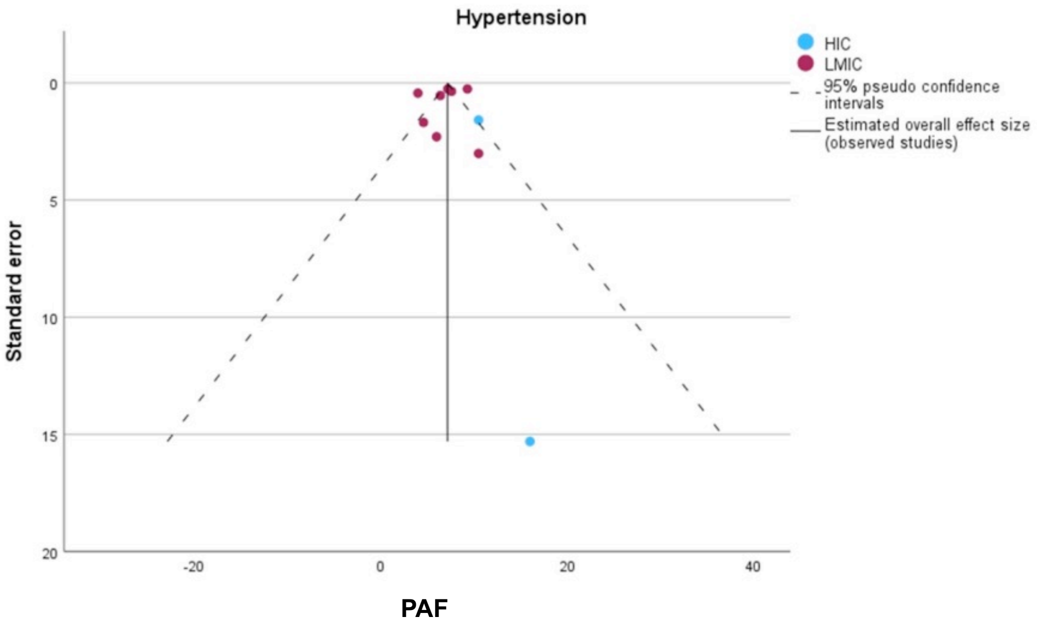

Egger's Regression-Based Test[a,b]

	Parameter	Coefficient	Std. Error	t	Sig. (2-tailed)	95% Confidence Interval	
						Lower	Upper
LMIC	(Intercept)	6.831	1.0567	6.464	<.001	4.245	9.417
	SE[c]	-.022	.9868	-.022	.983	-2.437	2.392
Overall	(Intercept)	6.739	.9811	6.868	<.001	4.476	9.001
	SE[c]	.498	.7154	.696	.506	-1.152	2.147

a. Random effects meta-regression with the Truncated Knapp-Hartung SE adjustment.

b. Regression Based Test cannot be computed for subgroup(s) Country_Status = HIC.

c. Standard error of effect size

Alcohol Consumption

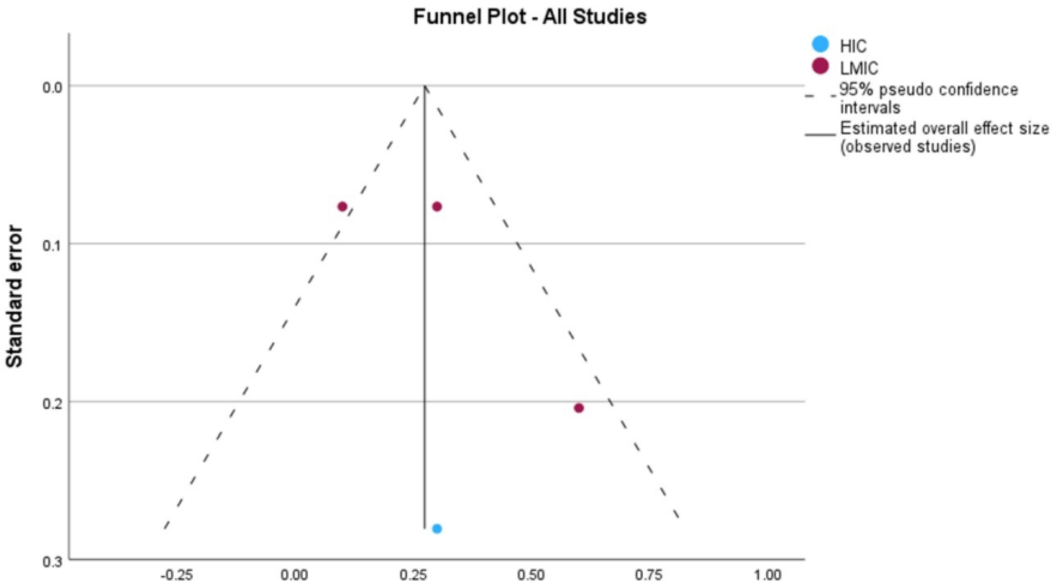

Egger's Regression-Based Test[a,b]

	Parameter	Coefficient	Std. Error	t	Sig. (2-tailed)	95% Confidence Interval Lower	95% Confidence Interval Upper
LMIC	(Intercept)	-.040	.2137	-.187	.882	-2.756	2.676
	SE[c]	3.136	2.0110	1.559	.363	-22.416	28.688
Overall	(Intercept)	.104	.1804	.576	.623	-.672	.880
	SE[c]	1.441	1.3695	1.052	.403	-4.451	7.333

a. Random effects meta-regression with the Truncated Knapp-Hartung SE adjustment.

b. Regression Based Test cannot be computed for subgroup(s) Country_Status = HIC.

c. Standard error of effect size

Obesity

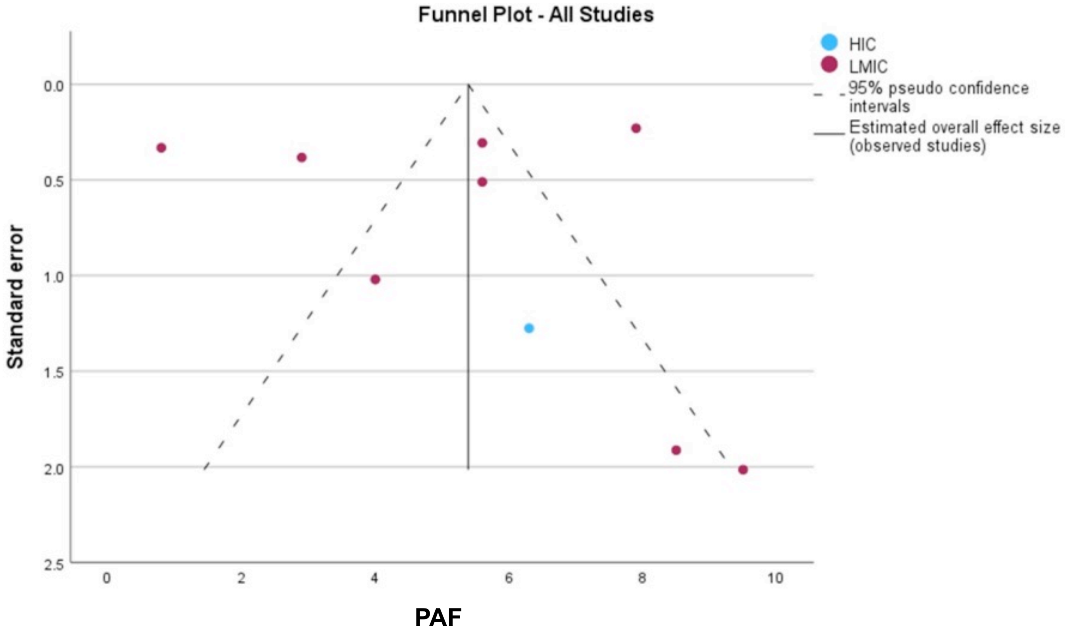

Egger's Regression-Based Test[a,b]

	Parameter	Coefficient	Std. Error	t	Sig. (2-tailed)	95% Confidence Interval Lower	Upper
LMIC	(Intercept)	3.565	1.4617	2.439	.051	-.012	7.142
	SE[c]	2.385	1.5347	1.554	.171	-1.371	6.140
Overall	(Intercept)	3.579	1.3758	2.601	.035	.325	6.832
	SE[c]	2.325	1.4064	1.653	.142	-1.001	5.650

a. Random effects meta-regression with the Truncated Knapp-Hartung SE adjustment.

b. Regression Based Test cannot be computed for subgroup(s) Country_Status = HIC.

c. Standard error of effect size

Smoking

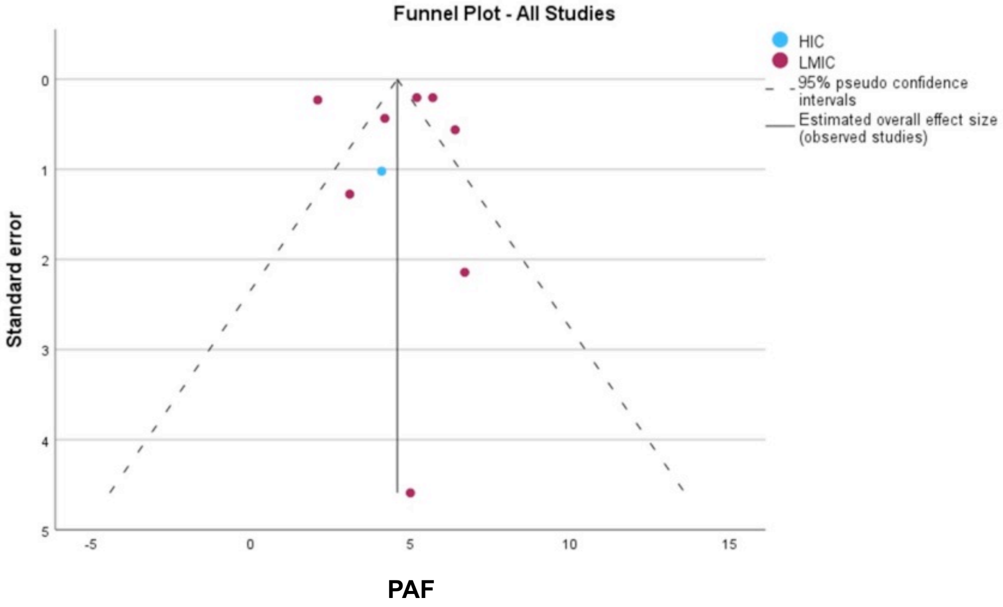

Egger's Regression-Based Test[a,b]

	Parameter	Coefficient	Std. Error	t	Sig. (2-tailed)	95% Confidence Interval Lower	95% Confidence Interval Upper
LMIC	(Intercept)	4.481	.8412	5.327	.002	2.423	6.540
	SE[c]	.287	.8730	.329	.753	-1.849	2.423
Overall	(Intercept)	4.445	.7909	5.621	<.001	2.575	6.316
	SE[c]	.231	.8391	.275	.791	-1.753	2.215

a. Random effects meta-regression with the Truncated Knapp-Hartung SE adjustment.

b. Regression Based Test cannot be computed for subgroup(s) Country_Status = HIC.

c. Standard error of effect size

Depression Lifetime

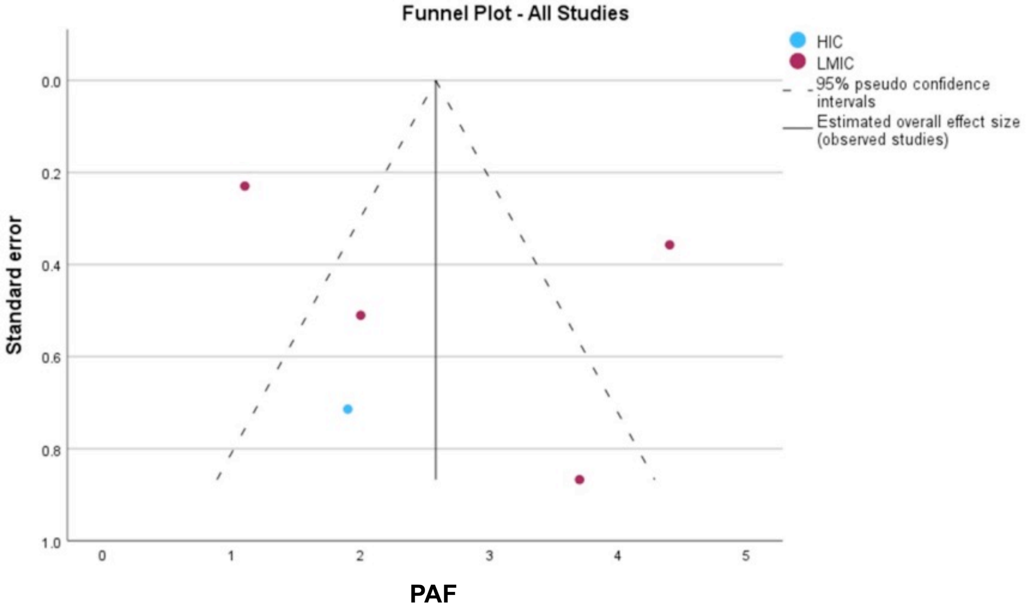

Egger's Regression-Based Test[a,b]

	Parameter	Coefficient	Std. Error	t	Sig. (2-tailed)	95% Confidence Interval Lower	Upper
LMIC	(Intercept)	1.590	1.9648	.809	.503	-6.864	10.044
	SE[c]	2.471	3.7439	.660	.577	-13.637	18.580
Overall	(Intercept)	1.851	1.7628	1.050	.371	-3.759	7.461
	SE[c]	1.438	3.1362	.458	.678	-8.543	11.419

a. Random effects meta-regression with the Truncated Knapp-Hartung SE adjustment.

b. Regression Based Test cannot be computed for subgroup(s) Country_Status = HIC.

c. Standard error of effect size

Depression Later Life

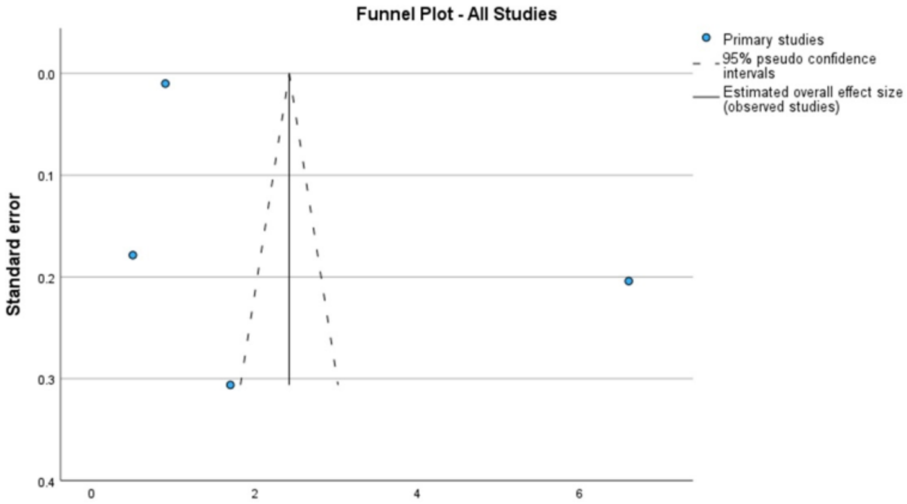

PAF

Egger's Regression-Based Test[a]

	Parameter	Coefficient	Std. Error	t	Sig. (2-tailed)	95% Confidence Interval	
						Lower	Upper
LMIC	(Intercept)	1.375	3.2094	.428	.710	-12.434	15.184
	SE[b]	6.015	15.7097	.383	.739	-61.578	73.608

a. Random effects meta-regression with the Truncated Knapp-Hartung SE adjustment.

b. Standard error of effect size

Social Isolation

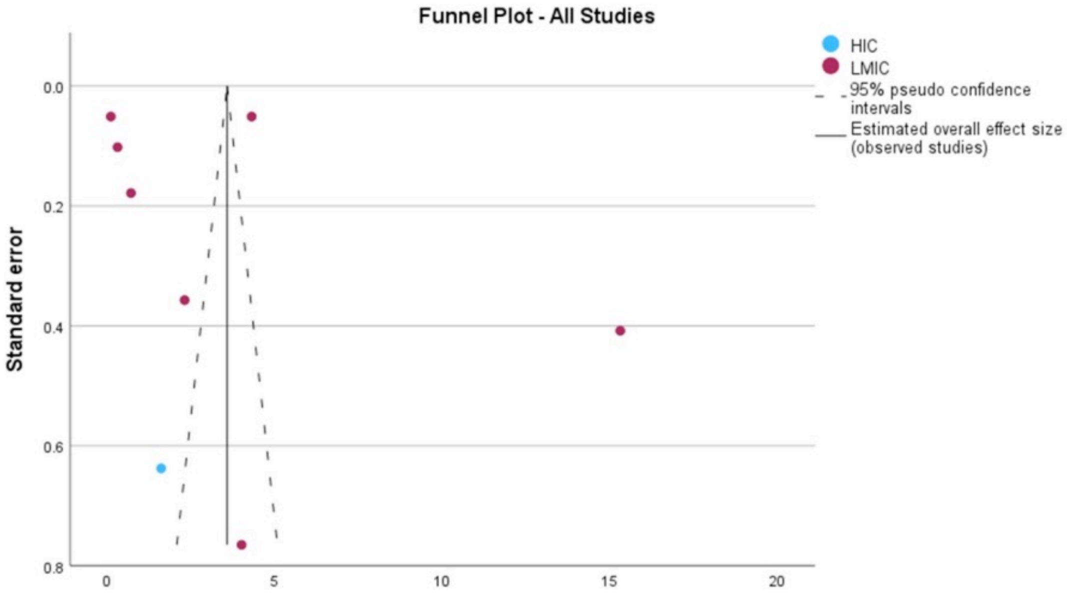

Egger's Regression-Based Test[a,b]

	Parameter	Coefficient	Std. Error	t	Sig. (2-tailed)	95% Confidence Interval Lower	95% Confidence Interval Upper
LMIC	(Intercept)	1.728	3.0884	.559	.600	-6.211	9.667
	SE[c]	7.805	8.5269	.915	.402	-14.114	29.724
Overall	(Intercept)	2.088	2.9496	.708	.506	-5.130	9.305
	SE[c]	4.675	7.2558	.644	.543	-13.079	22.429

a. Random effects meta-regression with the Truncated Knapp-Hartung SE adjustment.

b. Regression Based Test cannot be computed for subgroup(s) Country_Status = HIC.

c. Standard error of effect size

Physical Inactivity

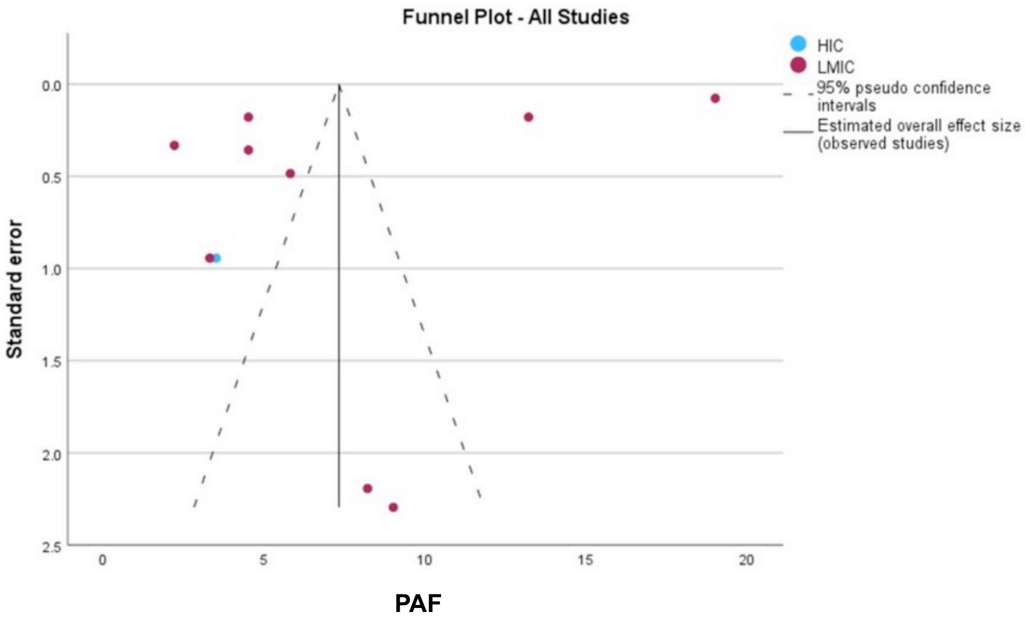

Egger's Regression-Based Test[a,b]

	Parameter	Coefficient	Std. Error	t	Sig. (2-tailed)	95% Confidence Interval Lower	95% Confidence Interval Upper
LMIC	(Intercept)	8.190	2.6894	3.045	.019	1.830	14.549
	SE[c]	-.614	2.4867	-.247	.812	-6.494	5.266
Overall	(Intercept)	7.884	2.5536	3.088	.015	1.996	13.773
	SE[c]	-.761	2.3971	-.317	.759	-6.288	4.767

a. Random effects meta-regression with the Truncated Knapp-Hartung SE adjustment.

b. Regression Based Test cannot be computed for subgroup(s) Country_Status = HIC.

c. Standard error of effect size

Diabetes

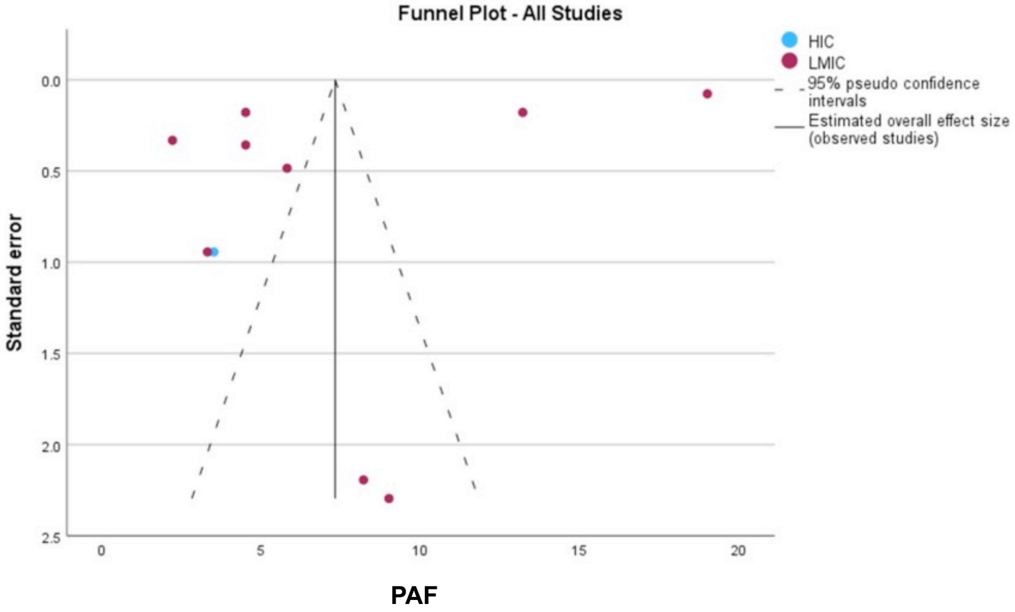

Egger's Regression-Based Test[a,b]

	Parameter	Coefficient	Std. Error	t	Sig. (2-tailed)	95% Confidence Interval Lower	95% Confidence Interval Upper
LMIC	(Intercept)	8.190	2.6894	3.045	.019	1.830	14.549
	SE[c]	-.614	2.4867	-.247	.812	-6.494	5.266
Overall	(Intercept)	7.884	2.5536	3.088	.015	1.996	13.773
	SE[c]	-.761	2.3971	-.317	.759	-6.288	4.767

a. Random effects meta-regression with the Truncated Knapp-Hartung SE adjustment.

b. Regression Based Test cannot be computed for subgroup(s) Country_Status = HIC.

c. Standard error of effect size

Appendix 15 PAF values (unweighted and weighted) associated with the Barnes (2011)[4] seven-factor model incorporating low education, mid-life hypertension, mid-life obesity, smoking, physical inactivity, depression, and diabetes

Reference	Location	Sample size	Outcome	PAF_{unw} (95%CI)	PAF_{w} (95%CI)
**Worldwide**					
Barnes (2011)[4]	Global	N/A	AD	50.7	Not reported
Norton (2014)[45]	Global	N/A	AD	49.4 (25.7–68.4)	28.2 (14.2–41.5)
**North America**					
Barnes (2011)[4]	USA	N/A	AD	54.1	Not reported
Norton (2014)[45]	USA	N/A	AD	52.7 (25.9–72.8)	30.6 (14.5–45.3)
**South America**					
Oliveira (2019)[46]	Brazil	Not reported	All-cause	55.3 (28.3–74.3)	32.3 (15.8–46.3)
**Australasia**					
Ashby-Mitchell (2017)[1]	Australia	Not clear	All-cause	57.0 (33.7-73.6)	48.4 (28.1-64.2)
Kotaki (2019)[30]	Japan	8,563	All-cause	50.5	29.6[#]
Liu (2020)[33]	China	RR Data source range 837-8,593	All-cause	55	Not reported
Woo (2014)[70]	Hong Kong	Not reported	AD	49.3	Not reported
**Africa**					
Oliverira (2019)[46]	Mozambique	Not reported	All-cause	44.0 (23.4–61.8)	24.4 (12.9–36.1)
**Europe**					
Luck (2016)[37]	Germany	Not reported	AD	Not reported	30.5 (13.9–45.4)
Mayer (2018)[40]	Italy	146,526	AD	56·8	45.2
Mayer (2018)[40]	Europe	N/A	VaD	66.8 (42.5-83.0)	37.8 (21.2-52.5)
Mayer (2018)[40]	Italy	146,526	VaD	65·9	53.1
Norton (2014)[45]	Europe	N/A	AD	54.0 (27.2–73.7)	31.4 (15.3–46.0)
Norton (2014)[45]	UK	N/A	AD	52.0 (25.6-71.9)	30.0 (14.3-44.4)
Oliverira (2019)[46]	Portugal	Not reported	All-cause	65.8 (36.2–83.4)	40.1 (20.7–55.4)

Key 95%CI=95% Confidence Interval; AD=Alzheimer's disease; N/A=Not applicable (as estimates are based on review findings); PAF_{unw}=Population Attributable Fraction Percent (unweighted); PAF_{w}=Population Attributable Fraction Percent (weighted for commonality between factors); RR=Relative risk; VaD=Vascular Dementia

Notes

PAF was adjusted for age (65–69, 70–74, 75–79, 80–84, or ≥ 85 years) and sex plus history of diabetes mellitus (present or absent), hypertension (history of hypertension or systolic blood pressure ≥ 140 mmHg and/or diastolic blood pressure ≥ 90 mmHg), body mass index (in kg/m2; < 30 or ≥ 30), time spent walking per day (< 30 or ≥ 30 min), Kessler 6-Item Psychological Distress Scale (K6) score (< 13 or ≥ 13), smoking status (former/never or current) and educational level (age at last school graduation; < 16 or ≥ 16 years). PAF=35.0 adjusted for age and sex only.

Appendix 16 Funnel plots and Egger's regression test results to assess publication bias in the analysis of the seven-factor model proposed by Barnes et al (unweighted and weighted)

Publication bias for unweighted estimates for the seven-factor model proposed by Barnes et al

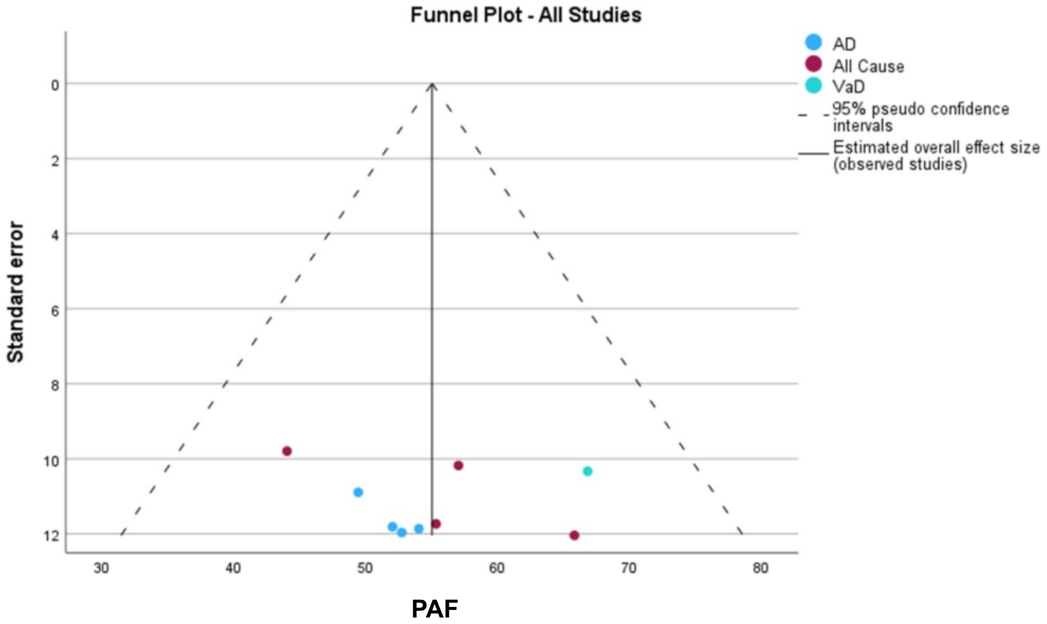

Egger's Regression-Based Test[a,b,c]

	Parameter	Coefficient	Std. Error	t	Sig. (2-tailed)	95% Confidence Interval Lower	95% Confidence Interval Upper
AD	(Intercept)	10.967	150.4800	.073	.949	-636.496	658.430
	SE[d]	3.530	12.9642	.272	.811	-52.251	59.310
All Cause	(Intercept)	-13.603	61.2353	-.222	.845	-277.077	249.871
	SE[d]	6.321	5.6649	1.116	.381	-18.053	30.695
Overall	(Intercept)	35.754	47.8563	.747	.479	-77.408	148.916
	SE[d]	1.742	4.3186	.403	.699	-8.470	11.954

a. Type_Analysis = Unweighted
b. Random effects meta-regression with the Truncated Knapp-Hartung SE adjustment.
c. Regression Based Test cannot be computed for subgroup(s) Outcome = VaD.
d. Standard error of effect size

Publication bias for weighted estimates for the seven-factor model proposed by Barnes et al

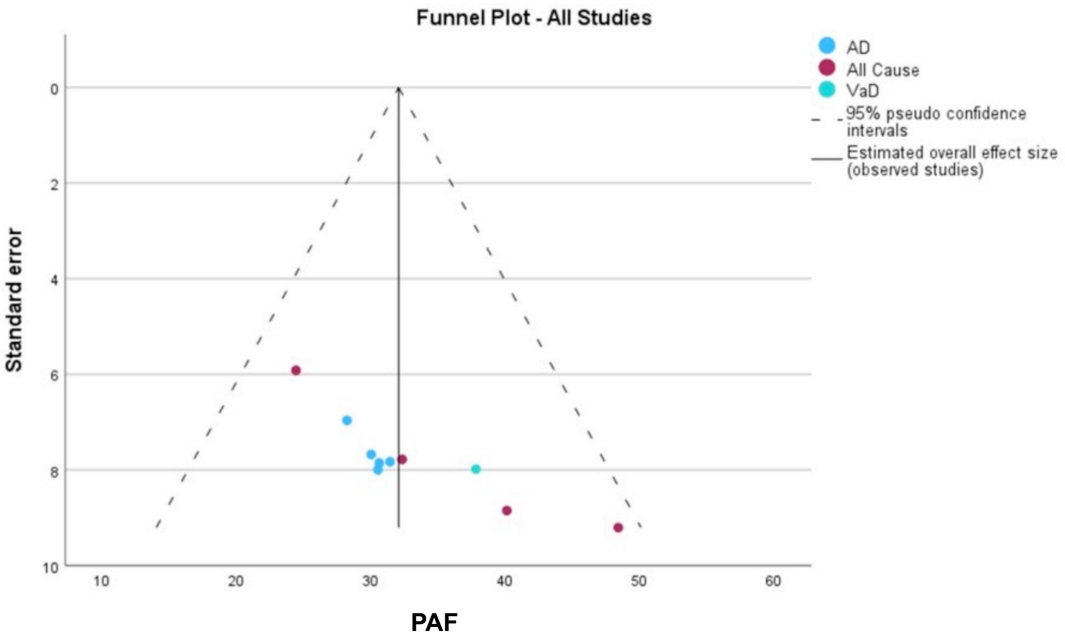

Egger's Regression-Based Test[a,b,c]

	Parameter	Coefficient	Std. Error	t	Sig. (2-tailed)	95% Confidence Interval Lower	Upper
AD	(Intercept)	9.553	67.4720	.142	.896	-205.173	224.279
	SE[d]	2.685	8.8337	.304	.781	-25.427	30.798
All Cause	(Intercept)	-13.603	20.9941	-.648	.583	-103.933	76.728
	SE[d]	6.280	2.7697	2.267	.152	-5.637	18.197
Overall	(Intercept)	-12.936	19.9447	-.649	.535	-58.929	33.056
	SE[d]	5.926	2.6082	2.272	.053	-.089	11.940

a. Type_Analysis = Weighted

b. Random effects meta-regression with the Truncated Knapp-Hartung SE adjustment.

c. Regression Based Test cannot be computed for subgroup(s) Outcome = VaD.

d. Standard error of effect size

Appendix 17 Combined PAF values (unweighted and weighted) stratified by ethnicity

Reference	Country	Sample Size	Outcome	Factor Number: Factors	Group	PAF_{unw} (95%CI)	PAF_{w} (95%CI)
Smith (2023)[59]	USA	2,035	All-cause	1: Hearing loss	White, non-Hispanic	20.6 (3.6-36.2)	Not applicable
		188			Black, non-Hispanic	<0	Not applicable
Ehrlich (2022)[15]	USA	Not clear	All-cause	12: Education, hearing loss, TBI, hypertension, alcohol, obesity, smoking, depression, social isolation, physical inactivity, diabetes & vision impairment	White, non-Hispanic	NR	60.6
		Not clear			Black, non-Hispanic	NR	68.6
		Not clear			Hispanic, any race	NR	69.3
Lee (2022)[32]	USA	Not reported	All-cause	12: Education, hearing loss, TBI, hypertension, alcohol, obesity, smoking, depression, social isolation, physical inactivity, diabetes & air pollution	White, non-Hispanic	NR	39.4 (22.7-55.9)
		Not reported			Black, non-Hispanic	NR	45.6 (25.7-60.5)
		Not reported			Hispanic	NR	46.7 (27.3-61.5)
		Not reported			Asian, non-Hispanic	NR	35.8 (19.5-49.9)
Nianogo (2022)[44]	USA	294,394	All-cause	8: Education, hearing loss, midlife hypertension, midlife obesity, current smoking, depression, physical inactivity & diabetes	White	NR	28.5 (28.1-28.9)
		29,956			Black	NR	39.8 (38.6-41.0)
		28,042			Hispanic	NR	34.2 (33.0-35.3)
		6,671			Native American and Alaska Native	NR	39.2 (36.1-42.0)
		8,043			Asian		15.8 (13.8-17.8)
Weiss (2021)[69]	USA	5,771	All-cause	3: Socio-economic resources, lifestyle characteristics and medical conditions	White, non-Hispanic (Men)	45.4 (35.5-53.8)	NR
		7,371			White, non-Hispanic (Women)	41.0 (33.0-47.9)	NR
		798			Black, non-Hispanic (Men)	78.7 (67.2-86.2)	NR
		1,305			Black, non-Hispanic (Women)	64.1 (51.9-73.2)	NR
		424			Hispanic (Men)	78.2 (57.0-89.0)	NR
		565			Hispanic (Women)	90.3 (79.7-95.4)	NR
MacDonald (2015)[39]	Canada	Not reported	AD	6: Education, midlife hypertension, midlife obesity, smoking, physical inactivity & diabetes	Non-indigenous	67.1	NR
		As above			Indigenous (Total)	76.1	NR
		As above			Indigenous (On-reserve only)	79.6	NR
		As above			Indigenous (Off-reserve only)	74.9	NR
Ma'u (2021)[38]	New Zealand	Not reported		6: Education, midlife hypertension, midlife obesity, smoking, physical inactivity & diabetes	European	NR	47.6
		As above			Māori	NR	51.4
		As above			Pacific Peoples	NR	50.8
		As above			Asian	NR	40.8

Reference	Country	Sample Size	Outcome	Factor Number: Factors	Group	PAF_{unw} (95%CI)	PAF_{w} (95%CI)
Thompson (2022)[63]	Australia	371	All-cause	11: Education, midlife hearing loss, midlife TBI, midlife hypertension, midlife alcohol, midlife obesity, later life smoking, later life depression, later life social isolation, later life physical inactivity & later life diabetes	First Nations peoples, including Torres Strait Islander and Aboriginal peoples in Far North Queensland*	75.4	52.8 (47.8–57.9)
		As above		12: Education, midlife hearing loss, midlife TBI, midlife hypertension, midlife alcohol, midlife obesity, later life smoking, later life depression, later life social isolation, later life physical inactivity, later life diabetes & chronic kidney disease	As above	77.9	52.2
Borelli (2023)[8]	Brazil	3,590	All-cause	10: Education, hearing loss, hypertension, alcohol, obesity, smoking, depression, social contact, physical inactivity & diabetes	White	NR	49.2
		887			Black	NR	50.0
		4,283			Brown	NR	50.1
		220			Indigenous	NR	38.9
		90			Asian	NR	41.2
Suemoto (2023)[61]	Brazil	4,047	All-cause	12: Education, hearing loss, TBI, midlife, hypertension, alcohol, midlife obesity, later life smoking, later life depression, later life social isolation, later life physical inactivity, later life diabetes & air pollution	White	76.7 (75.3-78.1)	47.9 (46.3-49.6)
		5,365			Black	78.0 (76.9-79.1)	46.9 (45.5-48.3)

Notes

*No comparable data for the non-indigenous population. Ashby-Mitchell (2017) reported PAF estimates for the total Australian population based on seven risk factors including low education, mid-life obesity, mid-life hypertension, physical inactivity, smoking, depression, and diabetes.

Key 95%CI=95% Confidence Interval; AD=Alzheimer's disease; PAF_{unw}= Population Attributable Fraction Percent (unweighted); PAF_{w}=Population Attributable Fraction Percent (weighted for commonality between factors); NR=Not reported; TBI=Traumatic Brain Injury

Appendix 18 Individual PAF values for risk factors reported in the Lancet Commission[35] stratified by ethnicity

Author (year)	Country	Ethnicity	Outcome	Education	Hearing Loss	TBI	Hypertension	Alcohol	Obesity	Smoking	DEP (Lifetime)	DEP (Later-life)	Social Isolation	Physical Inactivity	Diabetes	Air Pollution
**Unweighted**																
Thompson (2022)[63]	AUS	First Nations people including Torres Strait Islander and Aboriginal people	All	13.8	12.4	10.6	25.5	0.7	15.4	10.0	4.6		3.8	8.6	23.0	
Ma'u (2021)[38]	NZ	Total population	All	15.7	26.4	12.7	16.7	1.9	18.7	7.5		14.7	18.3	17.6	5.5	6.7
MacDonald (2015)[39]	Canada	Indigenous	AD	22.4 (14.6, 29.6)			14.2 (4.2, 25.1)		16.8 (10.3, 23.6)	19.4 (5.8, 32.9)				32.5 (10.1, 51.1)	6.0 (2.6, 9.7)	
MacDonald (2015)[39]	Canada	Non-Indigenous	AD	12.0 (7.5, 16.6)			15.9 (4.7, 27.8)		10.6 (6.3, 15.3)	9.9 (2.7, 18.2)				30.5 (9.2, 48.8)	4.1 (1.7, 6.8)	
MacDonald (2015)[39]	Canada	Indigenous (On-reserve only)	AD	28.0 (18.7, 36.2)			11.7 (3.4, 21.3)		19.4 (12.0, 27.0)	25.2 (7.9, 40.6)				33.9 (10.6, 57.2)	5.9 (2.5, 9.7)	
MacDonald (2015)[39]	Canada	Indigenous (Off-reserve only)	AD	20.5 (13.3, 27.3)			14.9 (4.4, 26.2)		16.0 (9.7, 22.6)	17.5 (5.1, 30.1)				32.1 (9.9, 50.7)	6.0 (2.6, 9.8)	
Weiss (2021)[69]	USA	White, non-Hispanic (men)	All	20.6 (15.9, 25.0)	4.3 (-1.0, 9.3)		-1.2 (-8.1, 5.3)	1.5 (-0.1, 3.1)	-1.2 (-5.2, 2.7)	3.6 (0.7, 6.5)				4.7 (-2.2, 11.1)	6.1 (3.7, 8.4)	
Weiss (2021)[69]	USA	White, non-Hispanic (women)	All	12.6 (9.2, 15.8)	1.3 (-1.3, 3.8)		3.8 (-1.7, 9.0)	-0.5 (-1.7, 0.7)	-1.0 (-4.0, 1.8)	2.6 (1.0, 4.2)				3.9 (-3.1, 10.5)	4.0 (2.5, 5.5)	
Weiss (2021)[69]	USA	Black, non-Hispanic (men)	All	15.8 (-15.9, 38.9)	4.0 (-3.0, 10.6)		8.0 (-8.9, 22.3)	5.8 (2.6, 8.9)	-3.2 (-13.9, 6.5)	12.4 (4.7, 19.5)				−4.5 (-26.8, 13.9)	5.6 (-0.3, 11.2)	
Weiss (2021)[69]	USA	Black, non-Hispanic (women)	All	26.3 (11.5, 38.5)	-1.2 (-6.6, 3.9)		5.7 (-9.9, 19.1)	0.2 (-1.0, 1.4)	5.3 (-4.1, 13.8)	2.0 (-1.8, 5.7)				−7.2 (-25.4, 8.4)	5.2 (0.4, 9.8)	
Weiss (2021)[69]	USA	Hispanic (men)	All	−6.8 (-82.6, 37.5)	4.0 (-8.2, 14.9)		-11.7 (-40.1, 11.0)	-1.8 (-9.5, 5.3)	-17.6 (-43.0, 3.2)	3.6 (-6.2, 12.5)				−20.2 (-76.8, 18.3)	21.4 (13.3, 28.8)	
Weiss (2021)[69]	USA	Hispanic (women)	All	59.9 (34.2, 75.5)	2.6 (-6.2, 10.7)		-7.1 (-30.6, 12.2)	-0.1 (-0.8, 0.6)	3.9 (-8.9, 15.3)	4.2 (0.4, 7.9)				26.8 (3.2, 44.7)	8.4 (0.9, 15.3)	
Ehrlich (2022)[15]	USA	Total population	All	6.5	14.7	13.0	26.3	0.6	19.5	6.7	19.4		4.1	6.5	10.9	
Ehrlich (2022)[15]	USA	Black, non-Hispanic	All	11.2	13.2	14.8	31.4	0.29	23.1	10	18.2		7.3	8.6	14.3	
Ehrlich (2022)[15]	USA	Hispanic, any race	All	21.1	19.1	8.8	26.2	0.42	21.4	7.2	21.4		7.4	7.4	16.4	
Ehrlich (2022)[15]	USA	White, non-Hispanic	All	5.0	14.9	12.5	24.7	0.61	19	6.1	19.4		3.5	6.3	9.9	
Lee (2022)[32]	USA	Total population	All	6.0 (2.7, 9.8)	8.9 (3.9, 15.7)	12.0 (8.5, 17.0)	20.2 (6.3, 34.4)	0.7 (0.2, 1.1)	20.9 (13.0, 28.8)	4.9 (1.3, 9.3)	6.2 (3.9, 9.0)		6.7 (3.7, 9.2)	20.1 (9.1, 29.6)	12.5 (8.6, 18.4)	2.2 (1.6, 2.4)
Lee (2022)[32]	USA	Hispanic	All	14.0 (6.6, 21.5)	10.5 (4.7, 18.5)	7.6 (5.3, 11.0)	18.8 (5.8, 32.3)	0.4 (0.1, 0.6)	22.5 (14.1, 30.8)	4.0 (1.0, 7.6)	8.8 (5.6, 12.5)		12.6 (7.1, 16.9)	21.5 (9.9, 31.5)	17.0 (11.9, 24.5)	4.3 (3.0, 4.7)
Lee (2022)[32]	USA	Asian, non-Hispanic	All	3.7 (1.6, 6.1)	5.8 (2.6, 10.7)	4.6 (3.1, 6.7)	18.8 (5.8, 32.3)	0.1 (0.0, 0.2)	8.1 (4.7, 11.8)	2.9 (0.7, 5.6)	3.7 (2.3, 5.4)		4.6 (2.5, 6.4)	18.5 (8.3, 27.5)	18.1 (12.7, 25.8)	5.2 (3.7, 5.7)
Lee (2022)[32]	USA	Black, non-Hispanic	All	6.0 (2.7, 9.7)	5.5 (2.4, 10.1)	6.9 (4.7, 9.9)	26.8 (8.9, 43.1)	0.5 (0.2, 0.8)	24.6 (15.6, 33.3)	6.6 (1.7, 12.3)	5.6 (3.5, 8.1)		6.8 (3.7, 9.3)	22.6 (10.5, 32.9)	15.7 (10.9, 22.7)	4.0 (2.8, 4.3)
Lee (2022)[32]	USA	White, non-Hispanic	All	3.2 (1.4, 5.3)	8.7 (3.9, 15.7)	13.9 (9.8, 19.4)	19.3 (6.0, 33.0)	0.8 (0.3, 1.3)	20.7 (12.9, 28.6)	4.8 (1.1, 9.2)	6.1 (3.8, 8.7)		6.1 (3.3, 8.4)	19.7 (8.9, 29.1)	11.3 (7.7, 16.7)	1.7 (1.2, 1.9)
Nianogo (2022)[44]	USA	Total population	ADRD	11.7 (11.5, 12.0)	2.3 (2.2, 2.3)		8.8 (8.7, 8.9)		17.7 (17.5, 18.0)	6.0 (5.9, 6.1)	8.5 (8.4, 8.6)			11.8 (11.7, 11.9)	7.3 (7.1, 7.4)	
Nianogo (2022)[44]	USA	Asian	ADRD	4.7 (3.6, 5.8)	1.2 (0.8, 1.5)		6.3 (5.5, 7.1)		7.4 (6.1, 8.6)	3.2 (2.7, 3.6)	3.7 (3.2, 4.1)			9.5 (8.7, 10.3)	5.9 (5.1, 6.8)	
Nianogo (2022)[44]	USA	Black	ADRD	12.5 (11.8, 13.1)	1.6 (1.5, 1.8)		11.9 (11.6, 12.2)		21.7 (21.1, 22.3)	6.7 (6.4, 7.0)	7.5 (7.2, 7.9)			13.7 (13.3, 14.1)	9.4 (9.0, 9.8)	
Nianogo (2022)[44]	USA	Hispanic	ADRD	26.5 (25.8, 27.1)	1.7 (1.5, 1.9)		7.8 (7.5, 8.2)		18.8 (18.0, 19.6)	4.9 (4.6, 5.2)	6.9 (6.6, 7.3)			14.2 (13.8, 14.7)	7.2 (6.7, 7.7)	
Nianogo (2022)[44]	USA	Native American and Alaska Native	ADRD	17.5 (15.6, 19.3)	4.1 (3.5, 4.7)		11.0 (10.2, 11.7)		20.3 (18.7, 21.8)	10.7 (9.7, 11.6)	11.3 (10.3, 12.2)			14.0 (12.8, 15.1)	10.5 (9.3, 11.7)	
Nianogo (2022)[44]	USA	White	ADRD	7.5 (7.3, 7.7)	2.6 (2.5, 2.7)		8.5 (8.4, 8.6)		17.3 (17.1, 17.5)	6.3 (6.2, 6.4)	9.3 (9.2, 9.5)			11.0 (10.9, 11.2)	6.9 (6.8, 7.0)	
Smith (2023)[59]	USA	White, non-Hispanic (M-HL)	All		11.2 (-5.2, 26.6)											
Smith (2023)[59]	USA	White, non-Hispanic (≥MOD-HL)	All		20.6 (3.6, 36.2)											

Author (year)	Country	Ethnicity	Outcome	Education	Hearing Loss	TBI	Hypertension	Alcohol	Obesity	Smoking	DEP (Lifetime)	DEP (Later-life)	Social Isolation	Physical Inactivity	Diabetes	Air Pollution
Smith (2023)[59]	USA	White, non-Hispanic (ANY-HL)	All		30.6 (-0.6, 58)											
Smith (2023)[59]	USA	Black, non-Hispanic (M-HL)	All		-12.2 (-35.8, 9.9)											
Smith (2023)[59]	USA	Black, non-Hispanic (≥MOD-HL)	All		-5.3 (-21.2, 8.4)											
Smith (2023)[59]	USA	Black, non-Hispanic (ANY-HL)	All		-17.9 (-54.4, 15.1)											
Borelli (2023)[8]	Brazil	Asian	All	10.0	13.4		2.5	0.1	1.9	3.2	11.8		0.4	5.1	2.1	
Borelli (2023)[8]	Brazil	Black	All	14.1	15.2		7.8	1.8	5.4	3.4	3.6		1.4	10.6	3.8	
Borelli (2023)[8]	Brazil	Brown	All	15.9	15.2		9.8	2	6.1	1.7	2.4		2.3	10.4	3.1	
Borelli (2023)[8]	Brazil	Indigenous	All	7.2	16.7		5.9	0	1.6	2.6	4.1		0.1	6.4	2.0	
Borelli (2023)[8]	Brazil	White	All	11.7	15.2		9.4	0.2	7.1	2.1	2.5		2.2	9.9	4.0	
Suemoto (2023)[61]	Brazil	Black	All	24.1 (22.9, 25.3)	19.5 (18.2, 20.9)	8.8 (7.9, 9.8)	22.6 (21.2, 24.0)	0.9 (0.6, 1.3)	15.6 (14.4, 16.9)	5.9 (4.9, 7.1)	11.2 (9.8, 12.7)		1.0 (0.6, 1.5)	13.0 (11.5, 14.6)	8.3 (7.1, 10.0)	7.4 (6.2, 8.7)
Suemoto (2023)[61]	Brazil	White	All	19.1 (17.8, 20.4)	19.1 (17.5, 20.8)	8.8 (7.6, 10.0)	20.4 (18.7, 22.2)	0.8 (0.4, 1.2)	15.9 (14.4, 17.5)	5.7 (4.5. 7.0)	13.9 (12.1, 15.8)		0.8 (0.4, 1.4)	12.4 (10.7, 14.2)	9.4 (7.9, 11.0)	8.0 (6.6, 9.5)
**Weighted**																
Thompson (2022)[63]	AUS	First Nations people including Torres Strait Islander and Aboriginal people	All	5.7 (3.3, 8.1)	5.1 (2.9, 7.3)	4.3 (2.3, 6.4)	10.5 (7.4, 13.6)	0.3 (-0.3, 0.8)	6.3 (3.8, 8.8)	4.1 (2.1, 6.1)	1.9 (0.5, 3.3)		1.6 (0.3, 2.8)	3.5 (1.7, 5.4)	9.5 (6.5, 12.5)	
Ma'u (2021)[38]	NA	Total population	All	4.6	7.8	3.7	4.9	0.6	5.5	2.2		4.3	5.4	5.2	1.6	1.9
Ma'u (2021)[38]	NA	Māori	All	5.6	6.5	3.5	5	0.7	7.3	4.3		4.2	5.1	5.0	2.4	1.8
Ma'u (2021)[38]	NZ	Pacific peoples	All	6.0	6.6	3.6	5.5	0.3	8.9	2.8		2.0	3.0	5.6	4.1	2.4
Ma'u (2021)[38]	NZ	Asian	All	3.8	7.3	3.9	4.9	0.3	2.9	1.1		2.6	3.1	5.5	2.8	2.6
Ma'u (2021)[38]	NZ	European	All	4.5	8.0	3.7	4.9	0.6	5	1.9		4.7	5.7	5.2	1.2	1.9
Ehrlich (2022)[15]	USA	Total population	All	3.1	7.0	6.1	12.4	0.3	9.2	3.2	9.1		1.9	3.1	5.1	
Ehrlich (2022)[15]	USA	Black, non-Hispanic	All	4.9	5.8	6.5	13.7	0.1	10.1	4.4	7.9		3.2	3.7	6.2	
Ehrlich (2022)[15]	USA	Hispanic, any race	All	9.0	8.2	3.8	11.2	0.2	9.1	3.1	9.2		3.2	3.2	7.0	
Ehrlich (2022)[15]	USA	White, non-Hispanic	All	2.4	7.2	6.0	11.9	0.3	9.2	3.0	9.4		1.7	3.0	4.8	
Suemoto (2023)[61]	Brazil	Black	All	8.2 (7.5, 9.0)	6.6 (5.8, 7.5)	3.0 (2.4, 3.6)	7.7 (6.8, 8.6)	0.3 (0.1, 0.6)	5.3 (4.6, 6.1)	2.0 (1.4, 2.8)	3.8 (3.0, 4.8)		0.3 (0.1, 0.7)	4.4 (3.5, 5.4)	2.8 (2.1, 3.6)	2.5 (1.8, 3.3)
Suemoto (2023)[61]	Brazil	White	All	6.8 (6.0, 7.7)	6.8 (5.8, 8.0)	3.1 (2.4, 3.9)	7.3 (6.3, 8.5)	0.3 (0.1, 0.7)	5.7 (4.7, 6.7)	2.0 (1.3, 2.9)	5.0 (3.9, 6.2)		0.3 (0.1, 0.7)	4.4 (3.4, 5.6)	3.4 (2.5, 4.5)	2.8 (2.0, 3.8)

Key AD=Alzheimer’s disease; ADRD=Alzheimer’s disease and associated disorders; ANY-HL=Any hearing loss; AUS=Australia; DEP=Depression; HL= Hearing Loss; M-HL=Mild hearing loss; MOD-HL=Moderate hearing loss; NZ=New Zealand; TBI=Traumatic brain injury; USA=United States of America

Appendix 19 Bar graphs showing Individual PAF values for risk factors reported in the Lancet Commission[35] stratified by ethnicity across the different countries

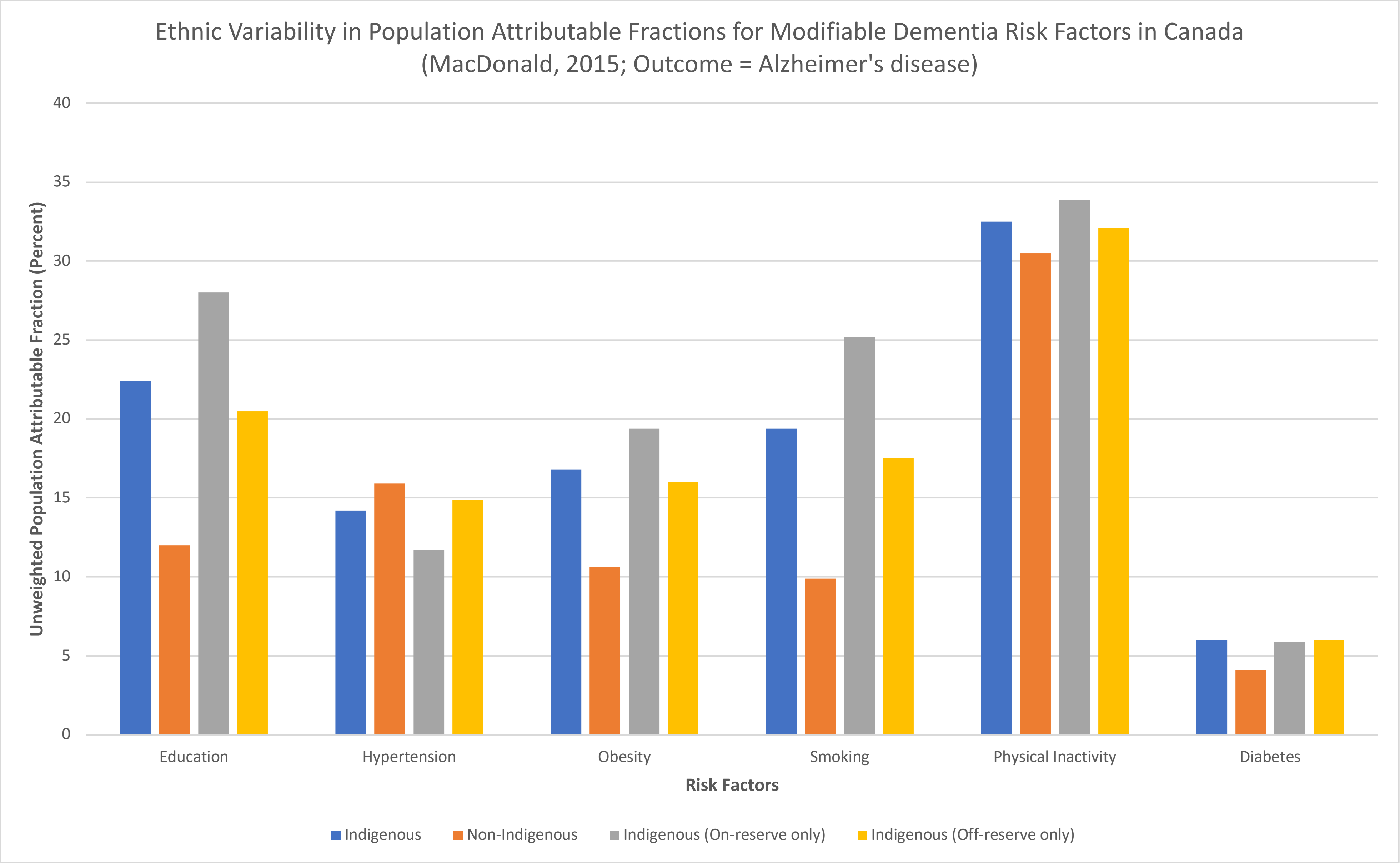

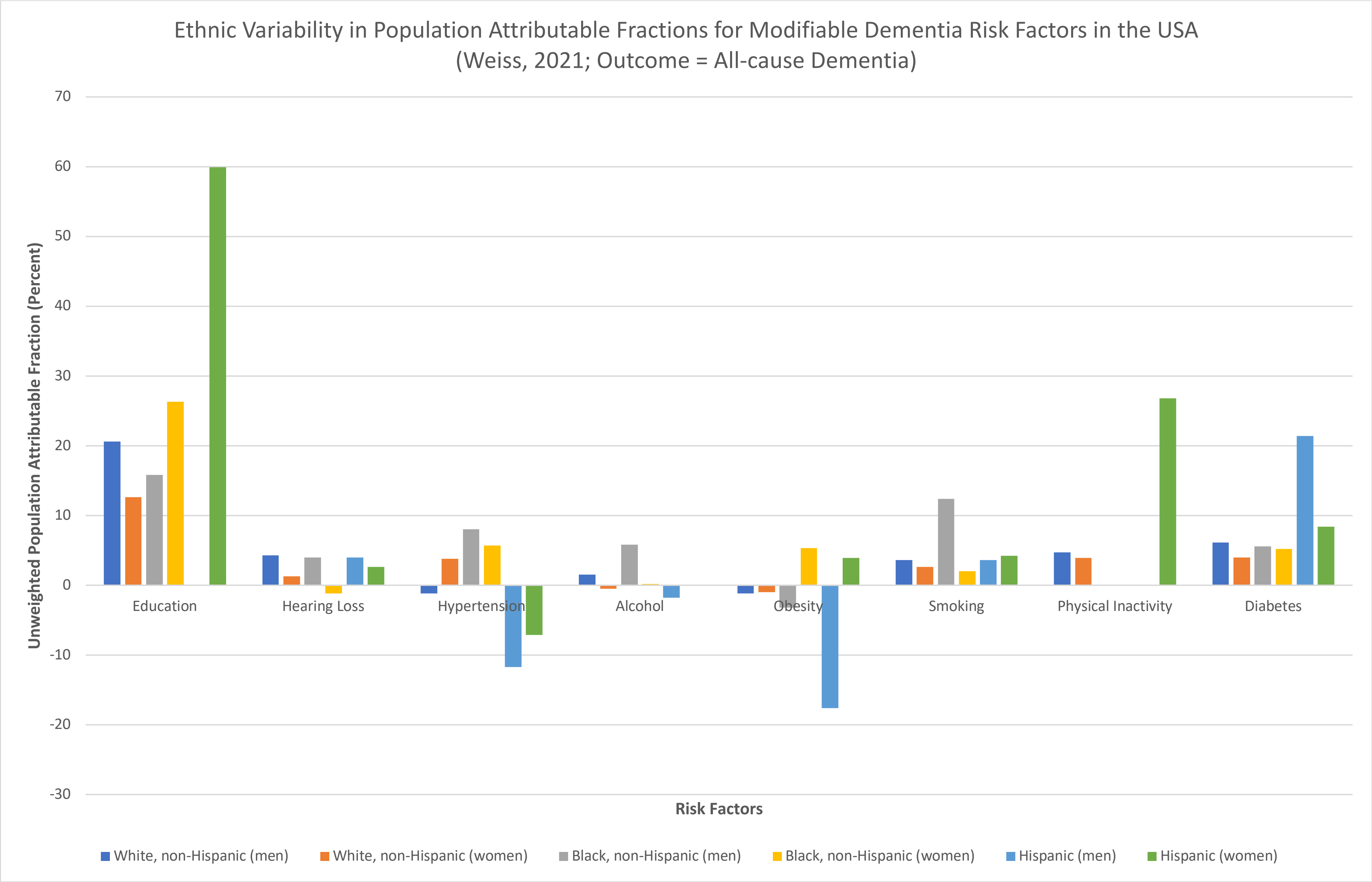
Ethnic Variability in Population Attributable Fractions for Modifiable Dementia Risk Factors in the USA
(Weiss, 2021; Outcome = All-cause Dementia)
Unweighted Population Attributable Fraction (Percent)
70
60
50
40
30
20
10
0
-10
-20
-30
Education
Hearing Loss
Hypertension
Alcohol
Obesity
Smoking
Physical Inactivity
Diabetes
Risk Factors
White, non-Hispanic (men)
White, non-Hispanic (women)
Black, non-Hispanic (men)
Black, non-Hispanic (women)
Hispanic (men)
Hispanic (women)

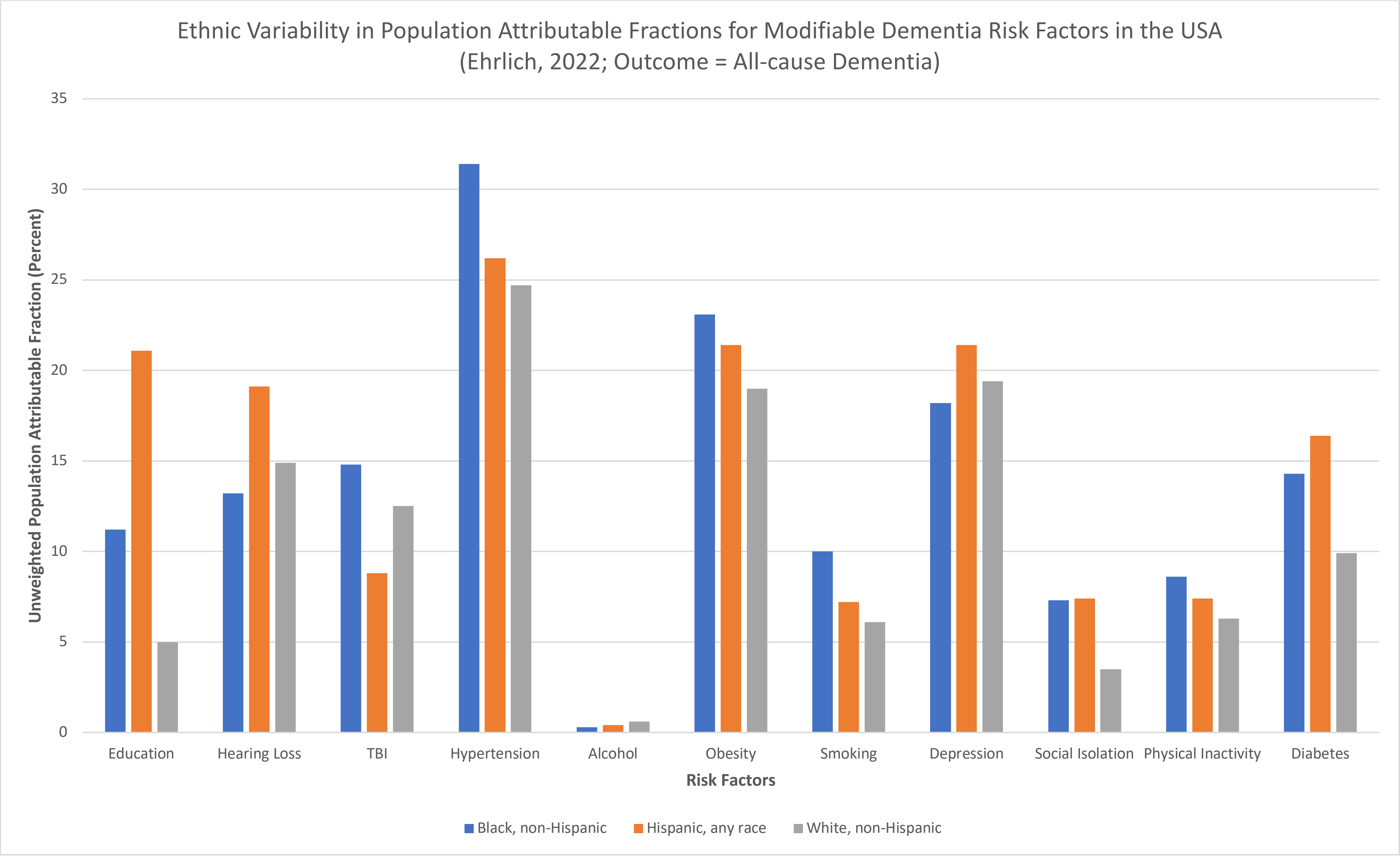
Ethnic Variability in Population Attributable Fractions for Modifiable Dementia Risk Factors in the USA
(Ehrlich, 2022; Outcome = All-cause Dementia)
Unweighted Population Attributable Fraction (Percent)
35
30
25
20
15
10
5
0
Education
Hearing Loss
TBI
Hypertension
Alcohol
Obesity
Smoking
Depression
Social Isolation
Physical Inactivity
Diabetes
Risk Factors
Black, non-Hispanic
Hispanic, any race
White, non-Hispanic

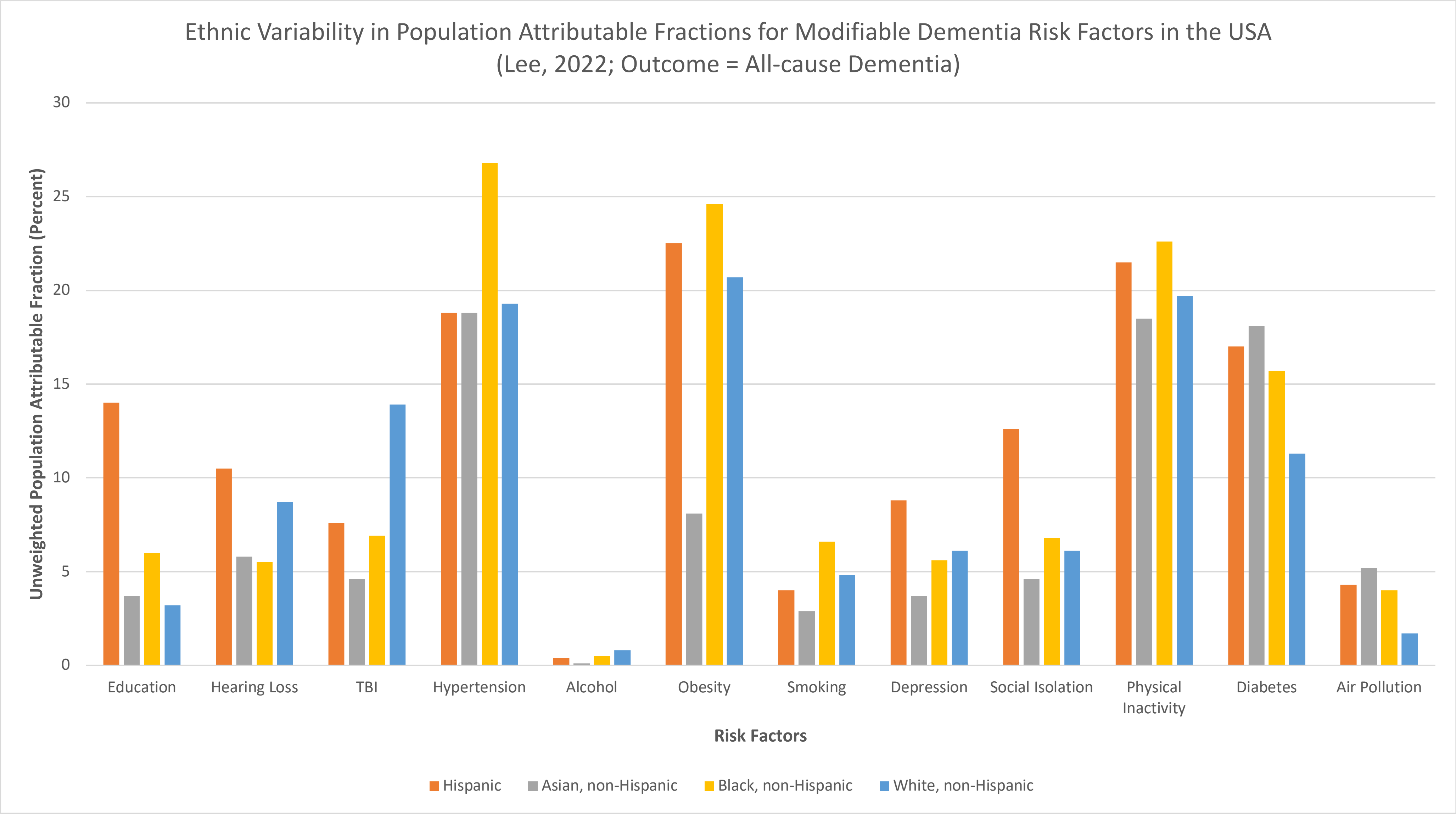
Ethnic Variability in Population Attributable Fractions for Modifiable Dementia Risk Factors in the USA
(Lee, 2022; Outcome = All-cause Dementia)
Unweighted Population Attributable Fraction (Percent)
30
25
20
15
10
5
0
Education
Hearing Loss
TBI
Hypertension
Alcohol
Obesity
Smoking
Depression
Social Isolation
Physical Inactivity
Diabetes
Air Pollution
Risk Factors
Hispanic
Asian, non-Hispanic
Black, non-Hispanic
White, non-Hispanic

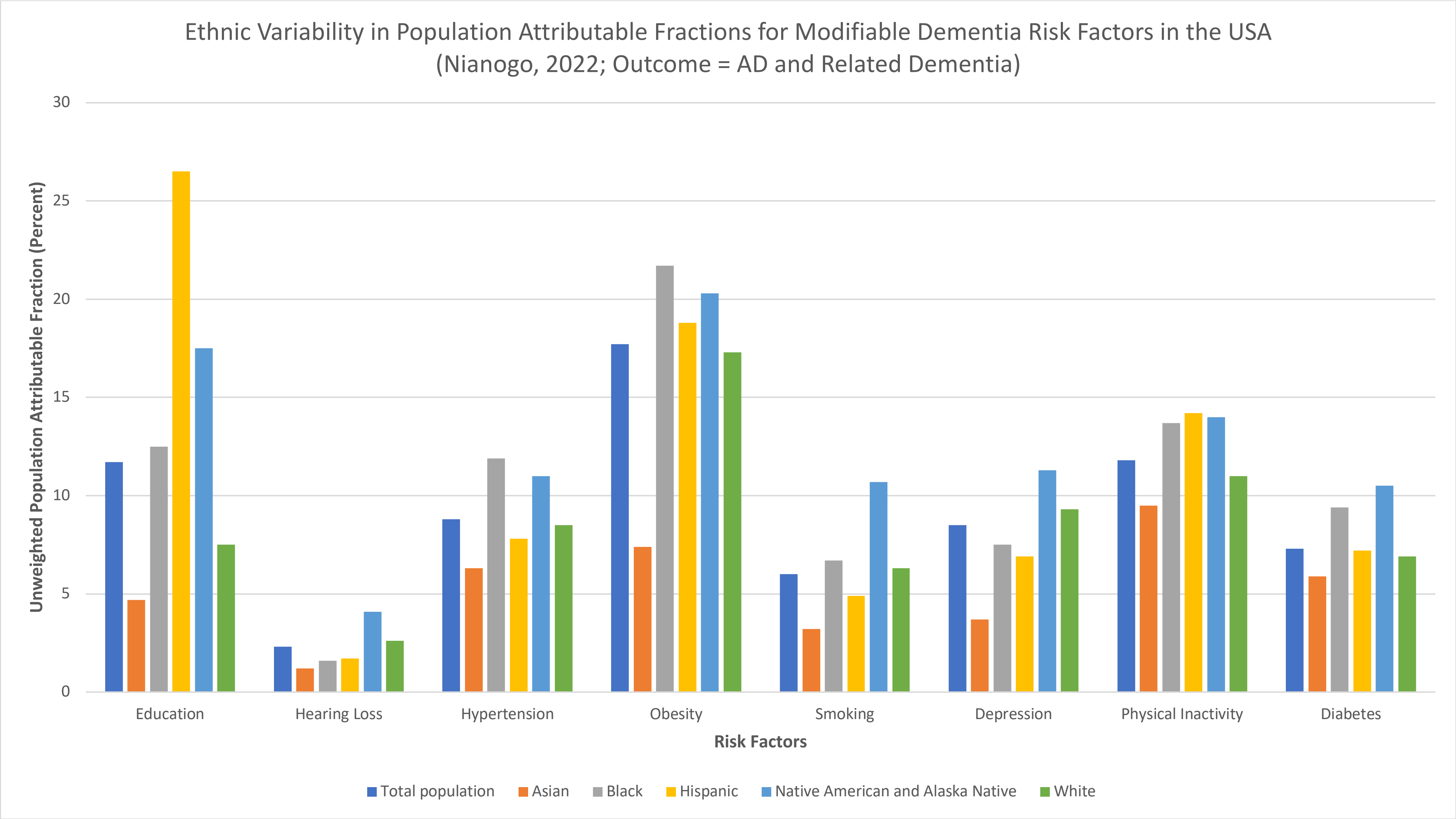

Ethnic Variability in Population Attributable Fractions for Modifiable Dementia Risk Factors in the USA
(Nianogo, 2022; Outcome = AD and Related Dementia)
Unweighted Population Attributable Fraction (Percent)
30
25
20
15
10
5
0
Education
Hearing Loss
Hypertension
Obesity
Smoking
Depression
Physical Inactivity
Diabetes
Risk Factors
Total population
Asian
Black
Hispanic
Native American and Alaska Native
White

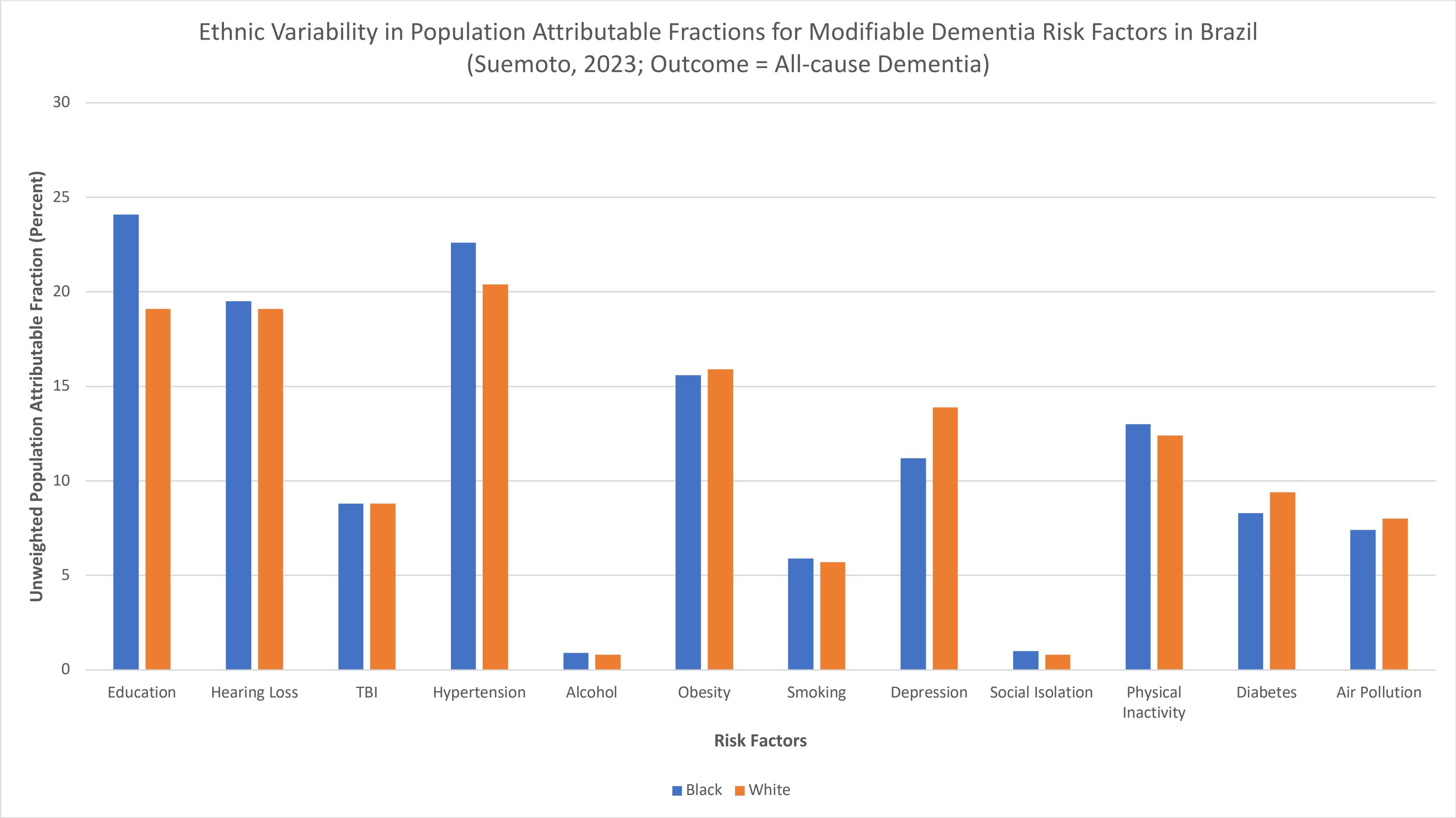

Ethnic Variability in Population Attributable Fractions for Modifiable Dementia Risk Factors in Brazil
(Suemoto, 2023; Outcome = All-cause Dementia)
30
25
20
15
10
5
0
Unweighted Population Attributable Fraction (Percent)
Education
Hearing Loss
TBI
Hypertension
Alcohol
Obesity
Smoking
Depression
Social Isolation
Physical Inactivity
Diabetes
Air Pollution
Risk Factors
Black
White

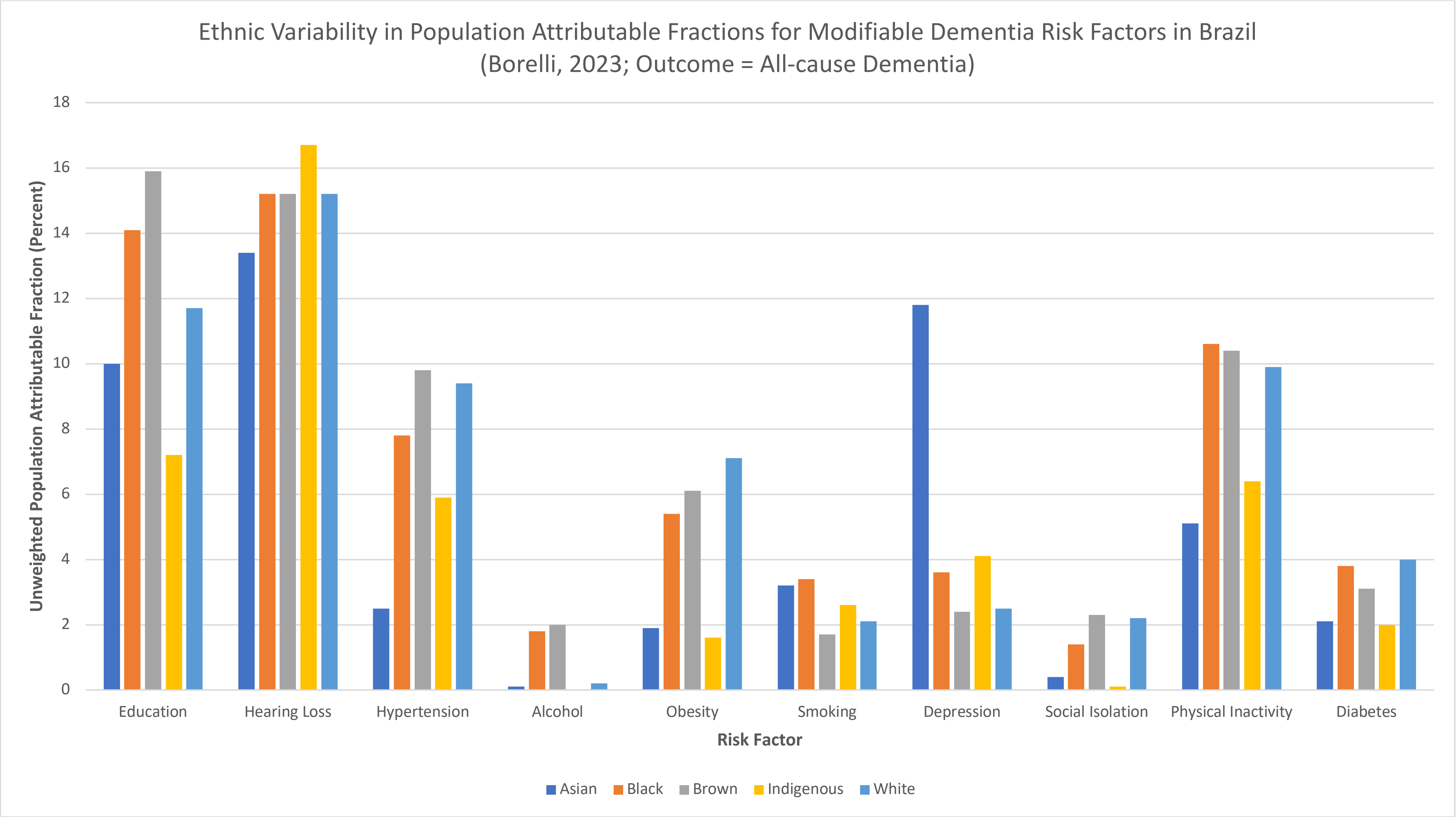
Ethnic Variability in Population Attributable Fractions for Modifiable Dementia Risk Factors in Brazil
(Borelli, 2023; Outcome = All-cause Dementia)
Unweighted Population Attributable Fraction (Percent)
0
2
4
6
8
10
12
14
16
18
Education
Hearing Loss
Hypertension
Alcohol
Obesity
Smoking
Depression
Social Isolation
Physical Inactivity
Diabetes
Risk Factor
Asian
Black
Brown
Indigenous
White

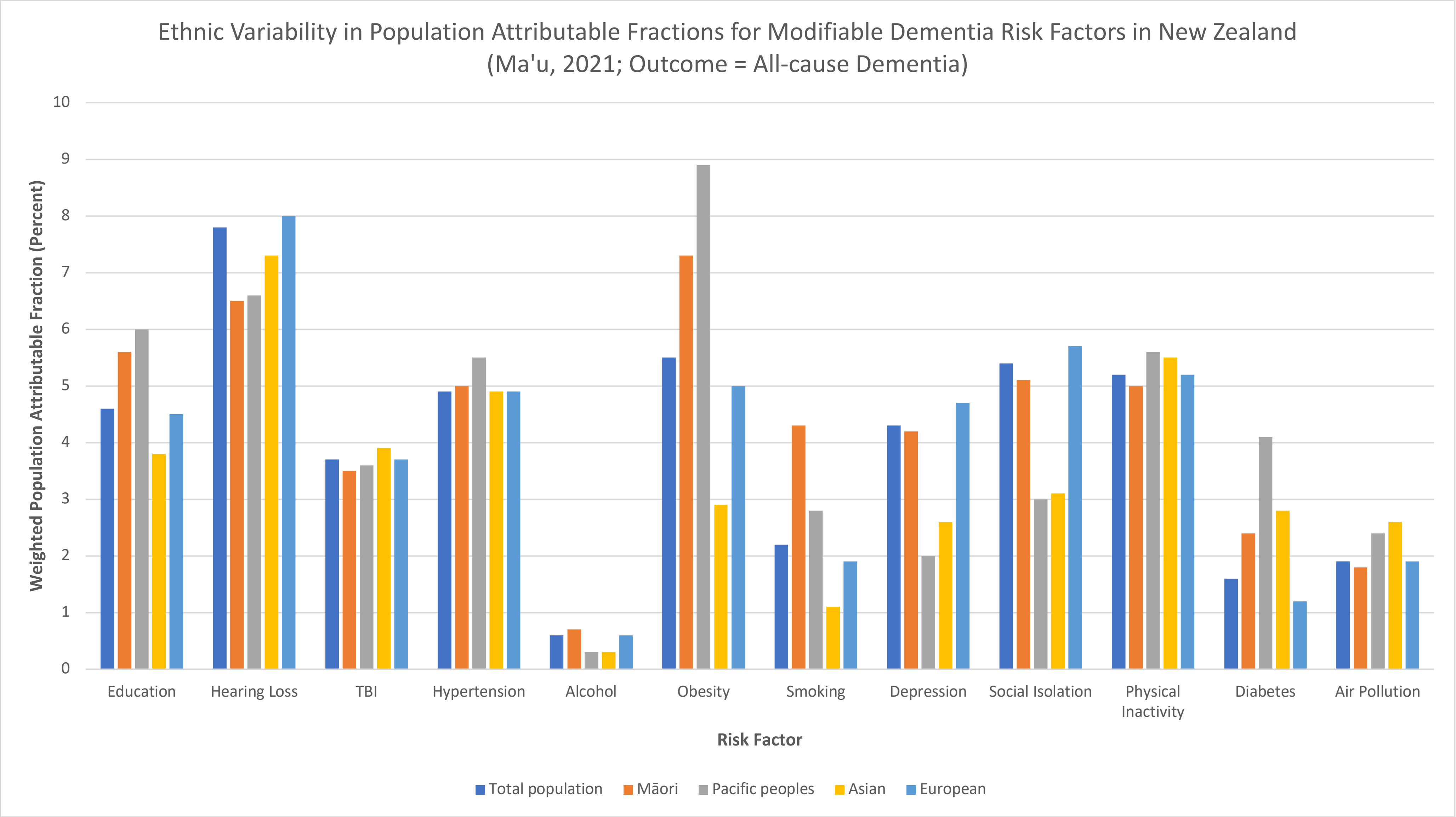
Ethnic Variability in Population Attributable Fractions for Modifiable Dementia Risk Factors in New Zealand
(Ma'u, 2021; Outcome = All-cause Dementia)
Weighted Population Attributable Fraction (Percent)
10
9
8
7
6
5
4
3
2
1
0
Education
Hearing Loss
TBI
Hypertension
Alcohol
Obesity
Smoking
Depression
Social Isolation
Physical Inactivity
Diabetes
Air Pollution
Risk Factor
Total population
Māori
Pacific peoples
Asian
European

References

1. Ashby-Mitchell K, Burns R, Shaw J, Anstey KJ. Proportion of dementia in Australia explained by common modifiable risk factors. *Alzheimers Res Ther* 2017; **9**(1): 11.

2. Ashby-Mitchell K, Burns R, Anstey KJ. The proportion of dementia attributable to common modifiable lifestyle factors in Barbados. *Rev Panam Salud Publica* 2018; **42**: e17.

3. Ashby-Mitchell K, Willie-Tyndale D, Eldemire-Shearer D. Proportion of Dementia Explained by Five Key Factors in Jamaica. *J Alzheimers Dis* 2020; **78**(2): 603-9.

4. Barnes DE, Yaffe K. The projected effect of risk factor reduction on Alzheimer's disease prevalence. *Lancet Neurol* 2011; **10**(9): 819-28.

5. Beydoun MA, Beydoun HA, Gamaldo AA, Teel A, Zonderman AB, Wang Y. Epidemiologic studies of modifiable factors associated with cognition and dementia: systematic review and meta-analysis. *BMC Public Health* 2014; **14**: 643.

6. Bobrow K, Hoang T, Barnes DE, Gardner RC, Allen IE, Yaffe K. The Effect of Sex and Wealth on Population Attributable Risk Factors for Dementia in South Africa. *Front Neurol* 2021; **12**: 766705.

7. Borelli WV, Leotti VB, Strelow MZ, Chaves MLF, Castilhos RM. Preventable risk factors of dementia: Population attributable fractions in a Brazilian population-based study. *The Lancet Regional Health - Americas* 2022; **11**: 100256.

8. Borelli WV, Formoso CR, Bieger A, et al. Race-related population attributable fraction of preventable risk factors of dementia: A Latino population-based study. *Alzheimers Dement (Amst)* 2023; **15**(1): e12408.

9. Bothongo PLK, Jitlal M, Parry E, et al. Dementia risk in a diverse population: A single-region nested case-control study in the East End of London. *Lancet Reg Health Eur* 2022; **15**: 100321.

10. Bubu OM, Brannick M, Mortimer J, et al. Sleep, Cognitive impairment, and Alzheimer's disease: A Systematic Review and Meta-Analysis. *Sleep* 2017; **40**(1).

11. de Bruijn RF, Bos MJ, Portegies ML, et al. The potential for prevention of dementia across two decades: the prospective, population-based Rotterdam Study. *BMC Med* 2015; **13**: 132.

12. Desai R, John A, Stott J, Charlesworth G. Living alone and risk of dementia: A systematic review and meta-analysis. *Ageing Res Rev* 2020; **62**: 101122.

13. Dodge HH, Chang CC, Kamboh IM, Ganguli M. Risk of Alzheimer's disease incidence attributable to vascular disease in the population. *Alzheimers Dement* 2011; **7**(3): 356-60.

14. Dragioti E, Radua J, Solmi M, et al. Global population attributable fraction of potentially modifiable risk factors for mental disorders: a meta-umbrella systematic review. *Molecular Psychiatry* 2022; **27**(8): 3510-9.

15. Ehrlich JR, Goldstein J, Swenor BK, Whitson H, Langa KM, Veliz P. Addition of Vision Impairment to a Life-Course Model of Potentially Modifiable Dementia Risk Factors in the US. *JAMA Neurol* 2022; **79**(6): 623-6.

16. Esteban-Cornejo I, Ho FK, Petermann-Rocha F, et al. Handgrip strength and all-cause dementia incidence and mortality: findings from the UK Biobank prospective cohort study. *J Cachexia Sarcopenia Muscle* 2022; **13**(3): 1514-25.

17. Feter N, Leite JS, Cassuriaga J, et al. Are gender differences in physical inactivity associated with the burden of dementia in low- and lower-middle income countries? *Glob Public Health* 2022; **17**(5): 727-37.

18. Gardner RC, Bahorik A, Kornblith ES, Allen IE, Plassman BL, Yaffe K. Systematic Review, Meta-Analysis, and Population Attributable Risk of Dementia Associated with Traumatic Brain Injury in Civilians and Veterans. *J Neurotrauma* 2023; **40**(7-8): 620-34.

19. GBD 2019 Dementia Collaborators. The Burden of Dementia due to Down Syndrome, Parkinson's Disease, Stroke, and Traumatic Brain Injury: A Systematic Analysis for the Global Burden of Disease Study 2019. *Neuroepidemiology* 2021; **55**(4): 286-96.

20. Hagström E, Kilander L, Nylander R, et al. Plasma parathyroid hormone is associated with vascular dementia and cerebral hyperintensities in two community-based cohorts. *J Clin Endocrinol Metab* 2014; **99**(11): 4181-9.

21. Hazar N, Seddigh L, Rampisheh Z, Nojomi M. Population attributable fraction of modifiable risk factors for Alzheimer disease: A systematic review of systematic reviews. *Iran J Neurol* 2016; **15**(3): 164-72.
22. Hegelund ER, Mehta AJ, Mortensen LH, Westendorp RGJ. The plasticity of late-onset dementia: A nationwide cohort study in Denmark. *Alzheimers Dement* 2022; **18**(7): 1287-95.
23. Hodis JD, Gottesman RF, Windham BG, et al. Association of Hypertension According to New American College of Cardiology/American Heart Association Blood Pressure Guidelines With Incident Dementia in the ARIC Study Cohort. *J Am Heart Assoc* 2020; **9**(22): e017546.
24. Hu FF, Cheng GR, Liu D, et al. Population-attributable fractions of risk factors for all-cause dementia in China rural and urban areas: A cross-sectional study. *J Neurol* 2022; **269**(6): 3147-58.
25. Hu M, Gao Y, Kwok TCY, Shao Z, Xiao LD, Feng H. Derivation and Validation of the Cognitive Impairment Prediction Model in Older Adults: A National Cohort Study. *Front Aging Neurosci* 2022; **14**: 755005.
26. Johannesdottir Schmidt SA, Veres K, Sørensen HT, Obel N, Henderson VW. Incident Herpes Zoster and Risk of Dementia: A Population-Based Danish Cohort Study. *Neurology* 2022; **99**(7): e660-8.
27. Jørgensen K, Nielsen TR, Nielsen A, Waldemar G. Potential for prevention of dementia in Denmark. *Alzheimers Dement* 2023; **19**(10): 4590-8.
28. Katzmarzyk PT, Friedenreich C, Shiroma EJ, Lee IM. Physical inactivity and non-communicable disease burden in low-income, middle-income and high-income countries. *Br J Sports Med* 2022; **56**(2): 101-6.
29. Kloppenborg RP, van den Berg E, Kappelle LJ, Biessels GJ. Diabetes and other vascular risk factors for dementia: which factor matters most? A systematic review. *Eur J Pharmacol* 2008; **585**(1): 97-108.
30. Kotaki Y, Tomata Y, Tanji F, Zhang S, Sugawara Y, Tsuji I. Joint impact of seven risk factors on incident dementia in elderly Japanese: the Ohsaki Cohort 2006 Study. *J Neurol* 2019; **266**(5): 1222-9.
31. Launer LJ, Hughes T, Yu B, et al. Lowering midlife levels of systolic blood pressure as a public health strategy to reduce late-life dementia: perspective from the Honolulu Heart Program/Honolulu Asia Aging Study. *Hypertension* 2010; **55**(6): 1352-9.
32. Lee M, Whitsel E, Avery C, et al. Variation in Population Attributable Fraction of Dementia Associated With Potentially Modifiable Risk Factors by Race and Ethnicity in the US. *JAMA Netw Open* 2022; **5**(7): e2219672.
33. Liu Y, Zhang S, Tomata Y, Nurrika D, Sugawara Y, Tsuji I. The impact of risk factors for dementia in China. *Age Ageing* 2020; **49**(5): 850-5.
34. Livingston G, Sommerlad A, Orgeta V, et al. Dementia prevention, intervention, and care. *Lancet* 2017; **390**(10113): 2673-734.
35. Livingston G, Huntley J, Sommerlad A, et al. Dementia prevention, intervention, and care: 2020 report of the Lancet Commission. *Lancet* 2020; **396**(10248): 413-46.
36. Loef M, Walach H. Midlife obesity and dementia: meta-analysis and adjusted forecast of dementia prevalence in the United States and China. *Obesity (Silver Spring)* 2013; **21**(1): E51-5.
37. Luck T, Riedel-Heller SG. Prevention of Alzheimer's dementia in Germany: A projection of the possible potential of reducing selected risk factors. *Nervenarzt* 2016; **87**(11): 1194-200.
38. Ma'u E, Cullum S, Cheung G, Livingston G, Mukadam N. Differences in the potential for dementia prevention between major ethnic groups within one country: A cross sectional analysis of population attributable fraction of potentially modifiable risk factors in New Zealand. *Lancet Reg Health West Pac* 2021; **13**: 100191.
39. MacDonald JP, Barnes DE, Middleton LE. Implications of Risk Factors for Alzheimer's Disease in Canada's Indigenous Population. *Can Geriatr J* 2015; **18**(3): 152-8.
40. Mayer F, Di Pucchio A, Lacorte E, et al. An Estimate of Attributable Cases of Alzheimer Disease and Vascular Dementia due to Modifiable Risk Factors: The Impact of Primary Prevention in Europe and in Italy. *Dement Geriatr Cogn Dis Extra* 2018; **8**(1): 60-71.

41. Mukadam N, Sommerlad A, Huntley J, Livingston G. Population attributable fractions for risk factors for dementia in low-income and middle-income countries: an analysis using cross-sectional survey data. *Lancet Glob Health* 2019; **7**(5): e596-e603.
42. Mukadam N, Anderson R, Knapp M, et al. Effective interventions for potentially modifiable risk factors for late-onset dementia: a costs and cost-effectiveness modelling study. *Lancet Healthy Longev* 2020; **1**(1): e13-e20.
43. Mulligan MD, Murphy R, Reddin C, et al. Population attributable fraction of hypertension for dementia: global, regional, and national estimates for 186 countries. *EClinicalMedicine* 2023; **60**: 102012.
44. Nianogo RA, Rosenwohl-Mack A, Yaffe K, Carrasco A, Hoffmann CM, Barnes DE. Risk Factors Associated With Alzheimer Disease and Related Dementias by Sex and Race and Ethnicity in the US. *JAMA Neurol* 2022; **79**(6): 584-91.
45. Norton S, Matthews FE, Barnes DE, Yaffe K, Brayne C. Potential for primary prevention of Alzheimer's disease: an analysis of population-based data. *Lancet Neurol* 2014; **13**(8): 788-94.
46. Oliveira D, Jun Otuyama L, Mabunda D, et al. Reducing the Number of People with Dementia Through Primary Prevention in Mozambique, Brazil, and Portugal: An Analysis of Population-Based Data. *J Alzheimers Dis* 2019; **70**(s1): S283-s91.
47. Ren L, Liang J, Wan F, Wang Y, Dai XJ. Development of a Clinical Risk Score Prediction Tool for 5-, 9-, and 13-Year Risk of Dementia. *JAMA Netw Open* 2022; **5**(11): e2242596.
48. Rogers MA, Plassman BL, Kabeto M, et al. Parental education and late-life dementia in the United States. *J Geriatr Psychiatry Neurol* 2009; **22**(1): 71-80.
49. Rolandi E, Zaccaria D, Vaccaro R, et al. Estimating the potential for dementia prevention through modifiable risk factors elimination in the real-world setting: a population-based study. *Alzheimers Res Ther* 2020; **12**(1): 94.
50. Rydén L, Zettergren A, Seidu NM, et al. Atrial fibrillation increases the risk of dementia amongst older adults even in the absence of stroke. *J Intern Med* 2019; **286**(1): 101-10.
51. Santabarbara J, Sevil-Perez A, Olaya B, Gracia-Garcia P, Lopez-Anton R. Clinically relevant late-life depression as risk factor of dementia: a systematic review and meta-analysis of prospective cohort studies. *Rev Neurol* 2019a; **68**(12): 493-502.
52. Santabárbara J, Villagrasa B, López-Antón R, et al. Clinically relevant anxiety and risk of Alzheimer's disease in an elderly community sample: 4.5 years of follow-up. *J Affect Disord* 2019b; **250**: 16-20.
53. Santabárbara J, Lipnicki DM, Olaya B, et al. Does Anxiety Increase the Risk of All-Cause Dementia? An Updated Meta-Analysis of Prospective Cohort Studies. *J Clin Med* 2020a; **9**(6).
54. Santabárbara J, Villagrasa B, Gracia-García P. Does depression increase the risk of dementia? Updated meta-analysis of prospective studies. *Actas Esp Psiquiatr* 2020b; **48**(4): 169-80.
55. Scazufca M, Almeida OP, Menezes PR. The role of literacy, occupation and income in dementia prevention: the São Paulo Ageing & Health Study (SPAH). *Int Psychogeriatr* 2010; **22**(8): 1209-15.
56. Shang X, Zhu Z, Wang W, Ha J, He M. The Association between Vision Impairment and Incidence of Dementia and Cognitive Impairment: A Systematic Review and Meta-analysis. *Ophthalmology* 2021; **128**(8): 1135-49.
57. Shang X, Zhu Z, Zhang X, et al. Association of a wide range of chronic diseases and apolipoprotein E4 genotype with subsequent risk of dementia in community-dwelling adults: A retrospective cohort study. *EClinicalMedicine* 2022; **45**: 101335.
58. Skirbekk V, Bowen CE, Håberg A, et al. Marital Histories and Associations With Later-Life Dementia and Mild Cognitive Impairment Risk in the HUNT4 70+ Study in Norway. *J Aging Health* 2023; **35**(7-8): 543-55.
59. Smith JR, Huang AR, Lin FR, Reed NS, Deal JA. The Population Attributable Fraction of Dementia From Audiometric Hearing Loss Among a Nationally Representative Sample of Community-Dwelling Older Adults. *J Gerontol A Biol Sci Med Sci* 2023; **78**(7): 1300-6.

60. Smith JR, Sharrett AR, Pike JR, et al. Dementia occurring over a 32-year follow-up attributable to hypertension observed at different ages: Implications for dementia prevention. *Alzheimers Dement* 2023; **19**(8): 3435-47.
61. Suemoto CK, Mukadam N, Brucki SMD, et al. Risk factors for dementia in Brazil: Differences by region and race. *Alzheimers Dement* 2023; **19**(5): 1849-57.
62. Suh SW, Han JW, Park JY, et al. Impacts of Illiteracy on the Risk of Dementia: A Global Health Perspective. *J Alzheimers Dis* 2016; **53**(2): 731-41.
63. Thompson F, Russell S, Quigley R, et al. Potentially preventable dementia in a First Nations population in the Torres Strait and Northern Peninsula Area of North Queensland, Australia: A cross sectional analysis using population attributable fractions. *Lancet Reg Health West Pac* 2022; **26**: 100532.
64. Tomata Y, Zhang S, Sugawara Y, Tsuji I. Impact of time spent walking on incident dementia in elderly Japanese. *Int J Geriatr Psychiatry* 2019; **34**(1): 204-9.
65. Tomata Y, Li X, Karlsson IK, Mosing MA, Pedersen NL, Hägg S. Joint impact of common risk factors on incident dementia: A cohort study of the Swedish Twin Registry. *J Intern Med* 2020; **288**(2): 234-47.
66. Vagelatos NT, Eslick GD. Type 2 diabetes as a risk factor for Alzheimer's disease: the confounders, interactions, and neuropathology associated with this relationship. *Epidemiol Rev* 2013; **35**: 152-60.
67. Vergara RC, Zitko P, Slachevsky A, San Martin C, Delgado C. Population attributable fraction of modifiable risk factors for dementia in Chile. *Alzheimers Dement (Amst)* 2022; **14**(1): e12273.
68. Wallace L, Hunter S, Theou O, Fleming J, Rockwood K, Brayne C. Frailty and neuropathology in relation to dementia status: the Cambridge City over-75s Cohort study. *Int Psychogeriatr* 2021; **33**(10): 1035-43.
69. Weiss J. Contribution of socioeconomic, lifestyle, and medical risk factors to disparities in dementia and mortality. *SSM Popul Health* 2021; **16**: 100979.
70. Woo J, Wong M. Targeting mid-life risk factors to reduce late-life dementia. *Public Health* 2014; **128**(10): 952-4.
71. Wu Y, Zheng H, Xu F, et al. Population attributable fractions for risk factors and disability burden of dementia in Jiangxi Province, China: a cross-sectional study. *BMC Geriatr* 2022; **22**(1): 811.
72. Xu W, Tan L, Wang HF, et al. Meta-analysis of modifiable risk factors for Alzheimer's disease. *J Neurol Neurosurg Psychiatry* 2015; **86**(12): 1299-306.
73. Zhang B, Zhang LS, Cheng GR, et al. Urban-rural differences in population attributable fractions for risk dementia factors. [Chinese]. *Chinese Journal of Disease Control and Prevention* 2021; **25(7)**: 854-63.
74. Zhang Y, Chen SD, Deng YT, et al. Identifying modifiable factors and their joint effect on dementia risk in the UK Biobank. *Nat Hum Behav* 2023; **7**(7): 1185-95.
